# miR-434-3p and DNA hypomethylation co-regulate eIF5A1 to increase AChRs and to improve plasticity in SCT rat skeletal muscle

**DOI:** 10.1038/srep22884

**Published:** 2016-03-11

**Authors:** Fei-Fei Shang, Qing-Jie Xia, Wei Liu, Lei Xia, Bao-Jiang Qian, Ling You, Mu He, Jin-Liang Yang, Ting-Hua Wang

**Affiliations:** 1 Institute of Neurological Disease, State Key Laboratory of Biotherapy and Cancer Center, West China Hospital, Sichuan University, and Collaborative Innovation Center for Biotherapy, Chengdu, 610041, P. R. China; 2 Institute of Neuroscience, Kunming medical University, Kunming, 650031, P.R. China; 3 Department of Neurosurgery, West China Hospital, Sichuan University, Chengdu, 610041, P. R. China; 4 Department of Anesthesiology and Translational Neuroscience Center, West China Hospital, Sichuan University, Chengdu, 610041, P. R. China

## Abstract

Acetylcholine receptors (AChRs) serve as connections between motor neurons and skeletal muscle and are essential for recovery from spinal cord transection (SCT). Recently, microRNAs have emerged as important potential biotherapeutics for several diseases; however, whether miRNAs operate in the modulation of AChRs remains unknown. We found increased AChRs numbers and function scores in rats with SCT; these increases were reduced following the injection of a eukaryotic translation initiation factor 5A1 (eIF5A1) shRNA lentivirus into the hindlimb muscle. Then, high-throughput screening for microRNAs targeting eIF5A1 was performed, and miR-434-3p was found to be robustly depleted in SCT rat skeletal muscle. Furthermore, a highly conserved miR-434-3p binding site was identified within the mRNA encoding eIF5A1 through bioinformatics analysis and dual-luciferase assay. Overexpression or knockdown of miR-434-3p *in vivo* demonstrated it was a negative post-transcriptional regulator of eIF5A1 expression and influenced AChRs expression. The microarray-enriched Gene Ontology (GO) terms regulated by miR-434-3p were muscle development terms. Using a lentivirus, one functional gene (map2k6) was confirmed to have a similar function to that of miR-434-3p in GO terms. Finally, HRM and MeDIP-PCR analyses revealed that DNA demethylation also up-regulated eIF5A1 after SCT. Consequently, miR-434-3p/eIF5A1 in muscle is a promising potential biotherapy for SCI repair.

No proven therapeutic modality for spinal cord injury (SCI) has emerged over the last several decades^1^. The drugs that are administered directly into the spinal cord in the clinic are far from satisfactory, and treatment options are limited. Despite the fact that injured axons fail to spontaneously regenerate after SCI, partial recovery of locomotor function (termed plasticity) occurs in mammals^2^,^3^,^4^,^5^. The underlying mechanisms of this phenomenon may involve neuron-muscle circuitry remodeling^6^,^7^. However, in view of current progress, most studies have focused on spinal neuroplasticity; thus, little is known concerning changes to the skeletal muscles, to which drugs could be easily delivered after cord injury.

The neuromuscular junction (NMJ) is a critical relay between motor neurons and skeletal muscles^8^. Released acetylcholine (Ach) binds to its receptors (AChRs) and stimulates muscle contractions^9^. AChRs affect motor functions and are expected to be affected by cord transection. Among the five subunits of AChRs, the γ-subunit (Chrng) plays an important role in original AChRs formation during muscle development^10^. Therefore, understanding how AChRs respond to SCI and how their responses can be modulated is important not only for reducing motor deficits but also for optimizing functional recovery.

MicroRNAs (miRNAs) are endogenously encoded, evolutionarily conserved small RNAs that regulate gene expression predominantly at the post-transcriptional level^11^. Thus, miRNAs can be considered possible biological drugs. Although direct evidence for the role of miRNAs in SCI is scarce, several miRNAs involved in neural development, regeneration and astrogliosis have begun to be recognized^12^. Emerging evidence has also demonstrated that miRNA sequences can regulate skeletal myogenesis by controlling the processes of myoblast proliferation and differentiation^13^. From a clinical perspective, different studies have clearly shown that miRNAs serve as potent therapeutic tools for several diseases^12^. Therefore, the identification of robust miRNAs in skeletal muscle is a promising direction for SCI treatment.

Here, we report that miR-434-3p targets eukaryotic translation initiation factor 5A1 (eIF5A1) to weaken locomotor function by decreasing the number of AChRs in the skeletal muscle of rats that have undergone spinal cord transection (SCT). During this process, miR-434-3p primarily regulates genes associated with muscle development. eIF5A1 is essential for the synthesis of a subset of proteins that contain proline stretches; this factor has been implicated in multiple functions, including cell survival, differentiation and proliferation^14^,^15^,^16^,^17^,^18^,^19^. We confirmed the DNA demethylation of eIF5A1 in hindlimb muscles after SCT. Our findings suggest that the miR-434-3p pathway plays a pivotal role in the recovery of locomotor function after SCI. Thus, targeting miR-434-3p to skeletal muscle provides a novel therapeutic strategy for SCI repair.

## Results

### Changes in AChRs and locomotor functions after SCT

**Figure 1:**
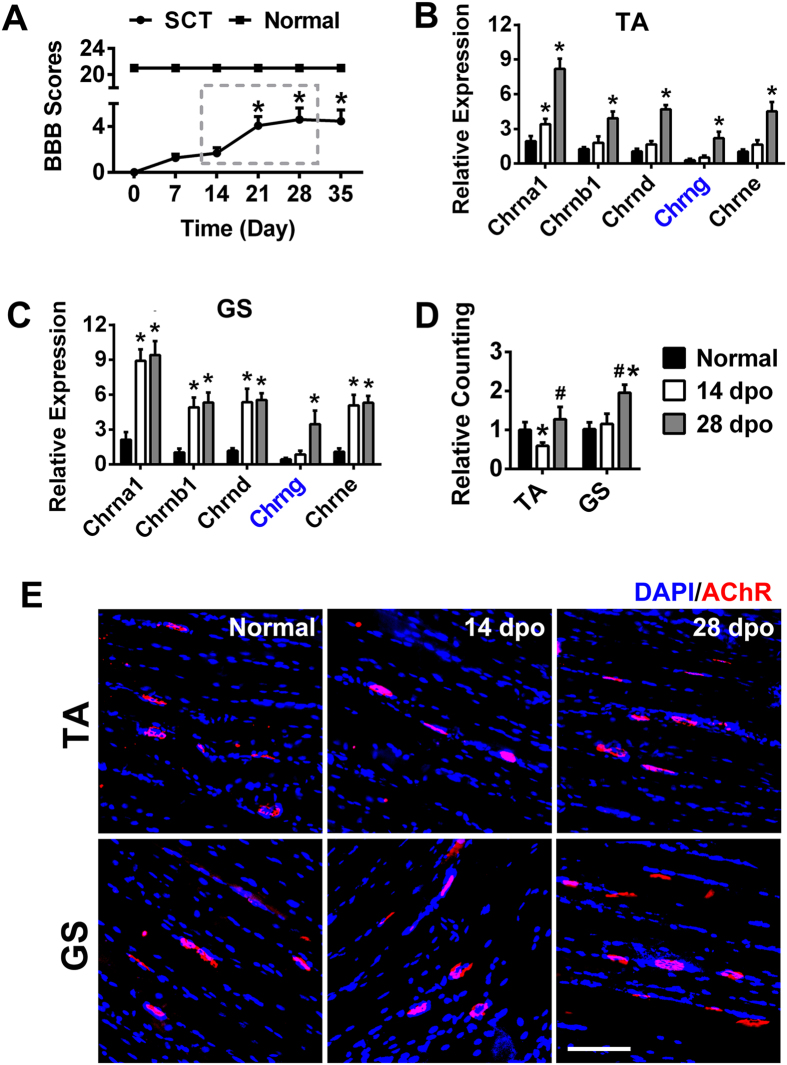
AChRs number changed dramatically in the SCT rat hindlimb muscles concordant with spontaneous hindlimb locomotor function recovery. (**A**) Partial functional recovery was observed in SCT rats. After 21 dpo, the BBB scores exhibited significant increases compared to 0 dpo. The normal group rats were not subjected to any surgical procedures, with an average score of 21 ± 0.00 throughout the experiment. Two groups exhibited significant differences. *P < 0.05 compared with 0 dpo. (**B,C**) The five AChRs subunits in the TA and GS were detected by RT-PCR. All of the subunits were up-regulated after injury. At 14 dpo, Chrng increased mildly. Its expression rapidly increased at 28 dpo. Note that the subunits increased more slowly in the TA compared to the GS at 14 dpo. *P < 0.05 compared with the normal group. (**D,E**) Labeling of AChRs (red) was accomplished using rhodamine-conjugated α-BTX on normal and SCT rat skeletal muscle. Nuclei were visualized by DAPI (blue). The number of AChRs decreased in the TA at 14 dpo and then increased at 28 dpo. The number of AChRs in the GS did not change at 14 dpo but increased significantly at 28 dpo. Quantification of AChRs density is shown in (D). *P < 0.05 compared with the normal group; ^#^P < 0.05 compared with 14 dpo. Scale bar = 100 μm. TA, tibialis anterior; GS, gastrocnemius; dpo, days post operation. n = 8–10.

Basso, Beattie, and Bresnahan (BBB) scores were used to observe changes in hindlimb locomotor function. The BBB scores exhibited a gradual increase from the day of the spinal cord transection through 0, 7, 14, 21, 28 and 35 dpo (days post operation), which shows that spontaneous functional recovery occurred after SCT. The scores increased rapidly from 14 dpo to 28 dpo. The scores of normal control (ctrl) rats remained at 21 ± 0.00 throughout the experiment (Fig. 1A).

The following five classes of muscle-type ACh receptor subunits have been identified: Chrna 1 (α1), Chrnb 1 (β1), Chrng (γ), Chrne (ε) and Chrnd (δ). In fetal muscle, the receptor composition is (α1)_2_β1γδ, whereas in adult muscle the composition is (α1)_2_β1εδ. During muscle development, the ε-subunit replaces the γ-subunit to form the adult receptor in combination with the α1-, β1-, and δ-subunits^10^,^20^. We investigated the expression levels of these five subunits in the tibialis anterior (TA) and gastrocnemius (GS) muscles of SCT rats (Fig. 1B,C). The results showed that denervation stimulated the transcription of AChRs subunits in SCT rat skeletal muscles. Compared with the other subunits, Chrng showed the lowest expression level in the normal group. After SCT, Chrng expression increased mildly at 14 dpo. However, its expression increased sharply at 28 dpo in both the TA and GS. Because Chrng is a necessary subunit for AChRs development, its up-regulation at 28 dpo probably promoted AChRs formation. This hypothesis is consistent with our later results showing that the number of AChRs increased at 28 dpo (Fig. 1D). We also noted that the numbers of the four mature subunits (α1β1εδ) increased less in the TA than in the GS at 14 dpo, representing a possible explanation for the reduction in the number of AChRs in the TA in SCT rats 14 dpo (Fig. 1D). The low levels of these four mature subunits in the TA cannot be maintained in the adult AChRs, which explains a previous report that destabilized AChRs were present in the TA but not in the GS 2 weeks after SCI^21^. Next, we used rhodamine-conjugated α-bungarotoxin (BTX) (red) to label AChRs in the TA and GS (Fig. 1E). The results showed that the number of AChRs decreased in the TA at 14 dpo and then increased at 28 dpo. In the GS, the number of AChRs remained unchanged at 14 dpo but rose significantly at 28 dpo. These results explained the following phenomenon. The ankle motion associated with TA, which acts to dorsiflex and invert the foot, showed extremely slight recovery until 28 dpo after SCT^2^,^3^,^4^,^5^,^22^,^23^. However, knee movement, which involves the GS, showed significant recovery from 14 dpo. The quantification of AChRs density is shown in Fig. 1D.

### Increased numbers of AChRs are regulated by eIF5A1 after spinal cord transection (SCT)

**Figure 2:**
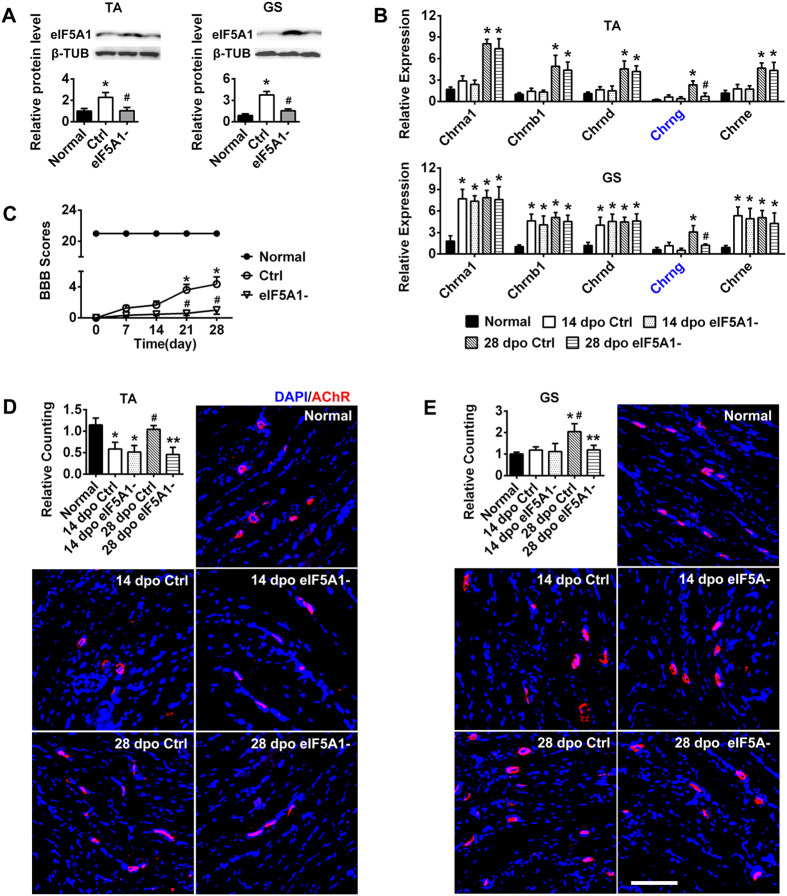
eIF5A1 down-regulation blocks AChRs up-regulation, thus impairing the recovery of hindlimb motor function after SCT. (**A**) Western blot analysis indicated that eIF5A1 expression was up-regulated in the ctrl group but down-regulated by the shRNA lentivirus in the GS and TA of SCT rats. β-TUB was used as an internal ctrl. The x-axis showed the average fold change relative to the normal group. *P < 0.05 compared with the normal group; ^#^P < 0.05 compared with the ctrl group. (**B**) After eIF5A1 interference, the expression levels of the AChRs subunits in the TA and GS were detected by RT-PCR. Consistent with Fig. 1B,C, the subunits were up-regulated in the ctrl group. In the eIF5A1 inhibition group, Chrna 1 (α1), Chrnb 1 (β1), Chrnd (δ) and Chrne (ε) changed only slightly. However, Chrng (γ) decreased rapidly at 28 dpo. *P < 0.05 compared with the normal group; ^#^P < 0.05 compared with the ctrl group. (**C**) The functional scores of the hindlimb indicated poor restoration after eIF5A1 suppression in SCT rat skeletal muscle. The scores of normal rats were consistently 21 ± 0.00 throughout the 28 d. The scores of the ctrl rats ranged from 0 to 6. In contrast, the scores of the SCT + shRNA lentivirus (eIF5A1−) rats never exceeded one. Compared to the normal group, each group (Ctrl and eIF5A1−) showed significant differences (P < 0.05). *P < 0.05 compared with 0 dpo of the ctrl group; ^#^P < 0.05 compared with the corresponding dpo of the ctrl group. (**D,E**) AChRs were labeled with α-BTX (red) on the hindlimb TA and GS sections. The numbers of AChRs decreased as eIF5A1 was down-regulated 28 dpo but were not changed at 14 dpo compared with the ctrl group. The ctrl group displayed results similar to the SCT rats (Fig. 1D). Quantification of AChRs density is shown with a column diagram. *P < 0.05 compared with the normal group; **P < 0.05 compared with the ctrl group; ^#^P < 0.05 compared with 14 dpo. Scale bar = 100 μm. TA, tibialis anterior; GS, gastrocnemius; dpo, days post operation; ctrl, control group; eIF5A1-, SCT + eIF-5A1- shRNA lentivirus; β-TUB, β-tubulin. n = 710.

eIF5A1 can promote hindlimb motor function recovery in GS after SCT^23^. However, the underlying mechanisms of this effect have remained elusive, including the identity of the molecule(s) that regulate it and the cell component(s) affected by its expression. Therefore, a shRNA lentivirus targeting eIF5A1 was injected into the hindlimb skeletal muscle immediately after SCT. Significant suppression of the eIF5A1 protein level was observed in the TA and GS at 28 dpo compared to the ctrl group (Fig. 2A).

We tested hindlimb motor function using the BBB scale from 0 dpo to 28 dpo. The use of blind scoring ensured that the observers were not aware of the treatments received by each rat. As shown in Fig. 1A, the ctrl rats exhibited spontaneous functional recovery, whereas this recovery was blocked by eIF5A1 down-regulation in shRNA lentivirus-injected SCT rats (Fig. 2C).

In the preceding results, Chrng was the only subunit expressed rapidly in the hindlimb muscle at 28 dpo but not at 14 dpo (Fig. 1B,C). The number of AChRs also increased at 28 dpo (Fig. 1D,E). We measured the expression of AChRs subunits in the TA and GS after eIF5A1 was inhibited. The subunits were up-regulated in the ctrl group, which was consistent with previous results (Fig. 1B,C). In the eIF5A1 inhibition group, the expression of Chrna 1, Chrnb 1, Chrnd and Chrne was slightly altered. However, Chrng expression decreased rapidly at 28 dpo, indicating that Chrng is an important downstream molecular target of eIF5A1. As predicted, following the inhibition of eIF5A1 expression, the number of AChRs in the TA and GS decreased; specifically, Chrng was down-regulated at 28 dpo compared with the ctrl group (Fig. 2D,E). Moreover, the ctrl group displayed results similar to those observed for the SCT rats (Fig. 1D). This experiment confirmed that eIF5A1 regulated the γ-subunit to increase AChRs formation and promote the SCT rats’ functional recovery.

### High-throughput screening identifies miR-434-3p as a direct target of eIF5A1

After SCT, the sequence of functional recovery was from the hip (near the spinal cord) to the ankle (far from the spinal cord)^2^,^3^,^4^,^5^,^22^,^23^. The TA acts to dorsiflex and invert the foot. However, the ankle showed extremely slight recovery after SCT until 28 dpo. Generally, the knee exhibited significant recovery from 14 dpo and served as a major contributor to hindlimb movement recovery. The GS can assist in knee motion; therefore, we focused primarily on the GS after SCT in the next experiment.

**Figure 3:**
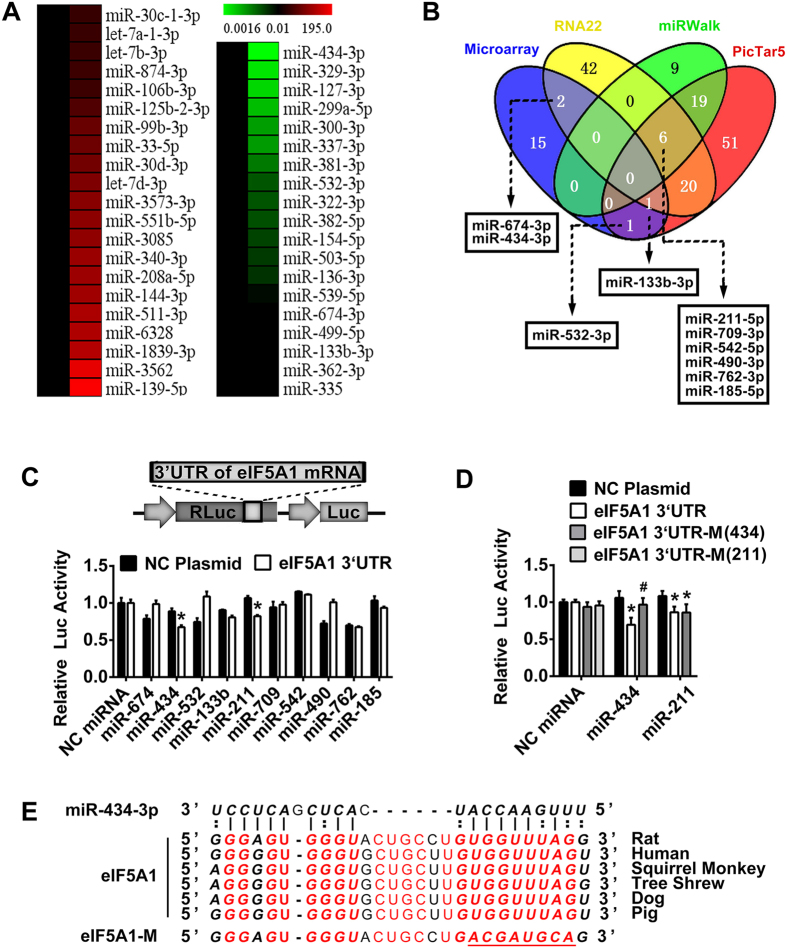
The conserved sequence of the eIF5A1 3′ UTR is a target of miR-434-3p. (**A**) Compared with the normal group, the microarray detected 21 enriched miRNAs (left list) and 19 depleted miRNAs (right list) in the GS of SCT rats. Only miRNAs with expression levels altered more than 2-fold were selected. (**B**) Nineteen depleted miRNAs (microarray) and three algorithms were used to predict the target miRNA of eIF5A1. Four depleted miRNAs were predicted by the software. Six additional miRNAs were predicted by all algorithms but were not depleted miRNAs. These 10 miRNAs were noted. (**C**) 293T cells were transfected with the luciferase constructs of the wild type eIF5A1 3′ UTR plasmid or NC (negative ctrl) plasmid together with the 10 miRNA mimics or NC miRNA. Then, luciferase activity was analyzed. miR-434-3p and miR-211-5p suppressed eIF5A1 translation. The upper image shows a partial plasmid profile. *Renilla* luciferase (RLuc) was used as the reporter, and firefly luciferase (Luc) was used as the ctrl. *P < 0.05 compared with the NC plasmid. (**D**) Luciferase reporter vector harboring the 3′ UTR of the eIF5A1 mRNA (eIF5A1 3′ UTR, wild type; eIF5A1 3′ UTR-M (434), mutated miR-434-3p target; eIF5A1 3′ UTR-M (211), mutated miR-211 target) or the NC plasmid were co-transfected into 293T cells together with the miR-434-3p, miR-211-5p or NC miRNA mimics. After the target was mutated, the miR-434-3p group showed significant recovery of luciferase activity. This result confirmed that miR-434-3p was the miRNA that targets the eIF5A1 3′ UTR. *P < 0.05 compared with the NC plasmid. ^#^P < 0.05 compared with the eIF5A1 3′ UTR. (**E**) The miR-434-3p binding site in the 3′ UTR region of eIF5A1 was highly conserved among several species. Red letters indicate the conserved sequence of eIF5A1. Bold letters indicate the target of miR-434-3p. The underline shows the mutation of the miR-434-3p target in eIF5A1.

Figure 2A shows that eIF5A1 was up-regulated at 28 dpo. To identify miRNAs with functional relevance in hindlimb recovery after SCT, we profiled miRNAs from the GS of normal and SCT rats at 28 dpo, with a specific emphasis on identifying miRNAs that negatively associated with eIF5A1 expression. We identified 719 miRNAs using microarray hybridization (shown in Supplementary miRNA microarray information). Next, the data were filtered stringently to include only miRNAs with at least either 2-fold enrichment or 2-fold depletion in the skeletal muscle; this strategy identified 21 enriched miRNAs and 19 depleted miRNAs (Fig. 3A). Then, three algorithms (PicTar 5, RNA 22, and miRwalk)^24^,^25^,^26^ were used to predict the target miRNA of eIF5A1. Using this integrative strategy, 4 depleted miRNAs were predicted by the software. miR-532-3p, which was down-regulated after SCT, was predicted by PicTar 5. miR-133b-3p was predicted by PicTar 5 and RNA 22. miR-674-3p and miR-434-3p were also included in the depleted miRNAs and predicted by RNA 22. Six additional miRNAs were predicted by all the algorithms but were not included in the 19 depleted miRNAs (Fig. 3B).

Next, we proceeded to validate which miRNAs were genuine targets of the mRNA encoding eIF5A1. The full-length 3′ UTR of the transcript was cloned downstream of a luciferase gene in a reporter vector (Fig. 3C). The vector, in combination with miRNA mimics, was co-transfected into 293T cells, and luciferase activity was monitored 48 h later. Robust decreases in luciferase activity were observed in the miR-434-3p and miR-211-5p groups; the negative ctrl (NC) miRNA had no effect on luciferase activity (Fig. 3C). Next, we tested whether the predicted miR-434-3p and miR-211-5p binding sites located within the eIF5A1 3′ UTR (wild type) were responsible for the miRNA-mediated inhibition of reporter gene expression. Accordingly, point mutations were introduced into the wild type eIF5A1 3′ UTR to abolish the predicted seed pairing of miR-434-3p and miR-211-5p. Upon co-transfection of the resulting mutant eIF5A1 3′ UTR-M, the miR-434-3p group showed restored luciferase activity compared with the wild type. However, the miR-211-5p group did not significantly recover luciferase activity (Fig. 3D). These results suggested that the repressive effect of miR-434-3p on the wild type eIF5A1 3′ UTR was mediated via a single, highly conserved binding site (Fig. 3E).

**Figure 4:**
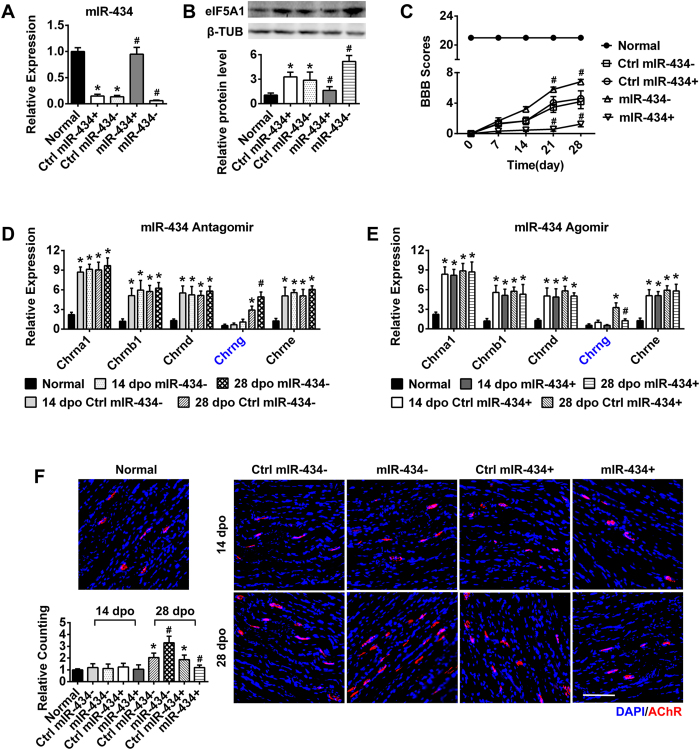
miR-434-3p down-regulates AChRs to impair motor function after SCT. (**A**) The miR-434-3p antagomir (miR-434−) and agomir (miR-434+) were injected into the GS of SCT rats. PCR showed that the miR-434-3p levels were successfully down- or up-regulated in the miR-434-3p interference group (miR-434−) or overexpression group (miR-434+), respectively, compared with the ctrl rats at 28 dpo. Ctrl rats exhibited decreased miR-434-3p levels compared with the normal rats. *P < 0.05 compared with the normal group; ^#^P < 0.05 compared with the ctrl group. (**B**) As anticipated, immunoblotting demonstrated that eIF5A1 negatively associated with miR-434-3p expression. Ctrl rats showed increased eIF5A1 protein levels. Compared with the ctrl, eIF5A1 was up-regulated significantly in the miR-434- group and down-regulated significantly in the miR-434 + group at 28 dpo. *P < 0.05 compared with the normal group; ^#^P < 0.05 compared with the ctrl group. (**C**) BBB scores showed that miR-434-3p changed the motor function of SCT rats. The scores of ctrl rats were lower than the miR-434-3p- rats but higher than the miR-434-3p + rats (P < 0.05). In the normal group, the BBB scores were 21 ± 0.00 throughout the experiment. ^#^P < 0.05 compared with the corresponding dpo of the ctrl group. (**D,E**) After miR-434-3p regulation, AChRs subunits in the GS were detected by RT-PCR. Consistent with Fig. 1B,C, the subunits were up-regulated in the ctrl group. Chrna 1, Chrnb 1, Chrnd and Chrne were changed slightly in the miR-434-3p- and miR-434-3p + groups. However, Chrng increased or decreased rapidly at 28 dpo. *P < 0.05 compared with the normal group; ^#^P < 0.05 compared with the ctrl group. (**F**) AChRs were labeled with α-BTX (red) on GS sections. The numbers of AChRs increased or decreased in agreement with the down- or up-regulation, respectively, of miR-434-3p at 28 dpo but didn’t change at 14 dpo compared with the ctrl group. The ctrl group displayed results similar to the SCT rats (Fig. 1D). Quantification of AChRs density is shown with a column diagram. *P < 0.05 compared with the normal group; ^#^P < 0.05 compared with the ctrl group. Scale bar = 100 μm; dpo, days post operation; ctrl, control group; β-TUB, β-tubulin. n = 8–10.

We further verified that miR-434-3p expression negatively associated with eIF5A1 expression. At 28 dpo, eIF5A1 expression was up-regulated in the GS (Figs 2B and 4B), whereas miR-434-3p expression was down-regulated (Fig. 4A) compared with normal rats. Fig. 4B also shows that eIF5A1 was up-regulated in the miR-434-3p interference group (miR-434−) but was down-regulated in the miR-434-3p overexpression group (miR-434+).

### miR-434-3p can increase AChRs formation to promote locomotor functions in SCT rat skeletal muscle

After confirming that eIF5A1 is a target of miR-434-3p, we investigated whether targeting miR-434-3p was an effective mechanism for promoting AChRs formation *in vivo*. For this purpose, we injected either a miR-434-3p agomir (miR-434+) or an antagomir (miR-434−) into the hindlimb skeletal muscle of SCT rats. Then, we confirmed the changes in miR-434-3p expression at 28 dpo. Real-time PCR indicated that the miR-434-3p levels were significantly up-regulated in the miR-434-3p overexpression group (miR-434+) but significantly down-regulated in the miR-434-3p interference group (miR-434−) compared with the ctrl rats (Fig. 4A). Immunoblot analysis indicated that eIF5A1 expression negatively associated with miR-434-3p regulation (Fig. 4B).

Then, we tested hindlimb motor function using the BBB scale from 0 dpo to 28 dpo. The scores of the ctrl rats were lower than those of rats in the miR-434-3p interference group but higher than those of rats in the miR-434-3p overexpression group. In the normal group, the BBB scores were 21 ± 0.00 throughout the experiment.

As mentioned above, Chrng combines with other subunits to form mature AChRs^10^. After determining miR-434-3p regulation, we measured the expression levels of the AChRs subunits in the GS. In the ctrl group, the results were consistent with Fig. 1B,C. After receiving injections of the miR-434-3p antagomir or agomir, the levels of Chrna 1, Chrnb 1, Chrnd and Chrne were altered slightly. However, Chrng increased or decreased rapidly at 28 dpo (Fig. 4D,E). The AChRs staining results (Fig. 4F) were similar to those observed for eIF5A1 regulation. At 28 dpo, miR-434-3p interference up-regulated eIF5A1 expression and increased AChRs formation, whereas its overexpression down-regulated eIF5A1 and decreased AChRs formation. The results from the ctrl groups were consistent with previous experiments with SCT rats (Fig. 1D,E). These experiments further confirmed that miR-434-3p targets eIF5A1 to deteriorate functional recovery *in vivo*.

### Gene expression profile survey of downstream molecular events triggered by miR-434-3p

**Figure 5:**
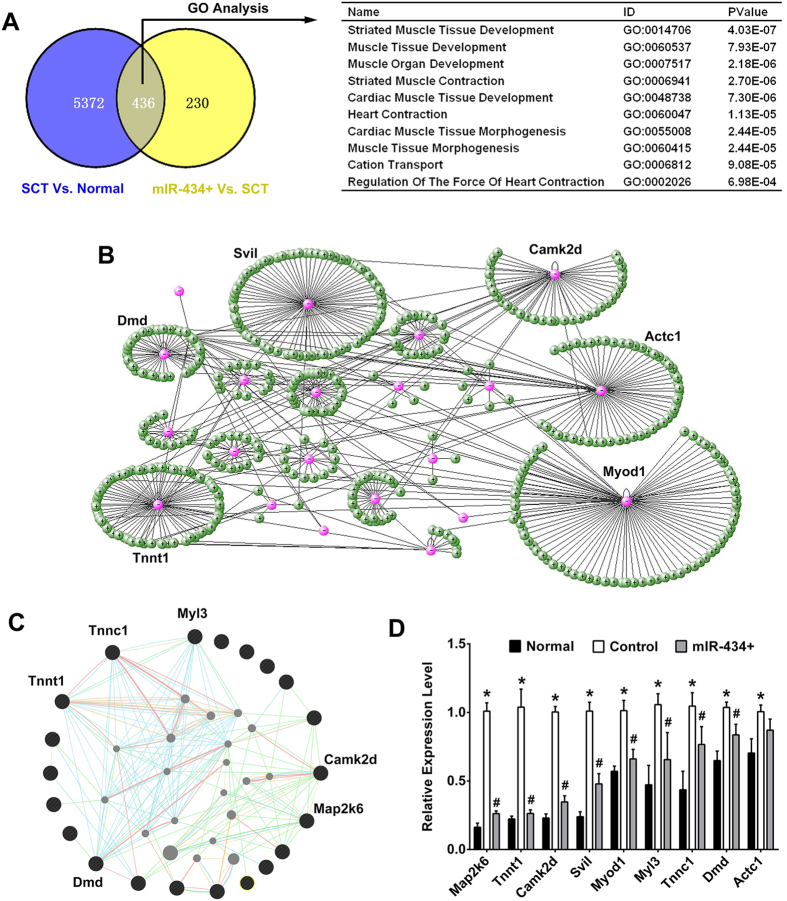
Gene expression profile and bioinformatics analysis to explore the downstream molecules triggered by miR-434-3p. (**A**) Left, Venn diagram showing miR-434-3p regulation. The microarray identified 5808 genes that were significantly enriched more than 2-fold in the SCT rats vs. normal rats and 666 genes that were depleted in the miR-434-3p overexpression rats (miR-434+) vs. SCT rats. Among these genes, 436 genes were highly expressed in the SCT group concordant with miR-434-3p down-regulation and minimally expressed in the miR-434-3p + group. Right table, GO analysis showing that the 436 genes triggered by miR-434-3p were enriched significantly in muscle development and morphogenesis terms. (**B**) The genes enriched in the top 3 GO terms were used as inputs for online analysis tools to discover their molecular interaction. VisANT displayed the relationships among these genes (pink nodes) and their interacting genes (green nodes). Myod1, Svil, Actc1, Camk2d, Tnnt1 and Dmd were found to have abundant interactions. (**C**) GeneMANIA was also used to find genes that were related to the input genes (black nodes). This tool identified Map2k6, Camk2d, Tnnc1, Tnnt1, Dmd and Myl3 as having abundant interactions. (**D**) Real-time PCR confirmed the expression levels of 9 potential central genes in the normal, ctrl and miR-434-3p + groups. Consistent with the microarray results, all of the genes were up-regulated in the ctrl rats and down-regulated in the miR-434-3p + group. However, no significant difference in Actc1 expression was observed between the ctrl and miR-434-3p + groups. *P < 0.05 compared with the normal group; ^#^P < 0.05 compared with the ctrl group.

In the present study, we confirmed that miR-434-3p targets eIF5A1 to regulate AChRs formation. However, the molecular events that participate in this process remained unknown. Therefore, we attempted to address this question using gene expression microarrays. In the GS of SCT rats vs. normal rats, 5808 genes were significantly enriched more than 2-fold, whereas 4428 genes were depleted. We confirmed that miR-434-3p is down-regulated after SCT. Upon miR-434-3p overexpression, we identified 666 down-regulated genes and 813 up-regulated genes compared with SCT rats (Supplementary mRNA microarray information). Among these genes, 436 genes with high expression in the SCT rats vs. normal rats were expressed at low levels in the miR-434-3p + rats vs. SCT rats (Fig. 5A). This finding indicated that these genes are downstream molecules of miR-434-3p. Next, these genes were analyzed using the DAVID program^27^. Gene Ontology (GO) analysis is a functional analysis that associates differentially expressed genes with GO categories. The results showed that the differentially expressed genes were significantly enriched in muscle development and morphogenesis terms (Fig. 5B). Thus, miR-434-3p plays an important role in maintaining skeletal muscular function after SCI.

The genes enriched in the top 3 GO terms were used as inputs for online analysis tools to discover their molecular interactions. VisANT displayed the relations among these genes (pink nodes) and their potential interacting genes (green nodes)^28^. Myod1, Svil, Actc1, Camk2d, Tnnt1 and Dmd have abundant interactions (Fig. 5B). Using an extremely large set of functional association data, GeneMANIA also found genes that are related to the input genes (black nodes)^29^. Specifically, this tool indicated that Map2k6, Camk2d, Tnnc1, Tnnt1, Dmd and Myl3 have abundant interactions with their potential interacting genes. (Fig. 5C).

A gene with abundant interactions is thought to make a significant contribution to functional recovery. Some of these genes, such as Myod1, Camk2d and Dmd, have been verified to benefit AChRs structure^30^,^31^,^32^. We measured the expression levels of these genes in the normal, ctrl and miR-434-3p + groups using real-time PCR. Consistent with the microarray results, all of these genes were up-regulated in the ctrl rats but down-regulated in the miR-434-3p + group (Fig. 5D). However, no significant differences were detected for Actc1 between the ctrl and miR-434-3p + groups.

### Of the GO terms triggered by miR-434-3p, Map2k6 was identified as a functional gene that could enhance AChRs formation to promote functional recovery

**Figure 6:**
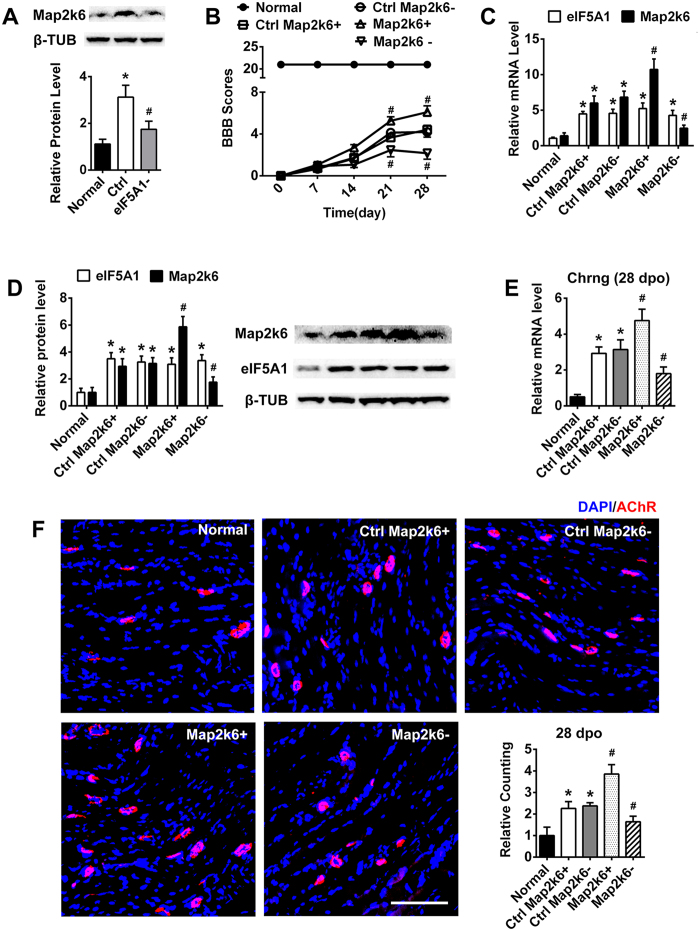
The predicted gene Map2k6 up-regulates AChRs and improves motor function after SCT. (**A**) Map2k6 expression positively associated with eIF5A1 in the GS. Immunoblotting indicated the Map2k6 was up-regulated in SCT rats (ctrl) and down-regulated by eIF5A1 interference (eIF5A1−). *P < 0.05 compared with the normal group; ^#^P < 0.05 compared with the ctrl group. (**B**) After Map2k6 overexpression (Map2k6+) or suppression (Map2k6−) in the GS of SCT rats by lentivirus infection, the BBB scores indicated enhanced or impaired hindlimb functional recovery, respectively. Map2k6− group obtained significantly lower scores at 21 dpo and 28 dpo. In contrast, Map2k6 + group obtained significantly higher scores. Each group showed significant differences compared to the normal group (P < 0.05). ^#^P < 0.05 compared with the corresponding dpo of the ctrl group. (**C**) RT-PCR determined the expression levels of eIF5A1 and Map2k6 after Map2k6 lentiviral infection. Map2k6 was up- or down-regulated significantly. eIF5A1 was overexpressed after SCT, and lentiviral infection had no effect on eIF5A1 expression. *P < 0.05 compared with the normal group; ^#^P < 0.05 compared with the ctrl group. (**D**) Immunoblotting indicated the expression of eIF5A1 and Map2k6 following Map2k6 regulation. The result showed the same trend as the PCR. *P < 0.05 compared with the normal group; ^#^P < 0.05 compared with the ctrl group. (**E**) After SCT, the transcription of Chrng was up-regulated in the ctrl group. Map2k6− resulted in a decrease in the Chrng mRNA level at 28 dpo. Map2k6 + also significantly enhanced Chrng expression. *P < 0.05 compared with the normal group; ^#^P < 0.05 compared with the ctrl group. (**F**) Compared with normal rats, the ctrl group rats exhibited a higher density of AChRs at 28 dpo. The same was true for the BBB scores. The AChRs displayed the expected low density in the Map2k6− group and high density in the Map2k6 + group compared with the ctrl rats. Quantification of AChRs density is shown subsequently. The x-axis showed the average fold change relative to the normal group. *P < 0.05 compared with the normal group; ^#^P < 0.05 compared with the ctrl group. Scale bar = 100 μm. ctrl, control group; β-TUB, β-tubulin.

As mentioned above, the mRNA levels of 9 factors were analyzed by RT-PCR. The mRNA levels of Map2k6, a mitogen-activated protein (MAP) kinase kinase, changed most rapidly, which was consistent with miR-434-3p regulation (Fig. 5D). Map2k6 activates p38 to promote cell proliferation and survival^33^. To date, a function for Map2k6 in SCI or AChRs formation has not been reported. We confirmed that Map2k6 is regulated by miR-434-3p (Fig. 5D). Next, we tested the Map2k6 translation level following eIF5A1 down-regulation by shRNA lentivirus. Surprisingly, Map2k6 expression positively associated with eIF5A1 in the rat GS. The Map2k6 level in the SCT group was increased compared with the normal group and decreased after the down-regulation of eIF5A1 at 28 dpo (Fig. 6A). Moreover, eIF5A1 was up-regulated in SCT rats but remained unaffected when Map2k6 was down- or up-regulated by a lentiviral vector (Fig. 6C,D). Thus, eIF5A1 is an upstream molecule of Map2k6.

To further study the role of Map2k6, a lentivirus vector was used to regulate the expression of Map2k6 in the GS of SCT rats. Map2k6 shRNA or an overexpression sequence was packed into the lentivirus vector. Four groups of SCT rats were administered the Map2k6 shRNA lentivirus (Map2k6−), Map2k6 expression lentivirus (Map2k6+), NC shRNA lentivirus (Ctrl Map2k6−) or NC expression lentivirus (Ctrl Map2k6+). Then, the expression levels of Map2k6 were evaluated using real-time PCR and immunoblotting. After transduction with the lentivirus at 28 dpo, the mRNA and protein levels of Map2k6 in the GS of SCT rats were significantly up-regulated in the Map2k6 overexpression group (Map2k6+) but significantly down-regulated in the Map2k6 interference group (Map2k6−) (p < 0.05) compared with the ctrl group (Fig. 6C,D). Therefore, Map2k6 was successfully regulated by the lentiviral vector in the GS.

Next, each group of 8 rats was tested for locomotor function recovery using BBB scores from 0 dpo to 28 dpo. The data showed that Map2k6− could reduce the recovery of locomotor function because rats in this group exhibited significantly worse scores. In contrast, Map2k6+, which resulted in Map2k6 overexpression in the GS of SCT rats, increased the BBB scores significantly (Fig. 6B). After measuring the BBB scores 28 dpo, the rat GS tissues were harvested and fixed in 4% paraformaldehyde. Then, the muscle was sliced, stained for AChRs, and observed under a fluorescence microscope. Similar to miR-434-3p regulation in the GS (Fig. 4F), the up- or down-regulation of Map2k6 was consistent with the BBB scores and with significant enhancement or inhibition of AChRs formation, respectively, in the SCT rats. Moreover, we detected the expression level of Chrng by real-time PCR. Chrng forms the original receptor in combination with α1-, β1-, and δ-subunits^10^,^20^. SCT stimulated the transcription of Chrng, and Map2k6− and Map2k6 + down- and up-regulated Chrng expression, respectively. These results suggested that Map2k6 is a downstream signaling molecule of miR-434-3p/eIF5A1 that stimulates AChRs formation to promote functional recovery.

### In addition to miR-434-3p, DNA hypomethylation also regulates eIF5A1 expression after SCT

**Figure 7:**
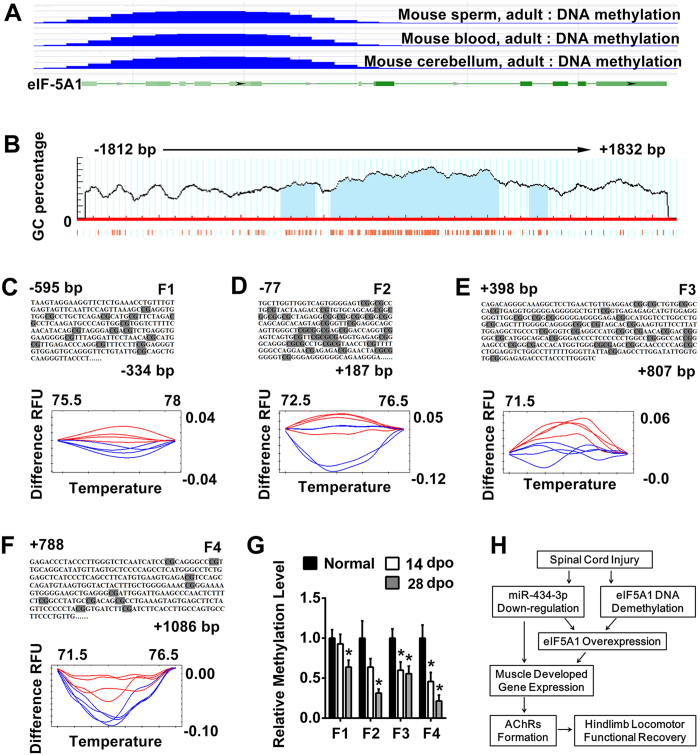
eIF5A1 subjected to DNA demethylation is up-regulated in the GS of SCT rats. (**A**) The NCBI epigenomics database showed that eIF5A1 DNA was methylated in three individual tissues of the normal adult mouse. The green line represents the DNA sequence of eIF5A1. The green rectangle represents the exon, and the light green rectangle represents the 5′ UTR of the mRNA. The blue lines indicate the 5-methylcytosine cluster level. (**B**) eIF5A1 promoter and near regions covering 3653 bp (−1812 bp to + 1832 bp) of DNA sequence contain three predicted 5′-CpG islands (filled light blue areas). The red fine bars under the x-axis panels indicate the isolated CpG dinucleotides. The figure was adapted from MethPrimer software. (**C–F**) The methylation–sensitive high-resolution melting analysis (MS-HRM) assay was used to assess the methylation of the eIF5A1 gene in DNA samples. In the predicted DNA methylated area, 4 segments (−595 bp to −334 bp, −77 bp to + 187 bp, + 398 bp to + 807 bp and + 788 bp to + 1086 bp) were designed as bisulfite conversion primers to perform the HRM assay. The upper panels indicate the sequence of the detected segment. The lower panels were created by Precision Melt Analysis software (Bio-Rad, USA). Compared with the eIF5A1 methylation levels in the GS from normal rats (red curve), the values for the SCT rats (blue curve) were lower. (**G**) Immunoprecipitation (MeDIP)-qPCR confirmed the DNA methylation levels of 4 segments. F1-4 correspond to (**C–F**), respectively. The MeDIP-qPCR analysis showed that the GS DNA methylation of the eIF5A1 was decreased in SCT rats compared to the normal group. *P < 0.05 compared with the normal group. (**H**) A schematic model for the promotion of AChRs that induced functional recovery in SCT rats and its underlying mechanism. After SCT, eIF5A1 is subjected to DNA hypomethylation in the hindlimb muscle. The miRNA targeting eIF5A1 (miR-434-3p) was down-regulated. These actions led to an increase in eIF5A1 levels. Then, the expression of muscle development genes was enhanced. These genes, such as Map2k6, promoted AChRs formation and aided the functional recovery in SCT rats. dpo, days post operation.

Notably, the miR-434-3p levels in SCT rats nearly reached the levels of the normal group after they received the miR-434-3p agomir injection (miR-434 + group) (Fig. 4A). In contrast, the eIF5A1 level in the miR-434 + group did not decrease to that of the normal rats (Fig. 4B). miRNAs generally inhibit mRNA translation without affecting the mRNA levels. However, the eIF5A1 mRNA level was up-regulated after SCT^23^ (Fig. 6C). Therefore, we hypothesized the existence of other mechanisms that could influence eIF5A1 transcription. To investigate the mechanism underlying eIF5A1 up-regulation, we focused on DNA demethylation. DNA hypomethylation changes protein-DNA interactions, leading to alterations in chromatin structure and the transcription rate^34^. The NCBI database showed that mass DNA methylated regions exist in the eIF5A1 5′ UTR of adult mouse tissues (sperm, cerebellum and blood) (Fig. 7A). Thus, we selected a 3643 bp (−1812 bp to + 1831 bp) DNA sequence from the rats’ eIF5A1 5′ UTR and predicted 3 potential CpG islands using MethPrimer software^35^ (Fig. 7B).

Next, we measured the DNA methylation level of eIF5A1 in the SCT rat model. DNA methylation occurs on cytosine residues, particularly on CpG dinucleotides enriched in small regions. Incubating the target DNA with sodium bisulfite can result in the conversion of unmethylated cytosine residues into uracil, while leaving the methylated cytosines unchanged. Therefore, PCR products resulting from an originally unmethylated template will have a lower melting point than those derived from a methylated template. To investigate whether the DNA methylation levels of eIF5A1 were altered in the GS, a validation experiment was performed to compare the melting curves of the normal and SCT groups using high-resolution melting (HRM) analysis. DNA methylated regions are enriched in CG islands, so the primers must not target CGs. The DNA was broken into small fragments (almost hundreds of bases) by sodium bisulfite. We selected 7 regions to design primers that eliminated “CG”. Finally, the PCR results for 4 primer pairs obtained specific amplified products. The HRM curves of these 4 regions are shown in Fig. 7C–F. These curves signified their methylation levels. Significant demethylation of eIF5A1 was observed in the GS at 28 dpo (blue curve) compared with normal rats (red curve). The corresponding DNA sequence information is displayed above the curves (Fig. 7C–F).

To confirm the methylation level, we employed methylated DNA immunoprecipitation (MeDIP), followed by qPCR analysis. The MeDIP analysis showed that GS DNA methylation of the eIF5A1 promoter was decreased in SCT rats compared to the normal group (Fig. 7G). These results indicated another mechanism by which eIF5A1 expression was regulated by DNA hypomethylation in the SCT rat skeletal muscle.

## Discussion

At present, an estimated 2.5 million people suffer from spinal cord injuries (SCI)^36^. As the most severe SCI, SCT leads to a complete loss of communication between the brain and muscle. However, the lower central nervous system (spinal cord) contains neurons that control movement^7^. The bulk of evidence has shown that partial spontaneous functional recovery can occur in subjects with SCT^2^,^3^,^4^,^5^. Therefore, we hypothesized that spinal cord neurons and associated skeletal muscle play a pivotal role in functional recovery. AChRs, the connections between motor neurons and skeletal muscle, control our ability to move and breath, and thus are essential for survival^37^. In this study, we confirmed that AChRs numbers were altered sharply in the skeletal muscle of SCT rats. Surprisingly, miR-434-3p can decrease AChRs numbers and therefore may serve as a potential therapeutic agent for SCI.

Motor nerve terminals release the neurotransmitter ACh, which diffuses across the synaptic cleft and binds to AChRs that stimulate muscle contractions^37^. The original AChRs composition is (α1)_2_β1γδ. Burns AS *et al.* reported that some destabilized AChRs were present selectively in the TA but not the GS 2 weeks after SCI^21^. However, the reason for this phenomenon was elusive. Here, we attempted to address this question through our experiments and showed in results. Finally, our results showed that the AChRs subunits, particularly the γ-subunit, play important roles in movement function recovery after SCT.

eIF5A1 contains the unusual amino acid hypusine. It can stimulate the differentiation and proliferation of various cells^16^. The protein levels of eIF5A1 differ from organ to organ. Tissues at the differentiation stage have a higher eIF5A1 content^38^. The γ-subunit of AChRs is also an important subunit during muscle development^10^,^20^. We measured the expression levels of the five subunits in the eIF5A1 knockdown group and observed that the expression of Chrng decreased rapidly. This finding indicates that Chrng is a crucial molecule downstream of eIF5A1. Moreover, we found that miR-434-3p, which targets eIF5A1, could regulate muscle development genes. These genes could represent important proteins that function in morphological and functional improvement of damaged tissue. Above all, the appropriate promotion of some development-related genes is a potential treatment for SCI or other traumatic injuries.

miRNAs regulate protein expression at the post-transcriptional level by decreasing transcript abundance or by inhibiting protein translation. In addition to miR-434-3p, other miRNAs that play important roles in skeletal muscle have also been identified using miRNA microarrays. Among these miRNAs, miR-499 enhances the expression of the β-myosin heavy chain, which promotes muscle development^39^. Let-7 mutants exhibit temporal misregulation of NMJ maturation^40^. Colangelo V *et al.* reported that miR-874 is involved in muscle differentiation^13^. Moreover, the fine coordination of activity of the transmembrane receptor smoothened by the miR-30 family allows the correct specification and differentiation of distinct muscle cell types during zebrafish embryonic development^41^. Interestingly, the miR-133 family, together with miR-1, miR-206 and miR-208, is specifically expressed in muscle; thus, these miRNAs are called myomiRs^42^. However, another experiment showed that the miR-206/133b cluster is dispensable for the development, survival and regeneration of skeletal muscle. Moreover, miR-133a, which differs from miR-133b by only one nucleotide, is crucial for early embryonic survival^43^.

After altering miR-434-3p expression, we identified muscle development GO terms that are regulated by miR-434-3p. Nine central molecules associated with these terms might be involved in functional recovery after SCT. Some of these molecules have been confirmed to contribute to muscle development and physiological functions. Troponin T (Tnnt), troponin I (Tnni), and troponin C (Tnnc) form the troponin complex, which, together with tropomyosin (Tm), acts as a regulatory switch for muscle contraction. Tnnt and myosin light chain (Myl) perform helpful functions during the development or denervation of muscle tissue^44^,^45^,^46^. Calcium/calmodulin-dependent protein-kinase II (Camk2d) is a ubiquitous enzyme that modulates the functions of many neuronal proteins in response to changes in intracellular calcium concentrations. Chun-Jen Lin C *et al.* reported that Camk2d enhanced the motor axon diameter and increased the synapse numbers at the larval neuromuscular junction^32^. Moreover, myogenic differentiation (Myod) deletion resulted in major alterations in the organization of the neuromuscular junction and was linked to perturbations in the nuclear localization of β-catenin^31^. Myod can also differentially promote the activation and repression of chromatin structures at myogenic genes early after the onset of skeletal muscle differentiation in the developing mouse embryo^47^. In dystrophin (Dmd) mutated mice, AChRs clusters were reduced in number and exhibited structural fragmentation^30^. Together, these factors may jointly promote neuron-muscle circuitry remodeling after SCI.

Many biological processes are not only determined by the DNA code itself but also regulated by activity-related chromosomal remodeling^34^,^48^. DNA promoter methylation is generally associated with reduced gene activity. In contrast, demethylation increases gene expression^49^. Previous studies have shown that DNA demethylation induced by 5-azacytidine (5AC) through epigenetic mechanisms plays an important role in reprogramming the murine C2C12 mesenchymal progenitor cell transcriptome to promote skeletal myotube maturation^50^. DNA methylation has been viewed historically as a static process following neural development and cell differentiation^51^. Neural and skeletal muscle development, regeneration, and repair are also mediated by changes in DNA methylation^52^. Accordingly, DNA methylation levels may also play a critical role in spontaneous locomotor function recovery in SCT rats.

In clinical applications, hundreds of miRNAs have been reported to be involved in disease development and progression. Many miRNAs, such as miR-122 (Santaris Pharma, Denmark) and miR-34 (Mirna Therapeutics, USA), need to be studied clinically^53^. However, the greatest challenge for nucleic acid drugs is designing *in vivo* delivery strategies. Depending on the disease and target tissues, different strategies will need to be carefully considered to achieve the desired delivery of oral or intravenous drugs. Because drug carriers cannot perfectly deliver drugs to target tissues, on-target side effects may result from interference with the miRNAs that are expressed in normal tissues. In addition, most oligonucleotides are easily taken up by the liver and kidney and are rapidly excreted in urine. In addition, the oligonucleotide dose required for *in vivo* inhibition is often high (~80 mg per kg for antagomirs), which increases the risk of off-target effects^53^. In our treatment strategy, miR-434-3p was injected directly into the target tissue, which avoided these problems.

The next step of this research is the assessment of drug safety, especially for long term injections. Overdoses of miR-434-3p agomirs and antagomirs may impair recovery of function (Figure S2A and S2B). The correct clinical therapeutic dose needs to be ensured, and some miRNAs have similar seed regions. Under physiological conditions, anti-miRs (antagomirs) are generally unable to distinguish between the miRNAs within the same family, especially those with identical seed regions^53^. This problem should also be considered and assessed before clinical research is conducted.

## Methods

### Animals

All animal procedures were approved by Sichuan University Committee on Animal Research, and the methods were carried out in accordance with the approved guidelines. Adult female Sprague-Dawley rats weighing 200–250 g (Animal Breeding Center of the Sichuan University, China) were maintained under a standard 12-h light/dark cycle with water and food pellets available ad libitum.

### Surgery and assessment of functional recovery

As described previously^23^, anesthetized (60 mg/kg ketamine and 0.25 mg/kg medetomidine) rats were exposed by laminectomy at T10. The spinal cord was transected using iridectomy scissors and microaspiration. The superficial back muscles were sutured along the midline, and the skin was closed with Michel wound clips. The animals were maintained on twice-daily bladder expression from 0 dpo.

Recovery was evaluated by hindlimb locomotor performance, which was assessed according to the open-field BBB scale as described previously^22^,^23^. The rats were observed and scored from 0 (no observable movements) to 21 (normal locomotion).

### Immunoblotting

Protein extraction and immunoblotting were performed as described by Shang *et al.*^23^. Antibodies directed against the following proteins were used: eIF5A1 (1:5000, Abcam, USA), phosphorylated map2k6 (1:1000, Abcam, USA), and β-tubulin loading ctrl (β-TUB, 1:2000, GeneTex, USA). HRP-conjugated secondary antibodies (1:5000, GeneTex, USA) were also used. Blotting was analyzed using ImageJ software. And each sample was normalized to β-TUB.

### Real-time RT-PCR

For gene expression analysis, total RNA was isolated from the muscle tissues of animals using TRIzol reagent (Life Technologies, USA). In total, 2000 ng of RNA was reverse-transcribed to cDNA using a RevertAid First Strand cDNA Synthesis Kit (Thermo Fisher Scientific, USA). PCR was performed using cDNA as a template in a 20 μl reaction mixture containing specific primers (Sangon Biotech, China). The primers are listed in Supplementary Table S1. Each sample was normalized to β-actin. The reaction was performed in a thermal cycler (Bio-Rad CFX96, USA) according to the following standard protocol: one cycle of 95 °C for 3 min, followed by 45 cycles of 95 °C for 15 s, annealing for 20 s, and 72 °C for 30 s. Next, we confirmed that β-actin was a stable reference gene in the gastrocnemius by determining the Ct values of the normal, 14 days post-operation (dpo) and 28 dpo groups, as shown in Supplementary Figure S1.

For microRNA expression analysis, 2000 ng of total RNA was reverse-transcribed by miR-434-3p- or U6 (internal control)- specific RT primer (RiboBio, China) and a RevertAid First Strand cDNA Synthesis Kit (Thermo Fisher Scientific, USA). PCR was performed using a standard protocol with a 20 μl reaction mixture containing specific primers (RiboBio, China). Each sample was normalized to U6.

### Morphological and acetylcholine receptor (AChR) staining

Skeletal muscle was fixed in 4% paraformaldehyde and dehydrated with successive sucrose gradients. Twenty micron thick sections were incubated with rhodamine-conjugated α-BTX (1:400, Life Technologies, USA) for 1 h at 37° After the sections were washed 3 times with PBST, the nuclei were stained with DAPI and images were acquired using a Leica AF6000 cell station (USA).

### High-resolution melting (HRM) analysis

In total, 25 mg of skeletal muscle was harvested from the animals. DNA was extracted using a DNeasy Tissue Kit (Qiagen, USA) according to the manufacturer’s instructions. Incubating the DNA with sodium bisulfite (EpiTect Plus DNA Bisulfite Kit, Qiagen, USA) resulted in the conversion of unmethylated cytosine residues into uracil, while leaving the methylated cytosines unchanged. The methylation status of the samples was distinguished by their melting curves.

HRM analysis enabled the rapid characterization of the DNA samples based on their melting behavior following PCR amplification. We predicted 3 CpG islands in the eIF5A1 DNA sequence of the 5′ UTR^35^ (Fig. 7B). After cytosine is converted to uracil, the DNA sequence is be broken into small fragments (generally less than 500 bp). Therefore, we designed 7 pair primers for HRM analysis (Sangon Biotech, China). Precision Melt Supermix (Bio-Rad, USA) was used to perform HRM according to the manufacturer’s protocol. The program was set as follows: one cycle of 95 °C for 3 min, followed by 40 cycles of 95 °C for 30 s, annealing for 20 s, and 72 °C for 30 s. After sequence extension at 60 °C for 10 min, HRM analysis was performed at 65–95 °C (in 0.2 °C increments). In the pilot experiment, 4 primer pairs (Supplementary Table S2) were designed successfully.

### Methylated DNA immunoprecipitation (MeDIP)

To confirm the methylation levels, MeDIP was performed using 3 μg of DNA isolated from the skeletal muscle. Using an EpiMark Methylated DNA Enrichment Kit (New England Biolabs, USA) according to the manufacturer’s instructions, methylated DNA was isolated from fragmented genomic DNA by binding to the methyl-CpG binding domain of the MBD2 protein that was fused to the Fc tail of IgG1 (MBD2-Fc), which was coupled to paramagnetic hydrophilic protein A beads (MBC2-Fc/Protein A Magnetic Beads). After simple washing steps, followed by magnetic capture, the enriched DNA sample was eluted in nuclease-free water by incubation at 65 °C. Then, the DNA was subjected to standard qPCR. Fragmented DNA untreated by the EpiMark Methylated DNA Enrichment Kit was used as the input ctrl to calculate the relative methylation level (MeDIP/input). The primers are listed in Supplementary Table S3 (Sangon Biotech, China).

### miR-434-3p agomir/antagomir synthesis and administration

miR-434-3p information was obtained from miRBase (ID: MIMAT0005315)^54^. The agomir and antagomir were synthesized by RiboBio (China). After SCT and closure of the back skin, the gastrocnemius was exposed using a scalpel. Four injection sites in each leg were administered a total of 20 μl (20 μM) of the miR-434-3p agomir or antagomir. The injection sites were evenly distributed in the gastrocnemius, and each site received the optimal dose of drug (5 μl per injection site, 5 μl/min) via a glass micropipette. Finally, the skin was closed with clips. The control rats were subjected to the same operation, and scrambled agomir or antagomir sequences were injected as negative controls (RiboBio, China). At 14 days post operation (dpo), identical doses of the drugs were reinjected.

The optimal dose at each injection site was determined, and four doss were used (1 μl, 2.5 μl, 5 μl and 10 μl); the drug concentration was 20 μM. At 28 dpo, the motor function recovery was measured using the BBB score. Supplementary Figure S2A and S2B shows the dose-dependent (1–5 μl) relationship between the drugs and the BBB scores. However, the 10 μl dose was an exception. Overdoses of antagomirs appeared to block recovery of function (Figure S2B). The 10 μl agomir dose also produced a large decrease in the BBB score (Figure S2A) but did not rapidly and proportionally increase the content of miR-434-3p in the gastrocnemius (Figure S2C). Therefore, overdoses might impair recovery of function. Next, a miR-434-3p agomir labeled with FAM or an antagomir labeled with Cy3 was also injected into the gastrocnemius. Then, the gastrocnemius muscle was harvested at 28 dpo, fixed in 4% paraformaldehyde, and dehydrated with successive sucrose gradients. Twenty micron-thick sections were imaged using a Leica AF6000 Cell Station (USA). Supplementary Figure S2D shows that the miR-434-3p agomir and the antagomir were successfully transfected into the skeletal muscle. Of all tested doses, 5 μl per injection site was the optimal dose.

### Lentivirus vector production and administration

First, we designed 4 shRNA sequences for the map2k6 (ID: 114495) lentivirus vector. Then, PC12 cells were cultured in high-sugar medium containing 10% FBS. They were transfected with the expression plasmid or ctrl vector using liposomes, and the effects of shRNA were examined by RT-PCR. The negative control (NC) plasmid was generally applied as the ctrl and theoretically had no effect on any gene. The results revealed that map2k6-shRNA-2 down-regulated the map2k6 mRNA significantly (Supplementary Table S4).

The design of the eIF5A1 shRNA was based on the NCBI database (ID: 287444). The target mRNA sequence was determined as described in a previous report^23^ (Supplementary Table S5). The interference sequence of map2k6-shRNA-2 is also shown in Supplementary Table S5. For map2k6 overexpression, the open reading frame of the plasmid was 1004 bp (Supplementary Table S5). All plasmids were supplied by GeneCopoeia (China). Thereafter, the expression vector and viral packaging system containing an optimized mixture of the two packaging plasmids (GeneCopoeia, China) were co-transfected into 293T cells. The ctrl plasmid was also packaged (designated NC-LV). The cell supernatant was harvested after 48 h and filtered through a 0.45 mm cellulose acetate filter. Then, 5 ml of supernatant containing the lentivirus vector was centrifuged (3500 × g for 25 min). Finally, supernatant containing the lentivirus were frozen at – 80 °C until use.

The methodology for lentivirus injection was similar to that of agomir/antagomir administration. After SCT and closure of the back skin, the skeletal muscle was exposed using a scalpel. Based on the findings of previous reports^55^,^56^,^57^, a total of 200 μl (~2 × 10^9^ TU/ml) of the lentivirus was administered via 8 injection sites in the gastrocnemius, or a total of 100 μl was administered via 4 injection sites in the tibialis anterior of each leg. The injection sites were evenly distributed in the gastrocnemius or tibialis anterior, and each site received 25 μl of drug (5 μl/min) via a glass micropipette. Finally, the skin was closed with clips. A shRNA NC plasmid or an overexpressing NC plasmid that contained scrambled sequences was also administered via a lentiviral vector as a negative control (NC-LV). The control rats were subjected to the same operation and injected with NC-LV. The lentivirus was injected only once at 0 dpo.

### Luciferase reporter assay

The miRNA mimic and eIF5A1 3′ UTR plasmid were supplied by RiboBio (China). 293T cells (5 × 10^4^ per well) were pre-seeded in a 96-well plate 24 h before transfection. Each well was transfected with 1 ng/μl of the 3′ UTR luciferase vector and 50 nM of the miRNA mimic using a FECT transfection kit (RiboBio, China). The assay was performed using the Dual-Luciferase Reporter Assay System (Promega, USA) 48 h after transfection using *Renilla* luciferase as the reporter and firefly luciferase as the ctrl. Luminescence was measured by a Synergy 2 microplate reader (BioTek, USA).

### miRNA microarray

Total RNA was extracted and purified using a mirVana miRNA Isolation Kit (Ambion, Austin, TX, USA), and an RNA integrity number (RIN) was obtained using an Agilent Bioanalyzer 2100 (Agilent Technologies, USA) to evaluate RNA integration. Then, the miRNA was labeled and hybridized using a miRNA Complete Labeling and Hyb Kit (Agilent Technologies, USA). After hybridization, the slides were washed in staining dishes (Thermo Shandon, USA) using a Gene Expression Wash Buffer Kit (Agilent Technologies, USA). The slides were scanned using an Agilent Microarray Scanner (Agilent Technologies, USA) and analyzed with Feature Extraction software 10.7 (Agilent Technologies, USA) using the default settings. Raw data were normalized using the quantile algorithm in Gene Spring Software 11.0 (Agilent Technologies, USA). miRNA microarrays were normalized by quantile normalization according to the Agilent GeneSpring software user manual. Each array contained a certain distribution of expression values, and this method was used to produce identical distributions across various arrays. It was done as follows. After the expression values from various arrays were loaded into a dataset with probesets along the rows, arrays and columns, each column was sorted in increasing order. Next, the value in each row was replaced with the average of the values in that row. Finally, the columns were unsorted (i.e., the effect of the sorting step was reversed so that the items in the column returned to their original positions). This process worked quite well for reducing the variance between arrays. The experiment and data analysis were performed by Shanghai Biotechnology Co., Ltd. (Shanghai, China).

### Bioinformatics analysis

MethPrimer software was utilized to forecast the rats’ eIF5A1 CpG islands^35^. In total, 3643 bp (−1812 bp to + 1831 bp) of the eIF5A1 DNA 5′ UTR was submitted to analysis by the software. The methylated modification was identified at conservative promoters in the mouse by the open-source web-based NCBI epigenomics tool. All information concerning the mRNA and DNA sequences was also obtained from NCBI. RNA22, miRwalk and PicTar5 software were used to predict the miRNA targeting eIF5A1^24^,^25^,^26^. GO analysis was completed using the DAVID program^27^. The gene interaction networks were drawn using GeneMANIA and VisANT^28^,^29^.

### Statistical analysis

Statistical analysis was performed with SPSS version 20.0. Statistical analysis of the data involving more than three groups was performed using one-way ANOVA. Data involving two groups were analyzed using an independent samples t-test. The data are presented as the mean ± SEM. For all analyses, significance was assigned at the P ≤ 0.05 level.

## Additional Information

**How to cite this article**: Shang, F.-F. *et al.* miR-434-3p and DNA hypomethylation co-regulate eIF5A1 to increase AChRs and to improve plasticity in SCT rat skeletal muscle. *Sci. Rep.* **6**, 22884; doi: 10.1038/srep22884 (2016).

## Supplementary Material

Supplementary Information
