# Supplementary material for: miR-434-3p and DNA hypomethylation co-regulate eIF5A1 to increase AChRs and to improve plasticity in SCT rat skeletal muscle
Source: Sci Rep. 2016 Mar 11;6:22884. doi: 10.1038/srep22884 (PMC4786822; doi:10.1038/srep22884)
Supplement: Supplementary Information [file srep22884-s1.pdf]

miR-434-3p and DNA hypomethylation co-regulate eIF5A1 to increase AChRs and to improve plasticity in SCT rat skeletal muscle

Short title: miR-434-3p/eIF5A1 mediated AChRs in SCT rat muscle

Fei-Fei Shang[1,*], Qing-Jie Xia[1,*], Wei Liu[2], Lei Xia[2], Bao-Jiang Qian[4], Ling You[4], Mu He[3], Jin-Liang Yang[1,*,#], Ting-Hua Wang[1, 2, 3, 4,*,#]

[1]Institute of Neurological Disease, State Key Laboratory of Biotherapy and Cancer Center, West China Hospital, Sichuan University, and Collaborative Innovation Center for Biotherapy, Chengdu, 610041, P. R. China

[2]Department of Anesthesiology and Translational Neuroscience Center, West China Hospital, Sichuan University, Chengdu, 610041, P. R. China

[3]Department of Neurosurgery, West China Hospital, Sichuan University, Chengdu, 610041, P. R. China

[4]Institute of Neuroscience, Kunming medical University, Kunming，650031, P.R. China

*These authors contributed equally to this work.

#Corresponding authors:

Ting-Hua Wang, Institute of Neurological Disease, State Key Laboratory of Biotherapy, Department of Anesthesiology and Translational Neuroscience Center, West China Hospital, Sichuan University, P.R. China. E-mail: tinghua_neuron@263.net

Or Jin-Liang Yang, State Key Laboratory of Biotherapy and Cancer Center, West China Hospital, Sichuan University, and Collaborative Innovation Center for Biotherapy. E-mail: jlyang01@163.com

Fig. S1

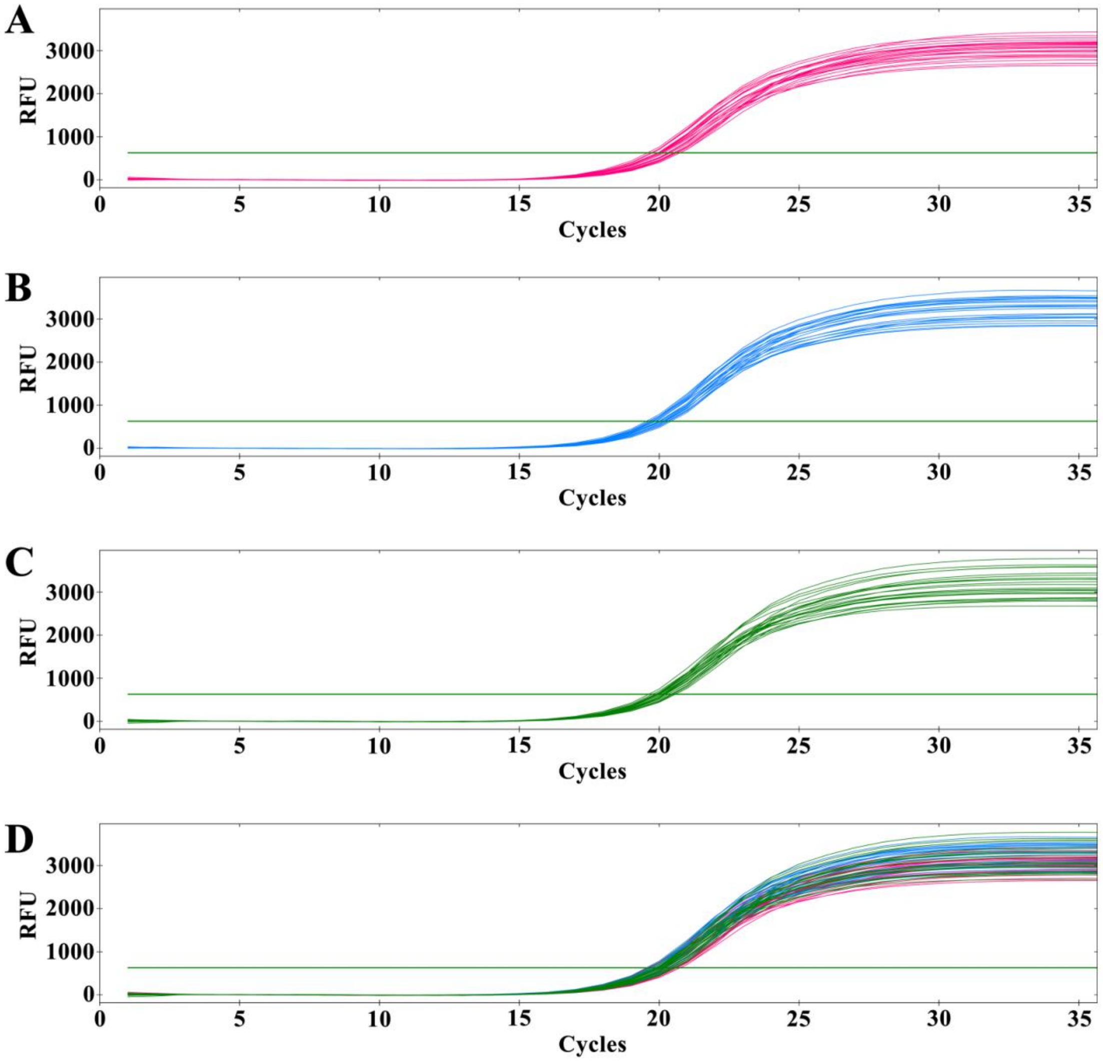

Fig. S1. The Ct values of β-actin in gastrocnemius.

(A) Normal group. (B) SCT 14 days post operation (dpo). (C) SCT 28 dpo. (D) Merged with normal, 14dpo and 28 dpo groups.

Fig. S2

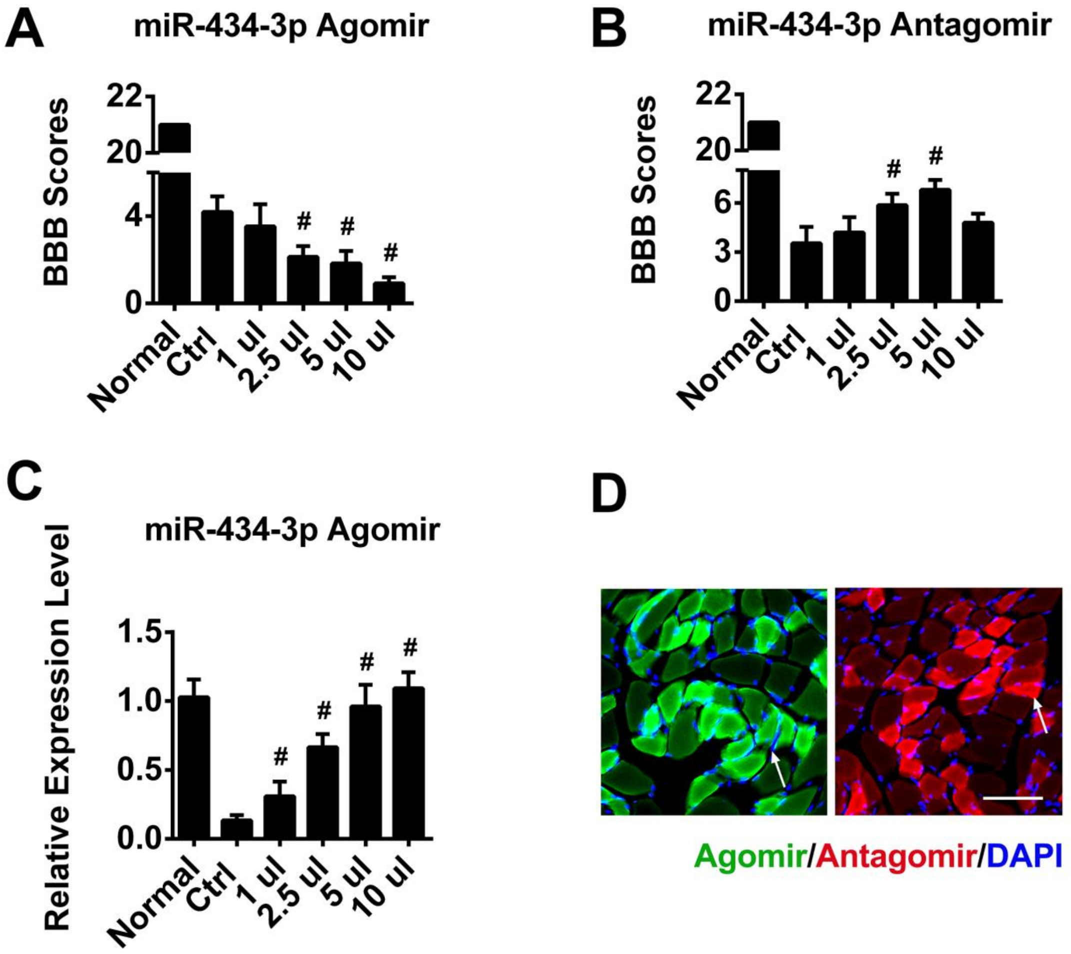

Fig. S2. In condition experiment, the optimal dose of miR-434-3p agomir or antagomir was 5 ul per injection site.

(A, B) After gastrocnemius received 4 gradient dosages of agomir or antagomir (20 μM), the motor function recovery was measured by BBB scores at 28 dpo. Normal group were 21±0.00 (C) After gastrocnemius received 4 gradient dosages of agomir (20 μM), the miR-434-3p was measured by RT-PCR at 28 dpo. (D) miR-434-3p agomir modified by FAM (green) and miR-434-3p antagomir modified by Cy3 (red) were injected in rats' gastrocnemius respectively. The arrow pointed out positive cells transfected successfully. Bar=100 um. Ctrl, SCT group; dpo, days post operation. #, $P<0.05$ compared with the ctrl group.

Table S1. The primers for RT-PCR

Gene	Forward primer	Reverse primer	Length
ACTB	GGTCACCCACACTGTGCCCATCTA	GCCACGCTCGGTCAGGATCTTCAT	118 bp
eIF5A1	GGGAGTGGGTACTGCCTGTGGTTT	AGATGCCTGAGGAGGGATCGTGTC	275 bp
Chrna1	GGCACTTGGACCTATGACGGCTCT	GACGCTGCATGACGAAGTGGTAGG	190 bp
Chrnb1	AGGGAAGGAAGGACATCGTGAGGA	GCCAGCAGCAGCAAGAACACAGT	207 bp
Chrnd	GCCGCAAGCCGCTCTTCTACATCA	CGTGCTGGGTGTTCGGAAGTGGAT	293 bp
Chrne	ACGCCAACGACTCACGCCACAT	TCCAAGTTCCATGCCGATGCCTCT	223 bp
Map2k6	AGCCACGGACTTGACTGGTTGACC	GGACTCCTCGTATGCCTCGCCATT	268 bp
Tnnt1	AGCCGTCCTGTGGTTCCTCCTT	CCTCCTCCTCCTTCCGCATCTTCT	289 bp
Camk2d	CGCCGTGATGGGAAGTGGCAGAAT	CGCCAGGTGCCTTGAGGACAGAAT	204 bp
Svil	AGAGTCTGCTGAACCAGGCGAACC	TGCACCTGCTCCACCTCTCCAAGT	192 bp
Myod1	CGCAACGCCATCCGCTACATTGAA	TCGCTGGACGCCTCACTGTAGTAG	257 bp
Myl3	CCAGCACATGGTCGCAGATCACTC	CGCAGGGTGGAGACAGAAAGGGTA	101 bp
Tnnc1	GATGGCAGTGGCACAGTGGACTTC	AGATCCGACAGCTCCTCCTCAGAC	101 bp
Dmd	CGTCTGAAGCAGCAGCATGAGCAT	GCAGCGAGGTAGAAGGAGAGGACA	280 bp
Actc1	CTGGATTCTGGCGACGGTGTGACT	CGTTCAGCGGTGGTGACAAAGGAG	161 bp

Table S2. The primers of eIF5A1 DNA 5' areas for HRM analysis

NO.	Forward primer	Reverse primer	Length
F1	TAAGTAGGAAGGTTTTTTGAAA	AAAATAACCCTTACAACT	261 bp
F2	TGTTTGGTTGGTTAGTGGGG	TCCCTTCTACCCCCCTCC	264bp
F3	TAGATAGGGTAAAGGTTTTT	AACCCAAAAATAAAATCTCTC	409 bp
F4	AGAGATTTTATTTTTGGGTT	CAACAAAAAAAAACACTAA	299 bp

Underline means the cytosine has been converted to uracil by sodium bisulfite.

Table S3. The primers of eIF5A1 DNA 5' areas for MeDIP analysis

NO.	Forward primer	Reverse primer	Length
F1	TAAGTAGGAAGGTTCTCTGAAA	AGGGTAACCCTTGCAGCT	261 bp
F2	TGCTTGGTTGGTCAGTGGGG	TCCCTTCTGCCCCCCTCC	264bp
F3	CAGACAGGGCAAAGGCTCCT	GACCCAAGGGTAGGGTCTCTC	409 bp
F4	AGAGACCCTACCCTTGGGTC	CAACAGGGAAGGCACTGG	299 bp

Table S4. Determining the effective shRNA fragment of Map2k6 in PC12 cell line.

Group	Mean	Std. Error	P-value
Normal	0.161	0.011	1.000
Control	0.147	0.016	
Map2k6-shRNA- 1	0.092	0.003	0.015
Map2k6-shRNA- 2	0.088	0.006	0.006
Map2k6-shRNA- 3	0.101	0.028	0.745
Map2k6-shRNA- 4	0.177	0.052	1.000

The Map2k6 interference level was compared by RT-PCR, using β-actin as an internal control. Map2k6-shRNA- 2 produced a significant down-regulation compared with Control group (P=0.006).

Table S5. The sequence information of eIF-5A1 and Map2k6 regulation lentiviruses vector.

Code name	Target Sequence
eIF-5A1-shRNA-lentivirus *	GCAAGGAGATTGAGCAGAA
Map2k6-shRNA-lentivirus *	CTACAGTAGTTGACGAGATT
Map2k6-expression-lentivirus *	ATGTCTCAGTCGAAAGGCAAGAAGCGAAACCCCGGCCTTAAGA
	TTCCAAGGGAAGCGTTTGAACAACCTCAGACCAGTTCCACGCCG
	CCTCGGGATTTAGACTCCAAGGCTTGCATTTCTATTGGAAACCA
	GAACTTTGAGGTGAAGGCCGATGACTTGGAGCCTATAGTGGAGC
	TGGGACGAGGTGCGTACGGGGTGGTGGAGAAGATGCGTCACGT
	GCCCAGCGGGCAGATCATGGCGGTGAAGCGGATACGGGCCACA
	GTTAATAGCCAGGAGCAGAAACGGCTACTGATGGATCTGGATGT
	TTCTATGAGGACAGTGGACTGTCCGTTTACCGTGACCTTCTATGG
	TGCACTCTTCCGGGAGGGCGATGTGTGGATCTGCATGGAGCTCA
	TGGATACGTCACTAGATAAATTCTACAAACAAGTTATTGATAAA
	GGCCAAACAATTCCAGAAGATATCTTAGGGAAGATAGCAGTTTC
	TATTGTAAAAGCATTAGAACATTTACACAGTAAGCTCTCTGTTA
	TCCATCGAGATGTCAAGCCTTCAAATGTGCTCATTAACACGCTG
	GGCCAAGTGAAGATGTGTGACTTTGGAATCAGCGGCTACCTGGT
	AGACTCTGTTGCGAAAACGATCGATGCCGGTTGCAAACCATACA
	TGGCTCCTGAACGAATAAATCCAGAGCTCAACCAGAAGGGGTA
	CAGTGTGAAGTCTGACATTTGGAGTCTGGGCATCACCATGATTG
	AGCTGGCCATCCTCCGGTTTCCCTATGATTCTTGGGGAACGCCCT

	TCCAGCAGCTAAAACAGGTGGTAGAAGAGCCGTCTCCACAACTC CCAGCGGACAAGTTCTCTGCTGACTTCGTTGACTTTACCTCACAG TGCTTGAAGAAAAATTCCAAAGAACGGCCCACGTATCCAGAGCT TATGCAACATCCATTTTTCACCGTCCATGAAGCCAAAGCAGCAG ATGTGGCGTCTTTCGTAAAACTGATACTCGGGGACTAG

* eIF-5A1-shRNA-lentivirus, the lentiviruses vector could interfere mRNA of eIF-5A1 was produced by viral packaging system. This effective sequence was obtained from our preceding report. *Map2k6-shRNA-lentivirus, the lentiviruses vector could interfere mRNA of Map2k6 was produced by viral packaging system. This is the most effective sequence code No.2 (Table S4.). *Map2k6-expression-LV, the lentiviruses vector could expression mRNA of Map2k6 was produced by viral packaging system.

Supplementary miRNA microarray information

Systematic Name	Fold change	chr	mirbase_accession_No
rno-let-7a-1-3p	44.63317711	chr7	MIMAT0017085
rno-let-7a-5p	0.916283535	chr8	MIMAT0000774
rno-let-7b-3p	45.38653041	chr7	MIMAT0004705
rno-let-7b-5p	0.755545739	chr7	MIMAT0000775
rno-let-7c-1-3p	0.95035801	chr11	MIMAT0017087
rno-let-7c-5p	0.881316426	chr7	MIMAT0000776
rno-let-7d-3p	98.31297324	chr17	MIMAT0000563
rno-let-7d-5p	1	chr17	MIMAT0000562
rno-let-7e-3p	0.95035801	chr1	MIMAT0004706
rno-let-7e-5p	0.926435858	chr1	MIMAT0000777
rno-let-7f-1-3p	0.95035801	chr17	MIMAT0017089
rno-let-7f-2-3p	0.95035801	chrX	MIMAT0017090
rno-let-7f-5p	0.936538365	chr17	MIMAT0000778
rno-let-7i-3p	0.95035801	chr7	MIMAT0004707
rno-let-7i-5p	1.052277344	chr7	MIMAT0000779
rno-miR-1-3p	1	chr18	MIMAT0003125
rno-miR-1-5p	0.95035801	chr18	MIMAT0003162
rno-miR-100-3p	0.95035801	chr8	MIMAT0017112
rno-miR-100-5p	0.947463979	chr8	MIMAT0000822
rno-miR-101a-3p	1.425541461	chr5	MIMAT0000823
rno-miR-101a-5p	0.95035801	chr5	MIMAT0004726
rno-miR-101b-3p	1.1431545	chr1	MIMAT0000615
rno-miR-101b-5p	0.95035801	chr1	MIMAT0017045
rno-miR-103-1-5p	0.95035801	chr10	MIMAT0017114
rno-miR-103-2-5p	0.95035801	chr3	MIMAT0017113
rno-miR-103-3p	0.784177743	chr3	MIMAT0000824
rno-miR-105	0.95035801	chrX	MIMAT0012825
rno-miR-106b-3p	47.33581642	chr12	MIMAT0004727
rno-miR-106b-5p	1.294586532	chr12	MIMAT0000825
rno-miR-107-3p	1.1154795	chr1	MIMAT0000826
rno-miR-107-5p	0.95035801	chr1	MIMAT0017115
rno-miR-10a-3p	0.95035801	chr10	MIMAT0004709
rno-miR-10a-5p	0.916943344	chr10	MIMAT0000782
rno-miR-10b-3p	0.95035801	chr3	MIMAT0017092
rno-miR-10b-5p	1.131512875	chr3	MIMAT0000783
rno-miR-1188-3p	0.95035801	chr6	MIMAT0017855
rno-miR-1188-5p	0.95035801	chr6	MIMAT0017854
rno-miR-1193-3p	0.95035801		MIMAT0017859
rno-miR-1193-5p	0.95035801		MIMAT0017858
rno-miR-122-3p	0.95035801	chr18	MIMAT0017116
rno-miR-122-5p	0.95035801	chr18	MIMAT0000827

rno-miR-1224	1.028686944	chr11	MIMAT0012827
rno-miR-124-3p	0.95035801	chr2	MIMAT0000828
rno-miR-124-5p	0.95035801	chr2	MIMAT0004728
rno-miR-1249	1.537374027	chr7	MIMAT0017892
rno-miR-125a-3p	1.096864032	chr1	MIMAT0004729
rno-miR-125a-5p	1	chr1	MIMAT0000829
rno-miR-125b-1-3p	0.95035801	chr8	MIMAT0004730
rno-miR-125b-2-3p	61.968219	chr11	MIMAT0026467
rno-miR-125b-5p	0.690715259	chr8	MIMAT0000830
rno-miR-126a-3p	0.783594404	chr3	MIMAT0000832
rno-miR-126a-5p	1	chr3	MIMAT0000831
rno-miR-126b	0.95035801	chr3	MIMAT0017843
rno-miR-127-3p	0.002903821	chr6	MIMAT0000833
rno-miR-127-5p	0.95035801	chr6	MIMAT0017117
rno-miR-128-1-5p	0.95035801	chr13	MIMAT0017118
rno-miR-128-2-5p	0.95035801	chr8	MIMAT0017119
rno-miR-128-3p	0.624645904	chr8	MIMAT0000834
rno-miR-129-1-3p	0.95035801	chr4	MIMAT0017120
rno-miR-129-2-3p	0.95035801	chr3	MIMAT0000601
rno-miR-129-5p	0.95035801	chr3	MIMAT0000600
rno-miR-1298	0.95035801	chrX	MIMAT0025060
rno-miR-1306-3p	1.37153715	chr11	MIMAT0024852
rno-miR-1306-5p	0.95035801	chr11	MIMAT0024851
rno-miR-130a-3p	0.778767185	chr3	MIMAT0000836
rno-miR-130a-5p	0.95035801	chr3	MIMAT0017121
rno-miR-130b-3p	0.95035801	chr11	MIMAT0000837
rno-miR-130b-5p	0.95035801	chr11	MIMAT0017122
rno-miR-132-3p	0.95035801	chr10	MIMAT0000838
rno-miR-132-5p	0.95035801	chr10	MIMAT0017123
rno-miR-133a-3p	1.247887606	chr18	MIMAT0000839
rno-miR-133a-5p	0.951981208	chr18	MIMAT0017124
rno-miR-133b-3p	0.404179485	chr9	MIMAT0003126
rno-miR-133b-5p	0.95035801	chr9	MIMAT0017205
rno-miR-134-3p	0.95035801	chr6	MIMAT0017125
rno-miR-134-5p	0.95035801	chr6	MIMAT0000840
rno-miR-135a-3p	0.95035801	chr7	MIMAT0004732
rno-miR-135a-5p	0.95035801	chr7	MIMAT0000841
rno-miR-135b-3p	0.95035801	chr13	MIMAT0017043
rno-miR-135b-5p	0.95035801	chr13	MIMAT0000611
rno-miR-136-3p	0.008330714	chr6	MIMAT0004733
rno-miR-136-5p	0.95035801	chr6	MIMAT0000842
rno-miR-137-3p	0.95035801	chr2	MIMAT0000843
rno-miR-137-5p	0.95035801	chr2	MIMAT0017126
rno-miR-138-1-3p	0.95035801	chr8	MIMAT0004734

rno-miR-138-2-3p	0.95035801	chr19	MIMAT0017127
rno-miR-138-5p	0.95035801	chr8	MIMAT0000844
rno-miR-139-3p	1.104704874	chr1	MIMAT0004735
rno-miR-139-5p	194.2667654	chr1	MIMAT0000845
rno-miR-140-3p	1	chr19	MIMAT0000574
rno-miR-140-5p	0.903843	chr19	MIMAT0000573
rno-miR-141-3p	0.95035801	chr4	MIMAT0000846
rno-miR-141-5p	0.95035801	chr4	MIMAT0017128
rno-miR-142-3p	0.921095086	chr10	MIMAT0000848
rno-miR-142-5p	0.95035801	chr10	MIMAT0000847
rno-miR-143-3p	0.858888643	chr18	MIMAT0000849
rno-miR-143-5p	0.95035801	chr18	MIMAT0017129
rno-miR-144-3p	123.6892835	chr10	MIMAT0000850
rno-miR-144-5p	0.95035801	chr10	MIMAT0017130
rno-miR-145-3p	0.95035801	chr18	MIMAT0017131
rno-miR-145-5p	0.96527124	chr18	MIMAT0000851
rno-miR-146a-3p	0.95035801	chr10	MIMAT0017132
rno-miR-146a-5p	0.887714089	chr10	MIMAT0000852
rno-miR-146b-3p	0.95035801	chr1	MIMAT0017334
rno-miR-146b-5p	0.662271463	chr1	MIMAT0005595
rno-miR-147	0.95035801	chr3	MIMAT0005297
rno-miR-148b-3p	0.70513725	chr7	MIMAT0000579
rno-miR-148b-5p	0.95035801	chr7	MIMAT0004645
rno-miR-150-3p	1.562544308	chr1	MIMAT0017133
rno-miR-150-5p	0.817662284	chr1	MIMAT0000853
rno-miR-151-3p	0.95035801	chr7	MIMAT0000614
rno-miR-151-5p	1.099569656	chr7	MIMAT0000613
rno-miR-152-3p	1.031679175	chr10	MIMAT0000854
rno-miR-152-5p	0.95035801	chr10	MIMAT0017134
rno-miR-153-3p	0.95035801	chr6	MIMAT0000855
rno-miR-153-5p	0.95035801	chr6	MIMAT0017135
rno-miR-154-3p	0.95035801	chr6	MIMAT0017136
rno-miR-154-5p	0.007799823	chr6	MIMAT0000856
rno-miR-15b-3p	0.95035801	chr2	MIMAT0017093
rno-miR-15b-5p	0.732401829	chr2	MIMAT0000784
rno-miR-16-3p	0.95035801	chr2	MIMAT0017094
rno-miR-16-5p	0.861987588	chr2	MIMAT0000785
rno-miR-17-1-3p	0.95035801	chr15	MIMAT0004710
rno-miR-17-2-3p	0.95035801	chrX	MIMAT0017295
rno-miR-17-5p	1.179616003	chr15	MIMAT0000786
rno-miR-181a-1-3p	0.95035801	chr13	MIMAT0000884
rno-miR-181a-2-3p	0.95035801	chr3	MIMAT0017138
rno-miR-181a-5p	0.89302471	chr3	MIMAT0000858
rno-miR-181b-1-3p	0.95035801	chr13	MIMAT0017139

rno-miR-181b-2-3p	0.95035801	chr3	MIMAT0017140
rno-miR-181b-5p	1.015117691	chr3	MIMAT0000859
rno-miR-181c-3p	0.95035801	chr19	MIMAT0017137
rno-miR-181c-5p	0.95035801	chr19	MIMAT0000857
rno-miR-181d-3p	0.95035801	chr19	MIMAT0017296
rno-miR-181d-5p	0.95035801	chr19	MIMAT0005299
rno-miR-182	0.95035801	chr4	MIMAT0005300
rno-miR-183-3p	0.95035801	chr4	MIMAT0017141
rno-miR-183-5p	0.95035801	chr4	MIMAT0000860
rno-miR-1839-3p	139.7376429	chr1	MIMAT0024844
rno-miR-1839-5p	1.693977485	chr1	MIMAT0024843
rno-miR-184	0.95035801	chr8	MIMAT0000861
rno-miR-1843-3p	0.95035801	chr6	MIMAT0024848
rno-miR-1843-5p	0.95035801	chr6	MIMAT0024847
rno-miR-185-3p	0.95035801	chr11	MIMAT0017142
rno-miR-185-5p	1.055072399	chr11	MIMAT0000862
rno-miR-186-3p	0.95035801	chr2	MIMAT0017143
rno-miR-186-5p	1.156527042	chr2	MIMAT0000863
rno-miR-187-3p	0.95035801	chr18	MIMAT0000864
rno-miR-187-5p	0.95035801	chr18	MIMAT0017144
rno-miR-188-3p	0.95035801	chrX	MIMAT0017297
rno-miR-188-5p	1	chrX	MIMAT0005301
rno-miR-18a-3p	0.95035801	chr15	MIMAT0017095
rno-miR-18a-5p	0.95035801	chr15	MIMAT0000787
rno-miR-190a-3p	0.95035801	chr8	MIMAT0017145
rno-miR-190a-5p	0.95035801	chr8	MIMAT0000865
rno-miR-190b-3p	0.95035801	chr2	MIMAT0017298
rno-miR-190b-5p	0.95035801	chr2	MIMAT0005302
rno-miR-1912-3p	0.95035801	chrX	MIMAT0017865
rno-miR-1912-5p	0.95035801	chrX	MIMAT0017864
rno-miR-191a-3p	0.708865538	chr8	MIMAT0017146
rno-miR-191a-5p	0.95035801	chr8	MIMAT0000866
rno-miR-191b	0.95035801	chr8	MIMAT0017817
rno-miR-192-3p	0.95035801	chr1	MIMAT0017147
rno-miR-192-5p	0.980729174	chr1	MIMAT0000867
rno-miR-193-3p	1.480931592	chr10	MIMAT0000868
rno-miR-193-5p	1.362405345	chr10	MIMAT0004736
rno-miR-194-3p	0.95035801	chr1	MIMAT0017148
rno-miR-194-5p	1.018258107	chr1	MIMAT0000869
rno-miR-1949	0.756275454	chr18	MIMAT0017852
rno-miR-195-3p	0.95035801	chr10	MIMAT0017149
rno-miR-195-5p	0.753468968	chr10	MIMAT0000870
rno-miR-196a-3p	0.95035801	chr7	MIMAT0004737
rno-miR-196a-5p	1.042616069	chr7	MIMAT0000871

rno-miR-196b-3p	0.95035801	chr4	MIMAT0017171
rno-miR-196b-5p	0.976094883	chr4	MIMAT0001082
rno-miR-196c-3p	0.95035801	chr10	MIMAT0017299
rno-miR-196c-5p	1.086795653	chr10	MIMAT0005303
rno-miR-199a-3p	0.896613421	chr13	MIMAT0004738
rno-miR-199a-5p	1.128215373	chr13	MIMAT0000872
rno-miR-19a-3p	1.196437637	chr15	MIMAT0000789
rno-miR-19a-5p	0.95035801	chr15	MIMAT0017098
rno-miR-19b-1-5p	0.95035801	chr15	MIMAT0017096
rno-miR-19b-2-5p	0.95035801	chrX	MIMAT0017097
rno-miR-19b-3p	0.968516411	chr15	MIMAT0000788
rno-miR-200a-3p	0.95035801	chr5	MIMAT0000874
rno-miR-200a-5p	0.95035801	chr5	MIMAT0017151
rno-miR-200b-3p	0.95035801	chr5	MIMAT0000875
rno-miR-200b-5p	0.95035801	chr5	MIMAT0017152
rno-miR-200c-3p	0.95035801	chr4	MIMAT0000873
rno-miR-200c-5p	0.95035801	chr4	MIMAT0017150
rno-miR-201-3p	0.95035801	chrX	MIMAT0017364
rno-miR-201-5p	0.95035801	chrX	MIMAT0012846
rno-miR-202-3p	1.149983292	chr1	MIMAT0017355
rno-miR-202-5p	0.95035801	chr1	MIMAT0012822
rno-miR-203a-3p	0.95035801	chr6	MIMAT0000876
rno-miR-203a-5p	0.95035801	chr6	MIMAT0017153
rno-miR-203b-3p	0.95035801	chr6	MIMAT0017800
rno-miR-203b-5p	0.95035801	chr6	MIMAT0017799
rno-miR-204-3p	0.95035801	chr1	MIMAT0004739
rno-miR-204-5p	1.142737787	chr1	MIMAT0000877
rno-miR-205	0.95035801	chr13	MIMAT0000878
rno-miR-206-3p	0.805209445	chr9	MIMAT0000879
rno-miR-206-5p	0.95035801	chr9	MIMAT0017154
rno-miR-207	0.95035801	chr5	MIMAT0003115
rno-miR-208a-3p	0.95035801	chr15	MIMAT0000880
rno-miR-208a-5p	121.8435355	chr15	MIMAT0017155
rno-miR-208b-3p	0.95035801	chr15	MIMAT0017845
rno-miR-208b-5p	0.95035801	chr15	MIMAT0017844
rno-miR-20a-3p	0.95035801	chr15	MIMAT0000603
rno-miR-20a-5p	0.963644752	chr15	MIMAT0000602
rno-miR-20b-3p	0.95035801	chrX	MIMAT0003212
rno-miR-20b-5p	1.102225034	chrX	MIMAT0003211
rno-miR-21-3p	0.95035801	chr10	MIMAT0004711
rno-miR-21-5p	0.960165497	chr10	MIMAT0000790
rno-miR-210-3p	1.045395098	chr1	MIMAT0000881
rno-miR-210-5p	0.95035801	chr1	MIMAT0017156
rno-miR-211-3p	1.038058249	chr1	MIMAT0017157

rno-miR-211-5p	0.95035801	chr1	MIMAT0000882
rno-miR-212-3p	1.306363783	chr10	MIMAT0000883
rno-miR-212-5p	0.95035801	chr10	MIMAT0017158
rno-miR-214-3p	0.967631674	chr13	MIMAT0000885
rno-miR-214-5p	0.95035801	chr13	MIMAT0017159
rno-miR-215	0.95035801	chr13	MIMAT0003118
rno-miR-216a-3p	0.95035801	chr14	MIMAT0017160
rno-miR-216a-5p	0.95035801	chr14	MIMAT0000886
rno-miR-216b-3p	0.95035801	chr14	MIMAT0017847
rno-miR-216b-5p	0.95035801	chr14	MIMAT0017846
rno-miR-217-3p	0.95035801	chr14	MIMAT0017161
rno-miR-217-5p	0.95035801	chr14	MIMAT0000887
rno-miR-218a-1-3p	0.95035801	chr14	MIMAT0017162
rno-miR-218a-2-3p	0.95035801	chr10	MIMAT0004740
rno-miR-218a-5p	1.329541122	chr10	MIMAT0000888
rno-miR-218b	0.95035801	chr14	MIMAT0017838
rno-miR-219-1-3p	0.95035801	chr20	MIMAT0004741
rno-miR-219-2-3p	0.95035801	chr3	MIMAT0005446
rno-miR-219-5p	0.95035801	chr3	MIMAT0000889
rno-miR-22-3p	0.91420968	chr10	MIMAT0000791
rno-miR-22-5p	1.149592855	chr10	MIMAT0003152
rno-miR-221-3p	0.875129606	chrX	MIMAT0000890
rno-miR-221-5p	0.95035801	chrX	MIMAT0017163
rno-miR-222-3p	0.95035801	chrX	MIMAT0000891
rno-miR-222-5p	0.95035801	chrX	MIMAT0017164
rno-miR-223-3p	0.836337641	chrX	MIMAT0000892
rno-miR-223-5p	0.95035801	chrX	MIMAT0017165
rno-miR-224-3p	0.95035801	chrX	MIMAT0017200
rno-miR-224-5p	0.95035801	chrX	MIMAT0003119
rno-miR-23a-3p	0.967043036	chr19	MIMAT0000792
rno-miR-23a-5p	0.95035801	chr19	MIMAT0004712
rno-miR-23b-3p	0.831277173	chr17	MIMAT0000793
rno-miR-23b-5p	0.95035801	chr17	MIMAT0017099
rno-miR-24-1-5p	0.95035801	chr17	MIMAT0003153
rno-miR-24-2-5p	1.345090646	chr19	MIMAT0005441
rno-miR-24-3p	0.871585741	chr17	MIMAT0000794
rno-miR-25-3p	0.963635401	chr12	MIMAT0000795
rno-miR-25-5p	0.95035801	chr12	MIMAT0004713
rno-miR-26a-3p	0.95035801	chr8	MIMAT0017100
rno-miR-26a-5p	1	chr8	MIMAT0000796
rno-miR-26b-3p	0.95035801	chr9	MIMAT0004714
rno-miR-26b-5p	1.433670015	chr9	MIMAT0000797
rno-miR-27a-3p	1.066995432	chr19	MIMAT0000799
rno-miR-27a-5p	0.95035801	chr19	MIMAT0004715

rno-miR-27b-3p	1.125387622	chr17	MIMAT0000798
rno-miR-27b-5p	0.95035801	chr17	MIMAT0017101
rno-miR-28-3p	0.95035801	chr11	MIMAT0004716
rno-miR-28-5p	1.041031916	chr11	MIMAT0000800
rno-miR-290	0.981902513	chr1	MIMAT0000893
rno-miR-291a-3p	0.95035801	chr1	MIMAT0000895
rno-miR-291a-5p	0.95035801	chr1	MIMAT0000894
rno-miR-291b	0.95035801	chr1	MIMAT0017822
rno-miR-292-3p	0.95035801	chr1	MIMAT0000897
rno-miR-292-5p	0.95035801	chr1	MIMAT0000896
rno-miR-293-3p	0.95035801	chr1	MIMAT0017365
rno-miR-293-5p	0.95035801	chr1	MIMAT0012847
rno-miR-294	0.95035801	chr1	MIMAT0012848
rno-miR-295-3p	0.95035801	chr1	MIMAT0017366
rno-miR-295-5p	0.95035801	chr1	MIMAT0012849
rno-miR-296-3p	0.95035801	chr3	MIMAT0004742
rno-miR-296-5p	1.689129923	chr3	MIMAT0000898
rno-miR-2964	0.95035801	chr3	MIMAT0017882
rno-miR-297	0.95035801	chr10	MIMAT0000899
rno-miR-298-3p	0.95035801	chr3	MIMAT0017166
rno-miR-298-5p	0.95035801	chr3	MIMAT0000900
rno-miR-2985	1.474206679	chr2	MIMAT0017891
rno-miR-299a-3p	0.95035801	chr6	MIMAT0017167
rno-miR-299a-5p	0.0037604	chr6	MIMAT0000901
rno-miR-299b-3p	0.95035801	chr6	MIMAT0017834
rno-miR-299b-5p	0.95035801	chr6	MIMAT0017833
rno-miR-29a-3p	1.162694439	chr4	MIMAT0000802
rno-miR-29a-5p	1.226902666	chr4	MIMAT0004718
rno-miR-29b-1-5p	0.95035801	chr4	MIMAT0005445
rno-miR-29b-2-5p	0.95035801	chr13	MIMAT0004717
rno-miR-29b-3p	1.755775027	chr4	MIMAT0000801
rno-miR-29c-3p	1.611248483	chr13	MIMAT0000803
rno-miR-29c-5p	1.796914419	chr13	MIMAT0003154
rno-miR-300-3p	0.00478863	chr6	MIMAT0000902
rno-miR-300-5p	0.95035801	chr6	MIMAT0004743
rno-miR-301a-3p	1.240018891	chr10	MIMAT0000552
rno-miR-301a-5p	0.95035801	chr10	MIMAT0017026
rno-miR-301b-3p	0.95035801	chr11	MIMAT0005304
rno-miR-301b-5p	0.95035801	chr11	MIMAT0017300
rno-miR-3065-3p	0.95035801	chr10	MIMAT0017840
rno-miR-3065-5p	0.95035801	chr10	MIMAT0017839
rno-miR-3068-3p	1.403149178	chr6	MIMAT0024846
rno-miR-3068-5p	0.95035801	chr6	MIMAT0024845
rno-miR-3072	0.95035801	chr6	MIMAT0025071

rno-miR-3074	0.95035801	chr17	MIMAT0017815
rno-miR-3075	0.95035801	chr16	MIMAT0025057
rno-miR-3085	110.9029169	chr1	MIMAT0017805
rno-miR-3099	0.95035801	chr1	MIMAT0025048
rno-miR-30a-3p	1.181024587	chr9	MIMAT0000809
rno-miR-30a-5p	1.139689637	chr9	MIMAT0000808
rno-miR-30b-3p	1.106712124	chr7	MIMAT0004721
rno-miR-30b-5p	1.257792554	chr7	MIMAT0000806
rno-miR-30c-1-3p	43.16537749	chr5	MIMAT0004719
rno-miR-30c-2-3p	1.240704631	chr9	MIMAT0005442
rno-miR-30c-5p	1	chr5	MIMAT0000804
rno-miR-30d-3p	88.7915573	chr7	MIMAT0004722
rno-miR-30d-5p	1.046962668	chr7	MIMAT0000807
rno-miR-30e-3p	1.217250421	chr5	MIMAT0004720
rno-miR-30e-5p	1	chr5	MIMAT0000805
rno-miR-3102	0.95035801	chr1	MIMAT0025051
rno-miR-3120	0.95035801	chr13	MIMAT0017900
rno-miR-31a-3p	0.95035801	chr5	MIMAT0017102
rno-miR-31a-5p	0.95035801	chr5	MIMAT0000810
rno-miR-31b	0.95035801	chr5	MIMAT0017816
rno-miR-32-3p	0.95035801	chr5	MIMAT0017103
rno-miR-32-5p	0.95035801	chr5	MIMAT0000811
rno-miR-320-3p	0.938153305	chr15	MIMAT0000903
rno-miR-320-5p	0.95035801	chr15	MIMAT0017168
rno-miR-322-3p	0.007249088	chrX	MIMAT0000547
rno-miR-322-5p	0.707125896	chrX	MIMAT0001619
rno-miR-323-3p	0.95035801	chr6	MIMAT0000550
rno-miR-323-5p	0.95035801	chr6	MIMAT0004637
rno-miR-324-3p	1.197608528	chr10	MIMAT0000554
rno-miR-324-5p	1.395843696	chr10	MIMAT0000553
rno-miR-325-3p	0.95035801	chrX	MIMAT0004639
rno-miR-325-5p	0.95035801	chrX	MIMAT0000557
rno-miR-326-3p	0.95035801	chr1	MIMAT0000560
rno-miR-326-5p	0.95035801	chr1	MIMAT0017028
rno-miR-327	0.95035801		MIMAT0000561
rno-miR-328a-3p	1.068722798	chr19	MIMAT0000564
rno-miR-328a-5p	0.954054905	chr19	MIMAT0017029
rno-miR-328b-3p	0.95035801	chr14	MIMAT0017904
rno-miR-329-3p	0.00242543	chr6	MIMAT0000566
rno-miR-329-5p	0.95035801	chr6	MIMAT0017031
rno-miR-33-3p	0.95035801	chr7	MIMAT0017104
rno-miR-33-5p	85.12649353	chr7	MIMAT0000812
rno-miR-330-3p	0.95035801	chr1	MIMAT0000568
rno-miR-330-5p	0.95035801	chr1	MIMAT0004641

rno-miR-331-3p	1.516131616	chr7	MIMAT0000570
rno-miR-331-5p	0.95035801	chr7	MIMAT0017033
rno-miR-335	0.452623846		MIMAT0000575
rno-miR-336-3p	0.95035801	chr10	MIMAT0017034
rno-miR-336-5p	0.95035801	chr10	MIMAT0000576
rno-miR-337-3p	0.004892026	chr6	MIMAT0000577
rno-miR-337-5p	0.95035801	chr6	MIMAT0017035
rno-miR-338-3p	0.715164981	chr10	MIMAT0000581
rno-miR-338-5p	0.95035801	chr10	MIMAT0004646
rno-miR-339-3p	1.346526852	chr12	MIMAT0004648
rno-miR-339-5p	0.95035801	chr12	MIMAT0000583
rno-miR-340-3p	119.9216726		MIMAT0000585
rno-miR-340-5p	1.821678416		MIMAT0004650
rno-miR-341	0.95035801	chr6	MIMAT0000587
rno-miR-342-3p	0.558513361	chr6	MIMAT0000589
rno-miR-342-5p	0.95035801	chr6	MIMAT0004652
rno-miR-343	0.95035801	chr1	MIMAT0000591
rno-miR-344a	0.95035801	chr1	MIMAT0017842
rno-miR-344a-3p	0.95035801	chr1	MIMAT0000592
rno-miR-344a-5p	0.95035801	chr1	MIMAT0004654
rno-miR-344b-2-3p	0.95035801	chr1	MIMAT0017812
rno-miR-344b-5p	0.95035801	chr1	MIMAT0017811
rno-miR-344g	0.95035801	chr1	MIMAT0025052
rno-miR-344i	0.95035801	chr1	MIMAT0025049
rno-miR-345-3p	0.95035801	chr6	MIMAT0004655
rno-miR-345-5p	1.094206628	chr6	MIMAT0000594
rno-miR-346	0.95035801	chr16	MIMAT0000596
rno-miR-347	1.870594953		MIMAT0000598
rno-miR-3473	0.709429388	chr13	MIMAT0024853
rno-miR-349	0.95035801	chr7	MIMAT0000599
rno-miR-34a-3p	0.95035801	chr5	MIMAT0017106
rno-miR-34a-5p	0.931489557	chr5	MIMAT0000815
rno-miR-34b-3p	0.95035801	chr8	MIMAT0017105
rno-miR-34b-5p	0.95035801	chr8	MIMAT0000813
rno-miR-34c-3p	0.95035801	chr8	MIMAT0004723
rno-miR-34c-5p	0.95035801	chr8	MIMAT0000814
rno-miR-350	1.034029826	chr13	MIMAT0000604
rno-miR-351-3p	0.95035801	chrX	MIMAT0017041
rno-miR-351-5p	0.95035801	chrX	MIMAT0000608
rno-miR-352	1.166347349		MIMAT0000610
rno-miR-3541	0.95035801	chr2	MIMAT0017795
rno-miR-3542	0.95035801	chr17	MIMAT0017796
rno-miR-3543	0.95035801	chr6	MIMAT0017797
rno-miR-3544	0.95035801	chr6	MIMAT0017798

rno-miR-3546	1.159624726	chr15	MIMAT0017801
rno-miR-3547	0.95035801	chr10	MIMAT0017802
rno-miR-3548	0.95035801	chr5	MIMAT0017806
rno-miR-3549	0.95035801	chr10	MIMAT0017807
rno-miR-3550	0.95035801	chr19	MIMAT0017808
rno-miR-3551-3p	0.95035801	chrX	MIMAT0017810
rno-miR-3551-5p	0.95035801	chrX	MIMAT0017809
rno-miR-3552	0.95035801	chrX	MIMAT0017813
rno-miR-3553	0.95035801	chr4	MIMAT0017814
rno-miR-3556a	0.95035801	chr4	MIMAT0017821
rno-miR-3556b	0.95035801	chr13	MIMAT0017818
rno-miR-3557-3p	0.95035801	chr18	MIMAT0017820
rno-miR-3557-5p	0.95035801	chr18	MIMAT0017819
rno-miR-3558-3p	0.95035801	chr6	MIMAT0017826
rno-miR-3558-5p	0.95035801	chr6	MIMAT0017825
rno-miR-3559-3p	0.95035801	chrX	MIMAT0017828
rno-miR-3559-5p	0.95035801	chrX	MIMAT0017827
rno-miR-3560	0.95035801	chrX	MIMAT0017829
rno-miR-3561-3p	0.95035801	chr7	MIMAT0017831
rno-miR-3561-5p	0.95035801	chr7	MIMAT0017830
rno-miR-3562	178.8827299	chr10	MIMAT0017832
rno-miR-3564	0.95035801	chr19	MIMAT0017837
rno-miR-3566	0.95035801	chr17	MIMAT0017841
rno-miR-3568	0.95035801	chr10	MIMAT0017848
rno-miR-3569	0.95035801	chr1	MIMAT0017849
rno-miR-3570	0.95035801	chr13	MIMAT0017850
rno-miR-3571	0.95035801	chr18	MIMAT0017851
rno-miR-3572	0.95035801	chr1	MIMAT0017853
rno-miR-3573-3p	105.724476	chrX	MIMAT0017857
rno-miR-3573-5p	0.95035801	chrX	MIMAT0017856
rno-miR-3574	0.95035801	chr1	MIMAT0017860
rno-miR-3575	0.95035801	chr4	MIMAT0017861
rno-miR-3576	0.95035801	chr6	MIMAT0017862
rno-miR-3577	0.95035801	chr10	MIMAT0017863
rno-miR-3578	0.95035801	chr6	MIMAT0017866
rno-miR-3579	0.95035801	chr6	MIMAT0017867
rno-miR-3580-3p	0.95035801	chrX	MIMAT0017869
rno-miR-3580-5p	0.95035801	chrX	MIMAT0017868
rno-miR-3582	0.95035801	chr18	MIMAT0017872
rno-miR-3583-3p	0.95035801	chr5	MIMAT0017874
rno-miR-3583-5p	0.95035801	chr5	MIMAT0017873
rno-miR-3584-3p	0.95035801	chr1	MIMAT0017876
rno-miR-3584-5p	0.816155411	chr1	MIMAT0017875
rno-miR-3585-3p	0.95035801	chrX	MIMAT0017879

rno-miR-3585-5p	0.95035801	chrX	MIMAT0017878
rno-miR-3586-3p	0.95035801	chr7	MIMAT0017881
rno-miR-3586-5p	0.95035801	chr7	MIMAT0017880
rno-miR-3587	0.95035801	chr4	MIMAT0017883
rno-miR-3588	1.793190672	chr11	MIMAT0017887
rno-miR-3589	0.95035801	chrX	MIMAT0017888
rno-miR-3590-3p	0.95035801	chr3	MIMAT0017890
rno-miR-3590-5p	0.95035801	chr3	MIMAT0017889
rno-miR-3591	0.95035801	chr18	MIMAT0017893
rno-miR-3592	0.95035801	chr6	MIMAT0017895
rno-miR-3593-3p	1.167307779	chr6	MIMAT0017897
rno-miR-3593-5p	0.95035801	chr6	MIMAT0017896
rno-miR-3594-3p	0.95035801	chr10	MIMAT0017899
rno-miR-3594-5p	0.95035801	chr10	MIMAT0017898
rno-miR-3595	0.95035801	chr6	MIMAT0017903
rno-miR-3596a	0.95035801	chr8	MIMAT0017886
rno-miR-3596b	0.95035801	chr17	MIMAT0017871
rno-miR-3596c	0.95035801	chr1	MIMAT0017877
rno-miR-3596d	0.95035801	chr17	MIMAT0017823
rno-miR-361-3p	0.95035801	chrX	MIMAT0017199
rno-miR-361-5p	1.164171093	chrX	MIMAT0003117
rno-miR-362-3p	0.429352025	chrX	MIMAT0017357
rno-miR-362-5p	0.95035801	chrX	MIMAT0012828
rno-miR-363-3p	0.95035801	chrX	MIMAT0003210
rno-miR-363-5p	0.95035801	chrX	MIMAT0003209
rno-miR-365-3p	1.220758226	chr10	MIMAT0001549
rno-miR-365-5p	0.95035801	chr10	MIMAT0017184
rno-miR-369-3p	0.95035801	chr6	MIMAT0003207
rno-miR-369-5p	0.95035801	chr6	MIMAT0003206
rno-miR-370-3p	1.058599223	chr6	MIMAT0003122
rno-miR-370-5p	0.95035801	chr6	MIMAT0017202
rno-miR-374-3p	0.95035801	chrX	MIMAT0017223
rno-miR-374-5p	1.198401554	chrX	MIMAT0003208
rno-miR-375-3p	0.95035801	chr9	MIMAT0005307
rno-miR-375-5p	0.95035801	chr9	MIMAT0017301
rno-miR-376a-3p	0.95035801	chr6	MIMAT0003198
rno-miR-376a-5p	0.95035801	chr6	MIMAT0003197
rno-miR-376b-3p	0.95035801	chr6	MIMAT0003196
rno-miR-376b-5p	0.95035801	chr6	MIMAT0003195
rno-miR-376c-3p	0.95035801	chr6	MIMAT0003194
rno-miR-376c-5p	0.95035801	chr6	MIMAT0017219
rno-miR-377-3p	0.95035801	chr6	MIMAT0003123
rno-miR-377-5p	0.95035801	chr6	MIMAT0017203
rno-miR-378a-5p	1.042217222	chr18	MIMAT0003378

rno-miR-378b	1	chr5	MIMAT0024855
rno-miR-379-3p	0.95035801	chr6	MIMAT0004791
rno-miR-379-5p	0.95035801	chr6	MIMAT0003192
rno-miR-380-3p	0.95035801	chr6	MIMAT0017302
rno-miR-380-5p	0.95035801	chr6	MIMAT0005308
rno-miR-381-3p	0.006003316	chr6	MIMAT0003199
rno-miR-381-5p	0.95035801	chr6	MIMAT0017220
rno-miR-382-3p	0.95035801	chr6	MIMAT0003202
rno-miR-382-5p	0.007369321	chr6	MIMAT0003201
rno-miR-383-3p	0.95035801	chr16	MIMAT0017197
rno-miR-383-5p	0.95035801	chr16	MIMAT0003114
rno-miR-384-3p	0.95035801	chrX	MIMAT0005310
rno-miR-384-5p	0.95035801	chrX	MIMAT0005309
rno-miR-409a-3p	0.95035801	chr6	MIMAT0003205
rno-miR-409a-5p	0.95035801	chr6	MIMAT0003204
rno-miR-409b	0.95035801	chr6	MIMAT0017870
rno-miR-410-3p	0.95035801	chr6	MIMAT0005311
rno-miR-410-5p	0.95035801	chr6	MIMAT0017303
rno-miR-411-3p	0.95035801	chr6	MIMAT0017304
rno-miR-411-5p	0.95035801	chr6	MIMAT0005312
rno-miR-412-3p	0.95035801	chr6	MIMAT0003124
rno-miR-412-5p	0.95035801	chr6	MIMAT0017204
rno-miR-421-3p	0.95035801	chrX	MIMAT0017175
rno-miR-421-5p	0.95035801	chrX	MIMAT0001320
rno-miR-423-3p	0.95035801	chr10	MIMAT0005313
rno-miR-423-5p	1	chr10	MIMAT0017305
rno-miR-425-3p	0.95035801	chr8	MIMAT0017306
rno-miR-425-5p	1	chr8	MIMAT0005314
rno-miR-429	0.95035801	chr5	MIMAT0001538
rno-miR-431	0.95035801	chr6	MIMAT0001626
rno-miR-433-3p	0.95035801	chr6	MIMAT0001628
rno-miR-433-5p	0.95035801	chr6	MIMAT0017192
rno-miR-434-3p	0.001573271	chr6	MIMAT0005315
rno-miR-434-5p	0.95035801	chr6	MIMAT0017307
rno-miR-448-3p	0.95035801	chrX	MIMAT0001534
rno-miR-448-5p	0.95035801	chrX	MIMAT0017177
rno-miR-449a-3p	0.95035801	chr2	MIMAT0017181
rno-miR-449a-5p	0.95035801	chr2	MIMAT0001543
rno-miR-449c-3p	0.95035801	chr2	MIMAT0017804
rno-miR-449c-5p	0.95035801	chr2	MIMAT0017803
rno-miR-450a-3p	0.95035801	chrX	MIMAT0017183
rno-miR-450a-5p	0.621707203	chrX	MIMAT0001547
rno-miR-451-3p	0.95035801	chr10	MIMAT0017193
rno-miR-451-5p	0.726341844	chr10	MIMAT0001633

rno-miR-455-3p	0.95035801	chr5	MIMAT0017308
rno-miR-455-5p	0.95035801	chr5	MIMAT0005316
rno-miR-463-3p	0.95035801	chrX	MIMAT0005317
rno-miR-463-5p	0.95035801	chrX	MIMAT0017309
rno-miR-465-3p	0.95035801	chrX	MIMAT0017367
rno-miR-465-5p	0.95035801	chrX	MIMAT0012850
rno-miR-466b-1-3p	1.018277658	chr17	MIMAT0017285
rno-miR-466b-2-3p	0.95035801	chr17	MIMAT0017286
rno-miR-466b-5p	0.884892496	chr17	MIMAT0005278
rno-miR-466c-3p	0.95035801	chr17	MIMAT0017287
rno-miR-466c-5p	0.95035801	chr17	MIMAT0005279
rno-miR-466d	0.95035801	chr17	MIMAT0017824
rno-miR-471-3p	0.95035801	chrX	MIMAT0017310
rno-miR-471-5p	0.95035801	chrX	MIMAT0005318
rno-miR-483-3p	1.85523636	chr1	MIMAT0003121
rno-miR-483-5p	1.026217477	chr1	MIMAT0017201
rno-miR-484	0.95035801	chr10	MIMAT0005319
rno-miR-485-3p	0.95035801	chr6	MIMAT0017222
rno-miR-485-5p	0.95035801	chr6	MIMAT0003203
rno-miR-487b-3p	0.932461652	chr6	MIMAT0003200
rno-miR-487b-5p	0.95035801	chr6	MIMAT0017221
rno-miR-488-3p	0.95035801	chr13	MIMAT0005341
rno-miR-488-5p	0.95035801	chr13	MIMAT0017320
rno-miR-489-3p	0.95035801	chr4	MIMAT0003113
rno-miR-489-5p	0.95035801	chr4	MIMAT0017196
rno-miR-490-3p	0.95035801	chr4	MIMAT0012823
rno-miR-490-5p	0.95035801	chr4	MIMAT0017356
rno-miR-493-3p	0.95035801	chr6	MIMAT0003191
rno-miR-493-5p	0.95035801	chr6	MIMAT0017217
rno-miR-494-3p	1.227892278	chr6	MIMAT0003193
rno-miR-494-5p	0.95035801	chr6	MIMAT0017218
rno-miR-495	0.95035801	chr6	MIMAT0005320
rno-miR-496-3p	0.95035801	chr6	MIMAT0012860
rno-miR-496-5p	0.95035801	chr6	MIMAT0017372
rno-miR-497-3p	0.95035801	chr10	MIMAT0017230
rno-miR-497-5p	0.743481629	chr10	MIMAT0003383
rno-miR-499-3p	0.95035801	chr3	MIMAT0017227
rno-miR-499-5p	0.20067308	chr3	MIMAT0003381
rno-miR-500-3p	0.642175754	chrX	MIMAT0005321
rno-miR-500-5p	0.95035801	chrX	MIMAT0017311
rno-miR-501-3p	0.95035801	chrX	MIMAT0017198
rno-miR-501-5p	0.95035801	chrX	MIMAT0003116
rno-miR-503-3p	0.95035801	chrX	MIMAT0017224
rno-miR-503-5p	0.008111718	chrX	MIMAT0003213

rno-miR-504	0.95035801	chrX	MIMAT0012830
rno-miR-505-3p	1.131993206	chrX	MIMAT0003380
rno-miR-505-5p	0.95035801	chrX	MIMAT0017226
rno-miR-509-3p	0.95035801	chrX	MIMAT0024850
rno-miR-509-5p	0.95035801	chrX	MIMAT0024849
rno-miR-511-3p	133.1238083	chr17	MIMAT0017358
rno-miR-511-5p	0.95035801	chr17	MIMAT0012829
rno-miR-532-3p	0.007138903	chrX	MIMAT0005323
rno-miR-532-5p	0.522582348	chrX	MIMAT0005322
rno-miR-539-3p	0.95035801	chr6	MIMAT0017212
rno-miR-539-5p	0.009666659	chr6	MIMAT0003176
rno-miR-540-3p	0.95035801	chr6	MIMAT0003174
rno-miR-540-5p	0.95035801	chr6	MIMAT0017211
rno-miR-541-3p	0.95035801	chr6	MIMAT0017213
rno-miR-541-5p	0.95035801	chr6	MIMAT0003177
rno-miR-542-3p	0.95035801	chrX	MIMAT0003179
rno-miR-542-5p	0.95035801	chrX	MIMAT0003178
rno-miR-543-3p	0.95035801	chr6	MIMAT0003175
rno-miR-543-5p	0.95035801	chr6	MIMAT0004787
rno-miR-544-3p	0.95035801	chr6	MIMAT0012831
rno-miR-544-5p	0.95035801	chr6	MIMAT0017359
rno-miR-547-3p	0.95035801	chrX	MIMAT0012851
rno-miR-547-5p	0.95035801	chrX	MIMAT0017368
rno-miR-551b-3p	0.95035801	chr2	MIMAT0005596
rno-miR-551b-5p	108.9579005	chr2	MIMAT0017335
rno-miR-568	0.95035801	chr11	MIMAT0012832
rno-miR-582-3p	0.95035801	chr2	MIMAT0017360
rno-miR-582-5p	0.95035801	chr2	MIMAT0012833
rno-miR-592	0.95035801	chr4	MIMAT0012834
rno-miR-598-3p	0.95035801	chr15	MIMAT0005325
rno-miR-598-5p	0.95035801	chr15	MIMAT0005324
rno-miR-615	0.95035801	chr7	MIMAT0012835
rno-miR-6215	1.229728643	chr5	MIMAT0024854
rno-miR-6216	1.052517995	chr3	MIMAT0024856
rno-miR-628	0.95035801	chr8	MIMAT0012836
rno-miR-6314	0.95035801	chr2	MIMAT0025047
rno-miR-6315	1.627792831	chr1	MIMAT0025050
rno-miR-6316	0.95035801	chr1	MIMAT0025053
rno-miR-6317	0.95035801	chr1	MIMAT0025054
rno-miR-6318	0.95035801	chr19	MIMAT0025055
rno-miR-6319	0.95035801	chr17	MIMAT0025056
rno-miR-632	0.95035801	chr10	MIMAT0012837
rno-miR-6320	0.95035801	chr15	MIMAT0025058
rno-miR-6321	0.95035801	chr14	MIMAT0025059

rno-miR-6322	0.95035801	chr12	MIMAT0025061
rno-miR-6323	0.95035801	chr11	MIMAT0025062
rno-miR-6324	0.95035801	chr11	MIMAT0025063
rno-miR-6325	0.95035801	chr11	MIMAT0025064
rno-miR-6326	0.95035801	chr10	MIMAT0025065
rno-miR-6327	0.95035801	chr10	MIMAT0025066
rno-miR-6328	136.1617147	chr20	MIMAT0025067
rno-miR-6329	0.95035801	chr8	MIMAT0025068
rno-miR-6330	0.95035801	chr8	MIMAT0025069
rno-miR-6331	0.95035801	chr6	MIMAT0025070
rno-miR-6332	0.95035801	chr5	MIMAT0025073
rno-miR-6333	0.95035801	chr5	MIMAT0025074
rno-miR-6334	0.95035801	chr3	MIMAT0025075
rno-miR-652-3p	0.896299626	chrX	MIMAT0005342
rno-miR-652-5p	1.313351878	chrX	MIMAT0017321
rno-miR-653-3p	0.95035801	chr4	MIMAT0017361
rno-miR-653-5p	0.95035801	chr4	MIMAT0012838
rno-miR-664-1-5p	0.95035801	chr18	MIMAT0017228
rno-miR-664-2-5p	0.95035801	chr13	MIMAT0017229
rno-miR-664-3p	1.417769009	chr13	MIMAT0003382
rno-miR-665	0.95035801	chr6	MIMAT0012844
rno-miR-666-3p	0.95035801	chr6	MIMAT0017371
rno-miR-666-5p	0.95035801	chr6	MIMAT0012855
rno-miR-667-3p	0.95035801	chr6	MIMAT0012852
rno-miR-667-5p	0.95035801	chr6	MIMAT0017369
rno-miR-668	0.95035801	chr6	MIMAT0012839
rno-miR-671	0.95035801	chr4	MIMAT0005326
rno-miR-672-3p	0.95035801	chrX	MIMAT0017312
rno-miR-672-5p	0.95035801	chrX	MIMAT0005327
rno-miR-673-3p	0.95035801	chr6	MIMAT0017313
rno-miR-673-5p	0.95035801	chr6	MIMAT0005328
rno-miR-674-3p	0.011164069	chr3	MIMAT0005330
rno-miR-674-5p	0.95035801	chr3	MIMAT0005329
rno-miR-675-3p	1.601520206	chr1	MIMAT0017363
rno-miR-675-5p	0.95035801	chr1	MIMAT0012841
rno-miR-678	0.95035801	chr20	MIMAT0012857
rno-miR-679	0.95035801	chr6	MIMAT0025072
rno-miR-702-3p	0.901690046	chr12	MIMAT0017885
rno-miR-702-5p	0.95035801	chr12	MIMAT0017884
rno-miR-708-3p	0.95035801	chr1	MIMAT0005332
rno-miR-708-5p	0.95035801	chr1	MIMAT0005331
rno-miR-711	0.95035801	chr8	MIMAT0012859
rno-miR-741-3p	0.95035801	chrX	MIMAT0017902
rno-miR-741-5p	0.95035801	chrX	MIMAT0017901

rno-miR-742-3p	0.95035801	chrX	MIMAT0005333
rno-miR-742-5p	0.95035801	chrX	MIMAT0017314
rno-miR-743a-3p	0.95035801	chrX	MIMAT0005334
rno-miR-743a-5p	0.95035801	chrX	MIMAT0017315
rno-miR-743b-3p	0.95035801	chrX	MIMAT0005280
rno-miR-743b-5p	0.95035801	chrX	MIMAT0017288
rno-miR-758-3p	0.95035801	chr6	MIMAT0005335
rno-miR-758-5p	0.95035801	chr6	MIMAT0017316
rno-miR-759	0.95035801	chr15	MIMAT0012856
rno-miR-760-3p	0.95035801	chr2	MIMAT0005337
rno-miR-760-5p	0.95035801	chr2	MIMAT0005336
rno-miR-761	0.95035801	chr5	MIMAT0012853
rno-miR-764-3p	0.95035801	chrX	MIMAT0017370
rno-miR-764-5p	0.95035801	chrX	MIMAT0012854
rno-miR-770-3p	1.147149405	chr6	MIMAT0017317
rno-miR-770-5p	0.95035801	chr6	MIMAT0005338
rno-miR-7a-1-3p	1.344089307	chr17	MIMAT0000607
rno-miR-7a-2-3p	0.95035801	chr1	MIMAT0017091
rno-miR-7a-5p	1.45818746	chr1	MIMAT0000606
rno-miR-7b	0.95035801		MIMAT0000780
rno-miR-802-3p	0.95035801	chr11	MIMAT0017362
rno-miR-802-5p	0.95035801	chr11	MIMAT0012840
rno-miR-871-3p	0.95035801	chrX	MIMAT0017289
rno-miR-871-5p	0.95035801	chrX	MIMAT0005281
rno-miR-872-3p	0.95035801	chr5	MIMAT0005283
rno-miR-872-5p	1.243201026	chr5	MIMAT0005282
rno-miR-873-3p	0.95035801	chr5	MIMAT0017318
rno-miR-873-5p	0.95035801	chr5	MIMAT0005339
rno-miR-874-3p	46.40523627	chr17	MIMAT0005284
rno-miR-874-5p	0.95035801	chr17	MIMAT0017290
rno-miR-875	0.95035801	chr7	MIMAT0012842
rno-miR-876	0.95035801	chr5	MIMAT0012843
rno-miR-877	0.95035801	chr20	MIMAT0005285
rno-miR-878	0.95035801	chrX	MIMAT0005286
rno-miR-879-3p	0.95035801	chr4	MIMAT0017291
rno-miR-879-5p	0.95035801	chr4	MIMAT0005287
rno-miR-880-3p	0.95035801	chrX	MIMAT0005288
rno-miR-880-5p	0.95035801	chrX	MIMAT0017292
rno-miR-881-3p	0.95035801	chrX	MIMAT0005289
rno-miR-881-5p	0.95035801	chrX	MIMAT0017293
rno-miR-883-3p	0.95035801	chrX	MIMAT0005290
rno-miR-883-5p	0.95035801	chrX	MIMAT0017294
rno-miR-92a-1-5p	0.95035801	chr15	MIMAT0017107
rno-miR-92a-2-5p	0.95035801	chrX	MIMAT0017108

rno-miR-92a-3p	1.018463234	chr15	MIMAT0000816
rno-miR-92b-3p	0.95035801	chr2	MIMAT0005340
rno-miR-92b-5p	0.95035801	chr2	MIMAT0017319
rno-miR-93-3p	0.95035801	chr12	MIMAT0017109
rno-miR-93-5p	1.188773615	chr12	MIMAT0000817
rno-miR-935	0.95035801	chr1	MIMAT0012845
rno-miR-96-3p	0.95035801	chr4	MIMAT0017110
rno-miR-96-5p	0.95035801	chr4	MIMAT0000818
rno-miR-98-3p	0.95035801	chrX	MIMAT0017111
rno-miR-98-5p	1.106923101	chrX	MIMAT0000819
rno-miR-99a-3p	0.954227719	chr11	MIMAT0004724
rno-miR-99a-5p	1.243159147	chr11	MIMAT0000820
rno-miR-99b-3p	82.13931438	chr1	MIMAT0004725
rno-miR-99b-5p	0.978458522	chr1	MIMAT0000821
rno-miR-9a-3p	0.95035801	chr1	MIMAT0004708
rno-miR-9a-5p	0.95035801	chr1	MIMAT0000781
rno-miR-9b-3p	0.95035801	chr1	MIMAT0017836
rno-miR-9b-5p	0.95035801	chr1	MIMAT0017835

Supplementary mRNA microarray information

Control_vs_Normal 2.0 fold up regulated genes

Fold change	Regulation	ACCESSION	GeneSymbol	GeneID
2.5321603	up	NM_001007759	3-Mar	364878
6.000517	up	XM_001080178	5-Mar	294079
2.239044	up	XM_001065952	6-Mar	294862
7.8270965	up	NM_057148	2-Sep	117515
7.8498096	up	NM_001011893	4-Sep	287606
9.984365	up	NM_022616	7-Sep	64551
2.8324516	up	XM_001063271	11-Sep	305227
21.22125	up	NM_133297	15-Sep	113922
192.48135	up	XM_001076609	A2bp1	302920
29.53327	up	XM_001057423	Aamp	301512
13.188923	up	XM_001077503	Aars	292023
2.5877452	up	NM_001034109	Aarsd1	619440
2.4969501	up	XM_344230	Aasdh	364136
7.48782	up	XM_001070860	Aasdhppt	300328
6.499917	up	NM_053720	Aatf	114512
3.5585437	up	NM_178095	Abca1	313210
2.7695181	up	NM_173307	Abca5	286970
3.35671	up	NM_207598	Abca7	299609
7.3138556	up	XM_001081603	Abca8a	303638
4.8900294	up	NM_001012166	Abcb10	361439
64.058136	up	NM_080582	Abcb6	140669
4.106377	up	NM_212518	Abcb7	302395
21.744947	up	NM_022281	Abcc1	24565
5.565488	up	NM_133411	Abcc4	170924
5.018865	up	NM_053924	Abcc5	116721
7.886269	up	NM_013039	Abcc8	25559
50.914726	up	NM_013040	Abcc9	25560
31.650076	up	XM_001064797	Abce1	361390
57.57595	up	XM_001056151	Abcf1	85493
18.05017	up	XM_001057136	Abcf2	311959
8.042051	up	NM_001011896	Abcf3	287982
6.8743954	up	NM_001024314	Abhd12	499913
5.6362	up	XM_001076411	Abhd13	306630
2.3718662	up	XM_001064693	Abhd2	293050
6.2116227	up	XM_001053662	Abhd4	364380
4.301085	up	NM_212524	Abhd5	316122
4.2911906	up	NM_001007680	Abhd6	305795
10.791459	up	NM_024397	Abi1	79249
2.7476451	up	NM_001044394	Ablim1	307989
2.6042612	up	NM_001001514	Ablim2	360958

110.376495	up	NM_175844	Abra	286965
9.75331	up	NM_001005902	Abtb1	297432
69.201385	up	NM_130433	Acaa2	170465
7.9568768	up	NM_053922	Acacb	116719
7.1480827	up	XM_001072510	Acad11	315973
19.423185	up	XM_001059349	Acad9	294973
33.392086	up	NM_016986	Acadm	24158
26.181276	up	NM_013084	Acadsb	25618
24.371769	up	NM_012891	Acadvl	25363
59.369328	up	NM_017075	Acat1	25014
3.3336012	up	NM_001006995	Acat2	308100
11.490718	up	NM_182843	Acbd3	289312
2.764437	up	NM_001012013	Acbd4	303577
11.946795	up	NM_001011906	Acbd6	289125
6.0442543	up	NM_012544	Ace	24310
12.656479	up	XM_001054451	Acin1	305884
38.53995	up	NM_017321	Aco1	50655
174.0852	up	NM_024398	Aco2	79250
7.7169228	up	NM_130756	Acot8	170588
53.98139	up	NM_001013960	Acot9	302640
8.001724	up	NM_017340	Acox1	50681
5.8309317	up	NM_021262	Acp1	24161
6.40473	up	NM_016988	Acp2	24162
2.2140758	up	XM_001079424	Acsf3	498962
31.874144	up	NM_012820	Acsl1	25288
45.02632	up	NM_057107	Acsl3	114024
6.566231	up	NM_053623	Acsl4	113976
4.284527	up	NM_053607	Acsl5	94340
2.959624	up	NM_130739	Acsl6	117243
13.425908	up	XM_001064684	Acss2	311569
1018.92096	up	NM_019212	Acta1	29437
69.88831	up	NM_031144	Actb	81822
24.48424	up	NM_019183	Actc1	29275
2.5368388	up	XM_001058533	Actg1	287876
4.5197883	up	NM_031005	Actn1	81634
19.282362	up	XM_001061664	Actn2	291245
305.7847	up	NM_133424	Actn3	171009
3.6932015	up	NM_031675	Actn4	63836
5.68593	up	NM_001039028	Actr1b	316333
43.398056	up	NM_001009268	Actr2	289820
6.7385473	up	NM_031068	Actr3	81732
18.266203	up	XM_001063449	Actr8	361107
3.3587387	up	NM_024486	Acvr1	79558
2.066603	up	NM_199230	Acvr1b	29381

3.746111	up	NM_031571	Acvr2a	29263
2.8916993	up	NM_001005383	Acy1	300981
18.543358	up	XM_001060176	Acyp1	299203
3.6294734	up	NM_130399	Ada	24165
5.914033	up	XM_001054737	Adam10	29650
6.6142163	up	NM_020308	Adam15	57025
2.2862673	up	XM_001054356	Adam4l1	500688
11.385195	up	NM_001014772	Adam9	290834
6.618955	up	NM_024400	Adamts1	79252
2.3827314	up	XM_213548	Adamts2	287899
3.6736531	up	XM_001058144	Adamts20	315263
4.0169187	up	NM_198761	Adamts5	304135
2.3295884	up	XM_001055774	Adamts8	300475
6.1983924	up	XM_001076761	Adamts9	312566
3.417174	up	NM_020101	Adap2	56826
2.3325517	up	NM_031006	Adar	81635
34.58998	up	NM_031007	Adcy2	81636
4.902949	up	NM_019285	Adcy4	54223
5.834807	up	XM_001077332	Adcy9	302950
2.4965441	up	NM_031552	Add3	25230
18.895517	up	NM_001007144	Adfp	298199
8.3343525	up	NM_001025423	Adhfe1	362474
2.723362	up	NM_199097	Adi1	298934
186.22127	up	NM_207587	Adipor1	289036
17.4825	up	NM_012895	Adk	25368
3.8220046	up	NM_012715	Adm	25026
5.3928375	up	NM_022681	Adnp	64622
10.938377	up	NM_183325	Adprh	25371
9.6545515	up	NM_001013054	Adprhl1	290880
5.9929714	up	NM_012492	Adrb2	24176
52.611755	up	NM_031708	Adrm1	65138
54.485783	up	XM_001076341	Adsl	315150
13.369838	up	XM_001059694	Adss	289276
13.746138	up	XM_001064302	Aen	361594
39.50045	up	NM_019220	Aes	29466
2.017053	up	XM_001073824	Aff4	303132
6.886626	up	XM_001079534	Afg3l1	361436
6.638204	up	XM_001061139	Afg3l2	307350
2.869712	up	XM_001055784	Aftph	305544
6.442519	up	NM_001031641	Aga	290923
3.3997002	up	XM_001063833	Agbl1	308724
5.862535	up	XM_001060407	Aggf1	310005
2.4261076	up	XM_001070395	Agk	502749
4.0805693	up	XM_001073642	Agl	362029

2.2620852	up	XM_001074707	Agpat2	311821
6.310175	up	XM_001071257	Agpat3	294324
12.883207	up	NM_001047849	Agpat6	290843
3.963369	up	NM_053350	Agps	84114
51.005424	up	XM_001061502	Agtpbp1	290986
4.861004	up	NM_001007654	Agtrap	298646
13.612818	up	XM_001068488	Ahcyl1	362013
2.0764534	up	XM_001062287	Ahcyl2	312192
3.074812	up	XM_342936	Ahdc1	362617
39.246582	up	XM_001078032	Ahnak	191572
2.6095138	up	XM_001072924	Ahnak2	314478
4.8196297	up	NM_031356	Aifm1	83533
8.189546	up	XM_001056179	Aifm2	361843
23.44167	up	NM_053757	Aimp1	114632
3.1875315	up	NM_001037348	Aimp2	288480
15.352392	up	NM_172327	Aip	282827
101.58822	up	NM_024349	Ak1	24183
6.610669	up	NM_001033967	Ak2	24184
12.256261	up	NM_013218	Ak3	26956
3.0123482	up	XM_001077273	Akap10	360540
2.8027196	up	NM_001011974	Akap2	298024
2.6466582	up	NM_001013946	Akap8l	299569
4.929081	up	NM_001030054	Akirin1	595134
30.953552	up	NM_001039914	Akirin2	297968
2.7817883	up	XM_001064334	Akna	362530
146.08061	up	NM_031000	Akr1a1	78959
30.733274	up	NM_012498	Akr1b1	24192
13.058733	up	NM_001013084	Akr1b10	296972
9.06148	up	XM_001055853	Akr1b1-ps2	500301
3.2376504	up	NM_138547	Akr1c14	191574
3.3826423	up	XM_001062695	Akr1c19	307096
10.852871	up	XM_001062861	Akr1cl1	361267
2.1570952	up	NM_001008342	Akr1cl2	307091
7.7203465	up	NM_033230	Akt1	24185
12.08868	up	NM_017093	Akt2	25233
2.8263206	up	NM_001011926	Aktip	291906
33.632755	up	NM_024484	Alas1	65155
7.360723	up	NM_013197	Alas2	25748
101.89897	up	NM_022407	Aldh1a1	24188
2.2776518	up	NM_053896	Aldh1a2	116676
4.283002	up	NM_022547	Aldh1l1	64392
10.010283	up	NM_032416	Aldh2	29539
3.121298	up	NM_031731	Aldh3a2	65183
19.512348	up	XM_001062804	Aldh5a1	291133

15.35261	up	NM_031057	Aldh6a1	81708
4.4918294	up	XM_001059375	Aldh7a1	291450
17.821646	up	NM_012495	Aldoa	24189
3.3566067	up	NM_012497	Aldoc	24191
4.061029	up	NM_001014176	Alg14	362031
6.0540533	up	NM_001011897	Alg3	287983
2.1964996	up	NM_001033709	Alg6	362547
9.430672	up	XM_001062293	Alkbh	362766
74.7198	up	NM_001014180	Alkbh3	362169
11.1195545	up	XM_001077537	Alkbh5	303193
2.1913896	up	XM_001053732	Alkbh7	679944
2.9805956	up	XM_001074073	Alms1	297408
2.386447	up	NM_012822	Alox5	25290
4.1660113	up	NM_017260	Alox5ap	29624
3.6144125	up	XM_001076258	Alpk1	310879
50.81985	up	XM_574162	Alpk2	498875
3.2314541	up	XM_001064402	Als2cr4	316412
2.8599336	up	NM_012816	Amacr	25284
2.5805383	up	NM_031011	Amd1	81640
53.42303	up	XM_001062954	Amfr	361367
2.717046	up	XM_001056974	Amot	300289
2.8098428	up	XM_001068539	Amotl2	65157
292.3485	up	NM_138876	Ampd1	25028
2.8529332	up	XM_001069998	Ampd2	362015
8.427021	up	NM_031544	Ampd3	25095
14.014046	up	NM_001014121	Amz2	360650
2.1628516	up	XM_001070328	Anapc10	361389
9.958425	up	XM_001081824	Anapc11	498030
4.8163958	up	XM_001060533	Anapc13	685029
12.35652	up	XM_001072691	Anapc2	296558
4.8366694	up	XM_001054052	Anapc4	305420
7.8486447	up	NM_001080147	Anapc5	288671
7.182477	up	XM_001079480	Anapc7	304490
3.1952474	up	XM_001068063	Angel2	305035
2.037773	up	XM_001054429	Angptl1	679942
3.3982532	up	NM_133569	Angptl2	171100
3.0313187	up	NM_199115	Angptl4	362850
6.408802	up	XM_342337	Ank2	362036
18.748466	up	NM_053714	Ankh	114506
34.295944	up	XM_001054207	Ankhd1-Eif4ebp3	679725
8.989602	up	XM_001074108	Ankmy2	314046
166.4432	up	NM_013220	Ankrd1	27064
2.1619973	up	XM_341466	Ankrd10	361183
10.633544	up	XM_001077732	Ankrd11	365023

2.8428707	up	NM_001012148	Ankrd13a	360823
24.571209	up	XM_001072667	Ankrd17	289521
3.0118604	up	XM_001055096	Ankrd2	309374
52.090214	up	XM_001056748	Ankrd23	316330
2.650079	up	XM_001079798	Ankrd27	361555
3.1961844	up	XM_001057687	Ankrd28	306264
3.0009418	up	XM_001059377	Ankrd32	294601
2.337372	up	XM_001056809	Ankrd39	367251
4.963217	up	NM_001013948	Ankrd46	299982
2.3951921	up	XM_001069855	Ankrd50	294988
2.8543224	up	NM_001012112	Ankrd9	314457
2.7347398	up	NM_001009676	Anks3	302937
11.27179	up	NM_001014219	Ankzf1	363255
4.4816146	up	XM_001066475	Ano1	309135
2.4952466	up	XM_001078269	Ano10	301111
33.251278	up	NM_131911	Anp32b	170724
15.70632	up	NM_001013200	Anp32e	361999
4.296673	up	NM_031012	Anpep	81641
17.96255	up	NM_001011918	Anxa11	290527
8.07941	up	NM_019905	Anxa2	56611
8.374845	up	NM_012823	Anxa3	25291
23.184551	up	NM_024155	Anxa4	79124
13.370571	up	NM_013132	Anxa5	25673
13.363781	up	NM_024156	Anxa6	79125
10.17161	up	NM_130416	Anxa7	155423
4.5783677	up	NM_031582	Aoc3	29473
6.5652194	up	NM_019363	Aox1	54349
2.8132002	up	XM_001072391	Ap1g1	171494
2.690689	up	NM_001044239	Ap1m1	306332
2.5762644	up	XM_001078324	Ap2a1	308578
2.3573399	up	NM_080583	Ap2b1	140670
78.42753	up	NM_053837	Ap2m1	116563
6.4795465	up	NM_022952	Ap2s1	65046
15.220906	up	XM_001067082	Ap3b1	309969
34.332226	up	XM_001076208	Ap3d1	314633
11.709057	up	NM_133593	Ap3m1	171126
2.8181615	up	NM_133305	Ap3m2	140667
3.409417	up	XM_001054882	Ap3s1	302290
5.0201273	up	XM_001077874	Apbb2	305338
6.5232234	up	NM_012500	Apeh	24206
20.408228	up	NM_024148	Apex1	79116
22.044313	up	NM_001047090	Aph1b	300802
5.467188	up	XM_001071044	Api5	362170
2.8543355	up	XM_001072220	Apip	295961

44.722656	up	XM_001055798	Aplp2	64312
19.722841	up	XM_001068886	Apoa1bp	295229
3.7775202	up	NM_012907	Apobec1	25383
77.949905	up	XM_001063358	Apobec2	301226
5.46784	up	NM_012777	Apod	25239
41.539253	up	NM_138828	Apoe	25728
5.8380165	up	XM_001075769	Apol11a	300060
3.5779715	up	NM_001013175	Apol3	315108
7.8390474	up	NM_001025066	Apol9a	503164
6.9394336	up	NM_001014105	Apool	317191
90.471054	up	NM_019288	App	54226
4.089921	up	XM_001081113	Appbp2	303396
13.467267	up	NM_001013061	Aprt	292072
8.884778	up	NM_012778	Aqp1	25240
41.17633	up	NM_001033663	Araf	64363
2.073893	up	XM_001066901	Arap1	361617
7.1024213	up	NM_001007662	Arcn1	300674
4.0675106	up	XM_001057202	Ard1a	363518
9.787642	up	NM_022518	Arf1	64310
5.3626866	up	NM_024150	Arf2	79119
3.2695446	up	NM_080904	Arf3	140940
7.6358023	up	NM_024151	Arf4	79120
4.8539157	up	NM_024149	Arf5	79117
4.2535024	up	NM_024152	Arf6	79121
4.627296	up	NM_181083	Arfgef2	296380
2.4701583	up	NM_021763	Arfip1	60382
23.030876	up	XM_001065757	Arhgap10	688429
6.111298	up	XM_001055788	Arhgap12	307016
5.669783	up	NM_022244	Arhgap17	63994
3.4826872	up	XM_001054365	Arhgap28	301709
3.114694	up	NM_001047869	Arhgap5	299012
2.1628036	up	NM_001007005	Arhgdia	360678
3.338776	up	NM_001009600	Arhgdib	362456
3.7610605	up	XM_001055379	Arhgef3	290541
5.666207	up	NM_001005565	Arhgef6	363509
2.4321694	up	NM_023957	Arhgef9	66013
16.646517	up	XM_001065794	Arid1a	297867
11.4550085	up	XM_001059099	Arid2	366980
3.978019	up	XM_001077431	Arid4a	314205
6.2825966	up	NM_001034934	Arid5a	316327
8.32094	up	NM_001013108	Arih1	300756
3.6545162	up	NM_001012275	Arih2	316005
8.2211	up	NM_022385	Arl1	64187
2.6341107	up	NM_031711	Arl2	65142

22.431414	up	NM_001024906	Arl2bp	498910
3.6325939	up	NM_053979	Arl5a	117050
8.47544	up	NM_198737	Arl6ip1	293551
4.9378667	up	NM_001025630	Arl6ip4	288656
49.739845	up	NM_023972	Arl6ip5	66028
43.175583	up	NM_001024332	Arl8b	500282
5.6389093	up	XM_231295	Armc10	296758
2.330012	up	XM_236599	Armc8	315949
3.1737704	up	NM_001014274	Armcx2	367903
6.632151	up	NM_001014273	Armcx3	367902
5.575323	up	NM_012780	Arnt	25242
3.7476935	up	NM_024362	Arntl	29657
6.925033	up	NM_031146	Arpc1a	81824
10.484723	up	NM_019289	Arpc1b	54227
16.201515	up	XM_001056906	Arpc2	301511
8.361481	up	XM_001079492	Arpc3	288669
2.5892081	up	XM_001078712	Arpc4	297518
4.70514	up	NM_001025717	Arpc5	360854
12.093276	up	NM_001037767	Arpc5l	296710
10.802543	up	XM_001059654	Arpc5l2	501132
11.544813	up	NM_031660	Arpp19	60336
2.0429704	up	XM_343492	Arpp-21	363153
2.6597683	up	XM_001071550	Arrdc1	366001
4.7321415	up	XM_001070067	Arrdc2	306344
57.7006	up	NM_001007797	Arrdc3	309945
2.313434	up	NM_001047917	Arsk	365619
68.34608	up	XM_001068204	Art1	308873
19.04833	up	NM_001012034	Art3	305235
3.073155	up	NM_080890	As3mt	140925
2.7610886	up	NM_053407	Asah1	84431
6.2895966	up	XM_001057256	Asb10	499972
49.34223	up	NM_001037367	Asb12	503446
11.578418	up	XM_001055574	Asb14	680076
63.044647	up	NM_001011984	Asb2	299266
3.8920708	up	XM_344277	Asb3	364227
2.1861699	up	NM_001024318	Asb4	500017
33.167694	up	NM_001044247	Asb5	361187
2.5254962	up	XM_001059593	Asb7	365277
3.0951343	up	XM_001062104	Ascc3	309887
12.737001	up	XM_001059946	Asf1a	294408
8.219282	up	XM_001074246	Ash1l	310638
29.502392	up	XM_001062523	Ash2l	290829
5.6914687	up	NM_021577	Asl	59085
3.7834642	up	XM_001075108	Asmtl	288527

3.3005311	up	XM_001066031	Asna1	288919
20.087944	up	XM_001060870	Asnsd1	299507
2.0561006	up	NM_024399	Aspa	79251
32.699738	up	NM_001098239	Asph	312981
2.023331	up	NM_001034922	Atad3a	298682
2.3972971	up	XM_001077907	Ate1	293526
4.878411	up	XM_235657	Atf1	315305
2.4626472	up	NM_012912	Atf3	25389
14.821287	up	NM_024403	Atf4	79255
13.208881	up	NM_001002809	Atf6b	406169
12.267808	up	XM_001070613	Atf7ip	312800
6.7329793	up	XM_343619	Atg16l1	363278
4.0643525	up	XM_233220	Atg4c	313391
3.91956	up	XM_001071698	Atg4d	686505
3.908768	up	NM_001014250	Atg5	365601
29.507113	up	NM_031014	Atic	81643
22.791864	up	XM_001063138	Atl2	298757
2.893929	up	XM_001078346	Atmin	315037
4.9451313	up	XM_001067830	Atp11b	361929
3.2146845	up	XM_001070411	Atp13a1	290673
3.850685	up	XM_001053119	Atp13a3	360728
76.67486	up	NM_012504	Atp1a1	24211
197.87514	up	NM_012505	Atp1a2	24212
10.173984	up	NM_013113	Atp1b1	25650
34.6453	up	NM_012507	Atp1b2	24214
2.2677236	up	NM_012913	Atp1b3	25390
111.51877	up	NM_058213	Atp2a1	116601
211.49785	up	NM_017290	Atp2a2	29693
34.174427	up	NM_053311	Atp2b1	29598
2.9598765	up	XM_001057885	Atp2b3	29599
215.4668	up	NM_023093	Atp5a1	65262
482.72385	up	NM_134364	Atp5b	171374
72.97612	up	NM_139106	Atp5d	245965
2.5501146	up	NM_139099	Atp5e	245958
140.97514	up	NM_134365	Atp5f1	171375
5.0476246	up	NM_017311	Atp5g1	29754
85.58982	up	NM_133556	Atp5g2	171082
72.6738	up	NM_053756	Atp5g3	114630
89.058784	up	NM_019383	Atp5h	641434
16.370697	up	XM_001057586	Atp5hl1	306478
77.29934	up	NM_080481	Atp5i	140608
12.60453	up	XM_001074466	Atp5j2	690441
40.449818	up	NM_212516	Atp5l	300677
21.982586	up	NM_138883	Atp5o	192241

44.36263	up	NM_031785	Atp6ap1	83615
5.427698	up	XM_001060853	Atp6ap2	302526
7.4682746	up	NM_031604	Atp6v0a1	29757
3.9915278	up	NM_053775	Atp6v0a2	116455
10.451377	up	XM_001068154	Atp6v0b	298451
53.27877	up	NM_130823	Atp6v0c	170667
38.955	up	NM_001011927	Atp6v0d1	291969
18.418705	up	NM_053578	Atp6v0e1	94170
3.244484	up	NM_001002253	Atp6v0e2	436582
2.1767688	up	XM_001058034	Atp6v1a	685232
5.5793324	up	NM_057213	Atp6v1b2	117596
18.678198	up	NM_001011992	Atp6v1c1	299971
67.069305	up	NM_199386	Atp6v1d	299159
61.60033	up	NM_053884	Atp6v1f	116664
2.0459306	up	NM_052803	Atp7a	24941
2.899825	up	XM_001064261	Atp8b1	291555
31.681538	up	XM_001074928	Atp8b2	361984
11.986331	up	XM_001057484	Atp9b	291411
2.5085518	up	NM_001014181	Atpbd4	362191
25.560417	up	NM_012915	Atpif1	25392
2.089606	up	XM_001055797	Atrip	301014
5.6856046	up	NM_031351	Atrn	83526
8.093949	up	NM_133313	Atxn10	170821
3.6116064	up	XM_341928	Atxn2l	361649
2.1690488	up	XM_001069337	Atxn7	361015
4.1122947	up	XM_001070248	Atxn7l2	310781
36.02868	up	XM_001053439	Auh	361215
379.9817	up	NM_001079899	Aup1	680423
21.43631	up	NM_001004237	Aurkaip1	298687
15.819605	up	XM_001080523	Aven	311299
3.3221467	up	NM_001025705	Azi2	316051
3.390028	up	NM_022585	Azin1	58961
23.337152	up	NM_012512	B2m	24223
2.3316505	up	XM_001072516	B3gnt1	293667
2.1898081	up	NM_001009539	B4galt3	494342
4.0964828	up	XM_001076733	B9d1	287383
3.6807668	up	NM_019204	Bace1	29392
2.3378658	up	NM_001002802	Bace2	288227
2.0727062	up	NM_022698	Bad	64639
74.20521	up	XM_001059235	Bag1	297994
4.875405	up	XM_001073000	Bag2	690038
14.277131	up	NM_001011936	Bag3	293524
6.6095986	up	NM_001008526	Bag5	366734
12.644788	up	NM_053631	Banf1	114087

3.9258	up	XM_001077496	Banp	292064
13.581373	up	NM_133300	Bat1	114612
19.32357	up	NM_001033968	Bat3	94342
20.515944	up	NM_001034157	Bat4	415064
7.847773	up	NM_017059	Bax	24887
2.0720322	up	XM_001077467	Baz1b	368002
3.0787008	up	XM_001058884	Baz2b	317627
2.0853593	up	NM_053618	Bbs2	113948
2.0439956	up	XM_001074489	Bbs9	315484
2.295225	up	NM_001079938	Bbx	303970
3.9494696	up	NM_031752	Bcam	78958
13.338813	up	NM_001004224	Bcap31	293852
42.189884	up	XM_001064236	Bcas2	295334
8.660483	up	XM_001081081	Bcas3	363662
23.746956	up	NM_022400	Bcat2	64203
12.5732155	up	XM_001055516	Bccip	361666
5.3586416	up	NM_012782	Bckdha	25244
40.14666	up	XM_001062965	Bckdhb	29711
20.302467	up	NM_019244	Bckdk	29603
3.3504298	up	NM_031328	Bcl10	83477
2.9141133	up	XM_001078625	Bcl2l12	361567
2.5636845	up	XM_001058979	Bcl2l13	312682
21.809916	up	XM_001078626	Bcl7c	293514
2.184531	up	XM_001059711	Bcorl1	302810
2.5550654	up	XM_001079915	Bcr	309696
5.08194	up	NM_001007666	Bcs1l	301514
2.4965022	up	XM_001075590	Bdh2	295458
20.016169	up	NM_001034117	Becn1	114558
2.8711295	up	XM_001071606	Bend7	361275
7.161517	up	XM_001080943	Best3	314847
4.6359253	up	NM_019251	Bet1	29631
7.3895063	up	NM_019368	Bet1l	54400
8.190391	up	NM_001013125	Bfar	304709
2.3314772	up	NM_207611	Bhlhb9	317407
12.073148	up	NM_053328	Bhlhe40	79431
2.4546218	up	NM_198765	Bicd2	306809
2.868189	up	NM_022684	Bid	64625
37.038155	up	NM_053959	Bin1	117028
28.09526	up	NM_001013186	Bin3	361065
28.161678	up	NM_133582	Blcap	171113
32.8155	up	NM_001024233	Bles03	266609
4.131518	up	NM_001034163	Blmh	287552
25.46678	up	XM_001068887	Bloc1s1	288785
10.6959095	up	NM_001037349	Bloc1s2	293938

15.708718	up	XM_001072142	Blvrb	292737
6.945328	up	NM_001017494	Blzf1	498266
3.1219976	up	XM_225606	Bmi1	307151
3.4946735	up	XM_573559	Bmp2k	498333
2.4708722	up	NM_012827	Bmp4	25296
4.13103	up	NM_013107	Bmp6	25644
4.726178	up	NM_030849	Bmpr1a	81507
9.506041	up	XM_001065181	Bmpr2	140590
2.843524	up	XM_001055775	Bnip2	300811
48.826645	up	NM_053420	Bnip3	84480
15.102681	up	NM_080888	Bnip3l	140923
8.383247	up	NM_001013854	Bod1	287173
11.279407	up	NM_001071776	Bola1	365875
5.346138	up	XM_001072676	Bola3	297388
4.7194037	up	NM_001024250	Bop1	300050
59.241627	up	NM_199382	Bpgm	296973
2.290188	up	NM_001037206	Bphl	361239
12.490537	up	NM_171990	Bpnt1	64473
2.901968	up	XM_001070228	Braf	114486
3.8309627	up	XM_001059392	Brcc3	316794
8.981144	up	XM_001053059	Brd1	315210
7.5126	up	NM_212495	Brd2	294276
2.0125089	up	XM_342396	Brd3	362092
10.359122	up	XM_001066875	Brd7	361374
3.2546442	up	XM_217740	Brd9	308067
3.298359	up	XM_001073175	Brf1	299347
48.92462	up	NM_001009604	Bri3	304284
3.2271814	up	NM_001017487	Bri3bp	498176
16.694239	up	NM_001009605	Brms1	293668
4.338918	up	XM_001075894	Brms1l	299053
4.791757	up	NM_001007707	Brp16	315094
4.2402864	up	XM_001053799	Brwd1	304061
2.18416	up	XM_001080483	Brwd2	309016
8.962216	up	XM_001061389	Bsdc1	297890
227.49521	up	NM_012783	Bsg	25246
14.338101	up	NM_001011932	Btbd1	293060
2.348517	up	NM_001014022	Btbd10	308890
5.826672	up	NM_001077683	Btbd6	690367
4.605985	up	NM_001013073	Btbd9	294318
64.534454	up	NM_017259	Btg2	29619
4.2549996	up	NM_001007148	Btrc	361765
2.2369063	up	NM_001047906	Bub3	361662
4.50847	up	NM_001025277	Bud13	300687
3.293831	up	NM_053556	Bud31	89819

6.5330644	up	NM_001077590	Bves	365603
5.242926	up	XM_001063295	Bxdc1	294436
9.5616865	up	NM_001029915	Bxdc2	294799
8.991022	up	XM_001059406	Bxdc5	499725
38.89032	up	NM_182674	Bysl	359727
15.120922	up	NM_198789	Bzw1	363232
3.0442598	up	NM_134402	Bzw2	171439
2.4271712	up	NM_001030033	C1galt1c1	302499
2.9095225	up	NM_001008515	C1qa	298566
10.535032	up	NM_019262	C1qb	29687
6.894697	up	NM_019259	C1qbp	29681
21.3199	up	NM_001008524	C1qc	362634
2.9778128	up	NM_001012123	C1qtnf5	315598
14.711772	up	XM_344414	C1qtnf9	364395
6.837448	up	XM_001061611	C1r	312705
3.2733378	up	NM_138900	C1s	192262
5.6766067	up	NM_001002805	C4-2	406161
8.1329	up	NM_031504	C4b	24233
35.90061	up	XM_217464	Cab39	301574
5.6589746	up	NM_001013185	Cabc1	360887
3.8937705	up	NM_053575	Cabin1	94165
3.8566473	up	XM_001059501	Cables2	311703
14.008239	up	NM_012919	Cacna2d1	25399
17.129946	up	NM_017346	Cacnb1	50688
129.12779	up	NM_019255	Cacng1	29658
13.3153305	up	NM_001004208	Cacybp	289144
8.8872595	up	XM_001063923	Cad	24240
94.12793	up	NM_139190	Calcoco1	246047
2.845629	up	NM_012717	Calcrl	25029
17.123108	up	NM_031969	Calm1	24242
10.015566	up	NM_017326	Calm2	50663
9.149396	up	NM_012518	Calm3	24244
3.188855	up	XM_001073968	Calm-ps2	686404
27.69333	up	NM_022399	Calr	64202
4.0287137	up	NM_022535	Calu	64366
3.898624	up	NM_134468	Camk1	171503
8.780509	up	NM_012920	Camk2a	25400
3.2684913	up	NM_001042356	Camk2b	24245
9.044523	up	NM_012519	Camk2d	24246
38.7659	up	NM_133605	Camk2g	171140
4.2433834	up	NM_173337	Camk2n1	287005
4.6154246	up	NM_053334	Camlg	81715
4.2969527	up	XM_001063719	Camsap1l1	289400
3.1917675	up	XM_001079889	Camta2	287462

7.4719954	up	NM_054004	Cand1	117152
31.595694	up	NM_172008	Canx	29144
112.325455	up	NM_053874	Cap2	116653
25.192085	up	NM_017116	Capn2	29154
5.556382	up	NM_001030037	Capn7	306260
46.25997	up	NM_017118	Capns1	29156
32.168545	up	NM_001012185	Caprin1	362173
12.038891	up	NM_001009180	Capza2	493810
136.7749	up	NM_001005903	Capzb	298584
6.0510445	up	NM_175708	Car11	308588
5.0597916	up	NM_019291	Car2	54231
82.034676	up	NM_019292	Car3	54232
9.558495	up	NM_019174	Car4	29242
3.2095249	up	NM_152790	Carhsp1	260416
58.661495	up	NM_001030041	Carm1	363026
8.621288	up	XM_001065753	Cars	293638
5.534884	up	NM_147144	Casc3	259170
3.5238395	up	NM_053736	Casp4	114555
4.04799	up	NM_022260	Casp7	64026
3.2238884	up	NM_017131	Casq2	29209
46.566135	up	NM_012520	Cat	24248
37.975243	up	NM_133651	Cav1	25404
3.3277876	up	NM_131914	Cav2	363425
3.069957	up	NM_019155	Cav3	29161
15.657298	up	NM_199412	Cbara1	365567
9.195261	up	NM_001013191	Cbfb	361391
3.4782376	up	NM_133601	Cblb	171136
4.2905254	up	XM_001073155	Cbll1	314028
2.055758	up	XM_001081346	Cbx1	360609
3.6466987	up	XM_001069676	Cbx5	300266
2.3255088	up	NM_001034078	Cbx8	303731
2.738537	up	NM_001013869	Cc2d1a	288908
5.442461	up	XM_001067280	Cc2d1b	313478
5.711236	up	XM_001058761	Ccar1	361849
5.0985	up	NM_001013164	Ccbl1	311844
11.072871	up	XM_001077352	Ccdc101	293488
10.818293	up	NM_001024866	Ccdc104	289859
4.2445984	up	XM_001063219	Ccdc107	313496
17.063381	up	XM_001054087	Ccdc109a	294560
5.0569277	up	XM_001079737	Ccdc123	292811
35.382374	up	XM_001066312	Ccdc124	290642
2.7143605	up	XM_001056062	Ccdc126	500117
7.5393353	up	NM_198766	Ccdc127	308060
4.6059914	up	NM_001037644	Ccdc130	304656

7.5736666	up	XM_001064458	Ccdc132	312083
8.520621	up	XM_001064000	Ccdc136	362331
2.5300078	up	XM_001060019	Ccdc22	317381
9.2889595	up	XM_001066869	Ccdc25	361059
4.3326726	up	NM_001037789	Ccdc28a	361454
3.9168158	up	XM_001061538	Ccdc28b	682445
3.7199562	up	NM_001014266	Ccdc41	366872
2.608636	up	NM_001013862	Ccdc45	287766
14.259357	up	NM_001013974	Ccdc47	303606
4.4916778	up	XM_001081381	Ccdc49	360613
4.6375527	up	XM_001079910	Ccdc53	299707
13.018948	up	XM_001081477	Ccdc56	498000
5.8555965	up	XM_001080741	Ccdc59	314799
5.1294622	up	XM_343001	Ccdc75	362685
2.4813442	up	XM_001073185	Ccdc76	499697
6.3158946	up	NM_001006974	Ccdc86	293738
11.2897005	up	NM_001024885	Ccdc90b	308820
10.376654	up	NM_001014061	Ccdc91	312863
2.0486848	up	NM_001083898	Ccdc92	1E+08
2.6359658	up	NM_001024997	Ccdc93	304743
9.195194	up	XM_001070408	Ccdc97	292724
2.6038368	up	NM_031530	Ccl2	24770
3.325593	up	NM_001008513	Ccl21b	298006
4.147306	up	NM_001013045	Ccl24	288593
2.1743116	up	NM_001004202	Ccl6	287910
5.901844	up	XM_001058860	Ccnc	114839
8.160855	up	NM_001013204	Ccndbp1	362201
115.8949	up	NM_012923	Ccng1	25405
20.464033	up	XM_001063045	Ccng2	29157
2.5878708	up	NM_052981	Ccnh	84389
4.230724	up	NM_053662	Ccnl1	114121
6.51954	up	NM_001013094	Ccnl2	298686
5.85205	up	XM_222617	Ccnt2	304758
12.946275	up	XM_001065252	Ccny	361261
22.53303	up	XM_001053208	Ccpg1	363098
4.988872	up	NM_001025752	Ccrk	364666
2.1144006	up	XM_001076293	Ccrl2	316019
8.102286	up	NM_053425	Ccs	84485
42.7172	up	NM_001005905	Cct2	299809
102.686325	up	NM_199091	Cct3	295230
44.91495	up	NM_182814	Cct4	29374
79.74785	up	NM_001004078	Cct5	294864
85.09993	up	XM_001073942	Cct7	297406
89.67262	up	XM_001067000	Cct8	288305

2.0531044	up	NM_021744	Cd14	60350
67.91685	up	NM_022523	Cd151	64315
2.7371173	up	XM_001061914	Cd163	312701
22.058336	up	NM_031812	Cd164	83689
5.0199065	up	NM_017079	Cd1d1	25109
3.31513	up	NM_031518	Cd200	24560
125.06201	up	NM_012752	Cd24	25145
3.7762468	up	XM_001069761	Cd248	293669
2.9095812	up	NM_181475	Cd2ap	316258
95.56574	up	NM_031561	Cd36	29184
2.9368775	up	NM_017124	Cd37	29185
5.9559345	up	NM_019195	Cd47	29364
6.838255	up	NM_139103	Cd48	245962
4.8423815	up	NM_022269	Cd55	64036
44.7832	up	NM_012925	Cd59	25407
472.81027	up	NM_017125	Cd63	29186
19.92722	up	NM_001031638	Cd68	287435
17.219	up	NM_013069	Cd74	25599
14.805586	up	NM_053018	Cd9	24936
48.8424	up	NM_001012164	Cd97	361383
2.6943119	up	NM_134459	Cd99l2	171485
18.30737	up	NM_001012156	Cdadc1	361052
2.0588112	up	XM_001080911	Cdan1	311348
17.041716	up	NM_053877	Cdc123	116656
4.9082522	up	XM_001073065	Cdc14a	310806
12.665949	up	NM_001024744	Cdc16	290875
8.731294	up	NM_133571	Cdc25a	171102
8.188706	up	NM_001013240	Cdc26	366381
2.7394168	up	XM_001053539	Cdc2l5	306998
8.366453	up	NM_001011941	Cdc37l1	293886
4.618226	up	XM_342154	Cdc40	361859
170.5083	up	NM_171994	Cdc42	64465
2.7964385	up	NM_001009689	Cdc42ep2	309175
5.5197883	up	NM_001039044	Cdc42se1	499672
19.675259	up	NM_053527	Cdc5l	85434
6.241849	up	NM_001024769	Cdc73	304832
3.0179	up	NM_001037214	Cdca4	500727
36.624775	up	NM_138889	Cdh13	192248
10.030221	up	XM_001057656	Cdh5	307618
3.2402925	up	NM_138899	Cdipt	192260
2.0954094	up	NM_001025722	Cdk10	361434
7.618134	up	XM_341076	Cdk2ap1	360804
15.342617	up	XM_001069623	Cdk2ap2	688405
25.010014	up	NM_053593	Cdk4	94201

3.7034216	up	NM_080885	Cdk5	140908
8.465618	up	NM_145721	Cdk5rap1	252827
3.349721	up	XM_001058949	Cdk5rap2	286919
7.933959	up	NM_024488	Cdk5rap3	80278
5.3679233	up	XM_001071127	Cdk7	171150
2.0545156	up	NM_001007743	Cdk9	362110
9.339441	up	XM_001061875	Cdkal1	361243
27.369062	up	NM_080782	Cdkn1a	114851
6.5586305	up	NM_001014000	Cdkn2aip	306455
2.9257338	up	NM_001037543	Cdnf	361276
2.6659768	up	NM_052809	Cdo1	81718
65.247086	up	NM_001014097	Cdv3	315970
2.7234313	up	NM_001014145	Cdyl	361237
2.7298281	up	XM_001078221	Cdyl2	292044
3.6250856	up	NM_024125	Cebpb	24253
8.359068	up	NM_013154	Cebpd	25695
10.328476	up	NM_012831	Cebpg	25301
6.718985	up	XM_001059054	Cebpz	362686
2.0003226	up	XM_001058617	Cecr5	312680
5.3363137	up	NM_001008366	Cenpn	361416
4.9000325	up	XM_577104	Cenpv	501702
5.729021	up	XM_001066167	Centd3	361314
3.1762292	up	XM_001073869	Cep57	315423
2.9721305	up	NM_001037772	Cep63	300963
6.271549	up	XM_001067027	Cep76	291540
6.9029512	up	NM_001007699	Cept1	310773
11.884252	up	XM_001061358	Cetn3	170895
2.0391939	up	NM_001077642	Cfd	54249
5.065846	up	NM_199378	Cfdp1	292027
9.887448	up	NM_130409	Cfh	155012
19.404205	up	NM_017147	Cfl1	29271
77.22207	up	XM_001079054	Cfl2	366624
9.208987	up	NM_001033864	Cflar	117279
9.481672	up	NM_053899	Cgrrf1	116679
4.2927327	up	XM_001080834	Chac1	362196
2.2001092	up	XM_001054484	Chac2	360994
3.2025077	up	XM_001061702	Chaf1a	363333
41.37581	up	NM_001007008	Chchd10	361824
34.029705	up	NM_001015019	Chchd2	316643
60.704067	up	XM_001066573	Chchd3	296966
6.494862	up	XM_001078868	Chchd5	296147
2.0765626	up	XM_001072660	Chchd6	297436
2.286408	up	XM_238731	Chd1	308215
2.8777342	up	XM_001060704	Chd1l	310707

62.97613	up	XM_001063352	Chd4	117535
5.9007263	up	XM_001071315	Chd7	312974
2.8865972	up	NM_053560	Chi3l1	89824
9.508309	up	XM_001076614	Chic2	83835
6.9421225	up	NM_001047854	Chid1	293628
10.771304	up	NM_017177	Chkb	29367
4.335953	up	NM_017067	Chm	24942
36.20636	up	XM_001055138	Chmp2a	365191
8.131731	up	XM_001063932	Chmp2b	363720
5.59728	up	XM_001054829	Chmp4bl1	679886
4.1614585	up	NM_001025410	Chmp5	297995
2.9045954	up	XM_001069293	Chmp7	364419
8.479533	up	XM_001075005	Chordc1	315447
14.045393	up	NM_024139	Chp	64152
5.78803	up	XM_001059575	Chrd	117275
155.67854	up	NM_024485	Chrna1	79557
25.905964	up	NM_019298	Chrnd	54240
6.236061	up	NM_017194	Chrne	29422
37.022354	up	NM_019145	Chrng	25753
3.331971	up	NM_001037775	Chst12	304322
2.5169733	up	XM_001061709	Chuk	309361
20.129736	up	NM_001008766	Ciao1	29231
18.692673	up	NM_001007689	Ciapin1	307649
8.416794	up	XM_214551	Cidea	291541
32.26614	up	XM_001076441	Cilp	315761
2.2892995	up	XM_001071553	Cinp	299334
6.2875385	up	NM_001007799	Cir1	362149
3.7153103	up	NM_031147	Cirbp	81825
8.896402	up	NM_001009640	Cirh1a	291987
11.169317	up	XM_001073750	Cisd1	294362
6.27585	up	XM_001077768	Cisd2	295457
35.562317	up	NM_053698	Cited2	114490
17.597733	up	XM_001079943	Ciz1	296639
3.328036	up	XM_001079545	Ckap4	362859
5.8631377	up	XM_001068589	Ckap5	311191
5.085857	up	NM_012529	Ckb	24264
86.64652	up	NM_012530	Ckm	24265
7.6111474	up	NM_053722	Clasp2	114514
23.541286	up	NM_053363	Clcn3	84360
40.106064	up	NM_022198	Clcn4-2	60586
20.998333	up	NM_031701	Cldn5	65131
10.706383	up	NM_022393	Clec10a	64195
7.9611726	up	NM_001002807	Clic1	406864
2.8191454	up	NM_001009651	Clic2	294141

2.1330311	up	NM_001002022	Clint1	360515
10.850651	up	NM_031745	Clip1	65201
9.501891	up	NM_001013942	Clip4	298801
34.823406	up	XM_001063325	Clk1	301434
5.9368863	up	NM_001014254	Clk2	365842
6.0169015	up	NM_134340	Clk3	171305
14.923058	up	NM_001013041	Clk4	287269
4.5647006	up	XM_001074158	Cln6	315746
3.0436008	up	NM_001007686	Cln8	306619
14.541622	up	NM_031719	Clns1a	65160
2.6820843	up	NM_021856	Clock	60447
4.792034	up	NM_001009599	Clp1	311166
14.040102	up	NM_022947	Clpb	65041
12.003879	up	XM_001055676	Clpp	301117
30.92172	up	XM_001067335	Clptm1	292696
13.097498	up	XM_001056887	Clptm1l	316916
36.940216	up	NM_001007803	Clpx	300786
8.53191	up	NM_019299	Cltc	54241
30.687202	up	NM_053021	Clu	24854
4.0020075	up	NM_001008770	Cmbl	310201
7.3592467	up	XM_001077986	Cmc1	363162
3.6667593	up	NM_001025655	Cmpk1	298410
123.5596	up	NM_022598	Cnbp	64530
28.436686	up	NM_001010920	Cndp2	291394
5.0951223	up	XM_001062581	Cnih4	289324
10.250109	up	NM_001039011	Cnksr1	298545
5.6593657	up	NM_019359	Cnn3	54321
4.505202	up	XM_001063389	Cno	364183
16.239101	up	XM_001056829	Cnot1	291841
5.2429404	up	NM_001007003	Cnot10	316034
18.387878	up	NM_001011988	Cnot2	299805
24.80612	up	NM_001037782	Cnot4	312227
4.4464226	up	NM_001013856	Cnot6	287249
3.2392614	up	XM_001058225	Cnot7	306492
2.3881269	up	NM_012809	Cnp	25275
30.241064	up	NM_001077585	Cnpy2	685814
2.668859	up	NM_001014232	Cnrip1	364208
3.9426458	up	XM_001060344	Cnst	498297
2.874506	up	NM_013166	Cntf	25707
3.1899745	up	NM_001003929	Cntfr	313173
5.4623456	up	XM_001072175	Cobra1	311796
15.737337	up	XM_001075681	Cog4	361407
7.9708567	up	NM_001004262	Cog6	310411
35.38938	up	XM_001074828	Cog8	291990

4.1996794	up	NM_017360	Coil	50998
8.16884	up	XM_001081230	Col1a1	29393
30.168715	up	NM_053356	Col1a2	84352
43.611385	up	NM_032085	Col3a1	84032
31.798918	up	XM_001067473	Col4a1	290905
14.418434	up	XM_001076134	Col4a2	306628
2.4155078	up	XM_001055156	Col4a5	363457
6.872646	up	NM_134452	Col5a1	85490
5.1550736	up	XM_001061982	Col5a2	85250
10.6175785	up	XM_001079642	Col6a2	361821
8.407541	up	XM_001068252	Col6a3	367313
2.339867	up	NM_019274	Colq	29755
5.537376	up	NM_001004276	Commd10	361323
9.3822365	up	NM_198732	Commd3	291339
11.754261	up	XM_001074149	Commd4	363068
4.610896	up	NM_001033692	Commd9	295956
2.3476143	up	XM_001065422	Comtd1	305685
12.744287	up	NM_080781	Copb1	114023
8.117743	up	XM_001068587	Cope	290659
14.070874	up	NM_153297	Cops2	261736
82.79271	up	NM_001004200	Cops3	287367
4.381929	up	NM_001004275	Cops4	360915
34.692245	up	NM_001025695	Cops5	312916
22.59242	up	XM_001066309	Cops6	304343
25.231167	up	NM_001047098	Cops7a	312710
2.4076877	up	XM_001064503	Cops7b	363273
22.416548	up	NM_001013227	Cops8	363283
3.7452233	up	XM_001069027	Copz1	315345
92.955536	up	XM_001081315	Copz2	360611
43.49864	up	NM_001044255	Coq2	498332
11.201948	up	NM_019187	Coq3	29309
8.673856	up	NM_001011983	Coq6	299195
4.0290613	up	NM_001035257	Coq9	498909
3.1686523	up	NM_130411	Coro1a	155151
38.576466	up	NM_139115	Coro6	245982
2.1458266	up	XM_001077234	Coro7	192276
2.154971	up	XM_001078708	Cotl1	361422
2.0994322	up	XM_001074020	Cox11	690300
20.340193	up	NM_001033699	Cox15	309391
2.1515925	up	NM_053540	Cox17	89786
8.546109	up	XM_001072704	Cox18	289522
7.87649	up	XM_001074417	Cox19	304330
57.5805	up	NM_017202	Cox4i1	29445
5.6267486	up	NM_001012165	Cox4nb	361425

127.948944	up	NM_145783	Cox5a	252934
29.524828	up	NM_053586	Cox5b	94194
21.163568	up	NM_012814	Cox6a1	25282
156.01904	up	NM_012812	Cox6a2	25278
9.203441	up	XM_001071713	Cox6b1	502592
13.856096	up	NM_019360	Cox6c	54322
2.4087033	up	NM_173303	Cox6c1	286962
28.105246	up	NM_022503	Cox7a2	29507
7.298852	up	XM_001056904	Cox7a2l	298762
69.00015	up	NM_182819	Cox7b	303393
68.0105	up	NM_012786	Cox8b	25250
4.4564185	up	NM_012836	Cpd	25306
4.1615205	up	NM_013128	Cpe	25669
6.017639	up	XM_001080170	Cpeb3	309510
9.323474	up	XM_001064017	Cpeb4	303010
2.4870894	up	XM_001062196	Cpne2	291861
2.3370073	up	XM_001057562	Cpne3	313087
6.5500736	up	XM_001075234	Cpsf1	366952
30.993057	up	NM_001030030	Cpsf3	298916
3.7641869	up	NM_001033892	Cpsf3l	298688
5.4039216	up	XM_001080973	Cpsf6	299811
3.8855965	up	NM_001014245	Cpsf7	365407
144.49237	up	NM_013200	Cpt1b	25756
6.273811	up	NM_012930	Cpt2	25413
3.3904786	up	XM_001054321	Cpxm2	293566
7.9852753	up	NM_001005265	Cr1l	54243
2.4247987	up	XM_001080418	Cradd	314756
312.9088	up	NM_001004085	Crat	311849
9.814938	up	NM_001015003	Crbn	297498
9.173784	up	NM_053670	Crcp	114205
18.763306	up	NM_001013092	Creb3	298400
2.7000895	up	XM_001062098	Crebzf	293112
8.132166	up	XM_001075616	Creg1	289185
7.018107	up	NM_001024783	Creld1	312638
4.491788	up	NM_001037208	Creld2	362978
2.3454204	up	NM_013086	Crem	25620
103.682884	up	XM_001064272	Crim1	298744
11.845988	up	NM_019907	Cript	56725
2.1511254	up	NM_138916	Crkrs	192350
3.8606632	up	XM_001080961	Crlf3	54395
14.5643015	up	NM_001014258	Crls1	366196
8.105855	up	NM_053797	Crnkl1	116481
2.438559	up	NM_001033895	Crtc2	310615
3.406118	up	NM_133405	Cry2	170917

2.4046028	up	NM_175757	Cryl1	290277
8.129669	up	NM_001013044	Cryzl1	288256
80.866516	up	NM_130755	Cs	170587
196.21747	up	NM_031979	Csda	83807
29.719015	up	NM_054006	Csde1	117180
2.0229056	up	NM_001029901	Csf1r	307403
5.270346	up	XM_001056935	Csgalnact2	297554
59.019688	up	NM_053615	Csnk1a1	113927
8.160327	up	NM_031617	Csnk1e	58822
20.552734	up	NM_001033870	Csnk1g2	65278
4.088149	up	NM_022855	Csnk1g3	64823
30.899601	up	NM_001035238	Csnk2b	81650
2.5035596	up	XM_001060285	Cspp1	362472
2.716615	up	NM_017148	Csrp1	29276
5.5134377	up	XM_001054440	Csrp2bp	362224
50.973103	up	NM_057144	Csrp3	117505
41.337467	up	NM_012837	Cst3	25307
9.314902	up	XM_001056457	Cst7	296257
11.545439	up	NM_012838	Cstb	25308
3.4082983	up	XM_001068092	Cstf2	683927
2.6558013	up	NM_001077672	Cstf3	362178
29.631828	up	NM_019201	Ctbp1	29382
8.761196	up	NM_053335	Ctbp2	81717
2.1001241	up	NM_031023	Ctbs	81652
26.765892	up	XM_001058378	Ctdp1	291414
3.6049063	up	XM_001077330	Ctdspl	301056
7.553417	up	NM_022266	Ctgf	64032
20.643759	up	NM_001007145	Ctnna1	307505
7.4160266	up	XM_001059679	Ctnnal1	298019
7.170866	up	XM_001075213	Ctnnbip1	503000
4.199721	up	NM_001024870	Ctnnbl1	296320
12.125343	up	XM_001053648	Ctps	313560
6.546154	up	XM_001077817	Ctr9	293184
15.861689	up	NM_001011959	Ctsa	296370
10.734612	up	NM_017097	Ctsc	25423
11.175459	up	NM_134334	Ctsd	171293
2.7396216	up	NM_001034110	Ctsf	361704
37.353195	up	NM_012939	Ctsh	25425
97.64516	up	NM_013156	Ctsl1	25697
6.9041677	up	NM_017320	Ctss	50654
22.898617	up	NM_183330	Ctsz	252929
5.339244	up	NM_021868	Cttn	60465
20.179745	up	NM_001013971	Cuedc1	303419
6.73863	up	NM_001079886	Cuedc2	294009

15.595758	up	NM_001025421	Cugbp1	362160
6.1538367	up	NM_001083586	Cugbp2	29428
33.508846	up	XM_001069456	Cul1	362356
32.119812	up	XM_001057445	Cul2	361258
3.520133	up	XM_001063669	Cul3	301555
3.2302954	up	NM_022683	Cul5	64624
3.6710908	up	XM_001063728	Cul9	316228
22.46775	up	NM_212494	Cuta	294288
14.599508	up	XM_001060579	Cutc	361760
3.044315	up	XM_001070410	Cux1	116639
30.72	up	NM_001024987	Cwc15	300361
3.1177204	up	NM_139089	Cxcl10	245920
3.7660136	up	NM_001033882	Cxcl12	24772
11.143226	up	NM_001013137	Cxcl14	306748
3.0393105	up	NM_001017478	Cxcl16	497942
4.2695026	up	NM_145672	Cxcl9	246759
3.636117	up	NM_022205	Cxcr4	60628
6.4653864	up	NM_022245	Cyb5a	64001
6.8454914	up	NM_030586	Cyb5b	80773
90.19974	up	NM_001013126	Cyb5r1	304805
6.0508485	up	NM_001014244	Cyb5r2	365345
11.854248	up	NM_138877	Cyb5r3	25035
4.440033	up	NM_133427	Cyb5r4	171015
2.0615273	up	NM_024160	Cyba	79129
3.56996	up	XM_001059652	Cyc1	300047
2.3401525	up	NM_012839	Cycs	25309
8.6782465	up	XM_001054108	Cyfip1	308666
2.6967208	up	XM_001058247	Cyld	312937
5.864506	up	NM_199401	Cyp20a1	316435
2.087533	up	NM_178847	Cyp27a1	301517
2.213298	up	NM_023025	Cyp2j4	65210
2.233753	up	NM_016999	Cyp4b1	24307
2.815618	up	XM_576190	Cyp4f17	500801
34.176067	up	NM_031327	Cyr61	83476
2.467084	up	NM_053911	Cyth2	116692
4.7426805	up	NM_001039455	Cytsa	361828
2.3084812	up	XM_001080564	Daam1	314212
3.1420856	up	NM_024159	Dab2	79128
8.773282	up	NM_138910	Dad1	192275
3.234583	up	XM_001072224	Daglb	304289
49.467266	up	NM_001014214	Dalrd3	363146
6.3446903	up	NM_022526	Dap	64322
10.076181	up	NM_001011950	Dap3	295238
5.2128105	up	XM_001060919	Dapk1	306722

6.059432	up	NM_022546	Dapk3	64391
11.424552	up	NM_053799	Dars	116483
3.575912	up	NM_001034143	Dars2	304919
4.990488	up	NM_080891	Daxx	140926
14.666377	up	NM_001013107	Dazap2	300235
21.898495	up	NM_031853	Dbi	25045
2.9919918	up	NM_031352	Dbnl	83527
50.491344	up	NM_012543	Dbp	24309
4.9818625	up	XM_001058421	Dbr1	681234
3.688839	up	XM_001073131	Dbt	29611
5.288455	up	NM_001009686	Dcaf11	305895
3.2056837	up	XM_001056278	Dcaf4	362762
24.963007	up	XM_001071288	Dcaf6	289181
3.5056915	up	NM_001007724	Dcakd	360639
53.25532	up	NM_017306	Dci	29740
6.2349544	up	NM_053343	Dclk1	83825
7.8380213	up	XM_001063735	Dclre1a	292127
62.008347	up	NM_024129	Dcn	29139
2.3066683	up	XM_001057512	Dcp1b	500305
18.038834	up	NM_153302	Dcps	266605
6.7752714	up	NM_024130	Dctn1	29167
23.186987	up	NM_001004239	Dctn2	299850
6.244371	up	NM_053404	Dctn4	84428
11.834647	up	NM_001037778	Dctn5	308961
31.27587	up	XM_001059405	Dctn6	290798
2.3516357	up	XM_001067883	Dcun1d1	310324
2.7509327	up	XM_001068808	Dcun1d2	688913
3.334473	up	NM_001024886	Dcun1d3	309035
5.669425	up	NM_001009696	Dcun1d5	315405
2.2486556	up	NM_199403	Dd25	360863
3.9493	up	NM_212532	Ddah2	294239
2.339397	up	NM_001034149	Ddi2	313668
2.241766	up	NM_080399	Ddit4l	140582
39.397552	up	NM_001012104	Ddost	313648
2.2529137	up	NM_013137	Ddr1	25678
7.8547926	up	XM_001065242	Ddr2	685781
77.549225	up	XM_001081180	Ddrgk1	296162
114.85998	up	NM_024131	Ddt	29318
103.68748	up	NM_053414	Ddx1	84474
4.4987054	up	XM_235480	Ddx17	315133
2.8210907	up	NM_001005381	Ddx19a	292022
5.410443	up	NM_001037201	Ddx21	317399
18.14988	up	XM_001060655	Ddx23	300208
18.953556	up	NM_199119	Ddx24	373065

13.561609	up	XM_001075340	Ddx27	362274
5.6037664	up	XM_001076081	Ddx28	364995
4.0938525	up	XM_001079220	Ddx31	311835
12.887377	up	NM_053563	Ddx39	89827
30.683641	up	XM_001060162	Ddx3x	317335
3.8955057	up	XM_001064233	Ddx41	314336
52.086357	up	NM_139098	Ddx46	245957
65.73583	up	NM_001007613	Ddx5	287765
2.0113418	up	XM_001080674	Ddx51	304570
4.420295	up	NM_053525	Ddx52	85432
3.6265357	up	XM_001079855	Ddx54	360815
2.3583364	up	XM_001067411	Ddx58	297989
2.133376	up	XM_001071010	Deadc1	361453
12.379829	up	NM_057197	Decr1	117543
11.923687	up	NM_001004255	Dek	306817
3.9090805	up	XM_001066914	Dennd1b	289051
19.696602	up	XM_001073504	Dennd5a	308942
4.1029706	up	XM_001065993	Depdc5	305464
7.4471254	up	XM_001079920	Derl2	691956
49.367943	up	NM_022531	Des	64362
7.542408	up	NM_001037194	Det1	308775
6.026564	up	XM_001078444	Dexi	497857
2.367773	up	NM_001012472	Dgcr14	360741
2.3481236	up	XM_001065223	Dgcr6	303794
3.9107437	up	XM_001064513	Dgcr8	287954
8.20417	up	NM_080787	Dgka	140866
6.887165	up	XM_001068249	Dguok	297389
2.0037742	up	NM_001011978	Dhdds	298541
20.059237	up	NM_001004207	Dhps	288923
4.4999456	up	NM_001014119	Dhrs11	360583
11.938601	up	NM_153315	Dhrs4	266686
6.5056386	up	NM_001008507	Dhrs7b	287380
40.08466	up	XM_001078936	Dhrs7c	287411
4.1742864	up	XM_213723	Dhrsx	288525
26.649769	up	XM_001054651	Dhx15	289693
15.042342	up	NM_212496	Dhx16	294232
3.9328325	up	NM_001013249	Dhx30	367172
2.9424906	up	XM_001078816	Dhx37	288647
3.2917242	up	NM_001005873	Dhx40	287595
3.2826054	up	XM_001062787	Dhx57	366532
3.2102568	up	XM_001081462	Dhx58	303538
3.9366288	up	NM_001047844	Dhx8	287727
5.2796493	up	NM_001008292	Diablo	288753
2.3318148	up	XM_001068155	Dicer1	299284

3.3359015	up	XM_001065350	Dimt1l	294718
2.7607126	up	XM_001073902	Dis3	306103
2.0587316	up	NM_001008380	Dis3l	363077
11.909129	up	NM_133419	Dkc1	170944
6.8344455	up	NM_031025	Dlat	81654
27.664204	up	NM_199385	Dld	298942
7.138936	up	NM_012788	Dlg1	25252
28.206076	up	NM_001006981	Dlst	299201
4.9418244	up	NM_001015006	Dmap1	298447
3.0840495	up	XM_577842	Dmbt1	170568
6.186767	up	NM_001005244	Dmd	24907
10.419917	up	XM_001066025	Dmpk	308405
9.872541	up	XM_001080139	Dmrt2	309430
3.2205164	up	NM_053693	Dmtf1	114485
3.7089806	up	XM_001054434	Dmxl1	307429
3.8192606	up	NM_022934	Dnaja1	65028
5.8948417	up	NM_032079	Dnaja2	84026
20.839537	up	NM_001038595	Dnaja3	360481
14.20258	up	XM_001069407	Dnajb1	361384
13.529241	up	NM_001015021	Dnajb11	360734
5.070878	up	NM_001013907	Dnajb12	294513
2.3453126	up	NM_001013076	Dnajb4	295549
13.626656	up	XM_001061825	Dnajb5	313811
3.607048	up	NM_001013209	Dnajb6	362293
11.124341	up	NM_012699	Dnajb9	24908
8.927833	up	XM_001063159	Dnajc10	295690
11.451715	up	XM_001076236	Dnajc11	362666
5.397524	up	XM_001072547	Dnajc13	363127
13.573345	up	XM_001072571	Dnajc15	290370
7.7356772	up	NM_001013887	Dnajc18	291677
4.237259	up	XM_342483	Dnajc24	362184
3.774616	up	XM_001077538	Dnajc30	368190
53.954098	up	NM_001013196	Dnajc4	361717
2.321244	up	NM_024161	Dnajc5	79130
19.34017	up	NM_213625	Dnajc7	303536
20.295422	up	XM_001064040	Dnajc9	364240
5.983137	up	NM_001009666	Dnal4	300078
103.16023	up	NM_001014223	Dnase1l1	363522
5.9180703	up	NM_053655	Dnm1l	114114
8.472759	up	NM_013199	Dnm2	25751
10.796343	up	NM_053354	Dnmt1	84350
6.3657565	up	NM_001024879	Dnpep	301529
4.142021	up	XM_001072013	Dnttip1	171437
2.5411725	up	XM_001073752	Dock6	367039

2.7810245	up	NM_001025006	Dohh	314644
3.280613	up	NM_212497	Dom3z	361799
5.1151915	up	XM_001055500	Dopey2	304077
13.115497	up	NM_199388	Dpagt1	300668
3.9201689	up	XM_238462	Dpf3	299186
4.7308307	up	NM_001017449	Dph5	295394
12.112689	up	XM_001072659	Dpm1	296394
3.645161	up	NM_019252	Dpm2	29640
13.592515	up	NM_053748	Dpp3	114591
5.8132205	up	NM_031973	Dpp7	83799
38.673195	up	XM_001075075	Dpp8	315758
100.06734	up	XM_001071146	Dpt	289178
2.3885372	up	XM_001053132	Dpy19l1	315496
2.0308588	up	XM_001072543	Dpy19l4	297824
7.86553	up	NM_173117	Dpy30	286897
2.747232	up	XM_001057161	Dqx1	680434
2.053006	up	NM_001011914	Dr1	289881
7.7779074	up	NM_001025018	Dram2	362011
17.014408	up	NM_001077668	Drap1	293674
7.6902976	up	NM_001009685	Drg1	305470
3.2078238	up	XM_001076012	Drg2	497915
2.7138276	up	XM_001054846	Dscr3	360703
2.3675125	up	XM_001053297	Dse	365583
13.229591	up	XM_001054738	Dst	316313
2.3576405	up	NM_001033666	Dstn	502674
6.6666517	up	XM_215862	Dstnl1	296197
4.3730726	up	XM_001054053	Dtna	307548
3.9372625	up	NM_001037664	Dtnbp1	641528
2.8794591	up	XM_001068990	Dtx2-ps1	688965
2.2177856	up	XM_001055773	Dtx3	500847
6.4961543	up	XM_001071259	Dtymk	301622
10.1732235	up	XM_001057815	Dupd1	361003
6.053137	up	XM_001076110	Dus2l	291978
2.8307447	up	NM_001034923	Dus3l	301122
8.317096	up	NM_001025650	Dusp11	297412
22.702885	up	NM_001007006	Dusp13	361002
8.924702	up	XM_001061713	Dusp22	361242
8.338146	up	NM_001012352	Dusp26	306527
11.98565	up	XM_001071732	Dusp27	498267
29.548288	up	XM_001081500	Dusp3	498003
2.7408986	up	NM_053883	Dusp6	116663
3.2348907	up	NM_001040271	Dut	497778
30.887226	up	XM_001054727	Dym	291433
91.380714	up	NM_019226	Dync1h1	29489

19.468027	up	NM_053880	Dync1i2	116659
5.2206335	up	NM_145772	Dync1li1	252902
4.8853507	up	NM_001013940	Dync2li1	298767
5.151352	up	NM_053319	Dynll1	58945
162.1404	up	NM_131910	Dynlrb1	170714
6.0798545	up	NM_001013228	Dynlt3	363448
23.17751	up	NM_012791	Dyrk1a	25255
2.6329486	up	XM_001078118	Dyrk1b	308468
83.69803	up	XM_001069038	Dysf	312492
79.8219	up	XM_001081801	Dysfip1	287881
3.4639726	up	XM_001053473	Dzip1	364475
19.051497	up	XM_001069459	E2f6	313978
2.5294743	up	XM_001075520	Eapp	299043
9.896728	up	NM_001009665	Ebag9	299864
3.5472925	up	NM_053820	Ebf1	116543
14.453445	up	NM_001008721	Ebna1bp2	114021
2.1378546	up	NM_057137	Ebp	117278
3.8931668	up	XM_001063508	Ebpl	361054
3.7706282	up	NM_053596	Ece1	94204
95.273384	up	NM_022594	Ech1	64526
2.6338818	up	NM_001007734	Echdc1	361465
28.207912	up	NM_078623	Echs1	140547
4.6793027	up	NM_053882	Ecm1	116662
2.5490167	up	XM_342698	Ecop	362374
39.652122	up	NM_001006986	Ecsit	300447
16.280397	up	XM_001068241	Eda2r	296872
2.4214509	up	XM_001075914	Edem1	297504
4.708483	up	NM_001004230	Edem2	296304
4.8013816	up	XM_001077770	Edf1	296570
7.7498164	up	XM_001061674	Eed	293104
19.317778	up	NM_175838	Eef1a1	171361
44.053406	up	NM_012660	Eef1a2	24799
4.7105303	up	XM_001071102	Eef1b2	363241
24.086817	up	NM_001013104	Eef1d	300033
7.2625756	up	XM_001063564	Eef1e1	291057
14.379252	up	NM_001004223	Eef1g	293725
437.06116	up	NM_017245	Eef2	29565
8.254339	up	NM_012947	Eef2k	25435
11.417784	up	XM_001075510	Eefsec	500255
3.4860811	up	NM_001014088	Eepd1	315500
2.2380118	up	NM_001031648	Efhd2	298609
6.406709	up	XM_001066760	Efr3a	362923
6.564814	up	XM_001062746	Eftud1	308789
8.710235	up	XM_001081526	Eftud2	287739

4.3581343	up	NM_012842	Egf	25313
8.169683	up	NM_139104	Egfl7	245963
4.01881	up	NM_178334	Egln1	308913
69.10277	up	NM_001004083	Egln2	308457
19.164938	up	NM_019371	Egln3	54702
13.5047	up	NM_001025117	Ehbp1l1	309169
9.9210615	up	XM_001074569	Ehd1	293692
5.1555915	up	NM_001024897	Ehd2	361512
8.482793	up	NM_139324	Ehd4	192204
4.436219	up	NM_133606	Ehhadh	171142
2.058307	up	NM_212463	Ehmt2	361798
6.105665	up	NM_001025660	Ei24	300514
7.4254327	up	XM_001081454	Eif1	287703
7.6316195	up	NM_001008773	Eif1a	317163
9.995291	up	XM_001054716	Eif1ay	302697
6.848189	up	XM_001077846	Eif1b	301068
22.487505	up	XM_001058163	Eif2a	502531
12.316049	up	NM_013223	Eif2ak1	27137
3.6244686	up	XM_579433	Eif2ak2	54287
2.7183638	up	XM_001074965	Eif2ak4	114859
10.713767	up	NM_172029	Eif2b1	64514
83.9747	up	NM_032058	Eif2b2	84005
24.59795	up	NM_053950	Eif2b4	117019
5.81123	up	NM_021597	Eif2c2	59117
9.437784	up	NM_019356	Eif2s1	54318
19.576998	up	NM_199380	Eif2s2	296302
27.873362	up	XM_001056580	Eif2s3x	299027
78.51802	up	NM_001047087	Eif3a	292148
20.21763	up	NM_001031640	Eif3b	288516
59.469078	up	XM_001079906	Eif3c	293484
25.516136	up	NM_001004283	Eif3d	362952
16.94735	up	NM_001011990	Eif3e	299872
84.768456	up	XM_001072798	Eif3f	293427
17.641392	up	NM_001013095	Eif3g	298700
134.39955	up	NM_198751	Eif3h	299899
47.104023	up	XM_001061306	Eif3i	682390
11.60559	up	XM_001074873	Eif3k	292762
19.175589	up	NM_001034134	Eif3s6ip	300069
18.389622	up	NM_199372	Eif4a1	287436
42.94702	up	NM_001008335	Eif4a2	303831
16.133432	up	XM_001081786	Eif4a3	688288
15.968724	up	NM_001008324	Eif4b	300253
3.7774777	up	NM_053974	Eif4e	117045
40.644436	up	XM_001081452	Eif4e2	363275

4.1744013	up	XM_001077522	Eif4e3	297481
2.8220382	up	NM_053857	Eif4ebp1	116636
23.822023	up	XM_001066327	Eif4enif1	305468
19.19962	up	XM_001060756	Eif4g1	287986
29.992758	up	XM_001072139	Eif4g2-ps1	171362
10.799395	up	XM_001062415	Eif4g2-ps2	308446
124.80731	up	XM_001070313	Eif4g3	298573
10.997259	up	NM_020075	Eif5	56783
5.212151	up	NM_001033681	Eif5a	287444
4.7148824	up	XM_001055509	Eif5b	308306
3.4091094	up	NM_001012181	Elf2	361944
2.440132	up	XM_001080272	Elk3	362871
6.343751	up	XM_001070626	Ell	306347
4.845451	up	XM_001054852	Ell2	309918
2.175192	up	XM_001072606	Elmo2	362271
4.5244517	up	NM_001013087	Elmod3	297342
5.929775	up	NM_001044275	Elovl1	679532
3.5889318	up	NM_134382	Elovl5	171400
23.90254	up	NM_001034145	Elp2	307545
4.197559	up	NM_022294	Eltd1	64124
4.0472236	up	NM_001004228	Emcn	295490
7.4128556	up	NM_012948	Emd	25437
14.758161	up	NM_001025741	Eml1	362783
2.4948714	up	XM_001061307	Eml4	313861
2.601069	up	NM_012843	Emp1	25314
6.7183056	up	NM_030847	Emp3	81505
2.4730675	up	NM_001007557	Emr1	316137
3.734067	up	NM_001012150	Enah	360891
3.779993	up	NM_001034938	Endog	362100
270.19907	up	NM_012949	Eno3	25438
6.4522343	up	NM_053535	Enpp1	85496
3.4057498	up	NM_057104	Enpp2	84050
5.6195073	up	NM_019370	Enpp3	54410
4.1980944	up	XM_001068428	Enpp4	301261
2.008869	up	NM_001012744	Enpp5	316249
32.846783	up	NM_001033974	Ensa	60334
18.421719	up	XM_001069087	Entpd4	361063
14.99627	up	NM_199394	Entpd5	314312
15.847399	up	XM_001062411	Eny2	685258
2.9984226	up	XM_001078513	Ep400	304569
2.4584484	up	XM_001063302	Epb4.1	313052
2.5533025	up	XM_001060647	Epb4.1l4a	307514
10.8055315	up	XM_001055791	Epc2	362132
7.071785	up	XM_001077513	Epc2l1	499105

4.342083	up	NM_001007625	Epdr1	291180
52.4132	up	NM_001024238	Eprs	289352
11.387577	up	NM_001009424	Eps15	313474
2.7055845	up	XM_001072957	Eps8	312812
6.2689977	up	NM_001013229	Eral1	363646
2.2657382	up	NM_030836	Erap1	80897
10.364397	up	NM_017003	Erbb2	24337
3.1800768	up	NM_001031644	Ercc3	291703
2.3138285	up	XM_001073831	Ercc8	310071
4.1777205	up	NM_133397	Erg	170909
4.9775996	up	NM_001024984	Ergic2	297728
38.34517	up	XM_001066442	Ergic3	296306
17.799181	up	XM_001071982	Eri3	313535
3.4664042	up	XM_001075018	Erich1	306622
18.250711	up	XM_001059292	Erlec1	289874
4.76343	up	NM_184050	Ermp1	373544
3.7872689	up	NM_138528	Ero1l	171562
6.947916	up	NM_001008317	Erp44	298066
14.453097	up	NM_001014071	Errfi1	313729
4.2535877	up	XM_001055446	Esco1	680014
45.582615	up	XM_001071513	Esd	290401
23.128311	up	XM_001081317	Esf1	366203
2.7259386	up	NM_203336	Esrrg	360896
13.254503	up	NM_017249	Esyt1	29579
21.395716	up	NM_001008344	Etf1	307503
8.308592	up	NM_001004220	Etfb	292845
27.32899	up	NM_198742	Etfdh	295143
12.44283	up	XM_001076689	Ethe1	292710
5.991896	up	XM_001069190	Etl4	291356
2.1775198	up	XM_001053903	Ets2	304063
4.923654	up	XM_001065285	Ewsr1	289752
2.7836795	up	XM_001053206	Exd2	362759
7.560633	up	NM_001024964	Exoc3	252881
3.2226472	up	XM_001063828	Exoc4	116654
3.555889	up	NM_022204	Exoc5	60627
4.784656	up	NM_019277	Exoc6	50556
5.184069	up	XM_001077923	Exosc2	366017
10.8546505	up	XM_001071395	Exosc3	313243
24.987085	up	XM_218343	Exosc5	308441
11.042989	up	XM_001078932	Exosc7	316098
4.3170047	up	XM_001055849	Exosc8	295050
12.630833	up	NM_001025406	Exosc9	294975
3.7406917	up	XM_001065900	Ext1	299907
3.260896	up	XM_001078606	Ext2	311215

2.4535372	up	XM_001068341	Extl1	313610
4.0482764	up	NM_020097	Extl3	56819
2.2251322	up	XM_001054107	Eya4	292172
2.5062032	up	XM_001081456	Ezh1	303547
2.4375002	up	XM_001073768	Ezh2	312299
4.374569	up	NM_022924	F2	29251
2.0862315	up	NM_183331	F8	302470
15.203831	up	NM_024162	Fabp3	79131
3.9672627	up	NM_053365	Fabp4	79451
6.4360747	up	NM_053445	Fads1	84575
5.515426	up	NM_173137	Fads3	286922
3.6017206	up	NM_080895	Faim	140930
2.6982455	up	NM_001014843	Faim3	548326
6.4951196	up	XM_001081585	Falz	303617
2.4914212	up	NM_001007668	Fam100a	302941
17.578775	up	NM_001034958	Fam104a	619573
2.854832	up	NM_001025129	Fam107a	361018
10.383375	up	NM_001014028	Fam108b1	309399
3.4703546	up	XM_001068403	Fam108c1	361601
2.166377	up	NM_001024341	Fam110b	500400
2.866578	up	XM_001072497	Fam115a	362353
3.0518217	up	XM_001057380	Fam116a	306229
3.445173	up	XM_001054735	Fam116b	362983
3.5609355	up	XM_001069873	Fam117b	363236
3.4465306	up	NM_001025283	Fam118b	315549
2.5549514	up	XM_001071910	Fam119a	301466
2.4611452	up	NM_001014029	Fam122a	309420
3.1232402	up	NM_199375	Fam125a	290635
2.1423385	up	XM_001061478	Fam126a	499975
4.41354	up	NM_001024799	Fam133b	362320
3.0910583	up	XM_001057799	Fam134a	363252
2.990064	up	XM_001081451	Fam134c	360632
3.209146	up	XM_001068890	Fam135a	367235
2.7142546	up	XM_001061083	Fam13b1	291694
5.827776	up	XM_001066002	Fam151b	499507
3.321849	up	NM_001008296	Fam158a	290224
3.4020135	up	NM_001029903	Fam162a	360721
3.9605966	up	XM_001058011	Fam164a	310244
4.0219374	up	XM_001061134	Fam173a	287150
2.4806228	up	XM_001066063	Fam173b	499561
3.0212903	up	XM_001054804	Fam175b	293570
4.0056305	up	XM_001062922	Fam178a	499360
4.258141	up	XM_001067322	Fam179a	313892
2.3556738	up	XM_001072337	Fam188a	291320

5.866617	up	XM_001078764	Fam189a2	309415
3.2821813	up	XM_001077686	Fam18b2	687358
2.2042274	up	XM_001067482	Fam18b-ps1	309146
2.8786778	up	XM_001059938	Fam193a	305452
6.8503532	up	XM_001069211	Fam193b	498703
2.5435598	up	XM_001081799	Fam195b	360677
2.58603	up	XM_001068245	Fam20b	304885
10.34601	up	NM_199207	Fam21c	297530
4.0259914	up	XM_001059804	Fam36a	289278
34.859993	up	NM_001077200	Fam38a	361430
2.1011982	up	XM_217218	Fam46a	300870
4.622716	up	XM_001073211	Fam49a	298890
20.458042	up	XM_001066035	Fam49b	299909
6.794	up	XM_001055598	Fam50a	293862
49.895233	up	NM_001013935	Fam54b	298549
2.006169	up	XM_001060299	Fam55c	681096
11.492837	up	NM_001025118	Fam63a	310665
2.1220276	up	XM_001054973	Fam63b	363089
5.977172	up	XM_001060943	Fam69a	360906
2.1891758	up	XM_001066463	Fam70b	290877
4.254561	up	XM_001065867	Fam76a	362618
2.6961532	up	NM_001037200	Fam82a1	313840
7.1403117	up	NM_001014046	Fam82a2	311328
3.6569412	up	XM_001053730	Fam92a1	297903
10.073448	up	NM_001008327	Fam96a	300797
6.1034393	up	NM_001014073	Fam98a	313873
2.5741117	up	XM_001064256	Fancl	305600
3.5766034	up	XM_001070569	Farp2	316639
8.463699	up	NM_001013139	Fars2	306879
13.418965	up	NM_001024237	Farsa	288917
23.925951	up	NM_001004252	Farsb	301544
2.0308769	up	NM_017332	Fasn	50671
48.619324	up	NM_001011967	Fastk	296741
3.277276	up	XM_001081175	Fastkd5	366192
7.82764	up	NM_031819	Fat1	83720
4.031611	up	NM_001012739	Fau	29752
2.3637311	up	XM_001062865	Fbln2	282583
6.5073223	up	NM_019153	Fbln5	29158
2.790307	up	NM_031825	Fbn1	83727
2.1950765	up	XM_232330	Fbxl14	312675
3.9417975	up	XM_001071661	Fbxl17	316663
9.655438	up	XM_001076775	Fbxl3	306129
5.935544	up	XM_001055670	Fbxl4	313101
9.833868	up	XM_001060082	Fbxl5	305424

2.1874087	up	NM_001005563	Fbxl6	362941
2.6282084	up	XM_001075450	Fbxl8	498941
4.8845983	up	NM_181631	Fbxo11	301674
3.9538398	up	XM_001069012	Fbxo18	291293
16.078798	up	NM_001037770	Fbxo22	300724
2.0036814	up	NM_001014239	Fbxo25	364637
2.646492	up	XM_001063044	Fbxo28	305105
34.581577	up	XM_001064070	Fbxo3	690634
3.9413717	up	NM_001007690	Fbxo30	308283
178.3578	up	NM_001044259	Fbxo31	498959
36.494404	up	NM_133521	Fbxo32	171043
11.665233	up	XM_001073518	Fbxo34	305830
9.687648	up	XM_001059463	Fbxo38	307390
3.1786835	up	XM_001054338	Fbxo4	310363
2.4848971	up	XM_001074239	Fbxo44	500587
237.17917	up	NM_138917	Fbxo6	192351
4.630184	up	NM_001012050	Fbxo8	306436
3.2214491	up	NM_001011998	Fbxo9	300849
2.7615933	up	XM_001063835	Fbxw11	303024
2.367495	up	XM_001080206	Fbxw2	311881
9.700541	up	XM_001058429	Fbxw4	309444
25.223045	up	NM_001025730	Fbxw5	362081
26.080494	up	NM_033351	Fcgrt	29558
3.6409202	up	NM_031348	Fcn1	83517
2.8590755	up	NM_019238	Fdft1	29580
4.9084163	up	NM_017126	Fdx1	29189
5.4089694	up	XM_001060280	Fech	361338
41.74082	up	NM_001025706	Fem1a	316131
2.9500706	up	XM_001053664	Fem1c	302288
71.93983	up	NM_001011915	Fermt2	289992
2.9595644	up	XM_001062414	Fert2	301737
23.033222	up	NM_053600	Fez2	94269
22.498165	up	NM_053428	Fgf13	84488
7.1687593	up	NM_024146	Fgfr1	79114
2.0227044	up	XM_001053923	Fgfr1l	500109
2.7269053	up	NM_201421	Fgfr1op2	362463
4.4239063	up	NM_001013932	Fggy	298250
5.5285306	up	NM_053455	Fgl2	84586
11.486305	up	NM_001033926	Fhl1	25177
16.663208	up	XM_001056311	Fhl3	313582
33.00927	up	XM_001072430	Fhod1	291964
2.916318	up	NM_001025042	Fibin	499856
5.1761265	up	NM_001010946	Ficd	288741
4.040925	up	NM_001047096	Fig4	309855

16.173454	up	NM_145682	Filip1	246776
12.502936	up	NM_001008295	Fip1l1	289582
29.13519	up	XM_001076962	Fis1	288584
51.224037	up	XM_001055627	Fitm1	290223
4.1796265	up	XM_001070393	Fitm2	311617
6.437015	up	NM_013102	Fkbp1a	25639
7.0275216	up	XM_001080093	Fkbp3	299104
6.4690633	up	XM_001066628	Fkbp4	260321
3.0070422	up	XM_215758	Fkbp7	295672
9.791819	up	NM_001037180	Fkbp8	290652
8.816365	up	NM_199390	Flcn	303185
95.21102	up	NM_001008279	Flii	287375
18.451206	up	XM_001056685	Flna	293860
4.4760666	up	XM_001071051	Flnb	306204
34.618732	up	XM_001061717	Flnc	362332
15.538179	up	NM_022701	Flot1	64665
2.7589018	up	NM_031830	Flot2	83764
2.6957273	up	XM_001064239	Flrt2	299236
6.9823003	up	NM_001037795	Fmc1	500087
2.0087686	up	NM_144737	Fmo2	246245
8.698572	up	XM_573235	Fn3k	498034
2.3026402	up	NM_138914	Fnbp1	192348
9.786998	up	XM_001065862	Fnbp4	311183
2.2540772	up	NM_001038615	Fndc1	308099
4.8114104	up	XM_001070746	Fndc3a	306022
2.2900367	up	XM_001057133	Fndc3b	294925
3.9637423	up	NM_172034	Fntb	64511
3.300373	up	NM_022197	Fos	314322
2.587575	up	NM_001013146	Fosb	308411
5.9149103	up	NM_012954	Fosl2	25446
3.112594	up	XM_001060923	Foxj2	502886
2.0912926	up	XM_001074052	Foxj3	313554
9.351596	up	XM_001065012	Foxn3	314374
2.8094797	up	XM_001056726	Foxo1	84482
2.0613086	up	XM_001056575	Foxp2	500037
11.840496	up	XM_235988	Foxred1	315547
2.8467212	up	XM_001070964	Frg1	689484
3.4857748	up	XM_001064153	Frg1l	361156
2.8957355	up	XM_001064688	Frmd6	257646
4.3838553	up	XM_001073918	Frrs1	310810
2.0494108	up	XM_001080960	Frs2	314850
8.252795	up	NM_012561	Fst	24373
57.341846	up	NM_012848	Fth1	25319
4.0235276	up	NM_022500	Ftl	29292

10.070874	up	NM_001039713	Fto	291905
3.9536126	up	XM_001062916	Ftsj1	363450
2.2155905	up	NM_001037653	Fubp1	654496
5.3169756	up	NM_012562	Fuca1	24375
19.289387	up	NM_001004218	Fuca2	292485
2.7404249	up	NM_001025027	Fundc1	363442
12.108043	up	XM_001059050	Fundc2	361288
6.7243705	up	NM_001025738	Fusip1	362630
14.305525	up	NM_001002289	Fut8	432392
2.8853009	up	NM_053371	Fxc1	84384
75.83663	up	NM_001012179	Fxr1	361927
3.592414	up	XM_001079469	Fxr2	287433
99.01101	up	NM_031648	Fxyd1	58971
2.4541585	up	NM_022008	Fxyd7	63848
4.1360784	up	XM_001078714	Fyco1	301085
10.050633	up	NM_001047899	Fyttd1	360726
9.330592	up	XM_001074757	G3bp1	171092
13.5664015	up	NM_001013989	G3bp2	305240
80.49585	up	NM_176077	G6pc3	303565
10.701765	up	NM_017006	G6pd	24377
21.16592	up	NM_199118	Gaa	367562
3.4734426	up	XM_001070145	Gab1	361388
29.918383	up	NM_172036	Gabarap	58974
137.00845	up	NM_001044294	Gabarapl1	689161
11.359174	up	NM_022706	Gabarapl2	64670
2.843493	up	XM_001065754	Gabpa	363735
3.0321655	up	XM_001054003	Gabpb1l	364738
5.590116	up	NM_017292	Gabrr2	29695
36.265644	up	NM_024127	Gadd45a	25112
3.3171532	up	NM_001008321	Gadd45b	299626
8.742067	up	NM_001077640	Gadd45g	291005
3.3854597	up	XM_001071308	Gadd45gip1	288916
6.7124395	up	NM_001013919	Galk2	296117
4.3997765	up	NM_001007704	Galm	313843
7.8001943	up	NM_024373	Galnt1	79214
2.2172322	up	NM_199393	Galnt11	311952
4.1622744	up	XM_001054081	Galnt2	292090
2.573398	up	NM_001025053	Galnt4	500826
2.3352916	up	NM_031796	Galnt5	83627
3.8248353	up	NM_001013089	Galt	298003
9.37661	up	XM_001077792	Ganab	293721
2.4416687	up	NM_017008	Gapdh	24383
2.8153155	up	XM_001070609	Gapdh-ps2	689689
10.045068	up	XM_001078902	Gapvd1	311880

23.238592	up	XM_001061006	Gars	297113
97.86661	up	NM_057100	Gas6	58935
4.161009	up	XM_001068512	Gatad1	500005
4.1156397	up	NM_001024888	Gatad2b	310614
2.2098882	up	XM_001077390	Gatc	360821
2.6168983	up	NM_001025128	Gatsl3	360969
8.308957	up	XM_001071921	Gba	684536
3.994475	up	XM_001069986	Gba2	298399
2.8689656	up	NM_001017486	Gbas	498174
3.9917114	up	XM_001066196	Gbf1	309451
2.7361007	up	XM_001078913	Gbp1	304266
5.5388074	up	XM_001055585	Gca	295647
3.968498	up	XM_001061993	Gcap14	306306
4.801658	up	XM_001053446	Gcc2	309798
26.236864	up	XM_001066810	Gcdh	364975
2.0845668	up	XM_001072034	Gcn1l1	690632
2.445114	up	NM_031749	Gcs1	78947
5.9799323	up	NM_133598	Gcsh	171133
8.037739	up	NM_031776	Gda	83585
27.552885	up	NM_032615	Gde1	60418
62.949806	up	NM_017088	Gdi1	25183
33.209167	up	NM_017276	Gdi2	29662
4.152098	up	NM_001044238	Gdpd1	303407
2.6171103	up	NM_053625	Gfm1	114017
2.3631608	up	NM_001002819	Gfpt2	360518
7.841916	up	NM_001011994	Gga1	300066
5.0525155	up	XM_001076216	Gga2	293455
3.714512	up	XM_001058292	Ggct	362368
4.1292477	up	NM_031756	Ggcx	81716
13.370792	up	NM_001004273	Ggnbp2	360584
75.1472	up	NM_001005908	Ghitm	290596
5.2858477	up	NM_017094	Ghr	25235
2.4197788	up	NM_173153	Gimap4	286938
3.1300523	up	NM_001024328	Gimap7	500113
2.8492825	up	NM_001008398	Gimap9	493865
2.009646	up	NM_001030027	Gins4	290842
11.816988	up	NM_053341	Gipc1	83823
3.2868266	up	NM_031814	Git1	83709
7.2971296	up	NM_001085381	Gja7	266706
5.001955	up	NM_001012160	Gkap1	361202
3.0035496	up	XM_001076727	Glb1	316033
3.6141427	up	NM_001025731	Gle1	362098
11.107749	up	NM_017211	Glg1	29476
36.39629	up	NM_207594	Glo1	294320

2.6265671	up	NM_001014227	Glod4	363644
7.742	up	NM_001013034	Glrx2	114022
9.972733	up	NM_032614	Glrx3	58815
6.101844	up	XM_343103	Glrx5	362776
2.819177	up	NM_012569	Gls	24398
2.8064868	up	XM_001065807	Glt25d1	290637
2.5543704	up	NM_001007683	Glt8d1	306253
2.3103979	up	XM_001057023	Glt8d3	300173
9.43094	up	XM_001080376	Gltp	288707
32.45731	up	NM_207591	Gltscr2	292624
18.115318	up	NM_012570	Glud1	24399
263.75012	up	NM_017073	Glul	24957
2.6722562	up	NM_001033931	Gmcl1	312516
6.9061737	up	NM_181091	Gmfg	113940
2.8376682	up	NM_001025056	Gmppa	501167
12.333809	up	NM_001024754	Gmps	295088
2.0700984	up	NM_001013119	Gna13	303634
59.021423	up	NM_031035	Gnai2	81664
3.208934	up	NM_013106	Gnai3	25643
88.332405	up	NM_019132	Gnas	24896
2.576985	up	NM_030987	Gnb1	24400
3.4185355	up	NM_031037	Gnb2	81667
8.249986	up	NM_130734	Gnb2l1	83427
2.229209	up	NM_001013910	Gnb4	294962
5.2895913	up	NM_031770	Gnb5	83579
2.8486657	up	NM_053660	Gng10	114119
2.495447	up	XM_001067408	Gng12	114120
15.98726	up	NM_024377	Gng5	79218
4.2402096	up	NM_212500	Gnl1	309593
6.4562	up	NM_001025736	Gnl2	362593
9.718471	up	NM_175580	Gnl3	290556
15.605793	up	NM_053410	Gnpat	84470
2.1598697	up	XM_001066558	Gnpda1	683570
3.4751318	up	XM_001079967	Gnptab	362865
7.2186017	up	NM_001011989	Gns	299825
133.53139	up	XM_001061798	Golga4	501069
4.7598166	up	NM_001033065	Golga5	299258
7.5239162	up	NM_001007731	Golga7	361171
3.4999108	up	NM_138885	Golgb1	192243
4.486544	up	XM_001062460	Golim4	310526
6.022777	up	XM_001059699	Gopc	309774
2.2348857	up	NM_019385	Gorasp1	56082
25.626541	up	NM_001007720	Gorasp2	113961
3.61021	up	NM_053584	Gosr1	94189

4.2431674	up	NM_031685	Gosr2	64154
60.542168	up	NM_012571	Got1	24401
3.8371544	up	NM_013177	Got2	25721
14.703333	up	NM_001004240	Gpaa1	300046
2.4405255	up	XM_001079698	Gpatch1	292810
4.8916197	up	XM_215485	Gpbp1	294734
3.078027	up	NM_030828	Gpc1	58920
2.1462023	up	NM_012774	Gpc3	25236
2.0850823	up	NM_001014108	Gpc4	317322
4.399436	up	NM_198779	Gpcpd1	362219
20.508905	up	NM_012736	Gpd2	25062
309.65564	up	NM_207592	Gpi	292804
5.308951	up	XM_001074228	Gpihbp1	300027
19.537102	up	XM_001054977	Gpkow	679890
10.168443	up	XM_001066907	Gpn2	362614
7.5530405	up	NM_201991	Gpn3	360810
223.99608	up	NM_133298	Gpnmb	113955
3.4041314	up	NM_199399	Gpr108	316136
4.5692987	up	NM_139110	Gpr116	245977
2.7995374	up	XM_001062029	Gpr155	311730
9.595371	up	NM_001085353	Gpr177	362065
7.868728	up	NM_001006994	Gpr180	306165
3.4948757	up	NM_053302	Gpr182	29307
4.1069226	up	NM_080579	Gpr19	312787
4.2388644	up	XM_342301	Gpr89	362003
8.943698	up	NM_134386	Gprasp1	171407
16.816343	up	XM_001081657	Gprc5c	287805
27.446053	up	NM_053969	Gps1	117039
42.629486	up	NM_001017477	Gps2	497941
10.878362	up	NM_031039	Gpt	81670
2.370516	up	NM_001012057	Gpt2	307759
85.90793	up	NM_030826	Gpx1	24404
35.01681	up	NM_022525	Gpx3	64317
104.84265	up	NM_017165	Gpx4	29328
6.166978	up	NM_001014160	Gramd1a	361550
8.6302805	up	NM_030846	Grb2	81504
44.99193	up	NM_153308	Grina	266668
7.1769505	up	NM_053807	Gripap1	116493
6.031893	up	NM_017113	Grn	29143
3.128774	up	NM_024487	Grpel1	79563
14.326714	up	XM_001073361	Grsf1	305256
2.1307707	up	NM_001012067	Grwd1	308592
3.815719	up	XM_001078877	Gse1	307913
88.30377	up	NM_001004080	Gsn	296654

16.98802	up	NM_001003978	Gspt1	24420
3.9590628	up	NM_012962	Gss	25458
8.328517	up	NM_031509	Gsta3	24421
6.1786766	up	NM_181371	Gstk1	297029
40.670425	up	NM_017014	Gstm1	24423
44.362144	up	NM_177426	Gstm2	24424
24.059175	up	NM_031154	Gstm7	81869
24.869823	up	NM_001007602	Gsto1	114846
2.0092049	up	NM_001012071	Gsto2	309465
2.1589625	up	NM_012577	Gstp1	24426
5.3914723	up	NM_053293	Gstt1	25260
15.36814	up	XM_574740	Gstt3	499422
4.240498	up	XM_001061506	Gstz1	681913
3.9601097	up	NM_022208	Gtf2a1	83830
9.90446	up	NM_031041	Gtf2b	81673
2.777252	up	XM_001062046	Gtf2e1	303918
4.517548	up	XM_224929	Gtf2e2	306516
2.8169687	up	NM_001007711	Gtf2f1	316123
6.427992	up	NM_031042	Gtf2f2	81674
4.5448036	up	NM_001077428	Gtf2h2	294693
2.1912808	up	NM_001024236	Gtf2h3	288651
6.1722755	up	NM_212501	Gtf2h4	294236
8.717176	up	XM_001067341	Gtf2h5	502227
2.7616744	up	NM_001001504	Gtf2ird1	246770
9.00952	up	XM_001069721	Gtf3a	246299
2.1269107	up	NM_133541	Gtf3c1	171063
2.6725152	up	XM_001066298	Gtf3c3	316810
23.88163	up	XM_001063347	Gtf3c6	361858
2.9448307	up	NM_001037978	Gtl3	307642
6.128704	up	XM_001075632	Gtpbp1	300077
5.8255243	up	NM_053689	Gtpbp4	114300
12.788372	up	NM_001013924	Gtpbp5	296462
4.0361905	up	XM_001078867	Gtpbp6	363931
2.7076464	up	NM_001025015	Gtpbp8	360714
40.26099	up	NM_001013115	Guk1	303179
4.2578135	up	NM_017015	Gusb	24434
49.32309	up	NM_031043	Gyg1	81675
31.327028	up	NM_001013233	Gypc	364837
13.281381	up	XM_001076928	Gys1	690987
2.5739365	up	XM_001057286	Gzf1	311508
8.548839	up	XM_001062255	H13	311545
8.88439	up	NM_012578	H1f0	24437
29.579927	up	NM_017182	H2afy	29384
5.95661	up	NM_022674	H2afz	58940

61.250504	up	NM_053985	H3f3b	117056
26.111511	up	NM_001079940	Habp4	361196
3.6273675	up	XM_342160	Hace1	361866
13.104062	up	NM_057186	Hadh	113965
100.09784	up	NM_130826	Hadha	170670
140.93251	up	NM_133618	Hadhb	171155
6.9287033	up	NM_033349	Hagh	24439
7.4635167	up	NM_001009657	Hat1	296501
2.9325805	up	NM_138864	Haus1	192228
2.1692438	up	NM_001013995	Haus4	305882
2.226385	up	NM_001024971	Haus8	290626
2.4652565	up	NM_181627	Hax1	291202
28.234001	up	NM_013096	Hba-a2	25632
100.68018	up	NM_033234	Hbb	24440
42.76762	up	NM_198776	Hbb-b1	361619
10.444405	up	NM_012945	Hbegf	25433
12.295886	up	NM_001011934	Hbs1l	293408
17.483175	up	XM_001063786	Hcca2	499288
9.575412	up	XM_001065465	Hccs	317444
2.2139704	up	XM_001057067	Hcfc1	363519
31.583406	up	XM_001055621	Hcfc1r1	287097
2.3622277	up	NM_001011898	Hcls1	288077
3.3668046	up	XM_001053786	Hdac10	362981
15.738929	up	XM_001061582	Hdac2	84577
2.5039377	up	NM_053448	Hdac3	84578
110.71278	up	XM_001067733	Hdac4	363287
33.91094	up	XM_001081495	Hdac5	84580
2.2258148	up	XM_001070606	Hdac8	363481
7.2864146	up	XM_001061439	Hddc3	308758
9.655384	up	NM_053707	Hdgf	114499
11.366699	up	NM_001014151	Hdhd2	361351
79.11012	up	NM_172039	Hdlbp	64474
6.4334183	up	XM_001061994	Heatr1	361262
3.0596843	up	XM_001055205	Heatr2	304332
4.2780614	up	XM_001067127	Heatr3	361375
3.9282207	up	XM_001053592	Heatr5b	362683
2.7488086	up	NM_001079897	Heatr6	497972
3.6438732	up	XM_001071946	Hebp1	362454
2.039835	up	XM_001072434	Hebp2	308632
4.169251	up	XM_218660	Heca	308624
80.45994	up	XM_001078686	Hectd1	362736
18.548292	up	XM_001075834	Herc1	315771
55.22604	up	XM_001054649	Herc2	308669
6.234274	up	NM_001012074	Herc4	309758

2.7886872	up	XM_342700	Herc6	362376
10.902525	up	NM_024360	Hes1	29577
4.1525526	up	NM_001011946	Hexb	294673
31.65515	up	NM_001025136	Hexim1	498008
91.9022	up	NM_001012080	Hfe2	310681
7.2547545	up	NM_019387	Hgs	56084
27.50308	up	XM_001078610	Hhatl	301073
54.007465	up	NM_022243	Hibadh	63938
6.2324014	up	NM_001013112	Hibch	301384
2.9898436	up	XM_001062399	Hif1an	309434
12.721913	up	NM_080902	Higd1a	140937
64.17594	up	XM_001070385	Higd2a	290999
5.8092694	up	XM_001073678	Hint1	690660
10.577319	up	XM_001070128	Hint2	313491
2.6560323	up	XM_001055319	Hint3	246769
15.018383	up	XM_001061381	Hip1	192154
4.609581	up	XM_001072438	Hip1r	81917
5.227559	up	XM_001066649	Hipk2	362342
5.5237412	up	NM_031787	Hipk3	83617
2.0597496	up	NM_001024776	Hipk4	308449
7.605125	up	NM_001025725	Hirip3	361650
4.9141107	up	NM_001080783	Hisppd2a	311355
8.339988	up	NM_024137	Hivep2	29721
4.716896	up	NM_012734	Hk1	25058
23.779543	up	NM_012735	Hk2	25059
4.696543	up	NM_013168	Hmbs	25709
4.632376	up	XM_001073808	Hmg20a	315689
4.856156	up	XM_001075699	Hmg20b	362825
6.792794	up	NM_024386	Hmgcl	79238
2.6961265	up	NM_001025624	Hmgn2	114637
11.8015585	up	NM_001007020	Hmgn3	113990
2.0957363	up	NM_012580	Hmox1	24451
17.273066	up	NM_024387	Hmox2	79239
2.607902	up	NM_001005876	Hn1	287828
2.1416688	up	NM_001013182	Hn1l	360492
12.392668	up	XM_001055961	Hnrnpa2b1	362361
2.3859334	up	NM_031330	Hnrnpab	83498
7.5017366	up	NM_001025633	Hnrnpc	290046
6.060171	up	NM_001037285	Hnrnpf	64200
10.993094	up	NM_001014019	Hnrnph2	308650
8.838892	up	XM_001074684	Hnrnph3	361838
6.987735	up	NM_057141	Hnrnpk	117282
30.25975	up	XM_001068144	Hnrnpl	80846
2.3430486	up	XM_001076759	Hnrnpll2	311776

11.09117	up	NM_175603	Hnrnpr	319110
4.592057	up	NM_057139	Hnrnpu	117280
3.1420667	up	XM_001070479	Hnrnpul1	361522
32.046474	up	NM_001082539	Hnrpd	79256
18.44431	up	NM_001033696	Hnrpdl	305178
58.30203	up	NM_080896	Hnrph1	140931
8.319705	up	XM_001063027	Hnrpll	313842
7.5706534	up	NM_053876	Hnrpm	116655
9.229147	up	NM_031707	Homer1	29546
2.8338218	up	NM_053310	Homer3	29548
3.6722982	up	XM_224952	Hook3	306548
2.2114122	up	XM_001059031	Hoxa5	79241
3.393353	up	XM_001056901	Hoxa9	500126
2.7488277	up	XM_001063804	Hoxd10	303991
4.046643	up	NM_012582	Hp	24464
4.0609202	up	NM_199108	Hp1bp3	313647
2.5517006	up	NM_017356	Hpcal1	50871
26.67938	up	NM_012583	Hprt1	24465
2.7425978	up	XM_001078272	Hps4	304555
54.31829	up	XM_001061671	Hras	293621
2.216734	up	XM_001053962	Hrasls	288025
38.986057	up	NM_181369	Hrc	292905
2.3531003	up	NM_031714	Hrsp12	65151
38.909668	up	NM_173119	Hsbp1	286899
2.34647	up	NM_017081	Hsd11b2	25117
92.6102	up	NM_031682	Hsd17b10	63864
14.2525625	up	NM_001004209	Hsd17b11	289456
2.5204163	up	NM_032066	Hsd17b12	84013
2.3043456	up	NM_024392	Hsd17b4	79244
3.5904598	up	NM_017235	Hsd17b7	29540
4.442333	up	NM_212529	Hsd17b8	361802
2.2706358	up	NM_001024896	Hsdl1	361418
25.933773	up	NM_001025697	Hsdl2	313200
13.252213	up	XM_001061027	Hsf1	79245
2.9618044	up	NM_031694	Hsf2	64441
9.493953	up	XM_001075468	Hsf4	291960
7.626899	up	NM_175761	Hsp90aa1	299331
25.51519	up	NM_001004082	Hsp90ab1	301252
75.59389	up	NM_001012197	Hsp90b1	362862
5.18695	up	NM_019271	Hspa13	29734
2.3031456	up	NM_001004257	Hspa14	307133
2.133624	up	NM_212504	Hspa1b	294254
22.06096	up	NM_212546	Hspa1l	24963
18.416653	up	NM_153629	Hspa4	266759

76.377686	up	NM_013083	Hspa5	25617
6.201036	up	NM_024351	Hspa8	24468
24.516865	up	XM_001062585	Hspa9	291671
169.96977	up	NM_031970	Hspb1	24471
5.4541388	up	NM_130431	Hspb2	161476
7.701902	up	NM_031750	Hspb3	78951
506.24106	up	NM_138887	Hspb6	192245
15.663043	up	XM_001072790	Hspb7	50565
229.64351	up	NM_053612	Hspb8	113906
2.9294274	up	NM_134419	Hspbap1	171460
2.0335698	up	XM_341259	Hspc159	360983
15.063482	up	NM_022229	Hspd1	63868
24.563017	up	NM_012966	Hspe1	25462
72.57901	up	NM_001011901	Hsph1	288444
19.592255	up	XM_001080298	Htatip2	292935
7.538555	up	XM_001058638	Htatsf1	317612
19.919199	up	NM_031721	Htra1	65164
2.0519898	up	XM_001058820	Htt	29424
9.749488	up	NM_172040	Hyal2	64468
6.6088843	up	NM_001034028	Hyou1	192235
2.2168057	up	XM_230510	Hypk	311359
3.0513113	up	XM_001071149	Iah1	298917
15.754178	up	XM_001065536	Iars2	364070
6.1729817	up	XM_001062352	Ibtk	315858
3.408689	up	NM_138886	Ick	84411
4.192606	up	XM_001079346	Icoslg	499415
32.748947	up	XM_001081688	Ict1	303673
3.0695724	up	NM_013060	Id2	25587
10.012742	up	NM_013159	Ide	25700
11.224986	up	NM_031510	Idh1	24479
15.977509	up	NM_001014161	Idh2	361596
25.327963	up	NM_053638	Idh3a	114096
92.414505	up	NM_053581	Idh3B	94173
10.547809	up	NM_031551	Idh3g	25179
2.7677667	up	NM_053539	Idi1	89784
5.747473	up	NM_212505	Ier3	294235
4.8832765	up	NM_203410	Ifi27	170512
49.153385	up	NM_206846	Ifi27l2b	299269
4.676815	up	NM_001009625	Ifi35	287719
3.5829523	up	XM_001079908	Ifi44	310969
4.644787	up	NM_172019	Ifi47	246208
3.0466616	up	NM_001007694	Ifit3	309526
7.135775	up	XM_001059109	Ifitm1	293618
40.57527	up	XM_001059289	Ifitm3	361673

3.4751306	up	XM_001073651	Ifnar1	288264
10.560866	up	NM_053783	Ifngr1	116465
2.472905	up	XM_001073758	Ifngr2	360697
8.08774	up	NM_019242	Ifrd1	29596
77.71943	up	NM_001047871	Ifrd2	300994
9.827916	up	XM_001080863	Ift20	287541
5.6038995	up	XM_001058583	Ift57	303968
2.65034	up	NM_001007001	Ift74	313365
11.806455	up	NM_031624	Igbp1	58845
5.3348975	up	NM_001082478	Igf1	24482
3.0602314	up	NM_052807	Igf1r	25718
2.5986087	up	NM_031511	Igf2	24483
7.922814	up	NM_012756	Igf2r	25151
2.874102	up	NM_012817	Igfbp5	25285
5.064488	up	NM_013104	Igfbp6	25641
49.212345	up	NM_001013048	Igfbp7	289560
102.138954	up	XM_001063557	Igfn1	304823
3.1458046	up	NM_031586	Ighmbp2	29532
3.7389784	up	NM_080899	Ikbkap	140934
3.0613968	up	XM_001053740	Ikzf5	309031
3.692684	up	XM_221655	Il10rb	304091
6.241259	up	NM_139116	Il11ra1	245983
3.910087	up	NM_013129	Il15	25670
6.0430737	up	NM_080889	Il2rg	140924
2.6038258	up	NM_001025766	Il34	498951
5.78755	up	NM_001047886	Ilf2	310612
29.384583	up	NM_133409	Ilk	170922
2.2158065	up	XM_001074573	Ilvbl	362843
17.554836	up	NM_001034928	Immt	312444
5.6903477	up	XM_001074037	Imp3	315697
22.57194	up	NM_001009700	Imp4	316317
11.43417	up	NM_032057	Impa1	83523
7.947888	up	NM_001012235	Impact	497198
4.9161687	up	NM_001038591	Ing1	306626
7.438031	up	XM_001059606	Ing2	290744
8.956083	up	NM_001034107	Ing3	312154
16.073788	up	NM_001079887	Ing4	297597
4.175465	up	NM_001048233	Ino80b	500225
11.790158	up	NM_001017446	Ino80c	291737
2.447123	up	NM_001012131	Inpp1	316376
3.5269296	up	NM_031002	Inpp4a	80849
37.489285	up	XM_001058238	Inpp5a	365382
4.0770183	up	NM_001013859	Inpp5k	287533
6.0602593	up	NM_022392	Insig1	64194

8.768517	up	NM_178091	Insig2	288985
5.3514433	up	XM_001070525	Ints10	290679
2.3950891	up	NM_001007640	Ints12	295448
6.6518126	up	XM_001075102	Ints3	361988
6.682478	up	XM_218937	Ints4	308837
3.9775577	up	XM_001073616	Ints5	309200
12.157764	up	XM_001066109	Ints9	290322
5.636152	up	XM_001075731	Ip6k2	59268
7.6077394	up	NM_134417	Ipmk	171458
72.68903	up	NM_053778	Ipo13	116458
9.446173	up	XM_001056047	Ipo4	290228
9.713686	up	XM_001075101	Ipo5	306182
7.762934	up	XM_001077641	Ipo7	308939
2.6688166	up	NM_001008556	Ippk	306808
2.6067665	up	XM_001062923	Iqcb1	303915
2.7176092	up	XM_001066654	Iqgap1	361598
15.522755	up	XM_001057078	Irak1	363520
2.5393538	up	XM_001061980	Irak1bp1	300862
2.7625399	up	NM_001025422	Irak2	362418
2.4521494	up	XM_001079164	Irak3	314870
2.5168576	up	NM_022863	Ireb2	64831
2.8480895	up	NM_012591	Irf1	24508
2.0081673	up	NM_001047086	Irf2	290749
20.049347	up	NM_001033691	Irf7	293624
4.1804857	up	NM_001012007	Irgm	303090
56.763847	up	XM_001078081	Iscu	288740
7.9571004	up	XM_001077878	Isg15	298693
2.9832044	up	NM_001007741	Isg20l2	361977
6.7781425	up	NM_001008367	Isoc2b	361501
2.9587576	up	NM_001008386	Ispd	493574
7.8244195	up	NM_001005887	Itch	311567
17.574871	up	NM_133557	Itfg1	171083
2.1153855	up	XM_001066574	Itfg2	362441
5.0572653	up	NM_001009701	Itfg3	360502
2.9815395	up	NM_030994	Itga1	25118
3.1113112	up	XM_001067316	Itga4	311144
2.151934	up	XM_001068715	Itgav	296456
30.791431	up	XM_001070826	Itgb1bp1	298914
4.9520025	up	XM_001069915	Itgb1bp2	317258
15.639413	up	NM_001004263	Itgb6	311061
6.5144753	up	NM_001025712	Itm2a	317218
138.97632	up	NM_001006963	Itm2b	290364
2.061451	up	NM_001009674	Itm2c	301575
14.814597	up	XM_001081181	Itpa	311422

5.8935013	up	NM_001007235	Itpr1	25262
2.1398249	up	XM_001069789	Itsn1	29491
76.01938	up	NM_012592	Ivd	24513
15.753736	up	NM_001047085	Ivns1abp	289089
27.533915	up	NM_001044272	Jagn1	502872
16.370481	up	XM_001061647	Jak1	84598
5.164202	up	NM_031514	Jak2	24514
5.548139	up	NM_001034004	Jam2	619374
45.735092	up	XM_001077497	Jkamp	299127
8.796921	up	XM_001080424	Jmjd1c	171120
9.480103	up	NM_001012143	Jmjd6	360665
10.686122	up	XM_001080592	Josd2	292876
21.150877	up	XM_001059299	Jph1	297748
2.0218694	up	XM_001073711	Jrkl	315417
15.988898	up	XM_001059651	Jsrp1	690423
14.488845	up	NM_019213	Jtb	29439
30.529383	up	NM_021835	Jun	24516
15.281739	up	NM_031047	Jup	81679
5.7519083	up	XM_001055725	Kank1	309429
28.092123	up	NM_001006967	Kars	292028
6.072739	up	XM_001081432	Kat2a	303539
7.9643044	up	NM_001024746	Katnb1	291852
265.92163	up	NM_057191	Kbtbd10	117537
7.07583	up	XM_001061645	Kbtbd2	312372
4.6794343	up	XM_001077889	Kbtbd4	311185
105.609634	up	XM_001078108	Kbtbd5	316088
3.7118073	up	NM_013186	Kcnb1	25736
2.3693786	up	XM_001070801	Kcnc4	684516
4.8546696	up	NM_031358	Kcnj11	83535
15.840952	up	NM_053981	Kcnj12	117052
7.8273373	up	NM_031828	Kcnma1	83731
20.374784	up	NM_019315	Kcnn3	54263
6.971181	up	XM_001069745	Kcnq5l	259273
5.573368	up	XM_001062473	Kctd18	301436
5.9504805	up	XM_001081684	Kctd2	498024
2.0343952	up	XM_001078592	Kctd20	294307
4.631237	up	XM_001070711	Kctd6	305792
4.8687735	up	XM_001068535	Kctd9	364410
3.0417151	up	XM_001060354	Kdelc1	316370
14.302342	up	NM_001017385	Kdelr1	361577
15.876909	up	NM_001013122	Kdelr2	304290
5.039495	up	XM_001069483	Kdm1	500569
3.4468029	up	XM_001075313	Kdm2b	304495
5.284765	up	NM_175764	Kdm3a	312440

5.1450152	up	XM_001067369	Kdm4c	298144
3.8749747	up	XM_001062221	Kdm5b	304809
4.775218	up	XM_001079385	Kdm6b	363630
2.2888114	up	XM_001064145	Kdsr	360833
3.108126	up	NM_001037220	Kiaa0415	641386
5.38381	up	XM_001053202	Kif16b	311478
15.666155	up	NM_057200	Kif1b	117548
4.5465493	up	XM_001056804	Kif21a	300158
2.821402	up	XM_001057533	Kif2a	84391
2.531004	up	XM_001061322	Kif3b	296284
5.6690784	up	NM_053486	Kif3c	85248
12.808537	up	NM_057202	Kif5b	117550
2.05927	up	XM_001065361	Kif7	293047
2.7604325	up	XM_001061174	Kifc3	307644
3.7629306	up	NM_001081972	Klc1	171041
3.219097	up	NM_138520	Klc3	171549
2.1534317	up	NM_001009601	Klc4	316226
6.2783685	up	NM_053536	Klf15	85497
8.820414	up	XM_223423	Klf3	114845
8.006994	up	NM_031642	Klf6	58954
12.5123415	up	NM_057211	Klf9	117560
4.812831	up	XM_001080363	Klhdc1	314190
3.3538742	up	NM_001017456	Klhdc10	312199
23.960344	up	NM_001034133	Klhdc2	299113
12.682559	up	NM_001012203	Klhdc3	363192
4.1973047	up	NM_001007685	Klhdc8b	306589
5.560457	up	XM_001061200	Klhl13	313445
7.5697427	up	XM_214331	Klhl2	290692
3.8905427	up	XM_001063385	Klhl22	303792
28.067398	up	XM_001067145	Klhl30	316624
5.0328417	up	XM_001058807	Klhl31	315833
2.5846765	up	NM_001025008	Klhl38	314996
2.3499844	up	NM_001047093	Klhl5	305351
17.636658	up	NM_001012187	Klhl7	362303
4.5273027	up	XM_001065538	Klhl8	289457
7.477376	up	XM_001054585	Klhl9	313348
33.540714	up	NM_001014792	Kpna3	361055
4.737711	up	NM_001015029	Kpna6	362607
12.731311	up	NM_017063	Kpnb1	24917
3.9465063	up	XM_001074187	Kptn	308107
14.69965	up	NM_001009413	Krcc1	312437
8.791628	up	XM_001064073	Krit1	362317
23.777357	up	XM_001072393	Krtcap2	295243
11.646726	up	XM_001080891	Ksr1	360573

2.728981	up	XM_001069417	Ktelc1	288091
6.415448	up	NM_001044288	Kti12	685656
7.839483	up	XM_341305	Ktn1	361029
2.3417687	up	XM_001071908	Ky	315962
3.6065366	up	XM_001080412	L2hgdh	314196
2.447569	up	XM_001062517	L3mbtl3	309550
3.3999014	up	XM_001056675	Lactb	300803
15.709045	up	NM_001024247	Lactb2	297768
5.893521	up	XM_001056585	Lage3	293863
4.9660983	up	XM_001061207	Lama4	309816
2.6918347	up	XM_001060527	Lama5	140433
5.6452518	up	XM_001075963	Lamb1	298941
5.6099634	up	NM_012974	Lamb2	25473
7.104297	up	XM_001071300	Lamc1	117036
107.858246	up	NM_012857	Lamp1	25328
4.558096	up	NM_053723	Lancl1	114515
10.297257	up	NM_001011910	Lap3	289668
114.417114	up	NM_199384	Laptm4a	298875
4.3557467	up	NM_053538	Laptm5	89783
2.3577387	up	XM_001060350	Large	361368
18.401789	up	NM_001014036	Larp1b	310348
3.7906024	up	XM_001065835	Larp5	307070
3.4603555	up	NM_001044290	Larp7	686883
7.133808	up	NM_001033700	Lass2	310667
6.5928397	up	XM_001063108	Lass5	366984
4.891243	up	NM_134453	Lbr	89789
2.4828644	up	NM_001047108	Lbx1	499362
4.2082953	up	XM_001060474	Lclat1	362702
2.7866611	up	NM_199405	Lcmt1	361643
2.2174733	up	NM_001011956	Lcmt2	296098
6.081969	up	XM_001058773	Ldb1	309447
2.0469	up	XM_001059368	Ldb2	289664
47.76684	up	XM_001059372	Ldb3	498587
118.178894	up	NM_017025	Ldha	24533
21.94084	up	NM_012595	Ldhb	24534
2.1342864	up	XM_575931	Ldlrap1	500564
2.5947826	up	XM_001055542	Lemd3	680066
4.564215	up	XM_001056203	Leng4	308309
2.338344	up	XM_001058154	Leng8	361506
12.120675	up	NM_001005548	Leo1	300837
2.0712345	up	XM_001062610	Leprel2	297595
28.540506	up	NM_020099	Leprot	56766
14.388309	up	NM_001013188	Leprotl1	361160
5.3000216	up	NM_001005884	Letm1	305457

2.364453	up	XM_001061371	Letmd1	681352
113.18862	up	NM_019904	Lgals1	56646
9.241762	up	NM_031832	Lgals3	83781
2.8459177	up	NM_139096	Lgals3bp	245955
8.749401	up	NM_053862	Lgals8	116641
4.189323	up	NM_012977	Lgals9	25476
27.266754	up	NM_022226	Lgmn	63865
13.586442	up	NM_001017489	Lgtn	498225
2.0822794	up	XM_001076874	Lgtnl1	308432
20.984581	up	NM_001012037	Lias	305348
2.1361122	up	NM_001012011	Lig3	303369
8.206424	up	XM_001077838	Limch1	305332
2.477243	up	XM_001076215	Lin37	292787
2.1408243	up	XM_001059713	Lins1	308704
2.5050826	up	NM_012732	Lipa	25055
2.425815	up	XM_001057736	Lipt1	316342
3.0747674	up	XM_001066655	Lipt2	365314
10.40081	up	XM_001078236	Litaf	65161
6.3757033	up	NM_053886	Lman1	116666
28.771368	up	NM_139189	Lmbrd1	246046
3.004168	up	NM_001008562	Lmcd1	494021
18.700571	up	NM_001002016	Lmna	60374
4.936631	up	NM_001037358	Lmo2	362176
24.819864	up	NM_001001515	Lmo7	361084
107.15271	up	XM_001060472	Lmod2	296935
16.004538	up	XM_575617	Lmod3	500267
7.0239663	up	NM_001013853	LOC287167	287167
25.82859	up	NM_198728	LOC288913	288913
15.144119	up	XM_001066750	LOC289378	289378
3.4267685	up	XM_001054611	LOC292662	292662
23.330605	up	NM_001013897	LOC293103	293103
7.3438754	up	XM_001056818	LOC293589	293589
7.3796167	up	NM_001039607	LOC294154	294154
5.2961693	up	XM_001067587	LOC295398	295398
14.424301	up	XM_001059497	LOC296165	296165
2.9490364	up	XM_001063958	LOC297828	297828
11.1556635	up	NM_001013930	LOC298139	298139
4.1833787	up	XM_001060121	LOC301124	301124
7.851447	up	NM_001044236	LOC301128	301128
4.8965406	up	XM_001070285	LOC302495	302495
2.6205795	up	NM_001037187	LOC302680	302680
9.031594	up	NM_001013979	LOC304131	304131
5.161665	up	NM_001013986	LOC305076	305076
6.5653214	up	NM_001014007	LOC306766	306766

3.9623106	up	XM_226278	LOC307671	307671
40.070553	up	XM_001068023	LOC307752	307752
5.3186965	up	XM_001063457	LOC310177	310177
6.6338525	up	NM_001009694	LOC314140	314140
14.646366	up	NM_001007722	LOC360504	360504
8.863381	up	XM_001080824	LOC360570	360570
2.8562732	up	XM_001067768	LOC361100	361100
4.8269696	up	NM_001047104	LOC361128	361128
5.025674	up	NM_001017463	LOC361635	361635
43.574585	up	NM_001014174	LOC361985	361985
6.4853015	up	NM_001014175	LOC361990	361990
4.9872828	up	NM_001025021	LOC362526	362526
2.735955	up	NM_207614	LOC362855	362855
3.7263243	up	XM_001061775	LOC363188	363188
2.0564613	up	XM_001056019	LOC363418	363418
2.548817	up	XM_001068543	LOC363529	363529
7.417733	up	XM_001065988	LOC363903	363903
2.6437993	up	XM_001061044	LOC364091	364091
8.932496	up	XM_001067967	LOC364984	364984
2.082305	up	XM_001061885	LOC365050	365050
16.904621	up	XM_001079986	LOC365370	365370
2.0133204	up	XM_001069260	LOC365824	365824
2.4317193	up	XM_001058386	LOC366068	366068
5.10566	up	NM_001014260	LOC366431	366431
3.736352	up	XM_345689	LOC366669	366669
3.6925654	up	XM_001054587	LOC366864	366864
2.381293	up	XM_001066861	LOC497729	497729
2.4211836	up	NM_001017474	LOC497934	497934
2.2386465	up	XM_001061569	LOC498122	498122
2.3298764	up	XM_001069849	LOC498249	498249
42.425896	up	XM_001063181	LOC498369	498369
6.763234	up	XM_001059576	LOC498453	498453
5.9006777	up	XM_001075110	LOC498555	498555
7.320557	up	XM_001064371	LOC498685	498685
16.180319	up	NM_001017510	LOC498750	498750
3.736441	up	XM_001054141	LOC498752	498752
2.359056	up	XM_001078842	LOC499124	499124
63.27731	up	XM_001076383	LOC499235	499235
4.5394635	up	NM_001025036	LOC499339	499339
2.3732598	up	NM_001025039	LOC499602	499602
8.289109	up	XM_001075928	LOC499706	499706
3.398907	up	XM_001078222	LOC499718	499718
4.027454	up	NM_001047110	LOC499770	499770
6.0882297	up	XM_001078515	LOC499782	499782

2.389135	up	XM_001057511	LOC500034	500034
2.085145	up	XM_001061323	LOC500046	500046
2.6221893	up	XM_001068033	LOC500195	500195
9.214297	up	NM_001024334	LOC500300	500300
3.7554445	up	XM_001068792	LOC500378	500378
8.741015	up	XM_001068868	LOC500522	500522
5.9316435	up	XM_001067281	LOC500654	500654
2.1214905	up	XM_001071951	LOC500881	500881
6.822727	up	NM_001025054	LOC500956	500956
22.007404	up	NM_001033072	LOC500959	500959
2.7673066	up	XM_576513	LOC501098	501098
5.983327	up	NM_001024361	LOC501110	501110
10.819745	up	XM_001055782	LOC501441	501441
11.347064	up	XM_577408	LOC501979	501979
2.3562593	up	XM_001069156	LOC502145	502145
5.99631	up	XM_001054404	LOC502627	502627
2.0263197	up	XM_001075598	LOC502859	502859
16.142515	up	NM_001044273	LOC503165	503165
2.6391232	up	XM_001077756	LOC503192	503192
3.5784314	up	XM_001069951	LOC503338	503338
32.77042	up	NM_001031627	LOC606294	606294
4.241918	up	NM_001034959	LOC619574	619574
18.434431	up	NM_022271	LOC64038	64038
18.952204	up	NM_001037369	LOC641520	641520
12.342932	up	NM_001037658	LOC652955	652955
2.4835277	up	NM_001037659	LOC652956	652956
3.1249316	up	XM_001053134	LOC678760	678760
5.219443	up	XM_001053095	LOC678769	678769
5.2230134	up	XM_001053100	LOC678772	678772
42.180264	up	XM_001053139	LOC678880	678880
16.006252	up	XM_001053741	LOC678902	678902
16.128618	up	XM_001054369	LOC679038	679038
4.0304303	up	XM_001054517	LOC679060	679060
3.2649121	up	XM_001054678	LOC679094	679094
9.156765	up	XM_001054754	LOC679114	679114
20.050951	up	XM_001054805	LOC679127	679127
8.414015	up	XM_001054866	LOC679140	679140
4.255931	up	XM_001054897	LOC679148	679148
2.8739111	up	XM_001054492	LOC679159	679159
15.562994	up	XM_001054731	LOC679203	679203
12.659015	up	XM_001054908	LOC679221	679221
2.2165105	up	XM_001055079	LOC679229	679229
2.550526	up	XM_001054178	LOC679330	679330
41.966137	up	XM_001056482	LOC679462	679462

6.967891	up	XM_001056631	LOC679498	679498
8.597648	up	XM_001053597	LOC679586	679586
7.661029	up	XM_001053626	LOC679594	679594
2.0763044	up	XM_001053736	LOC679612	679612
2.5855324	up	XM_001053770	LOC679623	679623
5.845734	up	XM_001054118	LOC679683	679683
2.9448383	up	XM_001054040	LOC679690	679690
81.70215	up	XM_001053017	LOC679739	679739
2.0363698	up	XM_001054331	LOC679753	679753
2.7859151	up	XM_001053720	LOC679778	679778
2.73171	up	XM_001053320	LOC679794	679794
2.4785533	up	XM_001054550	LOC679811	679811
18.2348	up	XM_001054789	LOC679823	679823
2.5381775	up	XM_001054883	LOC679899	679899
4.401772	up	XM_001054952	LOC679920	679920
5.6923847	up	XM_001054981	LOC679923	679923
9.935066	up	XM_001054993	LOC679930	679930
12.232918	up	XM_001055004	LOC679934	679934
6.6865053	up	XM_001056973	LOC679960	679960
7.5890117	up	XM_001055131	LOC679973	679973
3.2164273	up	XM_001057026	LOC679976	679976
3.9011683	up	XM_001055214	LOC680027	680027
3.7464588	up	XM_001055430	LOC680036	680036
2.0742495	up	XM_001054238	LOC680039	680039
7.291093	up	XM_001056153	LOC680040	680040
6.172698	up	XM_001055512	LOC680058	680058
7.959536	up	XM_001055808	LOC680121	680121
7.2380514	up	XM_001056161	LOC680217	680217
7.2196803	up	XM_001056242	LOC680233	680233
5.684924	up	XM_001056630	LOC680319	680319
8.348333	up	XM_001056856	LOC680363	680363
10.955039	up	XM_001057084	LOC680416	680416
2.4354844	up	XM_001057727	LOC680562	680562
2.6416366	up	XM_001058222	LOC680579	680579
17.830795	up	XM_001058343	LOC680815	680815
3.5485497	up	XM_001056401	LOC680835	680835
4.6236234	up	XM_001059333	LOC680889	680889
5.6871796	up	XM_001059658	LOC680960	680960
3.3371322	up	XM_001057248	LOC681004	681004
4.247395	up	XM_001060456	LOC681140	681140
2.0331175	up	XM_001060542	LOC681153	681153
5.4484563	up	XM_001058530	LOC681191	681191
18.730764	up	XM_001060794	LOC681219	681219
3.8062794	up	XM_001060821	LOC681222	681222

5.355145	up	XM_001060830	LOC681224	681224
7.4688497	up	XM_001060988	LOC681264	681264
8.63226	up	XM_001061130	LOC681300	681300
2.8442025	up	XM_001060981	LOC681314	681314
2.4593287	up	XM_001059415	LOC681338	681338
10.287882	up	XM_001061370	LOC681351	681351
14.0496	up	XM_001056725	LOC681423	681423
3.2574456	up	XM_001056855	LOC681449	681449
3.228323	up	XM_001056922	LOC681460	681460
6.2225256	up	XM_001056975	LOC681468	681468
15.491234	up	XM_001057588	LOC681578	681578
2.8726196	up	XM_001057704	LOC681634	681634
2.1154394	up	XM_001057182	LOC681647	681647
2.1089442	up	XM_001057798	LOC681663	681663
5.6532655	up	XM_001058015	LOC681699	681699
3.5040362	up	XM_001056690	LOC681740	681740
11.189037	up	XM_001058263	LOC681754	681754
2.6275387	up	XM_001058464	LOC681796	681796
12.895707	up	XM_001058594	LOC681825	681825
3.2874165	up	XM_001058694	LOC681849	681849
8.387172	up	XM_001058730	LOC681858	681858
2.9645908	up	XM_001058969	LOC681918	681918
4.0262055	up	XM_001061826	LOC681989	681989
131.90854	up	XM_001062110	LOC681996	681996
12.597899	up	XM_001060664	LOC682248	682248
10.744618	up	XM_001060932	LOC682306	682306
2.7575371	up	XM_001061367	LOC682404	682404
2.0589921	up	XM_001057922	LOC682408	682408
2.0064702	up	XM_001061636	LOC682469	682469
2.8806367	up	XM_001062710	LOC682699	682699
3.690057	up	XM_001062288	LOC682787	682787
2.7861435	up	XM_001059878	LOC682812	682812
4.4490895	up	XM_001063527	LOC682880	682880
8.263284	up	XM_001063647	LOC682908	682908
5.0274916	up	XM_001064066	LOC682999	682999
19.152	up	XM_001064186	LOC683007	683007
2.001826	up	XM_001064337	LOC683071	683071
3.3640058	up	XM_001058047	LOC683077	683077
3.9575791	up	XM_001064575	LOC683125	683125
17.311718	up	XM_001065596	LOC683356	683356
2.1163619	up	XM_001065821	LOC683414	683414
10.043441	up	XM_001065874	LOC683420	683420
2.676438	up	XM_001065905	LOC683429	683429
2.0311308	up	XM_001065985	LOC683447	683447

6.729275	up	XM_001066012	LOC683456	683456
3.015204	up	NM_001080907	LOC683504	683504
4.3549633	up	XM_001066288	LOC683512	683512
10.870117	up	NM_001077530	LOC683519	683519
4.9709444	up	XM_001066346	LOC683522	683522
4.3608804	up	XM_001066577	LOC683573	683573
10.811334	up	XM_001066969	LOC683661	683661
8.321168	up	XM_001066976	LOC683663	683663
3.0656738	up	XM_001066994	LOC683670	683670
8.146898	up	XM_001064018	LOC683674	683674
3.5301123	up	XM_001064124	LOC683722	683722
5.172568	up	XM_001067309	LOC683746	683746
2.6519902	up	XM_001067973	LOC683897	683897
6.151366	up	XM_001068209	LOC683955	683955
11.594931	up	XM_001068232	LOC683961	683961
4.0854964	up	XM_001068349	LOC683983	683983
3.2116578	up	XM_001068576	LOC684024	684024
8.783205	up	XM_001068630	LOC684035	684035
3.0641665	up	XM_001068728	LOC684063	684063
28.193764	up	NM_001044283	LOC684097	684097
17.123085	up	XM_001068984	LOC684112	684112
3.9383519	up	XM_001068152	LOC684233	684233
11.125013	up	XM_001059344	LOC684270	684270
2.3658462	up	XM_001069702	LOC684279	684279
2.3880558	up	XM_001069774	LOC684297	684297
3.376214	up	XM_001069799	LOC684304	684304
162.01997	up	XM_001069861	LOC684322	684322
2.5184011	up	XM_001069884	LOC684325	684325
11.854319	up	XM_001070490	LOC684352	684352
3.463682	up	XM_001070139	LOC684383	684383
3.3762028	up	XM_001070229	LOC684402	684402
152.26576	up	XM_001072835	LOC684425	684425
8.978683	up	XM_001070638	LOC684485	684485
14.399627	up	XM_001070932	LOC684548	684548
11.033116	up	XM_001071223	LOC684609	684609
2.6834064	up	XM_001071305	LOC684626	684626
7.6252933	up	XM_001075242	LOC684755	684755
2.8705611	up	XM_001071972	LOC684797	684797
4.8586073	up	XM_001072008	LOC684806	684806
2.1577034	up	XM_001072100	LOC684830	684830
3.4157457	up	XM_001072181	LOC684849	684849
2.0071228	up	XM_001062397	LOC684903	684903
4.1332316	up	XM_001061861	LOC685003	685003
3.7019825	up	XM_001062056	LOC685045	685045

2.0019193	up	XM_001062068	LOC685050	685050
14.511786	up	XM_001062200	LOC685079	685079
10.310522	up	XM_001062327	LOC685108	685108
3.0444057	up	XM_001062766	LOC685195	685195
7.9890504	up	XM_001060654	LOC685207	685207
6.0489907	up	XM_001063085	LOC685266	685266
6.463801	up	XM_001063401	LOC685337	685337
6.5222163	up	XM_001063506	LOC685367	685367
41.551514	up	XM_001064386	LOC685575	685575
10.306993	up	XM_001064444	LOC685590	685590
5.555126	up	XM_001064469	LOC685596	685596
3.797477	up	XM_001064507	LOC685605	685605
3.3293047	up	XM_001064562	LOC685626	685626
76.00669	up	XM_001064975	LOC685718	685718
3.8750145	up	XM_001060860	LOC685778	685778
2.199958	up	XM_001062929	LOC685939	685939
2.4863586	up	XM_001065880	LOC685941	685941
2.0706334	up	XM_001066160	LOC686011	686011
2.5256665	up	XM_001066359	LOC686055	686055
3.5472527	up	XM_001066392	LOC686066	686066
5.0322595	up	XM_001066660	LOC686139	686139
9.681729	up	XM_001072758	LOC686172	686172
3.7132785	up	XM_001072846	LOC686197	686197
16.060513	up	XM_001073157	LOC686259	686259
2.274835	up	XM_001073192	LOC686268	686268
16.987745	up	XM_001073320	LOC686289	686289
2.7569356	up	XM_001073550	LOC686326	686326
10.426562	up	XM_001073919	LOC686393	686393
5.3278985	up	XM_001074019	LOC686418	686418
2.5671515	up	XM_001074132	LOC686442	686442
5.769835	up	XM_001074155	LOC686547	686547
10.228831	up	XM_001074992	LOC686617	686617
2.6424978	up	XM_001075058	LOC686634	686634
6.514047	up	XM_001075174	LOC686661	686661
5.002107	up	XM_001075602	LOC686765	686765
5.156962	up	XM_001075752	LOC686791	686791
221.76659	up	XM_001076593	LOC686979	686979
14.224777	up	XM_001076594	LOC686980	686980
4.0948715	up	XM_001078569	LOC687029	687029
8.1314945	up	XM_001076906	LOC687057	687057
3.9701774	up	XM_001077450	LOC687188	687188
32.239426	up	XM_001077650	LOC687237	687237
64.393	up	XM_001073281	LOC687295	687295
2.0025055	up	XM_001077929	LOC687308	687308

5.763305	up	XM_001075972	LOC687346	687346
7.711821	up	XM_001073681	LOC687395	687395
3.6647708	up	XM_001075341	LOC687439	687439
7.5090547	up	XM_001079167	LOC687565	687565
29.301079	up	NM_001044291	LOC687575	687575
12.489047	up	XM_001079384	LOC687610	687610
9.038615	up	XM_001079870	LOC687711	687711
8.165037	up	XM_001079878	LOC687713	687713
3.946463	up	XM_001079978	LOC687738	687738
4.4991336	up	XM_001080119	LOC687780	687780
10.488231	up	XM_001080149	LOC687788	687788
11.738886	up	XM_001080179	LOC687797	687797
2.0107222	up	XM_001080216	LOC687808	687808
2.1500762	up	XM_001081046	LOC688073	688073
2.5254695	up	XM_001081257	LOC688136	688136
2.0327358	up	XM_001081294	LOC688146	688146
11.68443	up	XM_001081379	LOC688169	688169
118.61075	up	XM_001081394	LOC688173	688173
2.1739244	up	XM_001081414	LOC688176	688176
6.077757	up	XM_001081476	LOC688199	688199
6.232457	up	XM_001081553	LOC688228	688228
36.780838	up	XM_001081743	LOC688284	688284
3.637016	up	XM_001081751	LOC688289	688289
3.8268888	up	XM_001081771	LOC688296	688296
4.6333437	up	XM_001081793	LOC688310	688310
2.7156436	up	XM_001081794	LOC688311	688311
3.458111	up	XM_001081813	LOC688318	688318
28.637037	up	XM_001081867	LOC688342	688342
8.089191	up	XM_001081873	LOC688346	688346
12.088381	up	XM_001081917	LOC688369	688369
7.087788	up	XM_001066740	LOC688393	688393
6.3358407	up	XM_001066841	LOC688415	688415
2.9942355	up	XM_001067693	LOC688631	688631
2.5112512	up	XM_001067869	LOC688680	688680
9.8492775	up	XM_001068010	LOC688708	688708
2.2965598	up	XM_001068022	LOC688712	688712
4.9712744	up	XM_001068279	LOC688784	688784
9.092134	up	XM_001068644	LOC688869	688869
2.026814	up	XM_001068750	LOC688899	688899
2.2348864	up	XM_001066146	LOC688948	688948
2.1128304	up	XM_001068983	LOC688963	688963
2.80796	up	XM_001067949	LOC688966	688966
7.4583335	up	XM_001066261	LOC688981	688981
21.500626	up	XM_001069372	LOC689064	689064

16.68174	up	XM_001069421	LOC689075	689075
2.1194413	up	XM_001059239	LOC689141	689141
31.37539	up	XM_001065008	LOC689226	689226
5.4676795	up	XM_001070212	LOC689271	689271
8.0405445	up	XM_001070462	LOC689343	689343
11.4667635	up	XM_001070681	LOC689408	689408
7.8882217	up	XM_001070485	LOC689908	689908
7.327304	up	XM_001065356	LOC689959	689959
10.5048065	up	XM_001073955	LOC690000	690000
7.404454	up	XM_001072961	LOC690022	690022
2.7651463	up	XM_001073174	LOC690080	690080
4.561479	up	XM_001073696	LOC690214	690214
2.0718362	up	XM_001074295	LOC690384	690384
2.4830968	up	XM_001074777	LOC690550	690550
2.6760216	up	XM_001075416	LOC690732	690732
3.11965	up	XM_001075556	LOC690769	690769
2.2258346	up	XM_001075806	LOC690829	690829
6.1354427	up	XM_001076513	LOC691013	691013
3.336217	up	XM_001076820	LOC691093	691093
3.9501967	up	XM_001077205	LOC691204	691204
7.317188	up	XM_001077261	LOC691222	691222
12.69611	up	XM_001077406	LOC691255	691255
2.1729956	up	XM_001075994	LOC691392	691392
2.276435	up	XM_001078336	LOC691453	691453
3.875286	up	XM_001078599	LOC691510	691510
3.6373682	up	XM_001070198	LOC691532	691532
3.36319	up	XM_001072690	LOC691675	691675
2.123285	up	XM_001079338	LOC691708	691708
2.7504764	up	XM_001079361	LOC691716	691716
2.9415483	up	XM_001073008	LOC691729	691729
26.690962	up	XM_001079573	LOC691768	691768
5.1065645	up	XM_001079616	LOC691781	691781
2.0739424	up	XM_001079898	LOC691862	691862
6.9930625	up	XM_001080361	LOC691960	691960
12.368537	up	NM_133404	Lonp1	170916
4.884077	up	NM_001033689	Lonp2	291922
4.4445186	up	XM_001066614	Lonrf1	306505
2.5355303	up	NM_017061	Lox	24914
7.103039	up	NM_001012125	Loxl1	315714
2.1577847	up	XM_001069281	Loxl2	290350
2.2421312	up	XM_001066093	Loxl3	312478
3.2999835	up	NM_053936	Lpar1	116744
3.0435636	up	NM_001045843	Lpar6	691774
6.0913396	up	NM_001012189	Lpcat3	362434

5.9256215	up	XM_001053537	Lpgat1	679692
8.336193	up	NM_001014184	Lpin3	362261
46.730644	up	NM_012598	Lpl	24539
2.1226022	up	NM_001009649	Lpxn	293783
2.7983348	up	XM_001066905	Lrba	361975
3.1205244	up	XM_001076882	Lrig1	312574
2.499358	up	XM_001065921	Lrig2	310753
2.3981016	up	XM_001056970	Lrp1	299858
6.132874	up	XM_001071349	Lrp6	312781
5.0357485	up	NM_001024354	Lrrc14	500900
6.0468	up	XM_001059957	Lrrc14b	502225
7.5676737	up	NM_001012001	Lrrc2	301033
5.5899644	up	XM_001054231	Lrrc30	301711
2.646926	up	NM_001034926	Lrrc40	310946
9.968986	up	NM_001009710	Lrrc41	362566
3.498278	up	NM_001025653	Lrrc42	298309
5.078075	up	XM_001076460	Lrrc47	362672
2.1824	up	NM_001008280	Lrrc59	287633
7.103701	up	NM_001024761	Lrrfip2	301035
14.606638	up	NM_001013421	Lsg1	288029
23.888735	up	XM_001056695	Lsm10	366468
2.3054473	up	XM_001081506	Lsm12	287731
3.903534	up	XM_001060866	Lsm2	684148
11.535998	up	XM_001067054	Lsm4	290647
2.544901	up	XM_001059585	Lsm5	306222
2.833182	up	XM_001076086	Lsm7	362829
3.850489	up	XM_001058762	Lsm8	296913
4.0844526	up	XM_001079364	Lsmd1	287429
4.419414	up	XM_001064695	Lsp1	361680
2.0943778	up	NM_031049	Lss	81681
9.340592	up	NM_001030031	Lta4h	299732
25.055073	up	NM_021587	Ltbp1	59107
6.9134364	up	XM_001077795	Ltbp4	292734
11.342156	up	NM_001014157	Ltv1	361452
4.4867578	up	NM_001024269	Luc7l	360503
13.143048	up	XM_231620	Luc7l2	312251
8.843191	up	XM_001081177	Luc7l3	360602
4.2686152	up	NM_031050	Lum	81682
2.48505	up	XM_001054995	Lvrn	502160
2.8152764	up	NM_031655	Lxn	59073
4.5678945	up	NM_020103	Ly6c	56778
24.913897	up	NM_001017467	Ly6e	362934
2.8267787	up	NM_001024279	Ly96	448830
7.3134885	up	NM_001011911	Lyar	289707

2.8965366	up	XM_001069286	Lynx1	300018
6.1290035	up	NM_013006	Lypla1	25514
5.317867	up	XM_001079111	Lyrm1	365361
2.259559	up	XM_001059473	Lysmd2	300839
2.803811	up	NM_001009698	Lysmd3	315923
3.412011	up	NM_012771	Lyz2	25211
14.887085	up	XM_001066088	Lztr1	360745
17.494823	up	XM_001055153	Macf1	362587
135.49377	up	NM_139337	Macrod1	246233
2.640028	up	XM_001055286	Mad1l1	680006
8.422546	up	NM_001009699	Mad2l1bp	316237
7.1314554	up	NM_001012106	Mad2l2	313702
18.845701	up	NM_001008319	Maea	298982
81.51627	up	NM_001014085	Maf1	315093
6.507101	up	NM_022386	Mafg	64188
15.756464	up	NM_145673	Mafk	246760
7.645879	up	NM_080479	Maged2	113947
3.4461079	up	NM_001013250	Mageh1	367767
5.2426524	up	NM_001014109	Magix	317379
2.0027678	up	XM_001081552	Magmas-ps1	287065
3.2264392	up	XM_001066689	Magoh	298385
21.165878	up	NM_053946	Magt1	116967
8.916266	up	NM_001014002	Mak16	306526
3.4597597	up	XM_001060470	Malt1	307366
13.941937	up	XM_001062587	Man1a2	295319
2.2718618	up	XM_001067370	Man1c1	362625
10.725672	up	NM_139256	Man2c1	246136
4.424013	up	NM_001031655	Manba	310864
4.2356977	up	XM_001067976	Manbal	499934
2.0280516	up	NM_080785	Manea	140808
13.594519	up	XM_001072112	Manf	315989
2.2457855	up	NM_030995	Map1a	25152
37.395454	up	NM_199500	Map1lc3a	362245
44.055588	up	NM_022867	Map1lc3b	64862
59.253002	up	NM_031643	Map2k1	170851
50.229786	up	NM_133283	Map2k2	58960
5.3960953	up	NM_001033987	Map2k5	29568
8.5378685	up	NM_053703	Map2k6	114495
2.9697354	up	NM_053887	Map3k1	116667
3.733303	up	XM_001081582	Map3k3	303604
7.2425957	up	XM_001063040	Map3k4	308106
8.222978	up	XM_001062961	Map3k7	313121
11.551122	up	NM_001012062	Map3k7ip2	308267
5.399244	up	NM_001024278	Map4	367171

12.532485	up	XM_001055154	Map4k3	170920
3.205512	up	XM_001058485	Map4k4	301363
3.6322553	up	XM_001076790	Map4k5	503027
33.0264	up	XM_001058588	Map7d1	681287
24.949413	up	NM_053842	Mapk1	116590
64.79351	up	NM_021746	Mapk12	60352
2.3273497	up	NM_031020	Mapk14	81649
2.406444	up	NM_053777	Mapk8ip1	116457
3.1921313	up	NM_001011964	Mapkap1	296648
8.360099	up	NM_178102	Mapkapk2	289014
2.200281	up	NM_001012127	Mapkapk3	315994
34.874363	up	NM_001025761	Mapkapk5	498183
4.498789	up	NM_138509	Mapre1	114764
28.552498	up	NM_001007656	Mapre3	298848
10.927006	up	NM_017212	Mapt	29477
5.7819176	up	NM_001034936	Mare	360505
8.572399	up	NM_021699	Mark2	60328
6.4640317	up	NM_130749	Mark3	170577
2.0332747	up	NM_012757	Mas1	25153
2.3534045	up	NM_172043	Masp2	64459
6.423706	up	XM_001070900	Mast2	313819
14.6132	up	NM_134351	Mat2a	171347
3.8797202	up	NM_001044282	Mat2b	683630
8.7528715	up	NM_019149	Matr3	29150
27.506544	up	NM_021588	Mb	59108
20.312233	up	XM_001055992	Mbd2	680172
22.131853	up	XM_001076468	Mbd3	362834
6.9631786	up	XM_001059437	Mbd4	680915
2.1115682	up	XM_343219	Mbd6	362892
2.7344968	up	XM_001075945	Mbip	362740
11.86491	up	XM_001062557	Mbnl1	282635
9.076644	up	NM_053569	Mbtps1	89842
2.300178	up	NM_013182	Mc5r	25726
23.867529	up	NM_023983	Mcam	78967
10.0903425	up	NM_001024785	Mcart1	313241
5.21227	up	NM_001009653	Mccc1	294972
18.661585	up	NM_001012177	Mccc2	361884
4.1796374	up	XM_001058655	Mcee	293829
2.3141809	up	NM_053951	Mcf2l	117020
7.753562	up	NM_139253	Mcfd2	246117
7.537277	up	NM_021846	Mcl1	60430
4.1021338	up	XM_001079670	Mcm3ap	294339
20.166737	up	NM_001004203	Mcm7	288532
8.236077	up	XM_001057747	Mcoln1	288371

5.395574	up	XM_001062111	Mcrs1	300222
2.8258388	up	XM_227971	Mdc1	309595
6.746657	up	XM_241623	Mdga1	309659
20.034363	up	NM_033235	Mdh1	24551
117.60622	up	NM_031151	Mdh2	81829
10.583751	up	XM_001080981	Mdm2	314856
7.3806314	up	NM_001012026	Mdm4	304798
5.557255	up	XM_001059939	Mdp1	290230
15.468399	up	NM_012600	Me1	24552
8.989706	up	XM_001060753	Me3	361602
4.571356	up	NM_001044286	Mea1	685131
2.0009687	up	XM_001065376	Mecom	294924
12.345433	up	NM_017209	Mecr	29470
2.5056648	up	XM_001081351	Med1	497991
3.252328	up	XM_001062474	Med10	290939
4.91599	up	XM_001079811	Med11	287456
9.410716	up	XM_001054608	Med12	679693
3.0121999	up	XM_001081130	Med13	303403
2.9335938	up	XM_001079998	Med13l	360817
3.8172338	up	XM_001056908	Med14	317343
3.0035028	up	NM_001013178	Med20	316209
4.578519	up	XM_001074060	Med21	312849
2.177099	up	NM_001077679	Med22	499762
11.3015585	up	NM_001034079	Med24	619436
6.2759805	up	XM_001080653	Med25	292889
3.2947414	up	XM_001076126	Med27	296612
5.313355	up	XM_001065742	Med30	299905
5.6085505	up	XM_001055672	Med6	299180
24.721994	up	NM_001014035	Mef2a	309957
20.991358	up	XM_001056692	Mef2c	499497
10.739155	up	NM_001029917	Memo1	298787
6.753216	up	NM_001008345	Mesdc2	308796
7.516636	up	NM_031517	Met	24553
56.768368	up	XM_001078398	Metap1	295500
6.4392705	up	NM_022539	Metap2	64370
6.140598	up	XM_001053790	Mett11d1	305845
13.884694	up	NM_001025019	Mettl11a	362103
3.9762273	up	XM_001070243	Mettl13	289159
3.457479	up	XM_001075415	Mettl14	295428
4.290134	up	XM_001081558	Mettl2	363687
5.3383055	up	NM_001007623	Mettl6	290564
4.102428	up	NM_001037355	Mettl7a	315306
2.5435529	up	XM_225735	Mex3c	307271
5.238544	up	NM_001007609	Mfap3	287299

16.769636	up	NM_001034124	Mfap4	287382
20.914383	up	NM_001040186	Mfge8	25277
6.207044	up	NM_138976	Mfn1	192647
37.682247	up	XM_342258	Mfsd1	361957
3.3263085	up	NM_001024908	Mfsd3	500899
38.298725	up	XM_235689	Mfsd5	315329
5.6426663	up	NM_001047910	Mfsd8	361939
2.3160527	up	NM_030861	Mgat1	81519
5.4974484	up	NM_053604	Mgat2	94273
3.0146549	up	NM_001024357	MGC105560	500941
5.879122	up	NM_001012353	MGC108823	307414
7.121605	up	NM_001025718	MGC112830	361178
3.5386071	up	NM_001024905	MGC116121	498830
2.7611377	up	NM_001037362	MGC125086	498695
3.1713493	up	NM_001077231	MGC125239	686179
2.1662855	up	NM_001009538	MGC72612	494340
27.515537	up	NM_001009543	MGC72955	494346
3.352377	up	NM_198772	MGC72974	316976
14.814352	up	NM_001004221	MGC93975	292878
98.05871	up	NM_001004204	MGC94190	288616
2.8600962	up	NM_001004272	MGC94192	360550
3.2063525	up	NM_001007746	MGC94199	362483
4.2671075	up	NM_001007751	MGC94207	362946
150.43436	up	NM_001006964	MGC94542	290631
14.581758	up	NM_131904	Mgea5	154968
7.3821025	up	NM_138502	Mgll	29254
4.835514	up	NM_012861	Mgmt	25332
64.153145	up	NM_012862	Mgp	25333
4.5770316	up	NM_001013964	Mgrn1	302938
2.111917	up	NM_134349	Mgst1	171341
9.730585	up	XM_001054384	Mgst2	295037
3.55964	up	XM_001072091	Mib1	307594
14.455734	up	XM_001074357	Mical2	365352
2.2838612	up	XM_221956	Micall2	288515
3.4662	up	NM_206950	Mid1ip1	404280
2.7310789	up	XM_001058293	Mier1	313418
4.0928907	up	NM_031051	Mif	81683
11.955647	up	NM_001014122	Mif4gd	360659
5.2799444	up	NM_001017450	Miip	298643
2.4685342	up	XM_001079459	Mink1	303259
5.0807915	up	XM_001079775	Minpp1	29688
3.2956638	up	XM_001055421	Mios	362324
10.736557	up	NM_031052	Mipep	81684
2.5555122	up	NM_001009714	Mitd1	363219

3.3848767	up	XM_001065288	Mitf	25094
19.902142	up	NM_031359	Mkln1	83536
2.3975992	up	NM_001044267	Mknk1	500526
7.3331356	up	NM_001004233	Mkrn1	296988
2.4038105	up	NM_001008314	Mkrn2	297525
3.196344	up	NM_001013983	Mlec	304543
11.1388	up	XM_001065492	Mlf1	310483
34.878593	up	XM_001062959	Mlf2	312709
2.7558398	up	NM_031053	Mlh1	81685
2.0777028	up	XM_001067729	Mll1	315606
4.0997467	up	XM_341829	Mll4	361543
3.6919506	up	XM_001059647	Mll5	311968
2.00038	up	NM_001012162	Mllt10	361285
3.2976818	up	NM_053718	Mllt3	114510
14.782301	up	NM_001034112	Mlx	360631
2.3689573	up	NM_133552	Mlxipl	171078
4.288536	up	XM_001074396	Mmaa	291939
6.3002405	up	XM_001066599	Mmachc	313520
34.060085	up	NM_001004280	Mmadhc	362134
2.5710015	up	NM_001007673	Mmd	303439
3.1370227	up	XM_001057974	Mmgt1	302864
18.858606	up	NM_153472	Mnat1	266713
2.4198165	up	NM_001033891	Mobkl1b	297387
4.822501	up	NM_133528	Mobkl3	171050
3.0866776	up	XM_001062845	Mocs1	301221
34.852802	up	NM_001007633	Mocs2	294753
2.6549203	up	XM_001059515	Mogat1	363261
4.658569	up	XM_001074544	Mon1a	315999
4.32995	up	XM_001053843	Mon2	314894
3.2445707	up	XM_001065170	Morc2	289736
6.1748304	up	XM_001054868	Morc3	304074
2.7216902	up	XM_001053814	Morc4	315914
21.072481	up	NM_001011999	Morf4l1	300891
34.571877	up	NM_001007714	Morf4l2	317413
6.8690147	up	NM_001047847	Morg1	288924
3.7537568	up	NM_001024975	Morn4	293950
2.733753	up	NM_001014107	Mospd1	317312
7.3597646	up	NM_001025629	Mospd3	288557
7.7398252	up	XM_001078776	Mpdu1	303244
22.313118	up	NM_019196	Mpdz	29365
3.7963111	up	XM_001058593	Mphosph10	293828
10.3241415	up	NM_001017375	Mphosph8	290270
2.3988872	up	NM_001085406	Mpnd	681944
3.3078754	up	XM_001061133	Mpp3	114202

14.321079	up	XM_001080868	Mpp5	314259
20.434294	up	XM_001056864	Mpp6	362359
2.3622277	up	XM_001061023	Mpp7	307035
3.2997806	up	XM_001066434	Mppe1	361344
2.149718	up	NM_198778	Mpped2	362185
6.3894157	up	NM_001034022	Mprip	116504
10.83413	up	NM_138843	Mpst	192172
4.7370753	up	NM_001098240	Mpv17l	360463
2.6297317	up	XM_001072782	Mrc1	291327
7.7245607	up	XM_001056335	Mreg	501162
13.065869	up	NM_001009264	Mrfap1	282585
7.3835225	up	NM_001010947	Mri1	288912
2.7194726	up	XM_001069850	Mrpl1	289491
6.535639	up	NM_001006973	mrpl11	293666
5.2204127	up	NM_001006985	Mrpl13	299938
13.622522	up	XM_001067192	Mrpl14	301250
32.180115	up	XM_001062230	Mrpl15	297799
25.505781	up	NM_001009647	Mrpl16	293754
6.185593	up	NM_133539	Mrpl17	171061
7.9974146	up	XM_001062578	Mrpl18	292244
2.1757221	up	NM_001029898	Mrpl19	297372
4.429488	up	NM_001034136	Mrpl2	301240
20.600471	up	XM_001058684	Mrpl20	680747
9.646676	up	XM_001067014	Mrpl21	309140
20.027834	up	XM_001074937	Mrpl22	287302
5.6690316	up	NM_022529	Mrpl23	64360
11.362665	up	XM_001081298	Mrpl27	287635
85.01543	up	XM_001072824	Mrpl3	300974
9.473324	up	XM_001057859	Mrpl30	301352
11.276664	up	NM_001006965	Mrpl34	290632
7.747844	up	XM_001063053	Mrpl35	297334
9.597979	up	XM_001061033	Mrpl36	364656
21.633768	up	NM_001004235	Mrpl37	56281
45.503616	up	NM_001009369	Mrpl38	303685
7.838695	up	XM_001071006	Mrpl4	363023
2.1205273	up	NM_001013426	Mrpl41	296551
4.735394	up	XM_001077569	Mrpl42	299743
2.0496025	up	XM_001057703	Mrpl43	309440
9.037587	up	NM_001031650	Mrpl44	301552
46.96526	up	XM_001081368	Mrpl45	287656
6.539018	up	XM_001067066	Mrpl48	293149
8.564081	up	XM_001054942	Mrpl50	362517
12.551147	up	XM_001063524	Mrpl51	297601
6.773809	up	XM_001053719	Mrpl52	361037

22.371937	up	XM_001072540	Mrpl53	362388
8.873339	up	XM_001075791	Mrpl54	299628
6.7380395	up	XM_001075243	Mrpl55	287356
8.748748	up	NM_001007696	mrpl9	310653
8.871075	up	XM_001059288	Mrps11	499185
2.1721108	up	XM_001078443	Mrps12	292758
5.2334785	up	XM_001069236	Mrps14	289143
18.639025	up	NM_001007653	Mrps15	298517
10.292562	up	XM_001078418	Mrps17	288621
2.5904818	up	NM_198756	Mrps18a	301249
38.484505	up	NM_212534	Mrps18b	294230
2.781644	up	XM_001067441	Mrps18c	289469
13.926496	up	XM_001079091	Mrps2	362094
25.9949	up	XM_001057655	Mrps21l	364906
19.208302	up	XM_001081199	Mrps23	360594
3.919223	up	NM_001025408	Mrps25	297459
8.001023	up	NM_001013206	Mrps26	362216
3.537674	up	XM_001069213	Mrps28	689025
9.852839	up	XM_001068885	Mrps30	294767
4.7317615	up	NM_001047863	Mrps33	296995
20.435484	up	XM_001078600	Mrps5	296134
15.054354	up	XM_001081692	Mrps7	113958
9.689332	up	XM_001059532	Mrps9	301371
3.471426	up	NM_001008354	Mrrf	311903
3.5121734	up	NM_024001	Mrs2	79032
3.3337312	up	XM_001071573	Mrto4	298586
2.8696713	up	XM_001075321	Ms4a4a	361734
5.205077	up	XM_001063707	Msc	312897
3.1834033	up	NM_031058	Msh2	81709
10.088982	up	XM_239335	Msl1	303514
8.448501	up	XM_001071576	Msl2	315959
4.7354503	up	NM_030863	Msn	81521
3.5834994	up	NM_053307	Msra	29447
6.4011	up	NM_001031660	Msrb2	361286
11.454922	up	NM_019151	Mstn	29152
5.402968	up	XM_001074220	Msto1	295237
5.9507265	up	NM_138826	Mt1a	24567
61.181934	up	NM_022588	Mta1	64520
4.1553354	up	XM_001077908	Mta2	361724
2.537825	up	XM_216633	Mta3	298763
19.554506	up	XM_215358	Mtch1	294313
25.591305	up	XM_001065976	Mtch2	295922
13.984008	up	NM_133398	Mtdh	170910
4.781869	up	XM_001055855	Mtf1	362591

3.9117107	up	XM_001056894	Mtf2	360905
12.597525	up	NM_001009697	Mtfmt	315763
2.150465	up	XM_001055144	Mtfr1	311403
4.875161	up	NM_022508	Mthfd1	64300
2.9532404	up	XM_001065359	Mthfd1l	361472
2.67017	up	NM_001009349	Mthfs	300886
6.07886	up	NM_001004254	Mtif2	305606
5.1644087	up	NM_001013047	Mtm1	288762
7.5693145	up	XM_001059230	Mtmr1	317296
2.3478448	up	XM_001076560	Mtmr14	312634
4.1777234	up	XM_001081167	Mtmr4	287607
13.644967	up	XM_001065057	Mtmr6	305935
3.6906571	up	NM_001005761	Mtmr9	282584
4.89456	up	XM_217208	Mto1	300852
15.365149	up	NM_019906	Mtor	56718
4.754939	up	NM_001006960	Mtp18	289745
4.604474	up	XM_001056452	Mtpap	307050
12.315067	up	NM_024374	Mtpn	79215
2.7149804	up	NM_001025723	Mtrf1l	361473
9.400668	up	NM_001039003	Mtrr	290947
2.2977183	up	XM_001077379	Mtss1l	307845
5.5333896	up	NM_001015013	Mtvr2	309170
4.997358	up	XM_001074374	Mtx1	295241
3.2828712	up	XM_001055709	Murc	313225
4.1875706	up	NM_001025645	Mus81	293678
11.897574	up	NM_031061	Musk	81725
92.477875	up	NM_181368	Mustn1	290553
41.563286	up	XM_001067239	Mut	688517
2.700352	up	XM_001063623	Muted	306868
4.279303	up	NM_031063	Mvk	81727
21.127583	up	NM_022715	Mvp	64681
6.913128	up	NM_173096	Mx1	24575
9.2201	up	NM_013160	Mxi1	25701
3.9570231	up	NM_001007002	Mxra8	313770
4.578991	up	NM_183332	Myadm	369016
6.03351	up	XM_001081809	Myadml2	303744
10.574317	up	NM_031668	Mybbp1a	60571
361.7119	up	XM_001076591	Mybpc1	362867
229.47992	up	XM_001077870	Mybpc2	292879
261.19235	up	NM_031813	Mybph	83708
2.0797853	up	NM_012603	Myc	24577
2.430682	up	NM_198130	Myd88	301059
6.847363	up	XM_001080763	Myf5	299766
74.1583	up	NM_013172	Myf6	25714

10.2199	up	XM_001053402	Myh11	24582
10.823364	up	XM_001080622	Myh14	308572
32.899208	up	XM_001077462	Myh2	691644
10.992496	up	NM_012604	Myh3	24583
409.4073	up	NM_019325	Myh4	360543
14.911905	up	NM_017240	Myh7	29557
5.525319	up	XM_001078049	Myh8	252942
9.53442	up	NM_013194	Myh9	25745
162.43965	up	NM_001077656	Myl1	56781
53.220585	up	NM_017343	Myl12b	50685
52.97088	up	NM_012606	Myl3	24585
7.6028905	up	XM_001065562	Myl6	685867
3.4523463	up	XM_001067182	Myl9	296313
16.863203	up	NM_057209	Mylk2	117558
252.91457	up	NM_012605	Mylpf	24584
2.154738	up	NM_001012178	Mynn	361924
4.906358	up	XM_239684	Myo18b	304551
12.380071	up	NM_023092	Myo1c	65261
3.2174034	up	NM_173101	Myo1e	25484
5.2548933	up	NM_030865	Myoc	81523
15.682365	up	NM_176079	Myod1	337868
191.53506	up	NM_017115	Myog	29148
23.182	up	XM_001054913	Myom1	316740
11.339727	up	XM_001074764	Myom2	306616
249.31203	up	XM_001053135	Myot	291605
34.869846	up	XM_001064588	Myoz1	498440
9.874626	up	XM_001075328	Myoz2	295426
8.011902	up	XM_001080508	Mypn	309760
4.811894	up	NM_181081	Myst2	303470
2.0505836	up	XM_001068071	N4bp1	291921
8.745368	up	NM_001005533	N4bp2l2	288416
3.0015845	up	XM_001063156	N6amt2	290279
11.51829	up	NM_001047900	Naa25	360811
3.2727892	up	NM_001010967	Naaa	497009
10.683833	up	XM_001065645	Naca	288770
3.1785164	up	XM_001058289	Nacc2	296583
2.595293	up	NM_181480	Nadsyn1	353255
4.2278733	up	NM_001012120	Naga	315165
2.4960945	up	NM_001009409	Nanp	311530
5.8434744	up	NM_053561	Nap1l1	89825
4.508503	up	NM_001012170	Nap1l4	361684
4.656543	up	XM_001063511	Napg	307382
5.0790567	up	NM_001039207	Narf	360681
3.3369005	up	NM_001034921	Nars2	293128

3.037576	up	XM_001067534	Nat13	288108
8.308142	up	XM_001054968	Nat5	362228
2.4363306	up	XM_001081673	Nat9	303669
2.3424788	up	XM_001077277	Nbeal2	316014
4.08764	up	NM_031609	Nbl1	50594
3.4939356	up	NM_138873	Nbn	85482
6.687228	up	NM_001024765	Nbr1	303554
75.28583	up	NM_031521	Ncam1	24586
2.846361	up	NM_001034000	Ncapd3	315508
5.011656	up	NM_001024877	Ncaph2	300149
6.9137354	up	NM_001014785	Ncbp1	298075
2.655748	up	XM_001074363	Ncf4	500904
6.0195074	up	XM_001066601	Nck1	300955
6.060051	up	NM_012749	Ncl	25135
27.948454	up	XM_001070473	Ncoa1	313929
3.2932847	up	NM_001034007	Ncoa4	619385
7.9252157	up	XM_001064071	Ncoa6	116464
30.982262	up	XM_001077447	Ncor1	54299
9.593741	up	XM_001074041	Ncor2	360801
2.5160458	up	XM_001065306	Ncrna00117	289864
8.719893	up	NM_174864	Ncstn	289231
12.193109	up	NM_053347	Nde1	83836
3.264119	up	NM_133320	Ndel1	170845
24.760445	up	NM_001013059	Ndfip1	291609
11.2915745	up	XM_001077197	Ndfip2	361089
4.256451	up	XM_219708	Ndnl2	309259
412.76273	up	NM_133583	Ndrg2	171114
3.4384522	up	XM_001067405	Ndrg3	296318
9.01717	up	XM_001064811	Ndst2	114002
25.128368	up	XM_343760	Ndufa1	363441
5.3091702	up	NM_182671	Ndufa10l1	316632
8.743108	up	NM_212517	Ndufa11	301123
36.676125	up	XM_001080378	Ndufa12	299739
3.5177324	up	XM_001080448	Ndufa13	314759
4.514643	up	XM_001077432	Ndufa6	315167
3.5212128	up	XM_001079223	Ndufa7	299643
5.2837205	up	XM_215814	Ndufaf1	296086
4.818416	up	XM_001073799	Ndufaf2	361894
30.216166	up	NM_001033971	Ndufaf3	56769
74.97265	up	XM_001054860	Ndufb11	299310
3.0563135	up	XM_001070173	Ndufb2	362344
4.677583	up	XM_001068986	Ndufb3	301427
2.4938185	up	NM_001037338	Ndufb4	288088
45.36555	up	XM_001066565	Ndufb5	294964

23.783222	up	XM_001062275	Ndufb8	293991
65.876274	up	NM_001009290	Ndufc2	293130
86.089584	up	NM_001005550	Ndufs1	301458
65.24364	up	NM_001011907	Ndufs2	289218
44.501747	up	XM_001077876	Ndufs3	295923
2.7008214	up	NM_001025146	Ndufs4	499529
4.654201	up	NM_001030052	Ndufs5	362588
65.56391	up	NM_019223	Ndufs6	29478
28.405216	up	NM_001008525	Ndufs7	362837
15.161438	up	XM_001069669	Ndufs8	293652
16.537699	up	XM_001061260	Ndufv3-ps1	685005
677.10974	up	XM_001056398	Neb	311029
2.577945	up	XM_001080224	Nedd1	299730
266.81503	up	XM_001053672	Nedd4	25489
2.6293676	up	XM_001064485	Nedd4l	291553
84.598305	up	NM_138878	Nedd8	25490
3.4233205	up	NM_001011922	Nedd9	291044
2.0519722	up	XM_001065115	Nek3	306576
3.906327	up	XM_001060008	Nek4	306252
7.729229	up	XM_001066105	Nek7	360850
5.667497	up	XM_001057566	Nek9	299204
2.900222	up	NM_001002851	Nenf	289380
6.942471	up	XM_001074913	Neo1	81735
4.17516	up	XM_230848	Neurl2	311633
11.453672	up	XM_001079248	Neurl4	303248
26.380331	up	NM_139230	Nexn	246172
3.186713	up	XM_001067432	Nf2	25744
20.375607	up	NM_031789	Nfe2l2	83619
3.3714938	up	NM_012988	Nfia	25492
16.301085	up	XM_001059586	Nfib	29227
2.0231395	up	NM_053727	Nfil3	114519
51.60423	up	NM_030866	Nfix	81524
2.6049595	up	XM_001075876	Nfkb1	81736
6.577172	up	XM_001075778	Nfkbia	25493
31.999027	up	NM_030867	Nfkbib	81525
3.1890922	up	NM_199111	Nfkbie	316241
2.4446664	up	XM_001056399	Nfrkb	315523
26.313166	up	NM_053462	Nfs1	84594
21.311672	up	XM_001070447	Nfu1	297416
8.107326	up	XM_001059410	Nfx1	313166
7.262578	up	XM_001057531	Ngdn	305887
11.42984	up	NM_001014136	Ngly1	361014
2.079654	up	NM_001014217	Nhej1	363251
2.4396892	up	NM_199236	Nhlrc1	364682

9.906994	up	XM_001071992	Nhp2	287273
7.1180234	up	XM_001054079	Nid1	25494
4.146913	up	NM_173126	Nid67	286910
3.709716	up	NM_001024763	Nif3l1	301431
47.67306	up	NM_012867	Ninj1	25338
12.047258	up	NM_001014014	Nip30	307652
2.1304185	up	NM_138847	Nip7	192180
2.4609141	up	XM_001054428	Nipa2	308667
7.477601	up	XM_001058806	Nipal2	362899
7.1730704	up	XM_238213	Nipbl	294787
5.225697	up	XM_001057307	Nisch	306255
45.65796	up	NM_001082580	Nit1	289222
3.8284652	up	XM_001062503	Nkain1	500557
5.2631655	up	NM_001024872	Nkap	298342
9.486408	up	XM_001067083	Nmd3	310512
11.692296	up	NM_031833	Nme2	83782
15.943083	up	XM_001074672	Nme2-ps1	690520
12.751929	up	XM_001076342	Nme6	58964
10.328197	up	NM_138532	Nme7	171566
5.4752893	up	NM_001037556	Nmnat1	298653
6.414227	up	NM_001013224	Nmnat3	363118
3.3912115	up	XM_001079214	Nmral1	287063
2.2839518	up	NM_053601	Nnat	94270
3.365138	up	NM_199086	Nob1	291996
8.444229	up	NM_001033897	Noc2l	313777
3.2204442	up	XM_001080301	Noc3l	361753
2.773525	up	NM_001014129	Noc4l	360828
33.530384	up	NM_001012747	Nol12	362955
21.861576	up	NM_053516	Nol3	85383
5.0797973	up	XM_573999	Nol7	498727
14.956784	up	NM_022869	Nolc1	64896
2.082033	up	XM_001078710	Nop10	691534
14.340026	up	XM_001058941	Nop14	289724
4.792807	up	NM_001047095	Nop16	306768
10.269125	up	XM_001065060	Nop2	314969
32.699142	up	NM_001025732	Nop56	362214
4.6742806	up	NM_021754	Nop58	60373
3.7676933	up	NM_021838	Nos3	24600
2.253242	up	XM_001078434	Notch1	25496
2.9420977	up	NM_030868	Nov	81526
18.52961	up	XM_001064625	Np	290029
3.0796237	up	NM_001007800	N-pac	360477
2.0708277	up	NM_012991	Npap60	25497
2.5765572	up	XM_001058630	Npas2	316351

3.5149431	up	XM_001072137	Npat	315666
76.82881	up	NM_173118	Npc2	286898
2.1162863	up	XM_001055241	Npepl1	311671
3.093733	up	XM_001078692	Nphp1	296136
10.689682	up	NM_080577	Nploc4	140639
6.3109865	up	NM_019380	Nptn	56064
24.551434	up	NM_017000	Nqo1	24314
8.220399	up	NM_145775	Nr1d1	252917
7.1095686	up	NM_147210	Nr1d2	259241
19.888727	up	NM_031626	Nr1h2	58851
4.4170938	up	NM_031627	Nr1h3	58852
7.528135	up	NM_145780	Nr2c1	252924
2.3074286	up	NM_017323	Nr2c2	50659
3.3229208	up	NM_080778	Nr2f2	113984
30.785305	up	NM_024388	Nr4a1	79240
5.666746	up	NM_080766	Nras	24605
3.2420452	up	NM_022186	Nrbf2	58839
3.9521806	up	NM_001034997	Nrbp	619579
3.6422694	up	XM_001056969	Nrbp2	680451
70.76966	up	NM_012993	Nrd1	25499
2.2836127	up	XM_001067591	Nrip1	304157
4.268539	up	NM_145098	Nrp1	246331
2.21295	up	NM_021767	Nrxn1	60391
7.31785	up	NM_207607	Ns5atp4	311934
7.2026076	up	NM_001009399	Nsdhl	309262
4.3583546	up	NM_021748	Nsf	60355
212.61809	up	NM_031981	Nsfl1c	83809
2.9997509	up	NM_001034152	Nsg2	497878
5.7936172	up	NM_181389	Nsmaf	353233
22.660257	up	NM_001039611	Nsmce1	361645
2.6623049	up	NM_001024876	Nsmce2	299957
6.2106743	up	XM_001080533	Nsmce4a	293528
15.397447	up	XM_001057743	Nsun2	361191
4.4129596	up	XM_001070636	Nsun4	298426
2.993689	up	XM_231803	Nt5c3	312373
2.0190454	up	NM_021576	Nt5e	58813
2.5306735	up	NM_031073	Ntf3	81737
37.57083	up	NM_001011891	Nubp2	287125
7.7447567	up	NM_053463	Nucb1	84595
3.4002733	up	NM_021663	Nucb2	59295
3.6506336	up	NM_017271	Nudc	29648
3.9704452	up	NM_057120	Nudt1	117260
3.6418228	up	XM_001071036	Nudt12	367323
3.1023774	up	XM_001073187	Nudt14	299346

6.8740296	up	XM_001076872	Nudt16l1	497867
8.999288	up	XM_001071041	Nudt18	361068
3.843784	up	NM_001004258	Nudt19	308518
18.314632	up	NM_207596	Nudt2	297998
26.134003	up	NM_001039004	Nudt21	291877
102.63899	up	NM_001024243	Nudt3	294292
34.777534	up	NM_053598	Nudt4	94267
2.587882	up	NM_181363	Nudt6	207120
5.0932198	up	XM_001076428	Nudt7	361413
30.760061	up	XM_341975	Nudt8	361692
6.6163435	up	NM_001006991	Nudt9	305149
3.0131865	up	NM_001007758	Nufip1	364430
10.025452	up	XM_001068048	Numa1	308870
4.588616	up	NM_133287	Numb	29419
6.7306275	up	NM_053830	Nup107	116555
2.8949919	up	XM_001053507	Nup133	292085
8.644666	up	NM_053952	Nup155	117021
6.367608	up	XM_001065232	Nup205	362335
11.000666	up	NM_001004229	Nup35	295692
4.5089393	up	XM_001076503	Nup37	299706
5.8087187	up	NM_017361	Nup54	53372
3.7662365	up	NM_023098	Nup62	65274
8.61896	up	NM_053616	Nup88	113929
8.778071	up	NM_001011925	Nup93	291874
11.487658	up	XM_001068834	Nup98	81738
2.4396472	up	XM_001058252	Nus1	294400
43.18366	up	NM_001007629	Nutf2	291981
7.213226	up	XM_001062403	Nvl	289323
7.3212614	up	NM_021579	Nxf1	59087
2.1943402	up	XM_340857	Nxn	360577
14.013597	up	XM_001057228	Nxt1	296219
5.3518486	up	NM_001014090	Oaf	315594
2.1337447	up	NM_144752	Oas1b	246268
12.442205	up	NM_022521	Oat	64313
209.80295	up	NM_139081	Oaz1	25502
2.2456295	up	NM_001011943	Obfc1	294025
13.899736	up	NM_001034939	Obfc2b	362813
36.627556	up	XM_001076876	Obscn	338458
102.319855	up	XM_001075143	Obscnl	287353
54.914772	up	NM_001013874	Ociad1	289590
17.567495	up	NM_012615	Odc1	24609
4.301995	up	NM_017213	Odf2	29479
3.2024336	up	XM_001066370	Ofd1	302661
3.9195302	up	NM_053340	Ogfr	83525

6.754073	up	NM_001025708	Ogfrl1	316290
2.2737203	up	NM_030870	Ogg1	81528
8.782576	up	XM_001054599	Ogn	291015
19.24362	up	NM_017107	Ogt	26295
16.043894	up	NM_001033927	Ola1	296488
2.7474046	up	XM_001072555	Olfml2b	304960
6.142837	up	XM_001064747	Olfml3	310743
4.723069	up	XM_001063646	Oma1	298282
17.056467	up	NM_133585	Opa1	171116
2.7706444	up	NM_145081	Optn	246294
16.630531	up	NM_001013982	Orai1	304496
3.9821458	up	NM_001012003	Orc2l	301430
4.0738473	up	NM_001025282	Orc3l	313138
3.0662727	up	NM_001047897	Ormdl3	360618
5.8747725	up	XM_001065111	Osbpl11	303888
6.2779098	up	NM_172023	Osbpl1a	259221
4.620732	up	NM_001013079	Osbpl2	296461
2.1752884	up	XM_001065853	Osbpl5	361686
3.2640133	up	XM_001062227	Osbpl6	311129
6.3090363	up	NM_001029923	Oscp1	362595
5.780026	up	XM_001064067	Osgep	290028
5.1051893	up	NM_001024787	Osgepl1	314548
5.736968	up	XM_001056612	Osgin2	313085
5.2272034	up	NM_001005384	Osmr	310132
4.724791	up	XM_001072940	Ostalpha	303879
13.391132	up	NM_001029925	Ostm1	499474
4.079171	up	NM_207612	Ostn	360730
2.1087923	up	XM_001072480	Otub1	293705
2.048035	up	XM_001074167	Otud1	498803
5.4903793	up	NM_001037496	Otud5	363452
4.3297896	up	XM_001058882	Otud7b	310677
16.046116	up	XM_001080669	Ovca2	497954
4.607409	up	XM_001061616	Oxr1	117520
4.57328	up	XM_001062014	Oxsm	289934
3.6139753	up	XM_001078098	Oxsr1	316064
80.07015	up	NM_199102	p18	308869
6.559199	up	NM_031594	P2rx4	29659
3.2336187	up	NM_080780	P2rx5	113995
5.472145	up	NM_012800	P2ry1	25265
3.7342248	up	NM_133577	P2ry14	171108
7.2074847	up	NM_017255	P2ry2	29597
11.918135	up	NM_172062	P4ha1	64475
64.859566	up	NM_012998	P4hb	25506
3.214694	up	XM_001075237	P4htm	301008

139.97792	up	XM_001054606	Pabpc4	298510
28.912758	up	XM_001055786	Pabpn1	116697
8.954382	up	NM_130740	Pacsin2	124461
91.93929	up	NM_001009966	Pacsin3	311187
3.9636374	up	NM_001024898	Paf1	361531
14.158799	up	NM_031763	Pafah1b1	83572
7.819241	up	NM_080910	Paics	140946
14.666125	up	XM_001068994	Paip1	365684
14.353208	up	NM_001014148	Paip2	361309
18.382032	up	XM_001079699	Paip2l1	293469
2.9170506	up	NM_017198	Pak1	29431
2.8655615	up	NM_001037356	Pak1ip1	361232
7.4785576	up	NM_053306	Pak2	29432
6.235717	up	NM_001025688	Palmd	310811
44.642937	up	NM_013000	Pam	25508
3.235802	up	NM_001008862	Pan2	408200
2.499064	up	XM_001068449	Pan3	360760
7.4214926	up	XM_001081198	Pank2	296167
17.696602	up	NM_133531	Pank4	171053
2.5838377	up	NM_199397	Panx1	315435
3.9129987	up	NM_001008372	Papd4	361878
3.30368	up	XM_001066974	Papd5	307745
9.86043	up	XM_001069715	Papola	314417
4.5331545	up	XM_001057482	Papolg	305586
9.842098	up	NM_001012033	Paqr3	305203
2.4026866	up	XM_001066763	Pard3b	301455
5.9911494	up	NM_031339	Parg	83507
536.4663	up	NM_057143	Park7	117287
35.91057	up	NM_001035249	Parl	287979
4.2801304	up	NM_173114	Parm1	286894
2.8931477	up	XM_001075629	Parn	360464
11.061948	up	NM_013063	Parp1	25591
2.5031302	up	XM_001067057	Parp12	362343
2.4657016	up	XM_001071334	Parp14	303903
8.921959	up	XM_001063825	Parp2	290027
29.440325	up	NM_001008328	Parp3	300985
2.71586	up	XM_001059899	Parp4	361046
11.722443	up	XM_001073042	Parp6	300759
7.904748	up	XM_001071222	Parp9	303905
8.697353	up	NM_020656	Parva	57341
3.8256698	up	XM_001059112	Pbrm1	306254
4.7971687	up	XM_001072172	Pbx1	304947
2.3812175	up	NM_001002828	Pbx2	406164
3.8710897	up	XM_001073314	Pbxip1	310644

10.355905	up	NM_001024252	Pcaf	301164
3.2650685	up	NM_001007601	Pcbd1	29700
103.37837	up	XM_575592	Pcbp1	500242
4.605072	up	NM_001011945	Pcbp3	294336
3.924146	up	XM_001075496	Pcca	687008
4.172645	up	XM_001067802	Pcdh19	317183
3.528092	up	NM_001012215	Pcdhgb7	364844
2.2445886	up	XM_001062815	Pcf11	361605
3.4394934	up	NM_001007000	Pcgf1	312480
2.2950757	up	XM_001081335	Pcgf2	287662
9.045404	up	XM_001060303	Pcgf3	305624
6.541794	up	XM_001060464	Pcgf5	681178
3.1873512	up	XM_001070779	Pcif1	362269
53.0761	up	NM_013073	Pcmt1	25604
7.1427503	up	XM_001056609	Pcmtd2	311726
11.699868	up	NM_022381	Pcna	25737
5.6622043	up	NM_001047846	Pcnp	288165
4.8807335	up	XM_001055794	Pcnx	314288
6.8480825	up	NM_019237	Pcolce	29569
5.520949	up	XM_001068680	PCOLCE2	684050
44.765087	up	NM_001004132	Pctk1	81741
5.574146	up	NM_017225	Pctp	29510
5.911279	up	NM_145085	Pcyox1	246302
4.1330776	up	NM_053568	Pcyt2	89841
4.621034	up	NM_022595	Pdap1	64527
3.7039895	up	XM_001069234	Pdcd11	309458
6.09914	up	XM_001056283	Pdcd2	58934
6.05772	up	XM_001073061	Pdcd2l	689637
15.920541	up	NM_022265	Pdcd4	64031
10.95282	up	XM_001079809	Pdcd5	292814
9.517003	up	XM_001056202	Pdcd6	308061
79.57626	up	XM_001076624	Pdcd6ip	501083
5.265527	up	XM_001076457	Pdcd7	363082
10.738302	up	NM_001025709	Pdcl3	316348
2.3149223	up	XM_001062531	Pddc1	309110
2.2502933	up	NM_022236	Pde10a	63885
2.2183852	up	NM_001013998	Pde12	306231
2.399674	up	NM_031079	Pde2a	81743
9.947249	up	NM_017031	Pde4b	24626
7.2580953	up	NM_017032	Pde4d	24627
4.4101105	up	XM_001066038	Pde6d	363272
7.8826365	up	XM_001053781	Pde7a	81744
2.3551965	up	NM_198767	Pde8a	308776
2.811465	up	NM_031317	Pdgfc	79429

2.6953006	up	XM_001067574	Pdgfra	25267
11.683125	up	NM_001004072	Pdha1	29554
41.6373	up	NM_001007620	Pdhb	289950
9.658257	up	NM_001044242	Pdhx	311254
7.9532986	up	NM_017319	Pdia3	29468
49.40983	up	NM_001004442	Pdia6	286906
3.7934752	up	XM_001068002	Pdik1l	313609
82.39736	up	NM_030872	Pdk2	81530
47.967022	up	NM_053551	Pdk4	89813
26.654217	up	NM_017365	Pdlim1	54133
533.8848	up	NM_053650	Pdlim3	114108
23.120157	up	NM_019372	Pdp1	54705
2.9529114	up	NM_145091	Pdp2	246311
16.108614	up	NM_031081	Pdpk1	81745
3.7438853	up	NM_019358	Pdpn	54320
2.9178789	up	NM_001083624	Pds5a	305343
3.4500809	up	NM_001014249	Pdss2	365592
2.948241	up	XM_001077670	Pdxdc1	304721
4.9881697	up	XM_001059007	Pdzd11	302422
50.510708	up	XM_001075473	Pdzrn3	312607
2.6027734	up	NM_001013231	Pea15a	364052
10.18686	up	NM_017236	Pebp1	29542
19.74843	up	NM_031591	Pecam1	29583
8.171182	up	NM_001006966	Peci	291075
9.121919	up	NM_133299	Pecr	113956
22.077717	up	NM_001007634	Pelo	294754
7.5329313	up	NM_001024270	Pelp1	360552
7.229972	up	NM_001009641	Pepd	292808
3.6044786	up	NM_001044228	Pes1	289740
13.837264	up	NM_001025684	Pex11b	310682
4.417349	up	NM_053921	Pex12	116718
11.729124	up	XM_001062825	Pex13	305581
2.0595822	up	NM_172063	Pex14	64460
11.493354	up	NM_001012088	Pex16	311203
6.303892	up	NM_031350	Pex3	83519
3.082196	up	XM_001062081	Pex5	312703
2.7686176	up	NM_001034147	Pex7	308718
12.279897	up	XM_001063625	Pfdn1	361310
116.400826	up	XM_001067687	Pfdn5	300257
38.749695	up	NM_212506	Pfdn6	309629
4.8288717	up	NM_012621	Pfkfb1	24638
2.994083	up	NM_057135	Pfkfb3	117276
6.6233497	up	NM_013190	Pfkl	25741
89.04003	up	NM_031715	Pfkm	65152

2.9896598	up	NM_206847	Pfkp	60416
40.98519	up	NM_022511	Pfn1	64303
26.74488	up	NM_030873	Pfn2	81531
342.00043	up	NM_017328	Pgam2	24959
10.21292	up	NM_053895	Pgap2	116675
2.2342691	up	NM_031082	Pggt1b	81746
9.487262	up	NM_053291	Pgk1	24644
203.94774	up	NM_017033	Pgm1	24645
2.693239	up	XM_001078446	Pgm2	289632
5.072152	up	XM_001062178	Pgm2l1	685076
5.3254757	up	XM_001055264	Pgp	287115
6.5056105	up	NM_201988	Pgpep1	290648
23.46934	up	NM_001008374	Pgrmc2	361940
2.1591659	up	XM_001081747	Pgs1	303698
4.3716273	up	NM_173133	Phax	286917
13.219275	up	XM_001053030	Phb	25344
5.427076	up	NM_001013035	Phb2	114766
4.491783	up	NM_001024747	Phf10	292404
3.8700483	up	NM_001024272	Phf11	361051
2.4860957	up	XM_001080701	Phf12	303274
2.6586857	up	XM_001067059	Phf20l1	314964
12.014235	up	NM_138888	Phf5a	192246
7.6873064	up	NM_001012211	Phf7	364510
43.403107	up	NM_022626	Phka1	64561
8.972014	up	NM_001014152	Phkb	361377
23.528841	up	NM_031573	Phkg1	29353
17.22673	up	NM_080584	Phkg2	140671
31.406847	up	NM_001012206	Phlda3	363989
2.9439712	up	NM_021657	Phlpp1	59265
8.240774	up	NM_001007642	Phospho2	295663
34.848335	up	XM_001074086	Phpt1	296571
3.761135	up	XM_001065811	Phtf1	252962
93.722	up	NM_053674	Phyh	114209
6.2051263	up	NM_022301	Pi4ka	64161
5.2364616	up	NM_031083	Pi4kb	81747
19.614622	up	NM_053337	Pias2	83422
2.8257585	up	NM_031784	Pias3	83614
7.6048937	up	NM_053554	Picalm	89816
3.0622349	up	XM_001062621	Pid1	501174
2.2384505	up	XM_001078440	Pigh	362756
19.724047	up	NM_001011953	Pigk	295543
11.012995	up	XM_001055025	Pigp	288238
4.247241	up	NM_001006602	Pigs	303277
20.646599	up	XM_001070371	Pigt	296360

6.2335157	up	NM_181637	Pigu	353304
3.153062	up	NM_001010966	Pigv	366478
23.596369	up	NM_001024370	Pigy	502782
8.88274	up	XM_001064512	Pigz	689116
2.2461069	up	XM_001054196	Pik3ap1	294048
11.277901	up	NM_022958	Pik3c3	65052
5.1041617	up	NM_001017453	Pik3ip1	305472
3.3561797	up	NM_013005	Pik3r1	25513
3.5549793	up	NM_022185	Pik3r2	29741
2.0125992	up	XM_001072465	Pikfyve	316457
11.838696	up	NM_022602	Pim3	64534
4.502677	up	NM_001083337	Pinx1	305963
84.07685	up	NM_017231	Pitpna	29525
2.206652	up	NM_053742	Pitpnb	114561
5.872511	up	XM_001067446	Pitrm1	307081
2.6099467	up	NM_001042505	Pitx2	54284
2.5321176	up	NM_138896	Pja2	192256
4.8297224	up	XM_001058532	Pkd2	498328
3.8678284	up	NM_053772	Pkia	114906
4.5429907	up	NM_153469	Pkig	266709
138.45618	up	NM_053297	Pkm2	25630
5.731843	up	NM_017175	Pkn1	29355
2.3065958	up	XM_001076809	Pkn2	207122
21.700047	up	XM_001058290	Pkp4	295625
6.380023	up	NM_138882	Pla1a	85311
32.951126	up	NM_017060	Pla2g16	24913
2.4830253	up	NM_001009353	Pla2g7	301265
6.8335376	up	NM_053866	Plaa	116645
4.7973065	up	NM_013151	Plat	25692
12.397862	up	NM_013085	Plau	25619
2.3528895	up	NM_017350	Plaur	50692
13.994166	up	NM_001013927	Plbd1	297694
3.3964837	up	NM_017035	Plcd1	24655
10.771415	up	NM_080688	Plcd4	140693
2.3604615	up	NM_013187	Plcg1	25738
2.0688548	up	NM_053456	Plcl1	84587
15.203137	up	XM_001056257	Plcl2	301173
3.912268	up	NM_001012167	Pld3	361527
2.3797565	up	XM_001072894	Pld4	362792
2.3299181	up	NM_001025714	Pldn	317630
3.1063626	up	NM_001079894	Plekha1	361659
13.803106	up	NM_001013077	Plekha3	295674
2.8496442	up	XM_001060789	Plekha8	500132
3.1626978	up	XM_001055776	Plekhb2	301337

2.274787	up	XM_001072203	Plekhf2	362484
3.0340552	up	XM_234332	Plekhh1	314262
12.411405	up	XM_001071331	Plekhj1	314634
4.993805	up	XM_233611	Plekhm2	313667
5.83472	up	NM_031821	Plk2	83722
3.1585526	up	NM_053827	Plod1	116552
3.6771882	up	NM_178101	Plod3	288583
2.6853158	up	NM_207601	Plp2	302562
15.57657	up	NM_021757	Plrg1	60376
2.7890587	up	XM_001057425	Pls3	81748
8.224359	up	NM_001012139	Plscr3	360549
17.418253	up	XM_001072197	Pltp	296371
6.08019	up	XM_001054411	Plxnd1	312652
2.502479	up	XM_232864	Pm20d2	313130
7.7423944	up	NM_001008323	Pmm1	300089
7.872719	up	XM_001078730	Pmm2	302915
2.3886714	up	NM_017037	Pmp22	24660
18.786888	up	NM_001003673	Pmpca	296588
3.0527902	up	XM_001063043	Pms2	288479
3.0003042	up	NM_001008352	Pmvk	310645
3.6424356	up	NM_001004259	Pnkp	308576
6.2012386	up	XM_001079732	Pnn	368070
6.554212	up	NM_199083	Pno1	289809
3.1675518	up	XM_001062398	Pnpla2	361676
5.943418	up	XM_001059753	Pnpla7	246246
11.670127	up	XM_001078026	Pnpla8	314075
2.0950444	up	XM_001065076	Pnpt1	360992
33.051006	up	NM_173322	Pnrc1	286988
2.1083636	up	XM_001071803	Pogk	304941
2.0616486	up	XM_001078839	Pol3s	499270
7.596608	up	NM_017141	Polb	29240
103.95235	up	NM_001013050	Pold2	289758
2.284271	up	NM_001013195	Pold4	361698
2.3848329	up	XM_001080851	Poldip2	287544
10.931186	up	XM_001071977	Pole4	362385
3.7752771	up	NM_053528	Polg	85472
2.57703	up	XM_001060864	Polk	171525
2.6648748	up	NM_001014168	Poll	361767
10.220308	up	NM_031772	Polr1a	83581
2.519237	up	NM_001008330	Polr1c	301246
7.874176	up	XM_001065234	Polr1e	313245
3.359577	up	XM_001079162	Polr2a	363633
35.335804	up	XM_001075841	Polr2b	289561
65.31514	up	NM_001012473	Polr2c	361365

38.145813	up	XM_001076333	Polr2e	690966
24.82216	up	NM_031335	Polr2f	83503
7.5479894	up	NM_053948	Polr2g	117017
3.4029334	up	XM_001059014	Polr2h	498109
3.7466025	up	XM_001075773	Polr2i	292778
32.047302	up	XM_001077131	Polr2j	288588
11.495562	up	XM_577644	Polr2l-ps1	502186
2.4909608	up	XM_341388	Polr3a	361102
4.3307858	up	XM_343188	Polr3b	362858
29.966738	up	NM_001012081	Polr3c	310685
2.8700204	up	NM_001031653	Polr3d	306012
10.013226	up	XM_001077109	Polr3h	300088
80.122246	up	NM_001014259	Polr3k	366277
2.4618986	up	XM_001074109	Polrmt	299604
2.8002148	up	NM_001007747	Pomgnt1	362567
20.131426	up	XM_001060809	Pomp	288455
9.973712	up	NM_001013082	Pon2	296851
7.4797564	up	NM_001009642	Pop4	292831
4.717315	up	XM_001077549	Pop5	117241
22.138636	up	NM_199113	Popdc2	360718
6.1535316	up	NM_031576	Por	29441
2.2282064	up	XM_001055327	Postn	361945
13.612225	up	NM_172018	Pp3111	246185
33.775352	up	XM_001055932	Ppa1	294504
3.8945568	up	XM_001075518	Ppa2	310856
68.94658	up	NM_022538	Ppap2a	64369
3.4818242	up	NM_001034854	Ppapdc2	619549
17.182375	up	NM_001012349	Ppapdc3	296635
3.7054896	up	NM_031347	Ppargc1a	83516
6.749706	up	XM_001076038	Ppat	117544
2.0311246	up	NM_001039010	Ppcs	298490
4.183166	up	NM_001009316	Ppdpf	296470
5.1177063	up	XM_001061955	Ppfia4	140592
7.413819	up	XM_001074157	Ppfibp1	312855
6.650876	up	XM_001072553	Ppfibp2	308918
39.127644	up	NM_022536	Ppib	64367
4.5052943	up	NM_001004215	Ppic	291463
3.3200402	up	NM_001047868	Ppie	298508
2.6077995	up	NM_172243	Ppif	282819
27.554516	up	XM_001073803	Ppih	366461
4.927789	up	NM_175707	Ppil3	301432
2.580336	up	XM_001068646	Ppil4	361449
2.0025578	up	XM_001079075	Ppl	302934
26.28189	up	NM_033096	Ppm1b	24667

3.9306338	up	NM_147209	Ppm1g	259229
3.1027927	up	XM_001064332	Ppm1k	312381
2.3683991	up	XM_001054597	Ppox	289219
96.61765	up	NM_031527	Ppp1ca	24668
48.212643	up	NM_013065	Ppp1cb	25594
19.750624	up	NM_022498	Ppp1cc	24669
2.651402	up	NM_212542	Ppp1r11	294207
2.5640361	up	NM_053890	Ppp1r12a	116670
2.0916576	up	XM_001062402	Ppp1r12b	304813
2.3768008	up	XM_001071078	Ppp1r12c	499076
2.4124265	up	XM_001072164	Ppp1r13b	314465
6.57835	up	NM_172045	Ppp1r14b	259225
20.41623	up	NM_133425	Ppp1r14c	171010
2.95014	up	NM_133546	Ppp1r15a	171071
3.9086115	up	XM_001073136	Ppp1r16a	362944
94.44224	up	NM_022676	Ppp1r1a	58977
9.254986	up	NM_138823	Ppp1r2	192361
20.572927	up	XM_001057957	Ppp1r3a	500036
69.3471	up	NM_017039	Ppp2ca	24672
14.072926	up	NM_017040	Ppp2cb	24673
137.3717	up	NM_057140	Ppp2r1a	117281
10.586339	up	NM_053999	Ppp2r2a	117104
3.9110823	up	NM_144746	Ppp2r2d	246255
7.91984	up	XM_001059170	Ppp2r3a	363122
2.662965	up	XM_001077588	Ppp2r4	362102
7.3243036	up	XM_001066948	Ppp2r5a	312754
27.766066	up	NM_017041	Ppp3ca	24674
6.240329	up	NM_017042	Ppp3cb	24675
4.353559	up	NM_134367	Ppp3cc	171378
2.9442914	up	NM_017309	Ppp3r1	29748
8.7255125	up	NM_134359	Ppp4c	171366
4.1165977	up	NM_080907	Ppp4r1	140943
3.3919613	up	XM_001075424	Ppp4r2	297486
13.120475	up	NM_031729	Ppp5c	65179
7.2118573	up	NM_133589	Ppp6c	171121
23.003633	up	NM_001025703	Pppde2	315160
8.66615	up	XM_001079551	Pptc7	304488
2.8887763	up	XM_001056962	Ppwd1	294711
2.3680117	up	NM_001013957	Pqbp1	302557
5.0645146	up	NM_001012470	Pqcp	314329
3.8521655	up	XM_001071592	Pqlc2	362642
3.5060472	up	XM_346274	Praf2	367743
16.818573	up	XM_001068397	Prcc	310687
3.7397373	up	NM_001077648	Prdm2	313678

6.682588	up	NM_133312	Prdm4	170820
25.482138	up	NM_057114	Prdx1	117254
56.25798	up	NM_022540	Prdx3	64371
33.07708	up	NM_053610	Prdx5	113898
15.900851	up	NM_053576	Prdx6	94167
4.320375	up	NM_053385	Prelp	84400
9.817559	up	NM_031324	Prep	83471
6.2958193	up	NM_001010951	Prepl	298771
2.0379858	up	XM_230873	Prex1	311647
2.7479358	up	XM_001070215	Prg4	289104
2.318122	up	XM_001057421	Prickle1	315259
16.149763	up	NM_001014110	Prickle3	317380
4.821674	up	NM_001008768	Prim1	246327
3.6467516	up	NM_019142	Prkaa1	65248
4.9365625	up	NM_023991	Prkaa2	78975
10.57085	up	NM_031976	Prkab1	83803
6.4771266	up	NM_022627	Prkab2	64562
5.144165	up	XM_001072411	Prkaca	25636
3.275616	up	NM_001077645	Prkacb	293508
8.880979	up	NM_013010	Prkag1	25520
49.53978	up	NM_013181	Prkar1a	25725
4.144189	up	NM_019264	Prkar2a	29699
2.272639	up	NM_012713	Prkcb	25023
6.2671356	up	NM_134449	Prkcdbp	85332
2.5156715	up	NM_031085	Prkch	81749
2.4111652	up	XM_001064601	Prkci	84006
2.7583365	up	XM_001068336	Prkdc	360748
5.7296724	up	XM_001070562	Prkrip1	498171
12.254499	up	XM_001065056	Prkrir	308845
5.0113926	up	NM_024363	Prmt1	60421
10.297601	up	NM_053557	Prmt3	89820
7.3822074	up	NM_001014153	Prmt7	361402
14.614828	up	NM_012631	Prnp	24686
3.4225116	up	NM_021751	Prom1	60357
12.900869	up	XM_001061712	Prosc	306544
13.708176	up	XM_001057756	Prpf3	361995
12.249923	up	XM_001060413	Prpf38a	298374
3.430217	up	NM_001024305	Prpf38b	499691
3.5467381	up	XM_001076405	Prpf39	314171
7.6715393	up	NM_001011923	Prpf4b	291078
11.927876	up	NM_001079766	Prpf6	366276
10.844889	up	XM_001080695	Prpf8	287530
10.36203	up	NM_017243	Prps1	29562
3.0109904	up	NM_057131	Prpsap2	117272

5.4817286	up	NM_001008379	Prr13	363004
6.9477944	up	NM_001033887	Prrc1	291444
10.880803	up	XM_001079607	Prrg4	499847
9.749646	up	NM_153821	Prrx1	266813
26.318367	up	NM_001007691	Prss23	308807
2.8973467	up	XM_001069324	Prtfdc1	291355
2.2293158	up	NM_001007697	Prune	310664
100.21211	up	NM_001008764	Psenen	292788
2.6552854	up	NM_175765	Psip1	313323
2.3053474	up	XM_001076023	Pskh1	364993
75.88768	up	NM_017278	Psma1	29668
14.045235	up	NM_017279	Psma2	29669
18.979301	up	NM_001004094	Psma3l	408248
56.34624	up	NM_017281	Psma4	29671
9.64192	up	NM_017282	Psma5	29672
16.075254	up	NM_017283	Psma6	29673
185.5157	up	NM_001008217	Psma7	29674
82.95448	up	NM_053590	Psmb1	94198
2.1540024	up	NM_001025637	Psmb10	291983
22.321667	up	NM_017284	Psmb2	29675
7.6520147	up	NM_017285	Psmb3	29676
39.87153	up	NM_031629	Psmb4	58854
53.888103	up	XM_001054724	Psmb5	29425
6.687931	up	NM_057099	Psmb6	29666
49.06833	up	NM_053532	Psmb7	85492
8.586286	up	NM_080767	Psmb8	24968
21.278395	up	NM_057123	Psmc1	117263
140.62166	up	NM_033236	Psmc2	25581
168.83217	up	NM_031595	Psmc3	29677
14.575166	up	NM_057122	Psmc4	117262
43.93096	up	NM_031149	Psmc5	81827
12.849072	up	XM_001072705	Psmc6	289990
133.33554	up	NM_031978	Psmd1	83806
12.686877	up	XM_001080984	Psmd11	303353
49.577454	up	NM_001005875	Psmd12	287772
49.464893	up	XM_001058704	Psmd13	365388
38.88284	up	XM_001054967	Psmd14	311078
71.00703	up	NM_001008281	Psmd3	287670
48.47081	up	NM_031331	Psmd4	83499
11.048213	up	XM_001079109	Psmd5	296651
2.982945	up	XM_001076658	Psmd7	307821
2.5986912	up	XM_214888	Psmd8	292766
14.99341	up	NM_130430	Psmd9	161475
10.996601	up	NM_017264	Psme1	29630

9.787389	up	NM_001011894	Psme3	287716
6.884491	up	XM_001053403	Psmg1	288236
2.9626806	up	NM_001025672	Pspc1	305910
2.683137	up	XM_001070448	Pstpip1	300732
23.6895	up	XM_001056422	Pstpip2	307248
2.6144536	up	XM_001079960	Ptar1	286972
2.7706516	up	XM_001070504	Ptcd2	310025
8.804662	up	NM_001012113	Ptdss1	314553
2.4844315	up	XM_001062116	Ptdss2	293620
8.248912	up	NM_031606	Pten	50557
4.251714	up	XM_001078154	Ptges2	311865
5.211276	up	NM_138863	Ptgr1	192227
2.929715	up	XM_001060043	Ptk9l	294420
2.8708913	up	NM_031975	Ptms	83801
6.187691	up	NM_031579	Ptp4a1	29463
39.806637	up	NM_053475	Ptp4a2	85237
22.131582	up	XM_001069043	Ptp4a3	362930
15.930162	up	XM_001056514	Ptpla	680115
15.492713	up	XM_001077901	Ptpmt1	29390
3.472538	up	NM_057115	Ptpn12	117255
3.0659547	up	XM_001059696	Ptpn13	498331
2.4518924	up	XM_001066141	Ptpn14	305064
6.56035	up	NM_053990	Ptpn2	117063
2.2008424	up	NM_057204	Ptpn23	117552
2.3332973	up	XM_001055737	Ptpn3	362524
11.493107	up	NM_012763	Ptpra	25167
5.9140677	up	XM_001078855	Ptprb	314843
4.7065306	up	XM_001067936	Ptprd	313278
3.381948	up	NM_001029902	Ptprk	360302
8.813046	up	XM_001053209	Ptprm	29616
7.583453	up	NM_019140	Ptprs	25529
79.57218	up	XM_001057313	Puf60	84401
10.328787	up	XM_001063603	Pum1	362609
8.762725	up	XM_001068003	Pum2	298874
3.334863	up	XM_001063244	Pura	307498
5.4769526	up	NM_001025563	Pus1	304567
2.2287507	up	NM_001025278	Pus10	305583
3.6494756	up	XM_001057876	Pus3	315554
4.820686	up	XM_001061692	Pus7	296751
5.1433067	up	XM_001077744	Pusl1	362681
55.70644	up	NM_022499	Pvalb	25269
12.026629	up	NM_017076	PVR	25066
4.4291034	up	XM_001075998	Pwp1	362856
5.7831235	up	NM_031587	Pxmp2	29533

12.456368	up	NM_017234	Pxmp3	29534
4.825143	up	NM_001012147	Pxn	360820
2.328567	up	NM_001011993	Pycrl	300035
2.2622213	up	NM_022268	Pygl	64035
7.426434	up	NM_001007624	Qars	290868
43.2544	up	NM_022390	Qdpr	64192
5.0284023	up	XM_574316	Qk	499022
16.665504	up	XM_001069252	R3hcc1	361064
3.6101801	up	XM_001054959	R3hdm1	304763
14.103243	up	XM_001056644	R3hdm2	362894
37.86812	up	NM_031090	Rab1	81754
112.435326	up	NM_017359	Rab10	50993
19.387043	up	NM_031152	Rab11a	81830
26.239552	up	NM_032617	Rab11b	79434
133.85703	up	XM_001053530	Rab12	25530
3.751414	up	NM_031092	Rab13	81756
6.0552077	up	NM_053589	Rab14	94197
6.275916	up	NM_001012468	Rab18	307039
6.8247166	up	NM_001004238	Rab21	299799
28.427965	up	NM_001015023	Rab24	361208
4.131605	up	NM_053978	Rab28	117049
59.681698	up	NM_031718	Rab2a	65158
3.430185	up	NM_001037645	Rab2b	305853
3.6090252	up	NM_145094	Rab31	246324
2.2406912	up	NM_001012140	Rab34	360571
2.679969	up	NM_001013046	Rab35	288700
3.7400217	up	NM_013018	Rab3a	25531
9.698066	up	XM_001064454	Rab3gap2	289350
6.159319	up	NM_017313	Rab3ip	29885
7.2206106	up	XM_001081838	Rab40b	303754
2.9313014	up	NM_013019	Rab4a	25532
3.5811148	up	NM_017355	Rab4b	50866
2.2009048	up	NM_022692	Rab5a	64633
3.3205142	up	NM_001079936	Rab5b	288779
18.131107	up	XM_001081435	Rab5c	287709
5.1238585	up	XM_001062702	Rab6a	84379
40.688335	up	NM_023950	Rab7a	29448
5.1175103	up	NM_133590	Rab7l1	171122
5.6700864	up	NM_053998	Rab8a	117103
7.630401	up	NM_153317	Rab8b	266688
308.8508	up	NM_031774	Rabac1	83583
2.7937584	up	NM_019124	Rabep1	54190
2.2222292	up	XM_001069323	Rabgap1l	304914
9.705811	up	XM_001072217	Rabgef1	360797

4.5058584	up	NM_031654	Rabggta	58983
47.66696	up	NM_138708	Rabggtb	25533
5.94442	up	XM_001075907	Rabl4	300062
65.63303	up	NM_134366	Rac1	363875
3.4107347	up	NM_001024778	Rad17	310034
3.2101815	up	NM_001025701	Rad21	314949
130.35257	up	NM_001013190	Rad23a	361381
44.097664	up	NM_001025275	Rad23b	298012
3.5229812	up	XM_001057419	Rad52	297561
3.1171825	up	NM_012639	Raf1	24703
4.592635	up	NM_001001718	Rai12	287446
10.525889	up	NM_001011958	Raly	296301
9.292027	up	NM_031646	Ramp2	58966
82.98424	up	NM_053439	Ran	84509
2.5880358	up	XM_001073477	Ranbp10	361396
5.9281507	up	XM_001053839	Ranbp2	294429
27.658123	up	XM_001056647	Ranbp3	501281
2.2456594	up	NM_001012199	Rangap1	362965
5.083531	up	XM_001078960	Rangrf	287419
24.940947	up	NM_001005765	Rap1a	295347
96.33841	up	NM_134346	Rap1b	171337
2.1803322	up	XM_001069856	Rapgef2	310533
4.5715375	up	XM_001060956	Rapgef4	252857
4.1649647	up	XM_001066365	Rapsn	362161
2.2943072	up	XM_001059523	Rarb	24706
2.9970613	up	NM_001013427	Rarres2	297073
29.207443	up	XM_213276	Rars	287191
2.4948804	up	XM_001065097	Rars2	297969
5.2885094	up	NM_013135	Rasa1	25676
3.9075718	up	NM_031574	Rasa3	29372
2.3489525	up	XM_001075445	Rasl12	315762
2.7048352	up	NM_001037096	Rassf2	311437
2.370335	up	XM_001074931	Rassf8	312846
2.9551277	up	XM_001054971	Rb1cc1	312927
2.3630562	up	XM_001072631	Rbak	288489
4.449273	up	XM_001060117	Rbbp5	304794
7.245974	up	XM_001073098	Rbbp6	308968
46.80396	up	NM_031816	Rbbp7	83712
2.6324928	up	XM_001072535	Rbbp8	291787
3.7428882	up	NM_021764	Rbck1	60383
2.5736694	up	NM_031094	Rbl2	81758
4.5167584	up	NM_152861	Rbm10	64510
5.073121	up	NM_139094	Rbm16	245926
40.338524	up	NM_001013058	Rbm17	291295

2.4052505	up	XM_001062355	Rbm20	309544
9.28084	up	NM_001025676	Rbm22	307410
9.424801	up	XM_001073426	Rbm24	690139
11.924314	up	XM_001055193	Rbm25	366693
4.441683	up	XM_001069790	Rbm27	361317
2.7527447	up	XM_001063640	Rbm28	312182
22.975786	up	XM_001063211	Rbm3	114488
3.5769274	up	NM_001014015	Rbm34	307956
11.300613	up	XM_001054287	Rbm38	366262
7.5299215	up	XM_215201	Rbm4	293663
10.102599	up	NM_001014159	Rbm42	361545
3.696093	up	NM_153306	Rbm45	266631
2.1986735	up	NM_001007014	Rbm4b	474154
6.286034	up	XM_001073958	Rbm6	315997
4.383428	up	XM_001066319	Rbm7	315634
15.857212	up	XM_001059872	Rbm8a	295284
13.426871	up	NM_001079895	Rbm9	362950
5.4120364	up	NM_001025403	Rbms2	288771
3.6084325	up	XM_001061146	Rbms3	680726
7.8908396	up	XM_001071004	Rbmxrtl	307779
2.049832	up	XM_001064167	Rbpsuh	297767
4.894107	up	NM_001034135	Rbx1	300084
5.4128985	up	XM_001073857	Rc3h1	304916
4.598062	up	XM_001053333	Rc3h2	311909
19.093632	up	NM_175578	Rcan2	140666
5.967957	up	NM_199084	Rcbtb2	290363
8.811194	up	NM_001007618	Rchy1	289508
2.2878358	up	NM_001013152	Rcl1	309301
4.6277304	up	XM_001069334	Rcor3	684192
6.7565536	up	XM_001071544	Rcsd1	360872
18.174822	up	NM_212548	Rdbp	294258
3.8380523	up	NM_181478	Rdh10	353252
2.3289657	up	NM_001012193	Rdh11	362757
2.4785428	up	XM_001072505	Rdh14	500629
4.237154	up	NM_199208	Rdh2	299511
12.2703905	up	XM_001081488	Rdm1	287726
21.317673	up	NM_001005889	Rdx	315655
4.172528	up	XM_001081701	Recql5	287834
104.1151	up	XM_344661	Reep5	364838
6.728121	up	NM_199267	Rela	309165
2.865087	up	XM_224732	Rent1	306350
2.4251025	up	XM_001060578	Reps1	292944
44.18231	up	NM_001039012	Rer1	298675
4.083968	up	XM_001057878	Rev1	316344

4.4552345	up	NM_001083966	Rev3l	309812
3.189305	up	XM_001061328	Rexo1	314630
15.215358	up	NM_001033884	Rexo4	311826
4.454775	up	XM_001078203	Rfc1	89809
7.631768	up	NM_053786	Rfc2	116468
2.3809743	up	NM_021849	Rfng	60433
2.0188124	up	XM_001063006	Rft1	290552
2.2295234	up	XM_001062448	Rftn1	501095
3.8216698	up	XM_001075875	Rfwd3	361409
2.0770168	up	NM_001012172	Rfx3	361746
3.2245874	up	XM_001053787	Rfx7	315804
2.2061808	up	XM_576547	Rfx8	501127
3.94871	up	NM_001013136	Rfxank	306353
3.4083376	up	NM_001008337	Rg9mtd1	304012
2.2418275	up	NM_001013090	Rg9mtd3	298081
10.6140175	up	XM_001072931	Rgc32	117183
33.29319	up	NM_001004225	RGD1303003	294326
5.9907417	up	NM_001004219	RGD1303117	292764
8.351325	up	NM_001004244	RGD1303127	300206
9.010332	up	NM_001004226	RGD1303130	295231
23.187279	up	NM_201560	RGD1303142	296346
5.290158	up	NM_001009711	RGD1304567	362671
4.508604	up	XM_001076933	RGD1304587	303180
4.008017	up	XM_001073101	RGD1304592	362461
2.4999263	up	XM_001073554	RGD1304610	305831
13.18962	up	NM_001047874	RGD1304704	302247
2.1438465	up	XM_001067282	RGD1304963	313891
6.030055	up	XM_001068395	RGD1304982	289138
7.682884	up	NM_001013976	RGD1305007	303749
13.394687	up	XM_001074486	RGD1305045	296050
3.5263374	up	XM_001057128	RGD1305110	305579
6.8267064	up	XM_001065324	RGD1305158	297971
14.284513	up	NM_001025003	RGD1305178	311855
3.1450415	up	XM_001062898	RGD1305202	311550
5.273228	up	NM_001014013	RGD1305215	307643
5.7831864	up	NM_001017447	RGD1305235	292267
19.196585	up	XM_001074867	RGD1305350	313699
2.0103102	up	NM_001025652	RGD1305420	298072
2.4896636	up	XM_001057069	RGD1305455	288545
22.489637	up	NM_001047856	RGD1305481	294030
13.81938	up	NM_001013901	RGD1305592	293500
12.259883	up	XM_001065112	RGD1305593	288513
2.7180827	up	NM_001008297	RGD1305689	290529
2.1344063	up	XM_001067947	RGD1305793	309456

4.3064804	up	XM_001053257	RGD1305823	360461
7.7668815	up	XM_001078385	RGD1306001	362975
4.316327	up	XM_341506	RGD1306058	361224
8.255075	up	XM_001078821	RGD1306074	296601
93.36796	up	XM_001061185	RGD1306148	313196
2.2913222	up	XM_001055529	RGD1306192	361443
5.0603385	up	XM_001073540	RGD1306215	296565
3.0704603	up	XM_001080392	RGD1306228	362748
3.919499	up	NM_001008283	RGD1306284	287918
2.2604425	up	XM_001065721	RGD1306371	314061
28.366234	up	NM_001014126	RGD1306410	360768
59.31198	up	NM_001009635	RGD1306437	290303
3.0545816	up	NM_001013908	RGD1306583	294709
5.490138	up	NM_001025626	RGD1306595	287554
14.87473	up	XM_001081367	RGD1306682	360617
2.6432123	up	XM_001072521	RGD1306717	311257
5.4941916	up	NM_001039608	RGD1306730	309009
10.96275	up	XM_001080360	RGD1306739	361834
5.374336	up	XM_001069792	RGD1306746	312511
3.9839756	up	NM_001044252	RGD1306809	365699
2.6424785	up	XM_001054788	RGD1306820	309069
4.222864	up	XM_001081151	RGD1306862	287596
3.2492154	up	XM_342099	RGD1306917	361805
6.7864122	up	XM_001055149	RGD1306936	297082
12.918065	up	NM_001008288	RGD1306954	288269
8.6725025	up	XM_001073151	RGD1306959	361624
4.437718	up	XM_001055616	RGD1306962	309570
7.952691	up	XM_001077056	RGD1307067	362840
2.2262394	up	XM_001055449	RGD1307071	311502
8.861228	up	NM_001037184	RGD1307100	294978
2.0919971	up	NM_001037188	RGD1307155	302998
2.5504441	up	XM_001072759	RGD1307158	361773
3.2909148	up	NM_001025669	RGD1307161	305031
8.952056	up	XM_001080615	RGD1307222	303300
6.1111445	up	NM_001024875	RGD1307254	298712
5.451677	up	NM_001008343	RGD1307279	307210
2.5407474	up	XM_001060869	RGD1307392	299209
15.698167	up	XM_001081752	RGD1307394	360667
2.0365098	up	XM_001059692	RGD1307396	360757
32.0485	up	NM_001048043	RGD1307399	681315
3.0733073	up	NM_001009655	RGD1307465	296200
8.362713	up	XM_001059656	RGD1307525	308053
11.398735	up	XM_001072428	RGD1307554	292739
2.478727	up	XM_001056938	RGD1307597	299197

5.2608542	up	XM_001077827	RGD1307615	362084
2.165942	up	XM_001080064	RGD1307621	314168
3.7845273	up	NM_001013905	RGD1307648	294004
19.458841	up	NM_001024760	RGD1307682	300675
6.112302	up	XM_001057487	RGD1307704	314300
72.38223	up	NM_001013922	RGD1307752	296315
17.840126	up	NM_001017454	RGD1307799	307833
9.9876585	up	NM_001037192	RGD1307890	304851
2.1500733	up	XM_219870	RGD1307934	293953
3.6294303	up	XM_001074087	RGD1308026	363029
2.4764907	up	XM_001068778	RGD1308048	298557
5.6351085	up	XM_001075554	RGD1308093	361034
3.4878907	up	NM_001014248	RGD1308127	365493
2.9002843	up	XM_001080268	RGD1308139	360563
13.29294	up	NM_001014010	RGD1308147	307008
7.578096	up	XM_001055826	RGD1308165	362516
5.23835	up	XM_001076521	RGD1308251	362676
2.921678	up	XM_001061204	RGD1308396	290235
7.2136264	up	XM_001054512	RGD1308430	361038
2.1870255	up	NM_001014198	RGD1308470	362778
5.0905657	up	NM_001037181	RGD1308517	290722
4.631485	up	XM_001055958	RGD1308601	307249
28.815535	up	XM_214237	RGD1308772	290381
2.1209416	up	NM_001025677	RGD1308782	308318
7.665945	up	XM_001076708	RGD1308923	362678
3.903757	up	XM_001059736	RGD1308958	298020
3.3057508	up	NM_001013944	RGD1309051	299153
3.4781964	up	XM_001080751	RGD1309077	360569
6.941489	up	XM_001072347	RGD1309085	297821
2.345774	up	XM_001060014	RGD1309095	301381
6.236811	up	XM_001053125	RGD1309102	291750
10.315539	up	XM_001070787	RGD1309104	289084
3.199852	up	XM_001067663	RGD1309148	298147
4.0092545	up	NM_001017451	RGD1309228	298851
4.00276	up	XM_001061009	RGD1309308	313115
3.3919122	up	NM_001024884	RGD1309362	307415
3.490954	up	NM_001014246	RGD1309482	365458
10.727316	up	NM_001013918	RGD1309540	295930
9.809525	up	NM_001014140	RGD1309676	361118
5.5471706	up	NM_001014131	RGD1309708	360867
88.10014	up	XM_001072116	RGD1309710	293700
5.1985645	up	XM_001071109	RGD1309730	295952
18.446491	up	XM_001078661	RGD1309748	302913
9.304702	up	XM_001059108	RGD1309759	361448

11.377058	up	XM_001059837	RGD1309765	290746
2.422695	up	XM_001075851	RGD1309779	363074
9.59744	up	XM_001067111	RGD1309823	313525
2.8924487	up	XM_001080214	RGD1309829	296190
6.6333923	up	NM_001014243	RGD1309888	365215
3.0286398	up	NM_001009246	RGD1309906	287406
2.9997141	up	XM_001069514	RGD1309922	306007
3.8870044	up	XM_001076152	RGD1309995	314690
2.049064	up	XM_001079614	RGD1310016	309306
8.599079	up	NM_001039015	RGD1310230	301563
2.220318	up	NM_001014197	RGD1310311	362746
21.631466	up	XM_001059237	RGD1310313	298308
4.005594	up	XM_001063969	RGD1310348	360738
12.751267	up	XM_001064076	RGD1310351	298425
2.077996	up	XM_001073512	RGD1310352	303122
3.0371523	up	XM_001077296	RGD1310358	308537
5.2799635	up	XM_001071619	RGD1310427	362643
15.708614	up	XM_001058484	RGD1310552	300836
15.081357	up	XM_001065132	RGD1310587	360894
29.187237	up	NM_001008360	RGD1310686	360480
5.5058894	up	XM_001057961	RGD1310769	299207
3.800973	up	XM_001064739	RGD1310862	303016
6.687478	up	XM_001081737	RGD1311078	360664
4.644914	up	XM_001064550	RGD1311164	297607
2.6550765	up	NM_001014173	RGD1311265	361976
2.4564447	up	NM_001025719	RGD1311307	361238
28.233162	up	XM_235517	RGD1311310	315166
9.520416	up	XM_001067219	RGD1311345	361201
3.5854282	up	XM_001081569	RGD1311429	360642
27.637352	up	XM_213210	RGD1311433	287054
5.9148536	up	NM_001009678	RGD1311458	303948
3.6904633	up	NM_001008318	RGD1311578	298748
3.6717825	up	XM_001079649	RGD1311595	309307
3.218366	up	XM_001068870	RGD1311605	298841
2.8086905	up	XM_001067917	RGD1311634	293155
3.5125067	up	XM_001071724	RGD1311648	313949
10.7961645	up	XM_001074468	RGD1311660	288518
2.838093	up	NM_001013898	RGD1311703	293160
2.7217846	up	NM_001008329	RGD1311745	301079
3.6279085	up	XM_001065046	RGD1311756	362769
6.086711	up	NM_001009638	RGD1311805	291784
5.2645626	up	XM_001053590	RGD1311849	313346
10.814407	up	NM_001024974	RGD1311863	293871
10.530288	up	NM_001009630	RGD1311899	288704

3.6133637	up	XM_001057895	RGD1311910	307235
3.3985476	up	NM_001025683	RGD1311946	309145
2.4756124	up	NM_001007702	RGD1359108	313155
2.760386	up	NM_001007657	RGD1359127	299612
41.160313	up	NM_001007659	RGD1359310	300240
10.35214	up	NM_001007676	RGD1359380	303922
17.71095	up	NM_001014193	RGD1359529	362626
11.063678	up	NM_001007663	RGD1359616	300782
4.4802375	up	XM_213514	RGD1559482	498022
5.72023	up	XM_001080525	RGD1559545	503029
17.131346	up	XM_001056249	RGD1559610	313581
3.9353552	up	XM_001080284	RGD1559639	366656
3.6415713	up	XM_001077671	RGD1559667	497919
8.975693	up	XM_001075526	RGD1559682	361080
4.3451867	up	XM_574157	RGD1559715	498871
9.146895	up	XM_001074399	RGD1559750	295406
2.1374724	up	XM_001080437	RGD1559781	294376
6.3817563	up	XM_001056621	RGD1559794	503168
6.8029094	up	XM_001065275	RGD1559808	316158
2.307356	up	XM_001070266	RGD1559838	502591
4.52135	up	XM_001079026	RGD1559845	308494
2.743353	up	XM_001072458	RGD1559849	314468
2.7060628	up	XM_575487	RGD1559871	500135
15.836868	up	XM_001056490	RGD1559909	362592
8.497099	up	XM_001058240	RGD1559917	287993
5.40974	up	XM_001063047	RGD1559923	498489
3.8110213	up	XM_001081019	RGD1559940	503115
9.048006	up	XM_001071140	RGD1559955	367324
3.1818433	up	XM_001081070	RGD1559961	497974
2.0265515	up	XM_001057888	RGD1559972	363817
11.131097	up	XM_001081687	RGD1559979	503216
3.131261	up	XM_001065433	RGD1560000	365889
54.850613	up	XM_001073577	RGD1560011	315732
29.489895	up	XM_001062160	RGD1560015	499018
4.5924973	up	XM_213187	RGD1560124	302497
2.645887	up	XM_223076	RGD1560186	289384
3.6593146	up	XM_001059137	RGD1560263	499670
3.1296842	up	XM_001066043	RGD1560277	308763
9.960719	up	XM_001072503	RGD1560286	500575
44.73123	up	XM_001056272	RGD1560341	498869
2.6039307	up	XM_001061644	RGD1560433	296750
8.257157	up	XM_001061418	RGD1560451	366074
4.2708282	up	XM_001064366	RGD1560501	364239
4.156962	up	XM_001078107	RGD1560568	500914

2.1607087	up	XM_241817	RGD1560601	317432
6.824121	up	XM_001057549	RGD1560648	364583
2.689871	up	XM_001078366	RGD1560687	500804
4.433617	up	XM_001055827	RGD1560729	366411
3.4081337	up	XM_001062341	RGD1560755	361165
6.620023	up	XM_001074726	RGD1560778	316001
6.3291974	up	XM_001053952	RGD1560796	315798
7.097416	up	XM_001058159	RGD1560813	498894
2.3459964	up	XM_001058994	RGD1560821	367865
6.3094597	up	XM_001057158	RGD1560831	499803
2.017235	up	XM_574398	RGD1560854	499106
6.2818265	up	XM_001058121	RGD1560873	306238
6.9037294	up	XM_001061556	RGD1560888	498140
6.1800265	up	XM_001058746	RGD1560909	363221
9.330605	up	XM_001063031	RGD1560917	501028
2.2226176	up	XM_001079730	RGD1560936	293472
2.9076576	up	XM_001055728	RGD1561042	498386
7.7973766	up	XM_001070733	RGD1561055	501644
2.6498241	up	XM_001059526	RGD1561067	498642
3.7348557	up	XM_001069296	RGD1561134	307332
8.837554	up	XM_001059732	RGD1561137	366030
2.0242286	up	XM_226771	RGD1561161	294747
8.827897	up	XM_343494	RGD1561176	363155
8.324015	up	XM_574742	RGD1561179	499423
2.0426855	up	XM_001065499	RGD1561195	298126
7.756594	up	XM_001069254	RGD1561238	502091
131.54529	up	XM_001061778	RGD1561333	499374
2.1694489	up	XM_001073485	RGD1561381	498340
4.774525	up	XM_001059755	RGD1561394	311743
2.3605208	up	XM_001059953	RGD1561425	301378
6.718452	up	XM_001061954	RGD1561440	363942
3.6562004	up	XM_001078588	RGD1561453	309408
7.3583417	up	XM_001064409	RGD1561459	361606
8.370127	up	XM_001058999	RGD1561537	361197
12.279909	up	XM_228802	RGD1561594	317394
23.525745	up	XM_001064224	RGD1561618	367265
2.7269306	up	XM_001054273	RGD1561628	306428
5.4651647	up	XM_001053157	RGD1561671	361214
5.7964096	up	XM_001079196	RGD1561676	307917
29.114454	up	XM_001061583	RGD1561736	499794
4.9707637	up	XM_001070434	RGD1561792	298861
2.696597	up	XM_578478	RGD1561839	502972
3.61318	up	XM_001075153	RGD1561908	364036
2.2301204	up	XM_001060744	RGD1561963	301556

6.098606	up	XM_001073022	RGD1561993	296377
3.4877627	up	XM_001080895	RGD1562012	497962
2.457078	up	XM_001070383	RGD1562020	290820
2.1235628	up	XM_001077854	RGD1562055	287029
4.3905344	up	XM_579917	RGD1562079	499125
2.9433846	up	XM_573072	RGD1562125	497887
14.121373	up	XM_001058517	RGD1562165	366374
3.987605	up	XM_001056900	RGD1562178	500039
11.493984	up	XM_001070030	RGD1562259	500451
3.691261	up	XM_001081219	RGD1562272	303433
10.963677	up	XM_576917	RGD1562289	501516
2.2453387	up	XM_001057462	RGD1562291	301676
2.210479	up	XM_001080948	RGD1562299	299169
77.78609	up	XM_575338	RGD1562323	499984
2.1306708	up	NM_001040019	RGD1562373	501072
3.0352378	up	XM_001072344	RGD1562378	500351
2.5169683	up	XM_001080819	RGD1562381	314248
3.9154708	up	XM_001059720	RGD1562397	364060
4.2445827	up	XM_001064690	RGD1562399	301438
3.1748426	up	XM_001079874	RGD1562402	499133
2.0592597	up	XM_001058023	RGD1562404	314556
9.131399	up	XM_001064372	RGD1562407	307029
2.875098	up	XM_001055546	RGD1562461	503251
3.5605755	up	XM_575924	RGD1562484	500560
3.1353495	up	XM_001073075	RGD1562500	498096
3.5069165	up	XM_001053847	RGD1562502	363485
2.1560802	up	XM_001080393	RGD1562547	299740
2.0059237	up	XM_001062602	RGD1562552	498840
2.189722	up	XM_001067020	RGD1562553	501803
2.6852705	up	XM_001074693	RGD1562558	290019
148.43475	up	XM_001056645	RGD1562690	500965
37.037262	up	XM_001075489	RGD1562716	500254
5.5654435	up	XM_001057627	RGD1562717	363767
3.9702692	up	XM_001065962	RGD1562725	500559
9.956581	up	XM_001054261	RGD1562747	292328
2.9817228	up	XM_001067272	RGD1562758	498881
4.4275956	up	XM_001063505	RGD1562796	289715
18.392431	up	XM_001072232	RGD1562952	365661
13.544135	up	XM_001054334	RGD1562987	498886
2.462391	up	XM_001073245	RGD1562997	291773
2.9083006	up	XM_001069221	RGD1563091	500011
4.1918273	up	XM_001068217	RGD1563097	364613
4.2005467	up	XM_001066541	RGD1563099	292151
4.961103	up	XM_001078525	RGD1563106	303238

2.2752538	up	XM_001079410	RGD1563124	500817
2.279347	up	XM_001068099	RGD1563145	499937
6.07923	up	XM_001074453	RGD1563157	298910
18.829775	up	XM_001059413	RGD1563216	500694
13.779703	up	XM_001063662	RGD1563319	293632
2.6622117	up	XM_001058849	RGD1563412	368112
5.7123027	up	NM_001077643	RGD1563422	289182
13.378772	up	XM_001079603	RGD1563438	287442
17.740736	up	XM_001072081	RGD1563463	293699
3.3418949	up	XM_001079076	RGD1563482	498179
6.0462265	up	XM_001078729	RGD1563508	499250
2.0262861	up	XM_001067946	RGD1563524	302797
4.457662	up	XM_001077912	RGD1563570	498360
3.4137661	up	XM_001064539	RGD1563581	502110
62.594837	up	XM_001064421	RGD1563601	498731
5.920839	up	XM_001077328	RGD1563613	366605
2.1209576	up	XM_001068593	RGD1563636	292466
14.963414	up	XM_001068508	RGD1563658	366058
3.458878	up	XM_001075700	RGD1563705	501058
2.13503	up	XM_001060668	RGD1563812	498293
33.154804	up	XM_001055755	RGD1563861	498828
2.456576	up	XM_001068357	RGD1563888	360692
3.8194423	up	XM_001058897	RGD1563956	303815
5.195804	up	XM_001056192	RGD1563958	315521
2.341234	up	XM_346307	RGD1563991	367797
16.256464	up	XM_001053149	RGD1564051	501206
2.6661708	up	XM_001059661	RGD1564055	364059
8.532221	up	XM_001065641	RGD1564058	288925
2.006519	up	XM_001070796	RGD1564093	288920
3.50262	up	XM_001067726	RGD1564131	500871
3.2637146	up	XM_001079061	RGD1564148	287415
3.1848123	up	XM_574980	RGD1564171	499656
6.0771484	up	XM_345912	RGD1564209	367046
7.0390425	up	XM_001067032	RGD1564319	366089
3.265728	up	XM_001074520	RGD1564379	499758
7.8100204	up	XM_001058193	RGD1564400	295660
3.7786167	up	XM_001081206	RGD1564425	296169
28.127771	up	XM_001068311	RGD1564469	295340
6.538709	up	XM_001071990	RGD1564492	498914
2.4303806	up	XM_001069119	RGD1564583	310761
3.949595	up	XM_001064912	RGD1564698	497882
2.1762543	up	XM_001064201	RGD1564839	289401
5.978725	up	XM_001080930	RGD1564883	312363
5.4477105	up	XM_001060995	RGD1564899	498579

2.9350474	up	XM_575773	RGD1564940	500411
2.7372847	up	XM_001060653	RGD1564943	500551
12.41362	up	XM_001059335	RGD1564963	503169
3.6542275	up	XM_001062086	RGD1564964	315843
2.2061546	up	XM_001080634	RGD1565002	299135
3.1231596	up	XM_001079107	RGD1565010	314660
7.5079184	up	XM_001055900	RGD1565023	305671
5.0075793	up	XM_001081584	RGD1565033	498014
42.395084	up	XM_001066800	RGD1565054	299041
5.659435	up	XM_001072499	RGD1565073	502913
4.9095907	up	XM_001057297	RGD1565095	503252
2.89653	up	XM_223417	RGD1565119	305350
5.0514436	up	XM_001071597	RGD1565131	498143
2.1733065	up	XM_001076997	RGD1565145	367181
6.6478167	up	XM_001077968	RGD1565149	307923
5.8435206	up	XM_001057767	RGD1565183	293846
6.709072	up	XM_577347	RGD1565192	501923
2.0859847	up	XM_001071740	RGD1565210	499072
6.2462325	up	XM_001059889	RGD1565215	498998
3.0703588	up	XM_001054203	RGD1565222	498514
8.716358	up	XM_344408	RGD1565258	364385
2.6717741	up	XM_001057346	RGD1565310	362697
7.001726	up	XM_001080915	RGD1565317	363650
2.418868	up	XM_001065101	RGD1565368	500983
37.050865	up	XM_001063205	RGD1565371	501140
15.495816	up	XM_575764	RGD1565372	366307
3.061757	up	XM_576453	RGD1565403	501041
22.393206	up	XM_001078166	RGD1565456	314589
2.3837974	up	XM_001081626	RGD1565459	287782
2.2188919	up	XM_001079014	RGD1565486	497865
2.4877176	up	XM_001076292	RGD1565496	300783
2.1644375	up	XM_001072329	RGD1565545	308337
125.80369	up	XM_001060296	RGD1565566	499057
4.721446	up	XM_001056445	RGD1565579	367860
2.3122666	up	XM_001077692	RGD1565599	502599
2.6385565	up	XM_001053477	RGD1565641	499567
2.9916892	up	XM_001059638	RGD1565744	360494
3.5174396	up	XM_001059981	RGD1565767	311120
5.335695	up	XM_001070697	RGD1565775	361980
2.0636666	up	XM_001076633	RGD1565787	308429
16.626474	up	XM_001060204	RGD1565798	500923
2.8193698	up	XM_001063774	RGD1565829	317202
6.216225	up	XM_001053108	RGD1565900	300833
2.8887458	up	XM_001056823	RGD1565927	304020

2.2038035	up	XM_573334	RGD1565928	498123
2.6757402	up	XM_001080341	RGD1565947	299737
2.9112165	up	XM_001059689	RGD1565965	302513
2.8375921	up	XM_001064472	RGD1565996	288136
3.2148597	up	XM_001081529	RGD1566002	303576
5.8781815	up	XM_001064345	RGD1566035	363438
2.2837675	up	XM_001065417	RGD1566036	498119
5.484369	up	XM_001079792	RGD1566052	687694
2.8441105	up	XM_001053559	RGD1566078	363514
8.296065	up	XM_001056883	RGD1566136	300278
3.3919716	up	XM_001071067	RGD1566137	301299
5.497376	up	XM_235089	RGD1566161	314782
7.098976	up	XM_001077872	RGD1566189	499244
13.8045435	up	XM_574837	RGD1566212	499512
14.707372	up	XM_001065495	RGD1566215	301742
17.53158	up	XM_001067908	RGD1566239	306348
2.5028307	up	XM_001059999	RGD1566265	363487
2.0765667	up	XM_001065290	RGD1566319	502988
3.1861064	up	XM_001054551	RGD1566320	296207
2.6004896	up	XM_001056402	RGD1566355	315362
2.7811496	up	XM_001074530	RGD1566369	291353
7.211941	up	XM_574994	RGD1566380	365871
3.3792496	up	NM_139040	RGD621098	207123
8.977221	up	NM_199493	RGD735029	307480
74.55105	up	NM_199379	RGD735065	294311
6.5186734	up	NM_199112	RGD735175	316530
2.3568177	up	XM_001070966	Rgl1	289080
4.3661346	up	NM_212547	Rgl2	294283
2.988994	up	XM_001079315	Rgs10	54290
4.3707504	up	NM_021661	Rgs19	59293
23.30863	up	NM_019341	Rgs5	54294
10.739623	up	NM_001024891	Rhbdd1	316557
3.4860387	up	XM_001077358	Rhbdd2	360793
2.479398	up	NM_001013875	Rhbdd3	289753
28.68318	up	NM_013216	Rheb	26954
16.969366	up	NM_057132	Rhoa	117273
12.625474	up	NM_022542	Rhob	64373
4.598167	up	XM_001074130	Rhobtb1	309722
5.6276245	up	XM_001068816	Rhoc	295342
22.718542	up	NM_001037195	Rhog	308875
14.886008	up	XM_001080778	Rhoj	299145
7.5645227	up	NM_053522	Rhoq	85428
14.454698	up	NM_181823	Rhot2	287156
6.1730103	up	NM_001007715	Ribc1	317431

4.220694	up	XM_001058411	Ric8a	293614
2.788127	up	XM_001065608	Rif1	295602
2.3598204	up	XM_001080699	Rilp	287531
18.447798	up	XM_001079000	Rilpl1	304469
5.1181293	up	NM_001004205	Rilpl2	288652
2.6840363	up	XM_001054910	Rin2	311494
5.539192	up	NM_212549	Ring1	309626
35.625282	up	XM_001072670	Riok3	361293
2.238199	up	XM_001065998	Ripk1	306886
8.824067	up	XM_001056736	Ripk2	362491
10.896959	up	XM_001054292	Rlf	313566
2.0912824	up	NM_001024892	Rlim	317241
4.330817	up	XM_001058471	Rmnd1	292268
4.474398	up	XM_232051	Rmnd5a	312439
2.341074	up	NM_001017473	Rmnd5b	497900
3.1621335	up	NM_001012231	Rnase13	497194
4.3133364	up	NM_020082	Rnase4	56759
10.860072	up	NM_001013234	Rnaseh2a	364974
9.824584	up	NM_001007007	Rnaseh2b	361056
13.119738	up	XM_001079783	Rnasek	287453
16.771334	up	XM_001058381	Rnasen	310159
4.143807	up	XM_001070006	Rnaset2	292306
2.6250753	up	NM_001013222	Rnd1	362993
95.37726	up	NM_001011904	Rnf10	288710
6.387433	up	NM_053438	Rnf103	84508
7.831431	up	XM_001055544	Rnf111	300813
3.1034348	up	NM_001014791	Rnf113a1	313450
31.465937	up	NM_001001517	Rnf114	362277
7.21216	up	NM_001033702	Rnf126	314613
2.2813933	up	NM_001012010	Rnf135	303350
20.567978	up	XM_235338	Rnf139	315000
20.946247	up	XM_001066493	Rnf14	619577
3.9133465	up	XM_001067906	Rnf145	287212
14.722157	up	NM_001012060	Rnf146	308051
5.313687	up	XM_001055727	Rnf160	288308
6.0824676	up	NM_001002279	Rnf166	365022
5.6113653	up	NM_001024271	Rnf185	360967
31.366732	up	XM_233761	Rnf19b	313806
6.7761984	up	NM_001025667	Rnf2	304850
2.1566975	up	XM_001053479	Rnf20	313216
31.081566	up	XM_001057827	Rnf217	292188
10.842248	up	XM_001059460	Rnf31	364386
4.4897914	up	NM_001004075	Rnf34	282845
4.0548954	up	NM_019182	Rnf4	29274

5.3745437	up	NM_001012195	Rnf41	362814
5.1626406	up	XM_001070453	Rnf6	304271
7.006333	up	XM_001065291	Rnf7	300948
3.1877105	up	NM_001025727	Rnf8	361815
16.020775	up	XM_001081135	Rnft1	360595
2.4383357	up	XM_001057191	Rngtt	313131
20.406353	up	NM_139105	Rnh1	245964
3.8504813	up	NM_001008299	Rnmt	291534
6.1294937	up	NM_031097	Rnpep	81761
31.295122	up	XM_001070050	Robld3	295234
14.989892	up	XM_001066397	Robld3-ps1	686067
2.009169	up	NM_031098	Rock1	81762
8.310779	up	NM_013022	Rock2	25537
12.877656	up	NM_001024864	Rogdi	287061
5.1330204	up	XM_001053332	Romo1	679572
2.0851872	up	XM_001056400	Rora	300807
2.0576153	up	XM_001058675	Rorc	368158
7.0086308	up	NM_021582	Rpa2	59102
5.4206295	up	NM_001033060	Rpain	287463
6.478651	up	NM_001033999	Rpap1	311338
5.8368263	up	NM_001004243	Rpap3	300189
5.917083	up	NM_001033903	Rpe	501157
6.774653	up	XM_001062107	Rpia	362383
158.64456	up	NM_031100	Rpl10	81764
4.0870266	up	NM_031065	Rpl10a	81729
13.361131	up	NM_001025739	Rpl11	362631
4.7426586	up	XM_001059627	Rpl12	296644
4.3249865	up	NM_173340	Rpl13a	317646
4.4625	up	NM_022949	Rpl14	65043
5.1445765	up	NM_139114	Rpl15	245981
5.235643	up	NM_031102	Rpl18	81766
11.92239	up	NM_212510	Rpl18a	290641
2.1289122	up	NM_031103	Rpl19	81767
4.2035537	up	NM_031104	Rpl22	81768
3.5358727	up	XM_001064104	Rpl22l1	361923
3.0358908	up	XM_001057847	Rpl22l2	680575
7.3618035	up	NM_001007599	Rpl23	29282
2.5923455	up	XM_001059934	Rpl23a	360572
24.991165	up	XM_001060312	Rpl26l1	307636
20.192501	up	XM_001068745	Rpl26-ps1	685704
2.538263	up	NM_022514	Rpl27	64306
8.886663	up	XM_001077420	Rpl27a	293418
6.7075934	up	NM_022697	Rpl28	64638
47.595016	up	NM_198753	Rpl3	300079

4.0423784	up	NM_022699	Rpl30	64640
2.1254587	up	NM_022506	Rpl31	64298
7.6933	up	NM_013226	Rpl32	28298
2.7457495	up	NM_212511	Rpl35	296709
3.287145	up	NM_031106	Rpl37	81770
6.172988	up	XM_001055547	Rpl37a	363248
3.3523781	up	XM_001058739	Rpl37a-ps1	500547
8.374948	up	XM_001066244	Rpl37a-ps2	686028
3.5601988	up	XM_001053404	Rpl37-ps1	679570
2.4475656	up	NM_001077592	Rpl38	689284
6.4732466	up	NM_012875	Rpl39	25347
13.20305	up	XM_001058430	Rpl3l	287122
46.76025	up	NM_022510	Rpl4	64302
7.5257516	up	NM_139083	Rpl41	124440
20.489128	up	NM_031099	Rpl5	81763
5.1601143	up	NM_053971	Rpl6	117042
9.425128	up	XM_001068131	Rpl6-ps1	500714
3.4309642	up	XM_001062960	Rpl7	297755
9.059033	up	XM_001065509	Rpl7l1	317275
157.17615	up	NM_001034916	Rpl8	26962
78.822945	up	NM_022402	Rplp0	64205
11.645636	up	NM_001007604	Rplp1	140661
4.3136516	up	NM_001030021	Rplp2	140662
85.30404	up	NM_013067	Rpn1	25596
11.09377	up	NM_031698	Rpn2	64701
13.115519	up	XM_001060859	Rpp14	361020
3.4002464	up	NM_001002831	Rpp21	406230
3.2644017	up	NM_001033063	Rpp38	291317
6.569852	up	NM_001013055	Rpp40	291071
2.778801	up	XM_001056597	Rprd1a	291736
2.3316305	up	XM_001067861	Rprd1b	311591
3.2370534	up	NM_031109	Rps10	81773
112.47945	up	NM_031110	Rps11	81774
2.8078647	up	NM_031709	Rps12	65139
5.619554	up	NM_130432	Rps13	161477
9.87385	up	NM_022672	Rps14	29284
7.765059	up	NM_017151	Rps15	29285
5.1878486	up	NM_053982	Rps15a	117053
3.381123	up	NM_017152	Rps17	29286
52.406322	up	NM_213557	Rps18	294282
2.009713	up	NM_001037346	Rps19	29287
2.9387653	up	XM_001076866	Rps19bp1	500907
4.4160004	up	NM_031838	Rps2	83789
4.311083	up	NM_001007603	Rps20	122772

5.0058203	up	NM_031111	Rps21	81775
18.596693	up	NM_078617	Rps23	124323
11.381827	up	NM_031112	Rps24	81776
14.651924	up	NM_001005528	Rps25	122799
11.115071	up	NM_013224	Rps26	27139
28.606462	up	NM_053597	Rps27	94266
3.1562588	up	NM_031113	Rps27a	81777
33.674442	up	XM_001056747	Rps27l	681429
3.2100701	up	XM_001060434	Rps28-ps1	50718
4.751335	up	NM_012876	Rps29	25348
6.9568458	up	NM_001009239	Rps3	140654
18.394327	up	NM_017153	Rps3a	29288
55.13319	up	NM_001007600	Rps4x	29426
25.918827	up	XM_001062647	Rps5	25538
4.0704694	up	NM_017160	Rps6	29304
2.0277715	up	NM_031107	Rps6ka1	81771
5.732904	up	XM_001054840	Rps6ka3	501560
16.43919	up	NM_031985	Rps6kb1	83840
13.104889	up	NM_001010962	Rps6kb2	361696
8.266759	up	NM_031570	Rps7	29258
3.844906	up	NM_031706	Rps8	65136
12.129392	up	NM_031108	Rps9	81772
36.803932	up	NM_017138	Rpsa	29236
4.724613	up	XM_001078722	Rpusd3	362416
2.7393184	up	NM_001009357	Rqcd1	301513
16.192564	up	NM_053338	Rrad	83521
2.6137373	up	NM_001048184	Rragc	298514
4.6878166	up	XM_001063809	Rragd	297960
21.775745	up	XM_001080661	Rras	361568
6.6742115	up	NM_001013434	Rras2	365355
5.4237995	up	XM_001053542	Rrbp1	311483
5.2489777	up	XM_001064405	Rrm1	685579
15.514588	up	NM_001013236	Rrm1-ps1	365320
14.158387	up	XM_001053401	Rrn3	304714
7.616296	up	NM_001012073	Rrp1	309674
2.3279107	up	XM_001071108	Rrp1b	309673
2.2403467	up	NM_001008346	Rrp8	308911
2.1459703	up	XM_001071554	Rrp9	363134
2.0989091	up	NM_001079699	Rrs1	297784
2.8051586	up	NM_138881	Rsad2	65190
3.5470428	up	XM_001063020	Rsbn1l	311987
2.1673002	up	XM_001061808	Rshl2	361476
7.1658077	up	NM_001008876	Rsl1d1	302898
7.4675045	up	NM_001014212	Rsl24d1	363099

4.586198	up	XM_001059176	Rspo3	498997
20.344637	up	NM_001014128	Rsrc2	360807
32.73724	up	XM_001057090	Rsu1	680419
2.7541187	up	NM_001008831	RT1-Ba	309621
3.3684976	up	NM_001004084	RT1-Bb	309622
9.949883	up	NM_001008839	RT1-CE16	414819
8.671019	up	NM_001008847	RT1-Da	294269
15.50243	up	NM_001008884	RT1-Db1	294270
7.589453	up	NM_198741	RT1-DMa	294274
4.3448462	up	NM_198740	RT1-DMb	294273
2.659176	up	NM_012646	RT1-N1	24748
4.6023164	up	NM_001008854	RT1-N2	360323
29.927383	up	NM_001008886	RT1-S3	294228
2.5682268	up	NM_001008858	RT1-T24-1	361787
17.408562	up	NM_001008826	RT1-T24-4	414784
19.623634	up	NM_001004227	Rtcd1	295395
5.1090956	up	XM_001057789	Rtel1	362288
2.2089944	up	NM_201562	Rtn2	308410
10.164662	up	NM_001009953	Rtn3	140945
4.3075805	up	XM_001062754	Rtn4ip1	309912
11.116464	up	XM_001056345	Rtp4	360733
8.97464	up	XM_001071405	Rufy1	360521
2.004985	up	XM_001072800	Rufy2	690777
2.15944	up	XM_001081465	Rundc1	303552
3.596194	up	NM_147177	Ruvbl1	65137
18.540808	up	NM_001025405	Ruvbl2	292907
7.4375863	up	NM_147146	Rwdd1	259218
2.5945716	up	XM_001070573	Rwdd2b	304132
4.61409	up	NM_031765	Rxrg	83574
3.3210762	up	NM_080402	Ryk	140585
341.29175	up	XM_001078539	Ryr1l	114207
3.5411859	up	NM_001007636	S100a1	295214
2.3600981	up	NM_031114	S100a10	81778
2.4428158	up	XM_215607	S100a13	295213
17.873999	up	XM_001075704	S100a16	361991
3.4367626	up	NM_012618	S100a4	24615
3.340571	up	NM_053798	Sacm1l	116482
6.648634	up	XM_001063390	Sacs	305940
11.126279	up	NM_001012063	Sae1	308384
21.310642	up	NM_022394	Safb	64196
18.805332	up	NM_001012040	Samd8	305684
5.25031	up	XM_001069386	Samd9l	500015
20.03582	up	NM_001004241	Samm50	300111
4.5241046	up	XM_001075034	Sap30l	360531

14.235388	up	XM_001071295	Saps1	361502
2.740529	up	XM_001054912	Saps2	300146
3.5426176	up	XM_001067905	Saps3	309144
34.97911	up	NM_001007739	Sar1a	361842
77.62087	up	NM_001009622	Sar1b	287276
104.564896	up	NM_001007606	Sars	266975
4.553297	up	XM_001078462	Sars2	292759
24.367435	up	NM_031596	Sart1	29678
4.4933734	up	XM_001080462	Sart3	304582
43.57728	up	NM_001007667	Sat1	302642
8.046428	up	XM_001079461	Sat2	360547
11.070946	up	NM_001012129	Satb1	316164
20.88713	up	NM_001097581	Sav1	299116
67.46099	up	NM_001008289	Sbds	288615
4.216917	up	XM_001054780	Sbf1	300147
6.241142	up	XM_001074548	Sbno1	304470
3.985643	up	XM_001073102	Sbno2	314619
6.0616755	up	NM_053642	Sc5dl	114100
2.1804943	up	NM_021581	Sc65	59101
16.194706	up	XM_342174	Scamp1	29521
9.527726	up	NM_023955	Scamp2	65168
31.033075	up	XM_001074344	Scamp3	65169
10.731121	up	XM_001077128	Scap	301024
3.3621497	up	NM_031541	Scarb1	25073
3.7724607	up	XM_001057528	Scarf1	303313
11.479213	up	NM_001013985	Sccpdh	305021
2.752488	up	NM_001017499	Scfd2	498353
18.735817	up	XM_001061765	Schip1	295105
2.288605	up	XM_001053591	Scmh1	362581
270.8901	up	NM_017288	Scn1b	29686
6.764826	up	XM_001055032	Scnm1	310662
3.255067	up	XM_001078805	Sco1	497930
7.045967	up	NM_138508	Scp2	25541
6.5641847	up	NM_133383	Scpep1	114861
3.8900657	up	XM_001074724	Scrib	362938
8.26007	up	NM_001013162	Scrn3	311731
2.675826	up	XM_001077553	Scube2	499241
39.658386	up	XM_001060414	Scx	680712
3.279479	up	NM_001011938	Scyl1	293684
4.8076386	up	XM_001076749	Scyl2	314717
22.577604	up	NM_013082	Sdc2	25615
9.397431	up	NM_012649	Sdc4	24771
15.350042	up	NM_031986	Sdcbp	83841
2.6773343	up	NM_001013199	Sdccag10	361887

5.1326895	up	XM_001077607	Sdccag1-ps1	299114
31.340036	up	NM_001013135	Sdccag3	306322
2.0987966	up	NM_177929	Sdccag8	305002
5.219821	up	XM_001080807	Sdf2	287470
27.797516	up	NM_130412	Sdf4	155173
190.34729	up	NM_130428	Sdha	157074
8.896291	up	NM_001008371	Sdhaf2	361726
49.991814	up	NM_001005534	Sdhc	289217
80.81287	up	NM_198788	Sdhd	363061
8.742937	up	XM_001061937	Sdr39u1	361044
2.2749531	up	XM_001080769	Sec1	308586
35.356026	up	NM_031723	Sec11a	65166
68.69557	up	NM_001006978	Sec13	297522
12.263093	up	XM_001081756	Sec14l1	360668
2.0526931	up	NM_053571	Sec16b	89868
3.514691	up	NM_057147	Sec22a	117513
53.51553	up	NM_001025686	Sec22b	310710
17.68143	up	XM_001076053	Sec23a	58817
4.9251056	up	XM_001054580	Sec23b	362226
3.144633	up	XM_001080274	Sec23ip	309010
6.708977	up	XM_001075268	Sec24b	295461
4.052852	up	XM_001075384	Sec24d	310843
30.578138	up	NM_033021	Sec31a	93646
2.3424625	up	NM_001024770	Sec3l1	305287
6.6187625	up	XM_341558	Sec61a2	361273
2.5353317	up	XM_001056613	Sec61b	298068
2.8558748	up	NM_001034129	Sec62	294912
4.5225453	up	NM_024002	Secisbp2	79049
13.680724	up	XM_001077702	Secisbp2l	296115
9.655346	up	NM_023027	Secp43	65241
2.8797722	up	NM_199082	Sectm1b	287884
11.023137	up	NM_177933	Sel1l	314352
13.400753	up	NM_207589	Selk	290549
7.391009	up	NM_001085485	Selo	315216
3.8241944	up	NM_173120	Sels	286900
13.441906	up	NM_001014253	Selt	365802
4.078125	up	NM_001079942	Sema3b	363142
9.463491	up	XM_001064163	Sema3c	296787
2.2821941	up	XM_001077367	Sema6d	311384
3.6426246	up	NM_023989	Senp2	78973
19.227617	up	XM_001078584	Senp3	303245
6.6187935	up	XM_001061212	Senp6	300860
2.8701303	up	XM_001057745	Senp7	288167
3.9533145	up	NM_001079889	Sephs2	308993

18.551388	up	NM_013027	Sepw1	25545
35.06209	up	NM_001044285	Sepx1	685059
2.4130604	up	XM_574308	Serac1	499015
16.843605	up	NM_145086	Serbp1	246303
7.494993	up	XM_001080802	Sergef	365243
14.52121	up	NM_182951	Serinc1	294421
38.907227	up	NM_001031656	Serinc2	313057
9.881882	up	NM_138825	Serpina12	191570
2.3496234	up	NM_001031642	Serpinb1a	291091
7.7451177	up	XM_001060394	Serpine2	29366
14.817408	up	NM_177927	Serpinf1	287526
24.479012	up	NM_199093	Serping1	295703
5.42685	up	NM_001024903	Sertad2	498423
19.319017	up	XM_001063428	Sesn1	294518
8.649124	up	XM_001066119	Sestd1	295678
4.833836	up	XM_001075829	Setd2	316013
22.345997	up	XM_001069129	Setd3	299295
2.5646973	up	XM_001070821	Setd4	245975
2.4159915	up	XM_001056961	Setd6	291844
2.4585598	up	XM_573394	Setd8-ps1	498178
7.8668275	up	NM_058210	Sf1	117855
8.767079	up	NM_001025698	Sf3a3	313583
19.27864	up	XM_001066744	Sf3b1	84486
41.18455	up	XM_001073034	Sf3b2	293671
10.409397	up	XM_001077410	Sf3b3	292019
2.5781698	up	NM_001011951	Sf3b4	295270
12.381041	up	XM_001059359	Sf3b5	680891
4.9917784	up	NM_001011920	Sf4	290666
6.734101	up	NM_031647	Sfmbt1	58967
2.0493314	up	NM_053544	Sfrp4	89803
17.356853	up	XM_001072402	Sfrs1	689890
6.612111	up	NM_001035255	Sfrs11	502603
9.172322	up	NM_020092	Sfrs12	56763
17.496964	up	XM_001055451	Sfrs18	297942
3.0394742	up	NM_001009720	Sfrs2	494445
13.5902195	up	XM_001064077	Sfrs4	362612
28.664425	up	NM_001014185	Sfrs6	362264
3.0820758	up	NM_001039035	Sfrs7	362687
5.826501	up	NM_001034924	Sfrs8	304431
12.793834	up	NM_001008302	Sft2d1	292305
2.552496	up	XM_001073930	Sfxn4	361778
4.977682	up	NM_153298	Sfxn5	261737
40.94429	up	XM_001081302	Sgca	303468
4.5510116	up	XM_001056227	Sgcb	680229

49.69303	up	XM_001069547	Sgcd	497892
212.0373	up	NM_001006993	Sgcg	305941
4.7604146	up	NM_019232	Sgk1	29517
25.389349	up	NM_181386	Sgms1	353229
3.4079874	up	XM_001080791	Sgpp1	81536
10.40082	up	NM_022703	Sgta	64667
3.0464892	up	NM_053669	Sh2b2	114203
98.20129	up	XM_001053494	Sh3bgr	498066
4.1868453	up	XM_001067638	Sh3bgrl3	298544
159.7302	up	NM_001011929	Sh3glb1	292156
2.8248124	up	NM_001009692	Sh3glb2	311848
4.143611	up	NM_053360	Sh3kbp1	84357
10.296311	up	NM_001034187	Sh3rf2	307472
2.2782624	up	XM_001057749	Sh3tc1	305441
3.088929	up	NM_031153	Sharpin	81859
22.48104	up	NM_001077826	Shisa4	360848
27.210037	up	NM_001006989	Shisa5	301013
5.5064373	up	NM_001047842	Shmt1	287379
11.051339	up	NM_001008322	Shmt2	299857
5.8524485	up	NM_001013155	Shoc2	309548
2.8007581	up	XM_001069964	Shprh	308282
10.25865	up	NM_080905	Siah1a	140941
2.436474	up	NM_001015036	Siat7F	407765
2.5404098	up	XM_001068825	Sidt2	315617
4.5940595	up	NM_001012182	Sike	362007
3.1811566	up	NM_199376	Sil1	291673
8.206169	up	XM_343395	Sin3a	363067
12.217725	up	XM_001062859	Sin3b	683381
2.0355723	up	NM_001004089	Sipa1	361710
3.7793236	up	NM_139330	Sipa1l1	246212
2.4776409	up	NM_001009704	Sipa1l2	361442
3.3485687	up	XM_001080493	Sirt1	309757
22.83627	up	NM_001008368	Sirt2	361532
6.3276267	up	XM_001058755	Sirt3	293615
7.140189	up	NM_001004256	Sirt5	306840
3.1532516	up	NM_001031649	Sirt6	299638
2.0998604	up	XM_001072396	Siva1	362791
18.505146	up	XM_001080688	Six1	114634
2.8509724	up	XM_001059855	Six2	366542
4.680904	up	NM_213559	Skiv2l	294260
9.904751	up	NM_001007608	Skp1	287280
16.916681	up	XM_001076917	Slain2	305310
5.5480676	up	NM_001024368	Slc10a3	501665
5.9473386	up	NM_013173	Slc11a2	25715

28.828806	up	NM_031798	Slc12a2	83629
12.767719	up	NM_019229	Slc12a4	29501
2.1423159	up	XM_001066528	Slc12a6	691209
2.5794578	up	NM_001009713	Slc17a5	363103
3.354403	up	NM_031148	Slc20a1	81826
7.06197	up	NM_177421	Slc22a17	305886
3.8272278	up	XM_001065778	Slc22a23	64559
9.228933	up	NM_022270	Slc22a4	64037
3.4296114	up	NM_019269	Slc22a5	29726
78.59457	up	NM_022398	Slc25a11	64201
5.112881	up	XM_342445	Slc25a12	362145
2.1495433	up	NM_001047880	Slc25a15	306574
4.596383	up	XM_001076461	Slc25a17	300083
5.018639	up	NM_001007674	Slc25a19	303676
23.26765	up	NM_053965	Slc25a20	117035
2.106371	up	XM_001068401	Slc25a28	688811
28.788841	up	NM_139100	Slc25a3	245959
5.5126014	up	XM_001061863	Slc25a32	315023
7.079645	up	NM_053515	Slc25a4	85333
2.5049949	up	XM_001069918	Slc25a44	365841
16.655571	up	XM_001059020	Slc25a46	291709
2.713241	up	XM_001081843	Slc26a11	360670
9.971856	up	NM_031664	Slc28a2	60423
86.87886	up	NM_031684	Slc29a1	63997
38.78038	up	NM_012751	Slc2a4	25139
3.855146	up	NM_001083122	Slc30a2	25362
3.225537	up	NM_172066	Slc30a4	64469
7.76119	up	XM_001062806	Slc30a5	294698
3.9031754	up	XM_001065683	Slc30a6	298786
13.428535	up	XM_001057251	Slc30a9	498358
5.6206985	up	NM_133600	Slc31a1	171135
5.8178816	up	NM_001033693	Slc31a2	298091
2.2175252	up	NM_147140	Slc35a4	257647
67.9987	up	NM_199081	Slc35b1	287642
6.2882433	up	NM_001037215	Slc35b2	501103
2.479444	up	XM_001071455	Slc35c2	311637
2.2302089	up	XM_001080983	Slc35e3	362883
2.0248976	up	NM_153316	Slc35e4	266687
4.451815	up	XM_001056041	Slc35f5	288993
2.8935685	up	XM_001067156	Slc37a3	312255
29.073355	up	NM_031589	Slc37a4	29573
51.495216	up	NM_181090	Slc38a2	29642
4.5956936	up	NM_001035251	Slc38a9	310091
8.609522	up	NM_001039196	Slc39a13	295928

15.033505	up	NM_019283	Slc3a2	50567
2.6164434	up	NM_133315	Slc40a1	170840
2.573153	up	XM_001059484	Slc41a1	363985
6.6446576	up	XM_001065058	Slc43a1	311168
2.4699044	up	XM_001080703	Slc43a2	287532
3.626061	up	XM_001069690	Slc43a3	311170
13.988011	up	NM_001033852	Slc44a1	85254
2.1735952	up	NM_001024968	Slc46a3	288454
4.3483706	up	XM_001067937	Slc4a1ap	298805
2.0550141	up	NM_017048	Slc4a2	24780
4.2588058	up	NM_017049	Slc4a3	24781
2.58269	up	NM_053424	Slc4a4	84484
7.8461237	up	NM_130746	Slc5a6	170551
11.139577	up	NM_017206	Slc6a6	29464
47.65488	up	NM_017348	Slc6a8	50690
2.20997	up	XM_001074656	Slc7a6os	246187
2.870488	up	XM_001053956	Slc9a6	302863
32.77783	up	NM_177481	Slco3a1	140915
4.8523407	up	NM_133608	Slco4a1	171144
3.0391235	up	XM_001064830	Slco5a1	312907
5.1188855	up	NM_053687	Slfn3	114247
3.8266156	up	XM_001057214	Slmap	290533
95.91186	up	NM_001013247	Sln	367086
3.8906302	up	XM_001067382	Slu7	303057
4.826588	up	NM_013130	Smad1	25671
3.6318076	up	NM_019191	Smad2	29357
2.765734	up	NM_013095	Smad3	25631
6.013317	up	NM_019275	Smad4	50554
2.8799045	up	NM_021692	Smad5	59328
3.498182	up	NM_182817	Smagp	300236
45.8315	up	XM_001073453	Smarca4	171379
5.0602164	up	XM_001074047	Smarca5	307766
2.146983	up	XM_001066395	Smarcad1	312398
4.8971004	up	NM_001025728	Smarcb1	361825
2.551494	up	NM_031983	Smarcd2	83833
2.4465587	up	XM_001069997	Smc4	295107
2.7858	up	XM_001056555	Smchd1	316732
3.3714314	up	NM_001007709	Smcr7l	315141
2.401952	up	XM_001065812	Smek1	314388
2.6155045	up	XM_001053322	Smek2	360993
4.757347	up	NM_022509	Smn1	64301
6.925736	up	NM_001025400	Smndc1	287768
12.02527	up	XM_001053050	Smoc2	292401
15.675182	up	NM_001006997	Smpd1	308909

5.3640623	up	NM_001005539	Smpdl3a	294422
29.57417	up	NM_001025737	Smpdl3b	362619
2.4886558	up	XM_001066720	Smtn	289734
33.00937	up	XM_001069604	Smtnl1	311167
5.488242	up	XM_001053796	Smtnl2	679629
31.368296	up	NM_057195	Smu1	117541
22.072325	up	XM_001062526	Smyd1	297333
41.304718	up	XM_001066311	Smyd2	289372
3.0430236	up	NM_001025762	Smyd3	498295
2.0365353	up	XM_001080679	Smyd4	287525
4.1633997	up	NM_022689	Snap23	64630
3.6471264	up	NM_053810	Snap29	116500
4.708569	up	NM_001025648	Snapap	295217
2.3803818	up	NM_001013121	Snapc2	304204
3.68304	up	NM_001013212	Snapc3	362537
4.4746523	up	NM_031688	Sncg	64347
9.17773	up	NM_022694	Snd1	64635
6.0759997	up	NM_001007804	Snf8	287645
2.6685898	up	XM_001074745	Snrnp27	362392
9.23275	up	NM_001014127	Snrnp35	360803
19.791256	up	XM_232775	Snrnp40	313056
5.7257195	up	XM_001079238	Snrnp70	361574
2.9781199	up	NM_001008303	Snrpa	292729
4.877392	up	XM_001059527	Snrpb	171365
29.573055	up	XM_001053334	Snrpb2	362223
5.3010254	up	XM_001060646	Snrpd1	291794
2.4545147	up	XM_001058626	Snrpf	680737
39.441887	up	XM_001064932	Snta1	362242
3.4757576	up	XM_001059272	Snw1	500695
6.0830846	up	NM_053411	Snx1	84471
3.205776	up	XM_001069787	Snx12	363478
7.8595557	up	XM_001076819	Snx13	362731
5.8753743	up	XM_001065843	Snx14	315871
4.0562897	up	NM_022289	Snx16	64088
11.794777	up	NM_001011981	Snx17	298836
7.729439	up	XM_001057944	Snx2	291464
2.8665364	up	XM_001061047	Snx25	306471
11.254739	up	NM_152847	Snx27	260323
11.611617	up	XM_001053951	Snx5	296199
4.880645	up	NM_001012083	Snx7	310815
2.1458406	up	XM_001067064	Snx9	683687
4.847493	up	NM_058208	Socs2	84607
18.018675	up	XM_001063512	Socs6	307200
65.15164	up	NM_017050	Sod1	24786

102.36897	up	NM_017051	Sod2	24787
3.1591833	up	NM_012880	Sod3	25352
20.325779	up	XM_001068992	Son	304092
3.0704238	up	NM_017052	Sord	24788
5.1558013	up	XM_001065506	Sorl1	300652
5.672124	up	XM_001070445	Sort1	83576
8.544011	up	XM_001062567	Sos1	313845
10.878283	up	XM_001068302	Sox4	364712
7.639705	up	NM_001024751	Sox6	293165
6.590326	up	NM_012655	Sp1	24790
5.6038647	up	NM_001014220	Sp100	363269
2.002781	up	NM_001034137	Sp110	301570
4.648425	up	NM_001012133	Sp140	316580
20.301756	up	XM_001079933	Spag7	303260
8.84674	up	XM_001081278	Spag9	360600
370.45377	up	NM_012656	Sparc	24791
94.00192	up	NM_012946	Sparcl1	25434
7.418442	up	XM_001065741	Spast	362700
2.5251698	up	XM_001069059	Spata5	361935
3.6481657	up	NM_138862	Spata7	192225
2.6424096	up	NM_001014102	Spats2l	316426
21.85303	up	XM_001066421	Spcs2	293142
3.0088186	up	XM_001058844	Spcs3	680782
2.5197465	up	XM_001058207	Speg	363256
3.1238525	up	XM_242139	Spg11	311372
3.168377	up	XM_215564	Spg20	295053
16.244648	up	NM_001006987	Spg21	300791
2.474882	up	NM_181388	Spg7	353231
3.2267656	up	NM_001012066	Sphk2	308589
5.6859784	up	NM_199087	Spint2	292770
12.484652	up	NM_171983	Spna2	64159
4.9624047	up	NM_138533	Spon2	171569
87.52961	up	XM_001072862	Spop	287643
16.639465	up	XM_001081083	Sppl2a	311401
5.538901	up	XM_001080325	Sppl3	360822
3.6331797	up	NM_001037765	Spryd4	288772
4.774429	up	XM_001058835	Spsb3	302981
30.479889	up	NM_001013130	Sptbn1	305614
7.7687173	up	XM_001053124	Sptlc1	361213
3.125635	up	NM_001037097	Sptlc2	366697
8.188812	up	XM_001080417	Sqrdl	691966
177.9301	up	NM_175843	Sqstm1	113894
8.106929	up	NM_183329	Sra1	252891
3.1591947	up	NM_001013990	Srd5a3	305291

6.727992	up	XM_001075680	Srebf1	78968
3.3977995	up	XM_001058349	Srgap2	360840
9.385821	up	NM_020074	Srgn	56782
20.219128	up	XM_001079265	Srl	302948
59.82435	up	XM_001080727	Srp14	296076
14.3006315	up	XM_001060949	Srp19	291685
3.795616	up	XM_001066751	Srp72	498351
3.0484786	up	NM_001025726	Srpk1	361811
11.509358	up	XM_001059468	Srpk2	296753
68.85554	up	NM_001034150	Srpr	315548
2.2195785	up	NM_001013252	Srprb	300965
4.3916287	up	NM_198757	Srr	303306
23.361647	up	XM_001069368	Srrm1	313620
6.23345	up	XM_001073112	Ss18	361295
12.192081	up	NM_031119	Ssb	81783
2.1027489	up	NM_053358	Ssbp3	84354
4.26452	up	XM_001080435	Ssh1	304580
2.55193	up	XM_001080672	Ssh2	303342
13.20514	up	XM_001072559	Ssna1	311802
9.048317	up	NM_001008891	Ssr1	361233
16.009014	up	XM_001070197	Ssr2	295235
4.3864164	up	NM_031120	Ssr3	81784
12.31243	up	NM_017199	Ssr4	29435
34.86875	up	NM_031121	Ssrp1	81785
4.1729527	up	NM_175597	Ssx2ip	308023
15.577959	up	NM_031122	St13	81800
3.5300026	up	NM_031695	St3gal2	64442
3.4128883	up	NM_031697	St3gal3	64445
2.2611957	up	NM_203337	St3gal4	363040
15.701031	up	NM_031337	St3gal5	83505
8.051219	up	NM_207602	St3gal6	304023
3.4821784	up	NM_001007639	St7l	295344
139.47339	up	XM_001056439	Stac3	362895
2.9829845	up	XM_001067112	Stag1	315958
8.715255	up	XM_001059059	Stag2	313304
14.290431	up	XM_001067909	Stam	498798
5.677537	up	NM_138531	Stambp	171565
3.865446	up	NM_001025026	Stap2	363334
9.1150675	up	NM_001014229	Stard3	363675
2.3454545	up	NM_001008298	Stard3nl	291182
2.36085	up	NM_001007627	Stard6	291527
29.767834	up	XM_238280	Stard7	296128
5.8703547	up	NM_032612	Stat1	25124
5.282711	up	NM_001011905	Stat2	288774

28.379307	up	NM_012747	Stat3	25125
6.355762	up	NM_022380	Stat5b	25126
4.8822336	up	NM_001044250	Stat6	362896
2.7743556	up	NM_053436	Stau1	84496
3.6546478	up	NM_001007149	Stau2	171500
114.93144	up	NM_001013988	Stbd1	305234
9.186063	up	NM_001044265	Steap4	499991
5.6551495	up	XM_001069624	Stim1	361618
7.0046034	up	XM_001053661	Stim2	117087
34.2361	up	NM_138911	Stip1	192277
6.101223	up	XM_001076821	Stk11	314621
4.231417	up	XM_237307	Stk11ip	301535
2.5846457	up	NM_173142	Stk16	286927
6.446534	up	NM_184049	Stk25	373542
2.1913497	up	NM_031735	Stk3	65189
4.4275956	up	NM_001015025	Stk38	361813
2.0158567	up	NM_053440	Stmn2	84510
7.544323	up	NM_001011965	Stom	296655
5.966874	up	NM_182820	Strada	303605
15.290141	up	XM_001069230	Stradb	501146
37.575413	up	NM_001011969	Strap	297699
3.2131739	up	NM_053416	Strbp	84476
2.1379578	up	NM_019148	Strn	29149
55.85312	up	NM_001029897	Strn3	114520
2.1744885	up	XM_001074709	Strn4	308392
8.898188	up	NM_012661	Sts	24800
6.358983	up	XM_001061492	Stt3a	500972
46.420593	up	NM_001025625	Stub1	287155
11.83967	up	NM_001012151	Stx18	360953
2.8746324	up	NM_012748	Stx2	25130
14.924291	up	NM_031125	Stx4	81803
3.1037025	up	NM_031656	Stx8	59074
3.181555	up	NM_013038	Stxbp1	25558
2.5814357	up	NM_030843	Stxbp5	81022
2.4898858	up	XM_001070116	Styx	689246
32.098236	up	XM_001071843	Sucla2	361071
16.813814	up	NM_053752	Suclg1	114597
22.087168	up	XM_001064568	Suclg2	362404
11.211712	up	XM_001080131	Suds3	360819
3.0242114	up	XM_001073449	Sugt1	290408
6.8800077	up	NM_031834	Sult1a1	83783
3.8764122	up	NM_001025125	Sumf2	360800
18.397665	up	NM_001009672	Sumo1	301442
2.8356776	up	NM_001024295	Sumo3	499417

14.924642	up	NM_031127	Suox	81805
3.787657	up	XM_001075690	Supt16h	305851
5.091207	up	XM_001081172	Supt4h1	287608
13.465006	up	XM_001078198	Supt5h	308472
21.412207	up	XM_001080796	Supt6h	303281
2.994279	up	XM_001062292	Supt7l	313905
2.6984596	up	NM_172068	Surf1	64463
20.143686	up	NM_001033868	Surf4	619346
9.361538	up	NM_001015014	Surf6	303076
3.384312	up	XM_001079843	Susd2	294335
2.0309434	up	XM_001068585	Suv420h1	361688
11.898235	up	XM_001056293	Svil	361256
30.462496	up	NM_133417	Syf2	170933
24.040035	up	XM_001060926	Sync	362606
2.2469416	up	NM_001029909	Syne1	499010
25.394108	up	NM_053553	Syngr2	89815
3.8829646	up	XM_001073290	Synj1	85238
2.1251729	up	NM_032071	Synj2	84018
18.314487	up	NM_022599	Synj2bp	64531
2.430151	up	XM_001055657	Synm	308709
14.736811	up	XM_001074385	Synpo2	499702
9.781865	up	XM_001064648	Synpo2l	305675
5.7941666	up	NM_053419	Synrg	84479
25.211777	up	NM_001014263	Sypl1	366595
9.483283	up	XM_001071432	Sypl2	362018
35.449776	up	NM_001004415	Tacc2	309025
4.4245806	up	XM_001063180	Tada1l	360874
4.974516	up	XM_001063619	Tada2b	289717
2.103353	up	NM_001012141	Tada2l	360581
2.2797585	up	XM_001062309	Tada3lb	291150
9.696742	up	NM_001008350	Taf11	309638
10.710846	up	XM_001065010	Taf12	682902
2.7234259	up	XM_001071074	Taf13	310784
4.979008	up	XM_001074480	Taf1b	690450
3.6835902	up	XM_001066741	Taf2	170844
3.944533	up	NM_001044225	Taf6	288533
12.107426	up	NM_001037310	Taf9	373541
3.087004	up	NM_133615	Taf9b	171152
22.591434	up	NM_031549	Tagln	25123
11.464425	up	NM_031811	Taldo1	83688
10.319441	up	NM_145788	Tank	252961
7.5373445	up	NM_032055	Tap1	24811
2.795602	up	NM_033098	Tapbp	25217
15.705043	up	NM_001034941	Tarbp2	363006

14.017553	up	NM_001011979	Tardbp	298648
23.763578	up	NM_001006976	Tars	294810
2.558109	up	NM_001014040	Tars2	310672
13.648179	up	NM_001014020	Tarsl2	308701
6.8122406	up	XM_001078895	Tatdn2	500295
7.6736617	up	NM_001025748	Taz	363521
4.5656753	up	XM_001078373	Tbc1d10b	365372
2.9895184	up	NM_001012152	Tbc1d14	360956
105.91305	up	XM_001077952	Tbc1d17	292886
2.8607335	up	NM_001004281	Tbc1d20	362237
10.564489	up	XM_001060935	Tbc1d23	304019
3.7716105	up	XM_001063148	Tbc1d25	302552
6.5188437	up	XM_001055741	Tbc1d8	680133
7.0257726	up	XM_001070650	Tbc1d9b	360520
3.6229105	up	NM_001013245	Tbca	366995
8.821481	up	NM_001040180	Tbcb	292777
3.7846437	up	NM_001012016	Tbccd1	303830
12.411101	up	NM_001012161	Tbce	361255
4.433843	up	XM_001057805	Tbl1x	302711
2.343786	up	XM_001056572	Tbl1xr1	365755
4.3944936	up	NM_001008277	Tbl3	287120
5.4037876	up	NM_001004198	Tbp	117526
2.7430067	up	XM_001069243	Tbpl1	689030
7.249208	up	NM_001009344	Tbrg1	300521
5.131977	up	NM_001012154	Tbrg4	360977
27.890188	up	XM_001062167	Tbx15	295315
4.0302596	up	NM_001025735	Tcea1	362479
16.791462	up	NM_001015008	Tcea3	298559
2.132263	up	NM_001009675	Tceal1	302593
23.70095	up	NM_001014275	Tceal8	367909
13.472752	up	NM_017103	Tceb3	25562
2.4163644	up	XM_001068365	Tcerg1	307474
4.1489377	up	NM_013176	Tcf12	25720
2.399799	up	XM_001077683	Tcf20	366964
2.4137049	up	XM_001065059	Tcf3	312451
12.432598	up	NM_053369	Tcf4	84382
9.843738	up	XM_001063604	Tcfcp2	315309
4.2247	up	XM_222232	Tchp	304547
4.4803305	up	NM_199089	Tcirg1	293650
8.529446	up	NM_022534	Tcn2	64365
43.992077	up	NM_012670	Tcp1	24818
2.4428337	up	XM_001079577	Tcp11l1	499846
5.2861276	up	NM_001017458	Tcp11l2	314683
7.9822245	up	NM_053729	Tdg	114521

7.148851	up	NM_138871	Tdrd7	85425
2.9440258	up	XM_001069278	Tead1	361630
2.6071842	up	XM_001069188	Tec	84492
39.179226	up	NM_138549	Tecr	191576
5.5353613	up	NM_019194	Tef	29362
9.790249	up	XM_001054185	Tek	89804
11.444727	up	XM_001066149	Tenc1	315326
5.5882444	up	NM_022591	Tep1	64523
19.43254	up	NM_001013143	Terf2ip	307861
2.0164244	up	NM_001039344	Tes	500040
8.934967	up	NM_031578	Tesk1	29460
7.134273	up	XM_001077411	Tet2	310859
8.375706	up	XM_001054011	Tex10	298065
10.16118	up	XM_001069235	Tex2	303611
5.676126	up	NM_001017537	Tex261	297392
2.7977042	up	NM_001013110	Tf	24825
13.418699	up	NM_031326	Tfam	83474
2.5103798	up	NM_181474	Tfb1m	308140
12.710503	up	NM_001008293	Tfb2m	289307
36.028976	up	NM_001012144	Tfg	360709
7.506134	up	NM_001008291	Tfip11	288718
5.124047	up	NM_017200	Tfpi	29436
7.3905373	up	NM_138870	Tfpt	85423
5.6238832	up	XM_001072774	Tfrc	64678
2.3606458	up	XM_001078278	Tgds	306164
3.1231596	up	NM_021578	Tgfb1	59086
4.8502	up	XM_001077389	Tgfb1i1	84574
13.911503	up	NM_031131	Tgfb2	81809
3.8347833	up	XM_001069069	Tgs1	312947
3.8025498	up	XM_001060686	Thada	313865
7.5894833	up	XM_001075997	Thap3	362667
5.8046765	up	NM_001005564	Thap4	363291
3.8968937	up	NM_031771	Thbd	83580
3.471169	up	NM_001013062	Thbs1	445442
21.910997	up	XM_001066338	Thbs4	29220
2.3835042	up	NM_001025017	Them4	361992
2.777633	up	NM_001025035	Thnsl1	498805
5.151883	up	NM_001009658	Thnsl2	297332
2.7449183	up	XM_001058768	Thoc2	313308
2.3132756	up	XM_001068440	Thoc3	290519
22.16199	up	XM_001075100	Thoc4	690585
8.364537	up	NM_001012153	Thoc5	360972
11.824297	up	NM_024384	Thoc6	79227
12.9194355	up	XM_223828	Thoc7	305714

2.9486523	up	NM_172075	Thop1	64517
5.972458	up	NM_001017960	Thra	81812
4.5005646	up	NM_001009693	Thrap3	313591
5.821069	up	NM_012703	Thrsp	25357
2.3374856	up	XM_001078607	Thumpd3	500288
4.862532	up	NM_001013193	Tial1	361655
7.084137	up	XM_001069541	Tie1	89806
6.718492	up	NM_172074	Timm10	64464
40.98484	up	NM_145781	Timm13	252928
2.654943	up	NM_019351	Timm17a	54311
17.28575	up	XM_001079918	Timm17al1	500655
11.015962	up	XM_001063717	Timm17b	317374
11.964027	up	NM_019352	Timm23	54312
3.7005744	up	NM_053370	Timm8a1	84383
60.684067	up	NM_022541	Timm8b	64372
3.6118462	up	XM_001072892	Timm9	171139
2.4513552	up	NM_053819	Timp1	116510
6.5505304	up	NM_021989	Timp2	29543
11.962485	up	NM_012886	Timp3	25358
6.7249618	up	NM_053582	Tinagl1	94174
3.186458	up	NM_001006962	Tinf2	290232
2.758118	up	NM_001025287	Tipin	363076
2.0614865	up	XM_341145	Tiprl	360869
4.826606	up	XM_001063874	Tjap1	316233
4.4870763	up	XM_001058833	Tjp1	292994
3.6992574	up	NM_053773	Tjp2	115769
4.6211243	up	XM_001066887	Tle1	362533
4.1548343	up	NM_019141	Tle4	25565
6.688349	up	XM_001059845	Tlk1	311118
3.5559616	up	XM_001067052	Tlk2	303592
8.421889	up	XM_001053379	Tln1	313494
2.19182	up	XM_001056519	Tln2	315776
2.189573	up	NM_198791	Tlr3	364594
18.892614	up	XM_001059680	Tm2d1	362545
11.605636	up	XM_001056632	Tm2d3	292995
2.1873083	up	XM_001060042	Tm4sf1	295061
2.4329147	up	XM_001067588	Tm6sf1	361600
11.123204	up	NM_001012155	Tm9sf1	361043
7.653879	up	NM_001005554	Tm9sf2	306197
2.2960315	up	XM_220013	Tm9sf3	309475
2.671133	up	NM_001025649	Tm9sf4	296279
3.9780033	up	NM_001007713	Tmbim1	316516
7.778121	up	NM_199116	Tmbim4	362884
139.24174	up	NM_019381	Tmbim6	24822

11.630592	up	XM_001054467	Tmcc1	312654
4.823231	up	XM_001059693	Tmcc2	305095
37.184242	up	NM_001009631	Tmco1	289196
33.076393	up	XM_001075325	Tmco3	306607
3.2398546	up	NM_001034949	Tmco4	500573
15.578094	up	XM_001065172	Tmco6	291661
3.3402088	up	NM_001013432	Tmed1	315461
13.280969	up	NM_053467	Tmed10	84599
123.172485	up	NM_031722	Tmed2	65165
4.822369	up	XM_001070563	Tmed4	305502
3.1559396	up	NM_001007619	Tmed5	289883
38.987255	up	NM_001017479	Tmem100	497979
15.79592	up	XM_001081505	Tmem101	303564
2.2407267	up	XM_001075724	Tmem103	363150
8.155112	up	NM_001004267	Tmem106b	312132
2.0789104	up	XM_001069716	Tmem108	300967
6.4904647	up	NM_001007736	Tmem109	361732
5.9036427	up	XM_001076426	Tmem11	303196
2.2403111	up	NM_198774	Tmem110	361110
70.19185	up	NM_001008355	Tmem111	312640
14.044705	up	XM_001074283	Tmem115	363136
2.3441873	up	XM_001074467	Tmem116	690442
13.012756	up	XM_576330	Tmem117	500921
5.993284	up	NM_001010945	Tmem120a	288591
5.369462	up	NM_001014205	Tmem123	363013
6.8517094	up	NM_001011557	Tmem126a	293113
6.748925	up	XM_001062219	Tmem126b	293114
8.07946	up	XM_001062482	Tmem128	360952
9.722207	up	XM_001056771	Tmem131	316335
10.265925	up	NM_001078647	Tmem134	361695
3.4154353	up	NM_001013896	Tmem135	293098
30.341251	up	NM_001009709	Tmem140	362334
3.5580919	up	XM_001074131	Tmem141	499755
2.1732857	up	XM_001063426	Tmem144	361968
30.071571	up	NM_001038494	Tmem147	292792
64.07969	up	NM_134395	Tmem14c	171432
5.442344	up	NM_139107	Tmem150a	245966
3.3921835	up	NM_001024802	Tmem165	364137
2.7851806	up	XM_001071753	Tmem167b	499690
2.9930074	up	NM_001013991	Tmem175	305623
13.537005	up	NM_001039008	Tmem176a	297077
11.77861	up	NM_134390	Tmem176b	171411
4.296761	up	NM_001037776	Tmem177	304735
3.9488144	up	XM_001067532	Tmem181	502228

13.254889	up	XM_001059514	Tmem182	501129
2.314724	up	NM_001013871	Tmem183a	289034
8.861563	up	NM_178330	Tmem184c	291946
2.3258088	up	XM_001053713	Tmem185a	309357
29.037449	up	XM_001053834	Tmem185b	304731
7.3049216	up	XM_001063368	Tmem188	291914
26.329903	up	XM_342588	Tmem189	362278
3.9584687	up	NM_199098	Tmem19	299800
3.5639935	up	NM_001014141	Tmem192	361137
24.15648	up	NM_001024992	Tmem199	303332
2.012429	up	XM_001079816	Tmem2	309400
3.0829306	up	XM_220029	Tmem20	294072
6.8589735	up	XM_001077527	Tmem205	300441
4.1639037	up	XM_001075578	Tmem208	291963
2.7027378	up	NM_001014195	Tmem214	362711
6.3054485	up	NM_001008325	Tmem218	300516
5.5449986	up	XM_001080074	Tmem219	308986
2.0067964	up	XM_001078792	Tmem220	287405
4.319412	up	XM_001066468	Tmem222	313021
2.3391147	up	XM_001078413	Tmem229b	503035
21.110767	up	NM_001004248	Tmem30a	300857
5.9592547	up	NM_001034198	Tmem33	59303
93.973526	up	XM_001063213	Tmem38a	306327
140.70901	up	NM_001014191	Tmem38b	362521
8.109469	up	NM_001013865	Tmem39a	288092
11.593019	up	NM_001012358	Tmem41b	361626
2.6340168	up	XM_001060274	Tmem47	501569
14.93191	up	NM_138839	Tmem49	192129
3.9921927	up	NM_001024759	Tmem5	299841
48.703392	up	XM_001067840	Tmem50a	298552
15.005427	up	NM_001024900	Tmem55a	362490
10.298142	up	NM_001014233	Tmem55b	364298
3.6481526	up	NM_001025699	Tmem57	313618
13.178299	up	XM_001063081	Tmem60	296761
5.089363	up	XM_001080922	Tmem62	311350
83.50204	up	NM_001004213	Tmem66	290796
2.1017387	up	XM_001053339	Tmem67	313067
3.721856	up	XM_001062611	Tmem70	500384
4.3899193	up	NM_001017455	Tmem80	309109
14.148531	up	XM_001074437	Tmem85	296049
17.584198	up	XM_345422	Tmem87a	366170
2.517906	up	XM_001081122	Tmem87b	362212
3.0623999	up	XM_001079371	Tmem88	497936
6.8172317	up	XM_001063617	Tmem9	289046

28.995749	up	XM_001080240	Tmem93	287477
2.6638207	up	NM_001007672	Tmem98	303356
14.734984	up	XM_001073254	Tmem9b	293415
12.483091	up	NM_053671	Tmf1	114206
13.751808	up	NM_013044	Tmod1	25566
3.5333283	up	NM_001011997	Tmod3	300838
172.35704	up	XM_001054854	Tmod4	295261
5.893008	up	NM_012887	Tmpo	25359
28.860647	up	NM_031136	Tmsb4x	81814
6.3735757	up	NM_001031651	Tmub2	303567
19.59833	up	NM_001007643	Tmx2	295701
2.6304166	up	XM_001081259	Tmx4	296182
4.1251454	up	NM_001014039	Tnfaip8l2	310663
3.284269	up	NM_181086	Tnfrsf12a	302965
8.011082	up	NM_013091	Tnfrsf1a	25625
2.478551	up	XM_001065930	Tnfrsf26	361685
3.754494	up	NM_145681	Tnfsf10	246775
3.744889	up	NM_001001513	Tnfsf12	360548
2.9482703	up	NM_001009623	Tnfsf13	287437
3.0825331	up	XM_001057689	Tnik	294917
26.730883	up	XM_001073849	Tnip1	363599
2.6061065	up	NM_001024771	Tnip2	305451
4.0047264	up	XM_001059294	Tnks	290794
8.597695	up	XM_001080123	Tnks2	309512
35.319096	up	NM_001034105	Tnnc1	290561
17.569952	up	NM_001037351	Tnnc2	296369
105.68704	up	NM_017185	Tnni2	29389
188.97446	up	NM_134388	Tnnt1	171409
2.85216	up	NM_012676	Tnnt2	24837
418.34006	up	NM_031532	Tnnt3	24838
5.778236	up	XM_001070389	Tnpo1	309126
2.116883	up	XM_001077084	Tnrc6b	192178
4.1484604	up	XM_001078761	Toag1	363169
6.8396025	up	NM_133317	Tob1	170842
5.344343	up	NM_001007146	Tob2	315159
5.184664	up	XM_001071317	Toe1	298443
13.955829	up	NM_001008365	Tom1	361370
14.961368	up	NM_152935	Tomm20	266601
2.8052802	up	NM_212514	Tomm22	300075
23.00996	up	NM_001044244	Tomm34	311621
2.0860832	up	NM_212520	Tomm40	308416
3.9927206	up	XM_001055588	Tomm5	680080
13.840324	up	NM_212519	Tomm70a	304017
7.261145	up	NM_022615	Top1	64550

3.8783395	up	NM_001002798	Top1mt	300029
7.5755043	up	XM_001067804	Top3b	287930
3.7933147	up	XM_001072207	Topbp1	315969
4.9382567	up	NM_001007744	Tor2a	362112
6.581547	up	NM_001009683	Tor3a	304884
9.713461	up	NM_173324	Tox4	286990
3.7208562	up	NM_030989	Tp53	24842
4.926656	up	XM_001080798	Tp53i13	287550
3.2805448	up	NM_181084	Tp53inp1	297822
13.829742	up	NM_022922	Tpi1	24849
54.747787	up	NM_001034068	Tpm1	24851
121.39538	up	NM_001024345	Tpm2	500450
5.7038846	up	NM_012678	Tpm4	24852
11.641014	up	NM_031357	Tpp1	83534
9.897784	up	NM_031137	Tpp2	81815
10.677362	up	NM_001009639	Tppp3	291966
2.5684092	up	XM_001070158	Tpr	304862
4.897536	up	NM_001013926	Tprkb	297411
2.0278895	up	NM_001011903	Tpst1	288617
155.87791	up	NM_053867	Tpt1	116646
3.3347583	up	NM_057119	Tra2b	117259
13.999273	up	XM_001054533	Trabd	300142
82.8039	up	XM_001077727	Traf2	311786
2.1769636	up	NM_001014132	Traf3ip3	360900
9.811643	up	XM_001055375	Traf7	360491
2.6622856	up	XM_001076427	Trafd1	114635
2.1915221	up	XM_001077998	Trak1	316085
3.9250085	up	NM_133560	Trak2	171086
37.271877	up	NM_001007701	Tram1	312903
16.45892	up	NM_001039001	Trap1	287069
9.242856	up	NM_001039378	Trappc1	287427
4.2548237	up	NM_001024965	Trappc2	501550
6.137295	up	XM_001077701	Trappc2l	292074
3.2982738	up	XM_001063935	Trappc5	363858
27.378826	up	XM_001079724	Trappc6b	299075
4.835637	up	XM_001068491	Trappc9	315059
120.099655	up	NM_021666	Trdn	59299
4.3297997	up	NM_001024989	Trex1	1E+08
12.555313	up	XM_001077518	Triap1	691278
3.130157	up	NM_023985	Trib1	78969
2.674202	up	XM_001068999	Trib2	313974
2.128554	up	NM_144755	Trib3	246273
5.9130945	up	XM_001078237	Trim16	303214
2.5555906	up	XM_001064021	Trim23	81002

3.0257592	up	NM_001009536	Trim25	494338
4.244451	up	XM_001059726	Trim27	291171
12.185571	up	XM_001062234	Trim28	116698
12.660173	up	XM_001081108	Trim37	360592
2.8938756	up	NM_213562	Trim39	309591
4.423178	up	XM_001069403	Trim41	303088
2.8984416	up	NM_001014023	Trim5	308906
296.32816	up	NM_001013217	Trim54	362708
3.5632997	up	NM_001012218	Trim55	365751
156.99225	up	NM_080903	Trim63	140939
14.830323	up	NM_001077675	Trim72	365377
5.1940784	up	XM_001058824	Trio	310192
42.844517	up	NM_053920	Trip10	116717
40.342632	up	NM_001031659	Trip12	316575
8.848386	up	XM_001054252	Trit1	362586
5.9292774	up	NM_001013870	Trmt1	288914
3.7055752	up	XM_001078327	Trmu	362976
8.117963	up	NM_001024261	Trnt1	312616
6.133468	up	XM_001076425	Trpt1	293704
6.0662937	up	XM_001071336	Trrap	288471
6.930634	up	NM_001012173	Trub1	361775
14.632943	up	NM_001014257	Trub2	366012
3.1204925	up	NM_021854	Tsc1	60445
41.865753	up	NM_012680	Tsc2	24855
46.304993	up	NM_031345	Tsc22d3	83514
34.402958	up	XM_001066199	Tsen15	289083
3.7568872	up	XM_001074043	Tsen54	690308
21.374758	up	NM_181628	Tsg101	292925
10.481195	up	XM_001060440	Tshz1	307217
6.2158923	up	NM_021762	Tsn	60381
8.85503	up	NM_022262	Tsnax	64028
21.434511	up	NM_001015026	Tspan12	362326
102.738335	up	NM_001013244	Tspan13	366602
2.4828563	up	XM_001067025	Tspan14	306324
8.5229225	up	NM_001005547	Tspan3	300733
17.044144	up	NM_001008378	Tspan31	362890
6.676943	up	XM_001062106	Tspan33	500065
3.2068717	up	NM_001013070	Tspan4	293627
4.573798	up	XM_001067945	Tspan6	302313
26.60913	up	XM_001062155	Tspan7	363447
27.59086	up	NM_133526	Tspan8	171048
2.7211692	up	XM_001064403	Tspyl2	302612
9.762722	up	NM_001012192	Tssc1	362721
9.722895	up	NM_001013194	Tssc4	361682

3.6986864	up	NM_012808	Tst	25274
49.289894	up	XM_001070202	Tsta3	300036
6.521591	up	XM_001071828	Tstd2	362514
46.027588	up	NM_001005529	Ttc1	287208
6.744189	up	XM_001054055	Ttc13	292095
3.461385	up	XM_001071807	Ttc15	314013
11.727962	up	XM_230313	Ttc17	311224
2.065907	up	XM_001064202	Ttc18	361006
4.3543878	up	NM_001025681	Ttc23	308708
8.258255	up	XM_001065384	Ttc27	298782
5.125195	up	XM_001054505	Ttc3	360702
15.541364	up	XM_001054997	Ttc33	294774
10.029926	up	XM_001062644	Ttc35	362905
2.894199	up	XM_001059136	Ttc37	294595
11.356069	up	XM_001064941	Ttc7b	362768
5.529614	up	XM_001054586	Ttc9	500689
2.3781037	up	XM_001079280	Ttf1	499766
2.5821514	up	NM_138536	Ttl	171572
2.9168248	up	NM_001012200	Ttll1	362969
2.4875777	up	XM_001077354	Ttll7	310982
582.9465	up	XM_001065955	Ttn	84015
10.717715	up	NM_022298	Tuba1a	64158
20.091497	up	NM_001044270	Tuba1b	500929
15.986351	up	NM_001011995	Tuba1c	300218
2.4893675	up	NM_001040008	Tuba3a	500319
285.4124	up	NM_001007004	Tuba4a	316531
268.39438	up	NM_001024339	Tuba8	500377
10.73573	up	XM_001060757	Tubb2a	498736
2.075595	up	NM_001013886	Tubb2b	291081
43.79253	up	NM_199094	Tubb2c	296554
122.14458	up	NM_173102	Tubb5	29214
261.6368	up	NM_001025675	Tubb6	307351
23.14864	up	NM_145778	Tubg1	252921
12.417137	up	XM_001056506	Tubgcp2	309098
4.9152393	up	XM_001053658	Tubgcp5	308663
2.3473344	up	XM_001054588	Tubgcp6	362980
13.129246	up	XM_001077188	Tufm	293481
2.8292835	up	NM_001004212	Tusc3	290783
2.2641804	up	NM_001025744	Tusc4	363138
5.321626	up	NM_001033901	Tut1	499314
3.804599	up	NM_001008521	Twf1	315265
2.136334	up	XM_001076417	Twistnb	362728
3.2203686	up	XM_001072466	Twsg1	363294
20.009699	up	XM_001072039	Txlnb	308622

189.49178	up	NM_053800	Txn1	116484
16.665901	up	NM_053331	Txn2	79462
3.1466885	up	XM_001072487	Txndc16	361025
2.1355143	up	XM_001080157	Txndc17	287474
56.055073	up	NM_001008767	Txnip	117514
86.178894	up	NM_080887	Txnl1	140922
5.2290645	up	NM_001013891	Txnl4b	292008
10.545279	up	NM_031614	Txnrd1	58819
2.2573106	up	NM_022584	Txnrd2	50551
5.0396633	up	XM_216204	Txnrd3	297437
11.464327	up	XM_001078133	Tyw1	304423
4.703652	up	XM_001080203	Tyw3	499731
11.309626	up	NM_001008775	U2af1l4	361542
3.8559334	up	XM_001077658	U2af2	308335
20.180084	up	XM_001076929	Uba2	308508
11.898471	up	NM_057205	Uba3	117553
23.778742	up	NM_001009669	Uba5	300968
3.6289577	up	NM_031687	Uba52	64156
2.072887	up	XM_001074883	Uba6	305268
3.2582521	up	XM_001074704	Uba7	301000
59.128654	up	NM_001007742	Ubac1	362087
9.624087	up	NM_001034937	Ubac2	361094
2.500428	up	NM_001012190	Ubap1	362502
95.750854	up	XM_001068128	Ubap2	313169
11.249625	up	NM_138895	Ubb	192255
21.38745	up	NM_017314	Ubc	50522
5.2269526	up	NM_001013933	Ube2a	298317
44.947117	up	NM_031138	Ube2b	81816
9.665915	up	XM_342125	Ube2d1	361831
20.177017	up	NM_001037292	Ube2d2	641452
193.99959	up	NM_031237	Ube2d3	81920
2.4828422	up	NM_001047857	Ube2e3	295686
3.3760533	up	NM_001008381	Ube2f	363284
8.8041725	up	NM_022690	Ube2g1	64631
69.563255	up	XM_001079504	Ube2g2	294331
62.506725	up	XM_216362	Ube2j1	297961
22.006174	up	NM_001007655	Ube2j2	298689
7.603584	up	XM_001071289	Ube2k	289623
23.486403	up	XM_001067198	Ube2l3	363836
16.551983	up	XM_001062179	Ube2m	361509
10.810065	up	XM_001074719	Ube2q1	295252
6.641257	up	XM_001072896	Ube2q2	363065
5.347789	up	XM_001071810	Ube2s	292588
5.284482	up	NM_001037643	Ube2z	303478

5.110038	up	XM_001055915	Ube3a	361585
6.10836	up	XM_001055148	Ube3c	362294
15.755323	up	NM_207610	Ube4a	315608
6.7016835	up	XM_001075491	Ube4b	298652
37.94907	up	NM_001015030	Ubl3	363869
3.4084492	up	XM_001055202	Ubl4	293864
6.086616	up	NM_001048243	Ubl5	500954
2.2648458	up	XM_001079088	Ubn1	302935
3.931021	up	XM_001066467	Ubn2	312248
4.182078	up	XM_001076644	Ubp1	301038
17.919718	up	NM_053747	Ubqln1	114590
6.404967	up	XM_001061090	Ubqln2	317396
25.416704	up	XM_001074121	Ubqln4	310633
57.23791	up	NM_001039026	Ubr4	313658
18.428228	up	XM_001061308	Ubr5	117060
7.5413837	up	NM_001007705	Ubr7	314399
2.543485	up	XM_001081508	Ubtf	25574
32.088	up	NM_001034829	Ubxn1	293719
12.835782	up	NM_001012025	Ubxn4	304766
49.5367	up	XM_001059416	Ubxn6	363332
3.8510184	up	XM_001059749	Ubxn8	290802
10.191329	up	NM_001012149	Uchl5	360853
5.2817736	up	XM_001078114	Uck1	311864
8.839973	up	NM_019354	Ucp2	54315
180.26329	up	NM_013167	Ucp3	25708
41.206635	up	NM_001003709	Ufc1	445268
39.36738	up	NM_053418	Ufd1l	84478
3.5823674	up	XM_001057949	Ufm1	365797
7.478493	up	NM_001014142	Ufsp2	361151
2.5478315	up	NM_133596	Uggt1	171129
34.131836	up	NM_001024743	Ugp2	289827
3.0544686	up	NM_001008882	Uhrf1	316129
4.029779	up	XM_001076826	Uhrf1bp1l	363009
14.29678	up	NM_001013884	Uimc1	290997
6.3612156	up	NM_001025402	Umps	288051
3.4823534	up	NM_017188	Unc119	29402
6.806744	up	NM_001037647	Unc45a	308759
70.01407	up	XM_001081034	Unc45b	303373
8.429974	up	NM_138919	Unc50	192356
2.051949	up	XM_001076724	Unc84b	315135
6.886159	up	XM_001069535	Unc93b1	361689
2.4452474	up	XM_341556	Upf2	361271
4.6462793	up	NM_001012159	Upf3a	361176
2.9056487	up	XM_001058104	Upf3b	313449

2.6988118	up	NM_001030025	Upp1	289801
3.8194575	up	XM_001075870	Uqcr	690848
2.8702593	up	XM_001057294	Uqcrb	362897
3.6655972	up	XM_001074011	Uqcrb-ps1	690049
65.71776	up	NM_001004250	Uqcrc1	301011
79.84536	up	NM_001006970	Uqcrc2	293448
89.86711	up	NM_001008888	Uqcrfs1	291103
56.877094	up	NM_001009480	Uqcrh	366448
101.72796	up	NM_001025134	Uqcrq	497902
2.0278828	up	XM_001053807	Urb2	292087
3.3031416	up	XM_001067008	Urod	29421
2.5708528	up	NM_001012068	Uros	309070
17.195192	up	XM_001065114	Use1	290627
6.682023	up	NM_031777	Usf1	83586
2.123786	up	NM_031139	Usf2	81817
4.3608775	up	NM_133544	Usmg5	171069
8.578229	up	NM_019379	Uso1	56042
3.2822888	up	NM_001015015	Usp1	313387
9.672792	up	NM_001034146	Usp10	307905
77.42148	up	XM_001066621	Usp13	310306
13.955012	up	NM_001008301	Usp14	291796
17.828396	up	NM_145184	Usp15	171329
8.67742	up	XM_001066902	Usp16	288306
36.52818	up	NM_001001516	Usp19	361190
5.256408	up	NM_053774	Usp2	115771
3.415782	up	XM_001079722	Usp20	311856
3.505973	up	XM_001067071	Usp21	688466
4.2610807	up	XM_001064937	Usp24	313427
38.71389	up	XM_001065002	Usp25	304150
21.850147	up	XM_001070037	Usp28	315639
3.0983472	up	NM_001025424	Usp3	363084
2.6904542	up	XM_001080428	Usp30	304579
5.5635843	up	XM_001081109	Usp32	303394
2.0324543	up	XM_001081749	Usp36	303700
6.0478654	up	XM_001073885	Usp38	307764
18.5368	up	XM_001063779	Usp39	297336
34.449623	up	XM_001074606	Usp4	290864
2.4679208	up	XM_001065510	Usp40	316599
3.7032576	up	XM_001072044	Usp42	288482
2.3072784	up	XM_232828	Usp45	313098
14.345444	up	XM_001074180	Usp47	308896
3.2506945	up	NM_198785	Usp48	362636
2.1429744	up	XM_001064731	Usp49	316211
10.94093	up	XM_001063162	Usp5	297593

2.9518566	up	XM_001074318	Usp53	295425
16.683807	up	XM_001078286	Usp8	296121
22.536137	up	XM_001056701	Usp9x	363445
4.610718	up	NM_001014113	Utp14a	317579
4.358219	up	XM_001069733	Utp15	310019
9.661753	up	XM_001081275	Utp18	303456
3.987091	up	XM_235023	Utp20	314713
7.0745625	up	XM_001063726	Utp23	299900
8.811844	up	XM_001080950	Utp6	360574
6.2209935	up	NM_139336	Uxs1	246232
8.871711	up	NM_012663	Vamp2	24803
9.858836	up	NM_057097	Vamp3	29528
25.737034	up	NM_053555	Vamp5	89818
46.484863	up	NM_053531	Vamp7	85491
8.218039	up	XM_001076616	Vars	25009
2.8774602	up	XM_001066594	Vash2	498309
100.08742	up	NM_053864	Vcp	116643
2.871883	up	NM_176857	Vcpip1	286761
6.585224	up	NM_031353	Vdac1	83529
31.607853	up	NM_031354	Vdac2	83531
222.15518	up	NM_031355	Vdac3	83532
2.077691	up	NM_031836	Vegfa	83785
41.999565	up	NM_053549	Vegfb	89811
67.03252	up	XM_001081191	Vezf1	287615
17.218042	up	NM_001006984	Vezt	299738
6.615902	up	XM_001058013	Vgll2	309772
3.4903054	up	NM_001015004	Vgll4	297523
86.00565	up	NM_031140	Vim	81818
5.711189	up	NM_203335	Vkorc1	309004
3.9580567	up	NM_203338	Vkorc1l1	399684
6.472072	up	NM_013155	Vldlr	25696
9.455453	up	XM_001067193	Vma21	501658
8.715701	up	XM_001071998	Vprbp	315987
3.7882278	up	XM_001061508	Vps11	315600
5.3981824	up	NM_001005541	Vps16	296159
9.743979	up	NM_172331	Vps24	282834
7.052957	up	NM_001007740	Vps26a	361846
2.7444286	up	XM_001054836	Vps26b	300472
46.106354	up	XM_001073010	Vps28	300052
6.2146926	up	XM_001079533	Vps29	288666
2.5724227	up	NM_022961	Vps33a	65081
2.7456133	up	NM_022286	Vps33b	64060
13.176169	up	XM_001068608	Vps35	25479
22.879492	up	XM_214382	Vps36	290851

3.1156187	up	NM_001024867	Vps37a	290775
3.166727	up	XM_001075756	Vps39	362199
5.0534005	up	NM_172072	Vps45	64516
16.984556	up	NM_145678	Vps4a	246772
3.4991486	up	XM_001080583	Vps53	287535
29.543991	up	XM_001059613	Vps72	310661
3.3880343	up	NM_001012194	Vrk1	362779
2.3866124	up	XM_001064313	Vrk2	360991
3.0260231	up	NM_001005561	Vrk3	361565
3.1175532	up	NM_001025640	Vta1	292640
8.4122715	up	NM_023101	Vti1a	65277
4.239105	up	NM_019156	Vtn	29169
2.6461318	up	NM_198755	Vwa5a	301097
17.75224	up	NM_001013170	Wars	314442
4.0016212	up	XM_001055085	Wbp1	297381
4.0824294	up	NM_138975	Wbp2	192645
4.3922153	up	XM_001056814	Wbp5	680354
2.2904885	up	XM_001077926	Wbscr16	360796
9.823382	up	XM_001071583	Wbscr22	368084
4.3497963	up	NM_001008331	Wdfy1	301549
2.112597	up	XM_001060345	Wdfy3	305164
22.93032	up	NM_001014135	Wdr1	360950
5.8965797	up	XM_001057763	Wdr13	317370
3.537874	up	NM_001039027	Wdr18	314617
8.836211	up	XM_001061380	Wdr24	360497
2.057412	up	XM_001069823	Wdr25l	314443
2.901129	up	XM_001062709	Wdr26	498301
11.942085	up	XM_001065786	Wdr3	310720
4.979683	up	XM_001072062	Wdr35	298876
2.2670357	up	XM_001060069	Wdr36	361305
4.267248	up	XM_001060920	Wdr37	307075
7.152963	up	XM_342175	Wdr41	361879
4.70698	up	XM_001059356	Wdr43	362703
3.6220675	up	XM_001061437	Wdr44	246152
23.796959	up	NM_001013958	Wdr45	302559
10.24599	up	NM_001039587	Wdr45l	360682
4.7948165	up	NM_212491	Wdr46	309628
5.6145134	up	XM_001071369	Wdr47	310785
2.8029964	up	XM_001055273	Wdr54	500226
2.4350145	up	NM_001017932	Wdr55	307494
2.9250686	up	XM_001061189	Wdr60	314523
3.3362367	up	XM_001075797	Wdr62	308492
2.536334	up	XM_216928	Wdr67	299949
2.142762	up	NM_001013909	Wdr70	294783

10.656297	up	XM_001076401	Wdr74	690229
7.287272	up	NM_001047889	Wdr75	314545
51.01465	up	NM_001008771	Wdr77	310769
3.6274145	up	NM_001014262	Wdr8	366515
2.3218684	up	XM_001065006	Wdr91	312225
3.3755736	up	NM_001014179	Wdsub1	362137
4.6868715	up	NM_001025024	Wdyhv1	362914
12.415003	up	NM_031823	Wfs1	83725
2.5437589	up	XM_001067296	Whamm	293057
2.6443386	up	XM_001058223	Whsc1	680537
2.609606	up	XM_001081631	Wipi1	303630
16.011665	up	NM_001007615	Wipi2	288498
3.6653295	up	NM_053402	Wnt4	84426
4.3619385	up	NM_001007610	Wrap53	287432
4.6085877	up	NM_172332	Wrnip1	282835
24.56577	up	NM_001007616	Wsb2	288692
7.130294	up	XM_001076479	Wwox	292041
3.4930267	up	NM_001024757	Wwp1	297930
3.8905852	up	XM_001076615	Wwp2	291999
26.362322	up	NM_001024869	Wwtr1	295062
7.762934	up	NM_139109	Xab2	245976
5.140749	up	NM_017154	Xdh	497811
2.6357558	up	NM_022231	Xiap	63879
50.05021	up	XM_001077697	Xirp1	316071
139.98167	up	NM_201989	Xirp2	311098
3.8622127	up	XM_001071899	Xpa	298074
3.7084682	up	NM_131913	Xpnpep1	170751
27.016739	up	NM_053490	Xpo1	85252
2.585424	up	XM_001063318	Xpo4	290280
2.3168151	up	XM_001064242	Xpo5	363194
4.1542616	up	XM_001076854	Xpo6	293476
6.1632633	up	XM_001071291	Xpo7	361070
5.7189145	up	XM_001079263	Xpot	314879
2.015866	up	XM_001072331	Xpr1	289424
11.273611	up	NM_177419	Xrcc5	363247
6.4577727	up	NM_139080	Xrcc6	25019
2.8600273	up	XM_001069029	Xrn1	300944
7.0028462	up	XM_001055882	Xrn2	362229
5.006454	up	NM_001009627	Yars2	287924
5.1857505	up	NM_031563	Ybx1	500538
2.226254	up	XM_001079683	Ybx2	303250
36.408142	up	XM_001078961	Yeats4	299810
6.455645	up	NM_001014810	Yif1b	292768
27.002125	up	NM_199383	Yipf1	298312

5.9582996	up	NM_001007801	Yipf3	301245
18.475431	up	NM_001009712	Yipf4	362699
3.219529	up	NM_001025747	Yipf6	363476
4.8290615	up	XM_001077453	Yipf7	364147
5.662004	up	NM_031692	Ykt6	64351
10.956371	up	XM_001057111	Ylpm1	299199
11.243466	up	NM_053682	Yme1l1	114217
6.561137	up	NM_001035221	Ypel5	298792
8.139636	up	NM_175604	Yrdc	319113
6.5887184	up	NM_133423	Ythdc1	170956
3.0057573	up	NM_001024756	Ythdf1	296467
7.571599	up	NM_001047099	Ythdf2	313053
5.248105	up	XM_001054853	Ythdf3	361920
9.870721	up	NM_019377	Ywhab	56011
223.97728	up	NM_031603	Ywhae	29753
4.725041	up	NM_019376	Ywhag	56010
25.785736	up	NM_013052	Ywhah	25576
9.413329	up	NM_013053	Ywhaq	25577
46.567875	up	NM_013011	Ywhaz	25578
5.258768	up	XM_001060611	Zadh2	291403
9.012709	up	NM_001004444	Zbtb1	314246
3.5867782	up	NM_024489	Zbtb10	80338
12.207973	up	NM_001012105	Zbtb17	313666
2.0970695	up	XM_001065469	Zbtb2	308126
2.01253	up	NM_001009172	Zbtb22	309630
2.431042	up	XM_001058234	Zbtb33	501506
4.826269	up	XM_001060735	Zbtb8os	297885
2.3147693	up	NM_213564	Zbtb9	294289
6.075636	up	XM_001065797	Zc3h10	685928
2.5432353	up	XM_001071727	Zc3h12c	315658
10.807872	up	NM_001033951	Zc3h14	192359
6.334379	up	NM_001010963	Zc3h15	362154
8.859978	up	NM_201416	Zc3h18	292067
2.6180274	up	XM_001076011	Zc3h7a	360466
6.067936	up	NM_001012090	Zc3h8	311414
2.6296196	up	NM_173045	Zc3hav1	252832
3.4379067	up	XM_233345	Zcchc11	313481
6.2532353	up	XM_001062322	Zcchc17	500555
4.8947783	up	XM_001060307	Zcchc6	501515
5.3845487	up	XM_001074938	Zcchc8	288661
19.464083	up	NM_001034940	Zcrb1	362990
9.883109	up	NM_001039099	Zdhhc1	291967
2.0020568	up	NM_001013239	Zdhhc12	366014
6.149835	up	NM_001039037	Zdhhc13	365252

24.455381	up	NM_001039346	Zdhhc16	654495
2.9942205	up	NM_001039340	Zdhhc17	366889
2.214826	up	NM_001039336	Zdhhc20	305923
17.842533	up	NM_001039014	Zdhhc3	301081
16.53707	up	NM_001039338	Zdhhc5	362156
6.4103546	up	NM_001037652	Zdhhc6	361771
5.9669213	up	NM_133394	Zdhhc7	170906
35.03429	up	NM_013164	Zeb1	25705
3.6185005	up	NM_001033701	Zeb2	311071
2.1990957	up	XM_001055634	Zfand1	361917
3.9350495	up	NM_001025745	Zfand2b	363253
3.3502226	up	NM_001012175	Zfand3	361816
34.326077	up	XM_001079764	Zfand5	293960
28.440191	up	NM_001007630	Zfand6	293067
2.339018	up	XM_001078674	Zfc3h1	314836
2.4638002	up	XM_001076847	Zfhx3	307829
3.0331116	up	XM_226964	Zfhx4	310250
2.8170803	up	XM_001077676	Zfp1	498952
8.756469	up	NM_001024775	Zfp110	308362
2.1229389	up	XM_001055283	Zfp13	287095
3.5201657	up	NM_001012169	Zfp143	361627
5.344076	up	XM_001060246	Zfp187	266792
2.8824759	up	NM_182955	Zfp191	360204
21.575607	up	NM_001039020	Zfp207	303763
7.841867	up	NM_022678	Zfp238	64619
2.5270426	up	XM_001075451	Zfp251	366954
3.6658878	up	XM_001064960	Zfp259	500989
3.3223035	up	NM_017364	Zfp260	53982
3.402894	up	XM_001077681	Zfp277	298977
2.2889652	up	XM_001076089	Zfp317	500950
2.678463	up	XM_001062211	Zfp318	685082
9.508473	up	XM_001073428	Zfp330	361387
3.2734828	up	XM_001060009	Zfp335	259270
7.712285	up	XM_001063740	Zfp358	360754
3.2199833	up	NM_133290	Zfp36	79426
2.1484807	up	XM_216345	Zfp362	297879
2.2984817	up	NM_001036626	Zfp36l2	298765
7.9526753	up	NM_001034830	Zfp384	171018
11.921985	up	XM_001058294	Zfp410	314310
16.409327	up	NM_001009664	Zfp414	299647
3.1500027	up	XM_001068393	Zfp428	361519
2.3405237	up	XM_001078410	Zfp445	301076
7.756338	up	NM_001033705	Zfp451	316312
2.0617747	up	XM_001078828	Zfp568	308488

4.407211	up	XM_001060627	Zfp706	500855
2.6342525	up	XM_001078207	Zfp709l2	362845
2.1654222	up	XM_001078778	Zfp74	365224
2.1951287	up	XM_001064404	Zfp780b	685578
6.385375	up	XM_001065858	Zfp828	306647
4.0035553	up	XM_001056210	Zfr	365703
2.4204178	up	XM_001065337	Zfx	367832
6.9045897	up	XM_001067896	Zhx2	314988
2.0530467	up	XM_001070925	Zkscan5	304275
2.4153066	up	XM_001054961	Zmiz1	361103
3.0045831	up	XM_001058926	Zmym1	313604
6.1081557	up	XM_001060431	Zmym2	305913
2.3825724	up	XM_001059117	Zmym6	362602
4.679734	up	NM_203366	Zmynd11	291259
10.502817	up	XM_001056023	Zmynd17	289904
6.658464	up	XM_001061666	Zmynd8	296374
4.262285	up	XM_001075472	Znf23	307839
2.0790136	up	XM_001056267	Znf295	304056
21.17016	up	XM_001070965	Znf394	252860
12.878758	up	XM_001075266	Znf496	287361
3.9448996	up	XM_001065659	Znf503	305687
2.3007612	up	XM_001062542	Znf509	305428
3.562413	up	XM_001059063	Znf511	293586
2.1507058	up	NM_001030038	Znf518a	309478
11.304102	up	XM_001073019	Znf521	307579
3.268796	up	XM_001064928	Znf532	307362
5.272477	up	XM_001061608	Znf592	293038
14.169117	up	XM_001056388	Znf598	287119
3.47173	up	XM_225755	Znf608	307296
2.2034714	up	XM_001078412	Znf688	293511
3.848863	up	XM_001075322	Znf692	303164
2.810872	up	XM_001053818	Znf746	312303
2.026794	up	XM_001053412	Znf777	502764
4.6991987	up	NM_001037360	Znf830	497967
2.4951434	up	XM_001071353	Znf839	500723
2.08352	up	NM_001047860	Znfx1	296384
2.1661386	up	XM_001081104	Znhit3	497975
6.277812	up	XM_001079278	Znhit6	292160
3.9868867	up	NM_213567	Znrd1	361784
5.1997795	up	XM_001060895	Znrf2	362367
11.685678	up	NM_031616	Zranb2	58821
5.732154	up	NM_001017504	Zrsr1	498425
2.3695376	up	NM_001012021	Zscan21	304342
2.8649735	up	XM_001070698	Zswim3	311630

6.2221537	up	XM_001067858	Zswim4	304655
14.969494	up	NM_001024801	Zw10	363059
2.1035545	up	NM_147138	Zwint	257644
3.5818927	up	XM_001072096	Zyx	114636

Control_vs_Normal 2.0 fold down regulated genes

Fold change	Regulation	ACCESSION	GeneSymbol	GeneID
8.299269	down	XM_574883	11-Mar	499558
3.2765787	down	NM_001012460	1-Sep	293507
20.701687	down	XM_001077761	6-Sep	691335
11.061232	down	NM_001025634	A2ld1	290500
4.162712	down	XM_001067255	Aaas	300259
2.16116	down	XM_001062198	Aadacl2	295076
3.8256989	down	NM_134398	Aagab	171435
2.251931	down	XM_001070331	Aak1	500244
2.18158	down	NM_145093	Aard	246323
3.7086017	down	XM_001067517	Aars2	301254
5.556882	down	NM_031003	Abat	81632
3.1010847	down	XM_001075753	Abca15	293442
2.0041337	down	XM_001054596	Abca3	302973
3.2180715	down	XM_001062082	Abcb5	314537
5.556858	down	NM_053754	Abcg5	114628
2.0560272	down	NM_001013118	Abi3	303476
10.646641	down	XM_001068096	Abl2	304883
2.0399144	down	XM_001072491	Abt1	306960
3.497746	down	NM_012489	Acaa1	24157
4.647921	down	NM_001034014	Accn1	25364
2.0124934	down	NM_024154	Accn2	79123
3.537833	down	NM_172009	Ache	83817
3.00379	down	NM_001080096	Acsbg2	301120
5.1431317	down	NM_144748	Acsm2	246263
12.444978	down	NM_001014162	Acsm5	361637
15.229722	down	XM_001074422	Actbl2	294732
11.09194	down	NM_022441	Acvrl1	25237
2.212402	down	XM_001055227	Acyp2	364224
5.585696	down	XM_001054670	Adam12	679837
4.514208	down	NM_020078	Adam1a	56777
16.890736	down	XM_228290	Adamts14	309837
2.6849713	down	XM_001060610	Adamts19	361332
5.355706	down	XM_341154	Adamts4	66015
8.593815	down	XM_001072537	Adamts6	361886
21.685356	down	XM_001078833	Adamtsl2	311827
2.0093038	down	XM_001075711	Adat1	690810

2.224755	down	NM_001012065	Adck4	308453
4.0972166	down	NM_144744	Adipoq	246253
3.3320558	down	XM_001057958	Adprhl2	362600
2.7144709	down	NM_138506	Adra2c	24175
2.9002564	down	XM_001069152	Aebp1	305494
9.012444	down	NM_080900	Afap1	140935
3.9030876	down	XM_001058822	Afap1l1	291565
13.431624	down	XM_001066299	Agap1	316611
4.6442437	down	XM_342881	Agbl4	362562
2.5638835	down	NM_053336	Ager	81722
3.0133858	down	XM_226709	Aggf1	310005
20.671062	down	XM_001070306	Agps	84114
3.059936	down	NM_175754	Agrn	25592
4.0440702	down	NM_013149	Ahr	25690
2.7656295	down	XM_001066198	Aifm3	303786
2.841165	down	XM_214790	Aig1	292486
2.6406074	down	XM_001069409	Ak7	314416
2.0208218	down	NM_022680	Akp3	64621
5.4280176	down	NM_138510	Akr1c18	171516
5.399938	down	NM_013215	Akr7a3	26760
2.0341518	down	XM_001077092	Alkbh4	288587
2.3836877	down	XM_001079600	Alox12	287454
2.0877938	down	NM_013059	Alpl	25586
21.456038	down	NM_012921	Alx1	25401
6.0937743	down	XM_341644	Amfr	361367
4.8943563	down	XM_001057982	Ammecr1l	307526
2.011514	down	NM_031502	Amy2	497039
2.3653278	down	XM_345278	Amy2-3	365914
4.0745707	down	NM_001047092	Amz1	304317
6.074704	down	NM_001012359	Ang	497229
13.082161	down	NM_001006992	Ang1	305843
16.494251	down	XM_216613	Angptl6	298698
4.4441013	down	XM_576558	Ankar	501138
7.5344677	down	XM_346238	Ankdd1a	367646
3.3529134	down	NM_001047901	Ankle2	360829
2.59251	down	XM_001068648	Ankrd13d	361699
8.260152	down	XM_574104	Ankrd29	498823
2.5736651	down	NM_198684	Ankrd36	305491
4.834202	down	XM_001061163	Ankrd37	361149
2.690907	down	XM_001070685	Ankrd52	362811
5.5059514	down	XM_342195	Ankrd55	361898
11.38182	down	XM_001053864	Ankrd57	503306
2.963331	down	XM_001067026	Anln	307056
4.282546	down	XM_235024	Ano4	299714

5.742238	down	XM_001057694	Ano6	315272
2.959141	down	XM_001071546	Anxa10	498622
2.8912454	down	NM_001031654	Anxa8	306283
2.378863	down	NM_023979	Apaf1	78963
5.566303	down	XM_001079912	Apob48r	499264
8.227828	down	NM_001085352	Apoc2	292697
2.1487882	down	XM_001075828	Apol2	315111
3.1063163	down	NM_173105	Aqp11	286758
2.083774	down	NM_001024742	Ard1b	289482
4.9276166	down	XM_001081375	Arhgap23	303501
2.2780907	down	XM_001070699	Arhgap25	500246
3.3548567	down	XM_001066829	Arhgap26	307459
2.6428225	down	XM_001053966	Arhgap30	498282
3.1895685	down	NM_144740	Arhgap4	246249
19.35832	down	XM_001061568	Arhgdig	360500
3.1870332	down	NM_001013246	Arhgef12	367072
2.2050524	down	XM_001067343	Arhgef17	308862
2.9885247	down	XM_001073085	Arhgef5	140898
2.0587955	down	XM_001055321	Arl13b	304037
10.047133	down	NM_212459	Arl9	289565
5.378963	down	XM_001060650	Armc10	296758
10.187062	down	XM_001071122	Armc6	306352
2.7928028	down	NM_001024367	Armcx1	501619
5.9338946	down	XM_001055969	Arrdc5	680452
6.135757	down	NM_001047885	Arse	310326
2.5327764	down	XM_001068810	Asb13	361268
3.8399632	down	XM_001081507	Asb16	498005
3.4502347	down	NM_017189	Asgr2	29403
14.278155	down	NM_144750	Aspg	246266
2.6322587	down	XM_001076577	Aspscr1	691026
5.841935	down	XM_230576	Astl	296129
3.8673043	down	XM_001068889	Atf7	315333
3.97827	down	NM_001038495	Atg12	361321
5.221645	down	NM_001012097	Atg7	312647
17.100565	down	NM_012508	Atp2b2	24215
2.6352043	down	XM_001055453	Atp6v1e2	366545
14.861855	down	XM_223390	Atp8a1	289615
2.4407587	down	NM_021702	Atxn3	60331
2.803544	down	XM_228583	Awat2	302425
4.364897	down	NM_024355	Axin2	29134
2.7616425	down	XM_001081778	Azi1	360672
7.1085277	down	XM_001057849	B3galt1	366064
3.1598358	down	XM_001069916	B3gnt8	308440
2.7924328	down	XM_342584	B4galt5	362275

4.198874	down	NM_001031661	B4galt7	364675
7.328861	down	NM_017300	Baat	29725
28.818754	down	XM_001062170	Bai2	313058
14.831034	down	NM_022300	Basp1	64160
3.9635012	down	XM_341141	Bat2d1	360865
5.6085877	down	XM_001057854	Batf	299206
9.944945	down	XM_001071505	Batf2	309178
13.840635	down	NM_021865	Batf3	60462
2.267492	down	XM_222315	Baz2a	304601
5.4291697	down	NM_173837	Bbc3	317673
2.5914855	down	XM_001063816	Bcdin3d	363001
17.71974	down	XM_001057750	Bcl11a	305589
2.3395865	down	NM_016993	Bcl2	24224
2.8451672	down	NM_001033670	Bcl2l1	24888
9.782247	down	NM_021850	Bcl2l2	60434
3.1659358	down	XM_001057993	Bcl3	680611
9.285501	down	XM_001059897	Bend4	681008
3.2299335	down	NM_001033674	Bicd2	306809
7.715339	down	NM_001012223	Bin2	366988
2.217785	down	XM_001070524	Bmp1	83470
8.168703	down	XM_001053727	Bmp7	85272
15.872786	down	XM_001057343	Bms1	362426
4.562021	down	XM_001071364	Bmx	367786
2.4397054	down	XM_001073216	Bola2	503208
2.086543	down	XM_001063756	Bpil1	296290
2.7178984	down	XM_343175	Brd4	362844
13.045282	down	XM_001078549	Brpf3	309647
3.3811467	down	XM_001071463	Brsk1	499073
15.5344515	down	XM_001075067	Brunol6	300758
21.868116	down	XM_001061326	Bsdc1	297890
36.48825	down	NM_019146	Bsn	29138
2.9397473	down	XM_576181	Btbd2	500793
2.704895	down	NM_001007798	Btk	367901
9.166404	down	NM_213630	Btla	407756
6.8687654	down	XM_001077286	Btnl4	294268
2.1384199	down	XM_001058059	C1d	289810
2.561373	down	XM_001066953	C1d-ps1	689062
3.5442595	down	NM_001007675	C1qtnf1	303701
16.616453	down	XM_001059843	C1qtnf3	294806
8.087147	down	XM_001077863	C1qtnf4	311184
8.2740135	down	NM_001002804	C1rl	408246
21.432966	down	NM_001025769	C20orf165	499943
5.4818335	down	XM_001054007	C7	117517
3.8198931	down	XM_001063411	Cab39	301574

5.575489	down	NM_133529	Cabp1	171051
5.835933	down	XM_001068353	Cabp4	365394
3.5200865	down	NM_001007730	Cabp7	360970
5.637156	down	NM_147141	Cacna1b	257648
10.025061	down	NM_153814	Cacna1h	114862
2.005575	down	NM_053851	Cacnb2	116600
2.7539914	down	NM_012828	Cacnb3	25297
3.7763941	down	NM_080691	Cacng3	140724
2.5443292	down	NM_080695	Cacng7	140728
2.6087694	down	NM_080696	Cacng8	140729
11.396112	down	NM_001047107	Cadm4	365216
3.224109	down	NM_053988	Calb2	117059
14.003207	down	NM_017338	Calca	24241
24.08758	down	NM_001012054	Calml3	307100
3.163344	down	NM_138915	Caly	192349
2.531107	down	NM_021678	Camk2n2	59314
2.3052492	down	NM_022383	Cap1	64185
3.0062163	down	NM_001013086	Capg	297339
6.255817	down	NM_031673	Capn10	63834
4.5025463	down	XM_001078527	Capn12	308476
5.764862	down	NM_133309	Capn8	170808
2.0999722	down	XM_001057555	Capsl	294795
2.9108298	down	XM_001075425	Car6	298657
3.0850904	down	XM_001076142	Card10	315120
9.1544895	down	XM_001054821	Card6	294770
2.2233658	down	XM_001076079	Carkd	361185
2.550848	down	NM_001034088	Carm1	363026
3.453919	down	NM_080690	Caskin1	140722
2.3775387	down	XM_001053920	Cass4	296409
10.116476	down	XM_342941	Catsper4	362623
2.3599904	down	XM_001055098	Cbr3	304078
2.8799236	down	NM_001008313	Cbx3	297093
17.530815	down	XM_001081757	Cbx4	501403
3.2552843	down	NM_001012119	Cbx6	315136
10.890511	down	XM_001058226	Ccdc102a	361363
3.890216	down	XM_001081527	Ccdc103	498006
6.7232814	down	XM_001059637	Ccdc106	499071
2.112581	down	XM_001053914	Ccdc11	364899
23.177656	down	XM_001080782	Ccdc114	308594
10.598474	down	XM_001062881	Ccdc121	289329
3.1429257	down	XM_574843	Ccdc125	499518
4.767837	down	NM_001024355	Ccdc134	500909
2.0077624	down	XM_001071774	Ccdc14	288054
4.3764577	down	NM_001013953	Ccdc153	300663

16.11274	down	XM_001059305	Ccdc154	287131
3.0320132	down	XM_001080694	Ccdc155	308581
18.569473	down	XM_001062522	Ccdc157	681091
9.610928	down	NM_001038992	Ccdc23	362578
13.258381	down	NM_001014091	Ccdc33	315712
2.1095173	down	XM_001055346	Ccdc36	680018
2.3600092	down	XM_001081764	Ccdc40	287867
3.6905572	down	XM_001079849	Ccdc42b	304509
4.367532	down	XM_001063933	Ccdc54	498073
17.719494	down	NM_001014087	Ccdc67	315438
2.7093804	down	XM_001058720	Ccdc74a	680752
8.964924	down	NM_001009533	Ccdc8	494320
6.822966	down	XM_001076332	Ccdc85c	690965
2.0480437	down	XM_001061924	Ccdc94	501285
9.6402	down	NM_001034138	Ccdc99	303037
30.179735	down	NM_012829	Cck	25298
5.461916	down	XM_001067590	Ccl1	688605
2.0946298	down	NM_019205	Ccl11	29397
2.0866163	down	NM_019233	Ccl20	29538
3.3791413	down	XM_001077331	Ccne1	25729
3.6122963	down	XM_001064132	Ccne2	362485
6.5424814	down	XM_001071133	Ccnf-ps1	689538
2.2081563	down	XM_001057184	Ccnj	294053
4.224866	down	XM_001059952	Ccnt1	315291
2.003735	down	NM_001037794	Cct8l1	499967
3.6786385	down	XM_001079863	Cd19	365367
26.394825	down	XM_001058705	Cd209a	288375
36.836327	down	XM_001064144	Cd209b	288378
7.4493856	down	NM_182824	Cd276	315716
12.53164	down	NM_001025111	Cd300lf	287818
3.0518348	down	NM_013169	Cd3d	25710
2.1569946	down	NM_012523	Cd53	24251
25.742844	down	NM_001025685	Cd5l	310693
2.639215	down	NM_175577	Cd6	25752
3.4575374	down	NM_020081	Cd86	56822
2.5622435	down	XM_001061998	Cd99	652929
2.6269422	down	NM_001013103	Cdc34	299602
14.578326	down	XM_001070494	Cdc42ep5	361505
24.106274	down	XM_001076282	Cdh3	116777
2.8703873	down	NM_053891	Cdk5r1	116671
2.2555594	down	XM_001068770	Cdk6	114483
2.990148	down	NM_130812	Cdkn2b	25164
2.330163	down	NM_001033860	Ceacam1	81613
7.406398	down	NM_173339	Ceacam10	287009

5.3920817	down	NM_012524	Cebpa	24252
3.2409277	down	NM_017095	Cebpe	25410
28.98266	down	XM_575656	Cecr6	500307
2.8401763	down	NM_001014163	Cend1	361675
3.4025748	down	NM_001017470	Cep70	367153
3.0239246	down	NM_133295	Ces3	113902
19.304834	down	XM_001071230	Cetn1	84592
2.386891	down	NM_212466	Cfb	294257
3.5028121	down	XM_001055310	Cfc1	501121
6.864637	down	NM_012525	Cgm4	24257
2.0587811	down	NM_198731	Chdh	290551
17.673725	down	NM_021655	Chga	24258
8.024045	down	XM_001055438	Chic1	363484
4.1265473	down	NM_001079689	Chit1	289032
2.496862	down	NM_001017466	Chmp4c	361916
3.3441768	down	NM_032084	Chn2	84031
78.549614	down	XM_001079984	Chpt1	362866
2.1687083	down	XM_001065852	Chrdl2	308854
14.89401	down	NM_031016	Chrm2	81645
4.0281825	down	NM_052805	Chrna3	25101
10.131892	down	XM_001079715	Chst11	314694
2.1307368	down	XM_001068489	Chst14	691394
12.250862	down	XM_001060886	Chsy3	291577
2.278175	down	XM_001060640	Chtf18	287146
6.53576	down	XM_001076412	Chtf8	364996
2.8402138	down	XM_001073489	Ckap2	306575
2.4718957	down	NM_001012738	Ckmt1	29593
2.435586	down	XM_579997	Cks1b	499655
43.97428	down	XM_001054024	Cks2	498709
4.6286526	down	XM_001074014	Clcn6	295586
2.089676	down	XM_001076934	Cldn15	304388
8.298775	down	NM_001008514	Cldn19	298487
2.0265808	down	XM_001054180	Cldn2	300920
7.747135	down	XM_001054648	Cldn20	680178
12.569667	down	XM_001059255	Cldn22	306454
8.655304	down	NM_001033062	Cldn23	290789
8.2770605	down	NM_001012022	Cldn4	304407
29.033245	down	NM_031702	Cldn7	65132
2.9851632	down	XM_213209	Clec16a	287044
2.3383348	down	XM_001067934	Clec1a	500337
17.26512	down	NM_130402	Clec2d	113937
6.0351114	down	NM_001048075	Clec2g	362447
2.595619	down	NM_001005891	Clec4a3	362431
7.483139	down	XM_578404	Clec7a	502902

26.640688	down	NM_001013080	Clic3	296566
2.732417	down	NM_176078	Clic6	304081
2.151052	down	NM_021997	Clip2	29264
2.0260732	down	NM_001006971	Cln3	293485
3.1252105	down	NM_013139	Clps	25680
19.633879	down	NM_001029914	Cmtm1	291823
2.22543	down	NM_001013142	Cmtm2a	307616
2.0914018	down	XM_001056147	Cmtm5	290214
5.28338	down	NM_053495	Cnga3	85257
6.1246114	down	NM_021686	Cnksr2	59322
2.2467544	down	NM_001012061	Cnksr3	308113
4.5457454	down	XM_001066271	Cnot3	308311
2.9730325	down	XM_341191	Cnot6l	360917
4.44901	down	NM_057118	Cntn1	117258
2.3424609	down	NM_012884	Cntn2	25356
18.951796	down	NM_013225	Cntn6	27256
2.7775228	down	XM_001076244	Cntnap4	307865
4.8578253	down	NM_001033889	Cog7	293456
2.3890898	down	XM_243912	Col12a1	25683
2.394312	down	XM_001056763	Col13a1	499431
4.2460704	down	XM_001062408	Col16a1	366474
8.815141	down	XM_001072793	Col22a1	315071
13.7988405	down	NM_181636	Col23a1	353303
8.480946	down	NM_198747	Col27a1	298101
3.6075737	down	NM_012929	Col2a1	25412
15.433117	down	XM_001053671	Col9a2	362584
24.587725	down	XM_001059028	Col9a3	362285
16.656199	down	XM_343396	Commd4	363068
2.6126611	down	NM_139108	Commd5	245974
3.0060852	down	NM_012834	Comp	25304
30.405672	down	XM_340887	Copz2	360611
5.651601	down	NM_001009671	Coq10b	301416
2.3028634	down	NM_053472	Cox4i2	84683
2.5616982	down	XM_001078870	Cp110	361634
3.0749836	down	NM_001013083	Cpa2	296959
2.7477913	down	XM_001074218	Cpe	25669
30.111963	down	NM_022864	Cplx1	64832
5.5099325	down	XM_001071808	Cplx3	501005
8.040199	down	XM_001080977	Cpm	314855
2.3220234	down	NM_001013963	Cpped1	302890
5.195426	down	XM_001075677	Cpt1c	308579
13.426609	down	XM_001081157	Cpxm1	296156
4.628455	down	NM_031766	Cpz	83575
2.5569458	down	NM_031017	Creb1	81646

4.9697714	down	NM_001007093	Creb3l4	310616
3.1700466	down	NM_001015027	Crebl2	362453
2.6222692	down	NM_001011710	Crisp2	360445
36.400333	down	XM_001072349	Crocc	313663
17.57273	down	NM_173140	Cryba2	286925
2.2978086	down	NM_173289	Cryge	24279
5.56247	down	NM_023981	Csf1	78965
2.4671004	down	XM_001074265	Csf2	116630
5.745023	down	NM_001037660	Csf2ra	652957
2.982604	down	NM_133652	Cspg5	50568
10.979818	down	NM_177425	Csrp2	29317
3.628869	down	NM_017074	Cth	24962
2.2885792	down	XM_232077	Ctnna2	297357
2.2630067	down	XM_001065193	Cts7	290970
5.001489	down	XM_001080387	Ctu1	292847
6.2676554	down	XM_001065365	Ctxn1	29145
2.2232158	down	XM_222184	Cux2	288665
2.701942	down	XM_001056781	Cwf19l1	365465
15.867582	down	NM_030845	Cxcl1	81503
24.686623	down	XM_342315	Cyb561d1	362017
2.7665493	down	XM_001073843	Cyp11b1	500892
2.7630951	down	NM_012538	Cyp11b2	24294
13.749363	down	XM_001070774	Cyp2b12	29295
3.610439	down	NM_031543	Cyp2e1	25086
3.1590538	down	NM_019303	Cyp2f4	54246
2.4424937	down	XM_001069311	Cyp46a1	362782
29.750189	down	XM_001074594	Cys1	690489
2.0926576	down	NM_001013980	Cyyr1	304138
5.9973583	down	XM_001068559	Dapl1	362136
2.5459535	down	XM_001058071	Dcbld1	309773
3.7499325	down	NM_130419	Dcbld2	155696
2.7066712	down	NM_138892	Dctpp1	192252
3.317049	down	XM_001068407	Dda1	688813
2.167988	down	XM_001077485	Ddx19a	292022
3.894907	down	XM_342582	Ddx27	362274
5.2021084	down	NM_171996	Decr2	64461
2.0205834	down	NM_001037503	Defb13	641626
25.92068	down	NM_001037504	Defb14	641627
4.0360346	down	NM_001037520	Defb15	641645
5.256269	down	NM_001037512	Defb21	641636
2.7476768	down	NM_022544	Defb4	64389
2.5650456	down	NM_001037511	Defb40	641637
6.8527966	down	XM_001056934	Dfna5	353316
16.607439	down	NM_001012146	Dgcr2	360742

2.750359	down	NM_019304	Dgkb	54248
3.0968237	down	NM_013126	Dgkg	25666
3.2419262	down	NM_001080148	Dhcr24	298298
6.0548897	down	XM_001076463	Dhdh	691002
6.169422	down	XM_001058220	Dhdpsl	293949
2.3363988	down	NM_017210	Dio3	29475
2.2049415	down	XM_001066151	Dis3l2	367307
12.821172	down	NM_001037654	Dixdc1	363062
6.7758555	down	XM_001077211	Dkk2	295445
2.111922	down	NM_138519	Dkk3	171548
2.3920505	down	XM_001080698	Dkkl1	499150
7.5365996	down	XM_001058879	Dlg5	305645
10.916248	down	NM_053744	Dlk1	114587
3.2594864	down	XM_001059233	Dlx1	296500
2.1305072	down	XM_001081314	Dlx4	303469
2.5498018	down	XM_233404	Dmbx1	313512
2.6229434	down	NM_001025288	Dmrtc1a	501668
4.912485	down	XM_001080801	Dnajc17	311329
2.4741287	down	XM_001056836	Dnajc6	313409
4.184282	down	NM_001003957	Dnmt3a	444984
4.4075227	down	NM_001011937	Doc2g	293654
5.939885	down	XM_001068649	Dock2	360509
6.7381663	down	NM_001025416	Dok1	312477
4.098395	down	NM_001008287	Donson	288257
4.276656	down	XM_001071372	Dot1l	362831
4.6214237	down	NM_001011928	Dpep2	291984
3.7377267	down	NM_001015007	Dph2	298452
2.1998708	down	XM_001061312	Dpp9	301130
7.1058497	down	XM_001063497	Dppa2	685364
21.854338	down	NM_001047864	Dppa3	297576
8.538472	down	NM_023023	Dpysl5	65208
2.2379718	down	XM_001055011	Dram	679937
3.7761781	down	XM_001053969	Dsc1	291759
2.0373554	down	NM_133587	Dscam	171119
7.318634	down	NM_199490	Dsg4	291754
2.382529	down	XM_001070652	Dtx2	304591
4.6603107	down	XM_001081001	Duoxa1	311374
2.1799474	down	XM_001060408	Dusp15	362238
6.4130483	down	XM_001070874	Dusp7	300980
22.894947	down	XM_224643	Duxbl	306226
2.9754574	down	XM_001059597	Dvl3	303811
8.722816	down	XM_214529	Dym	291433
17.348938	down	NM_001024767	Dyrk3	304775
4.1054854	down	NM_153303	Dyt1	266606

3.7711966	down	NM_001007010	Dyx1c1	363096
15.738486	down	NM_001009534	Eapa2	494321
4.506179	down	XM_001068246	Ebf2	361060
3.8173687	down	XM_001056705	Ebf3	361668
2.0700388	down	XM_001070886	Echdc3	684538
65.2443	down	XM_001064110	Ecop	362374
2.33015	down	XM_574055	Edaradd	498769
2.577789	down	XM_001080460	Eea1	314764
5.2135673	down	NM_001013972	Efcab3	303589
2.1120434	down	XM_574979	Efna3	170901
20.990429	down	XM_001079433	Efnb3	360546
2.3848312	down	XM_001056024	Efs	290212
9.038573	down	NM_001004070	Egfl8	406166
2.296704	down	NM_031599	Eif2ak3	29702
7.306846	down	XM_001063995	Eif5a2	310261
3.2337651	down	NM_001077651	Elavl4	432358
2.8659167	down	NM_001024768	Elf3	304815
8.189933	down	XM_001074045	Elfn1	288512
4.156267	down	XM_001059832	Elk4	304786
6.653345	down	XM_001076941	Elp4	691133
4.260834	down	XM_001069605	Emilin3	362262
6.1815815	down	XM_001069752	Eml1	362783
5.4086585	down	NM_138921	Eml2	192360
11.086223	down	XM_001064022	Eml5	444982
5.2193	down	NM_139325	Eno2	24334
3.5158587	down	XM_001080308	Enpep	64017
6.779991	down	XM_001065686	Enthd1	685900
3.3918953	down	NM_022587	Entpd1	64519
2.4496026	down	NM_172090	Epb4.1l1	59317
8.483817	down	NM_053927	Epb4.1l3	116724
4.3807197	down	XM_233522	Epha10	298528
7.7031646	down	XM_001069612	Ephb2	313633
8.297541	down	NM_021852	Epn2	60443
3.7284026	down	XM_001071045	Eps8l1	361503
3.617979	down	XM_001061258	Eps8l2	361674
2.6411521	down	NM_001044257	Epsti1	498547
4.4067245	down	NM_021687	Erbb4	59323
9.94295	down	XM_001077374	Ercc4l1	360465
3.0947804	down	XM_217387	Ercc5	301382
14.819641	down	XM_001077208	Erf	292721
7.123366	down	XM_001079523	Ern2	365363
3.9991803	down	NM_019622	Espn	56227
11.085952	down	NM_012754	Esr2	25149
5.7050667	down	XM_001079212	Etv2	361544

2.510813	down	NM_024147	Evl	79115
22.035696	down	XM_001073624	Extl2	310803
8.849673	down	XM_001077628	Fa2h	307855
2.5841484	down	NM_145878	Fabp5	140868
3.3408945	down	NM_017098	Fabp6	25440
3.4666529	down	NM_152937	Fadd	266610
2.2357361	down	NM_001025034	Fam107b	498796
3.253028	down	NM_001012348	Fam113a	296158
3.791264	down	XM_573600	Fam114a1	498366
2.6715755	down	XM_001056810	Fam114a1l1	316729
23.764214	down	XM_001081251	Fam117a	497983
13.583667	down	XM_343576	Fam117b	363236
6.019179	down	XM_001068090	FAM120C	317423
2.5924416	down	XM_001080165	Fam125b	362118
9.08209	down	XM_001076007	Fam131c	690880
3.3550284	down	XM_574844	Fam159b	499520
3.7741618	down	NM_001013876	Fam161a	289833
12.206794	down	XM_001062665	Fam163b	685169
2.3391495	down	NM_001014079	Fam164c	314321
2.0015473	down	XM_001067261	Fam168a	361614
31.712778	down	XM_001073605	Fam168b	690188
7.291968	down	XM_001059420	Fam169b	680906
12.617564	down	XM_001054549	Fam170a	498864
3.069267	down	XM_215438	Fam172a	294606
4.469436	down	NM_001009633	Fam175a	289468
2.6586232	down	XM_001058964	Fam184b	289671
33.506763	down	XM_001061851	Fam186a	684999
2.4927456	down	XM_001071695	Fam189b	310640
2.13344	down	XM_001058142	Fam19a2	680647
39.829407	down	XM_001066663	Fam47e	686140
3.4532125	down	XM_001054307	Fam53b	309060
4.0615644	down	XM_001074776	Fam60a	686611
52.502354	down	XM_001080097	Fam64a	360559
8.119119	down	XM_001061354	Fam69c	498891
2.978505	down	NM_182822	Fam70a	313453
27.379078	down	NM_001025031	Fam71b	497888
6.4529295	down	XM_001079857	Fam78a	499776
2.545409	down	XM_001079852	Fam83e	292913
12.856745	down	XM_001064041	Fam90a9	685497
2.3825936	down	NM_139194	Fas	246097
16.284864	down	NM_012908	Faslg	25385
3.5832815	down	XM_001078267	Fbln1	315191
2.2645037	down	XM_222254	Fbrsl1	304572
3.370569	down	NM_001025700	Fbxl12	313782

5.4873953	down	XM_001066947	Fbxl15	309453
8.35477	down	NM_001009504	Fbxl16	494223
6.448369	down	NM_053511	Fbxo2	85273
13.0588455	down	XM_341091	Fbxo21	360818
2.9783611	down	NM_001025642	Fbxo46	292686
3.0073562	down	XM_001075538	Fbxw12	301015
5.9303713	down	NM_001033924	Fcer2	171075
8.84156	down	XM_001066113	Fchsd1	307482
17.493586	down	XM_001058446	Fcrl3	680697
13.877909	down	XM_001076361	Fermt3	309186
6.792819	down	XM_001066375	Fes	361597
2.6241705	down	XM_001060067	Fezf2	305719
4.6964006	down	NM_020071	Fgb	24366
3.0906937	down	NM_139263	Fgd4	246174
15.267709	down	NM_012951	Fgf10	25443
2.331703	down	NM_130814	Fgf12	170630
14.683368	down	NM_021867	Fgf16	60464
2.2695982	down	NM_019199	Fgf18	29369
44.650818	down	NM_019305	Fgf2	54250
2.0544565	down	NM_053809	Fgf4	116499
2.7986188	down	NM_131908	Fgf6	170700
2.0745854	down	NM_022182	Fgf7	29348
3.6610868	down	XM_001077810	Fgfr2	25022
4.293002	down	NM_001044268	Fhad1	500577
2.3835807	down	NM_031677	Fhl2	63839
13.485502	down	XM_001079844	Fibcd1	311861
2.1779747	down	XM_575589	Figla	500239
2.6467535	down	NM_001014120	Fkbp10	360627
3.0530272	down	NM_001013105	Fkbp11	300211
3.7736354	down	NM_001013210	Fkbp14	362366
2.2688205	down	XM_001057312	Fkbp15	362528
5.006181	down	XM_001065788	Fkbp7	295672
2.0938075	down	NM_001025678	Fkrp	308390
19.00014	down	XM_001054999	Flg	24641
4.1972675	down	XM_001060505	Fndc5	260327
7.9142556	down	NM_012953	Fosl1	25445
3.473748	down	NM_012742	Foxa1	25098
4.449269	down	XM_001078067	Foxb2	691398
26.217428	down	XM_001079064	Foxc2	171356
2.4496083	down	XM_233422	Foxd2	313504
33.97038	down	XM_001055972	Foxd4	252886
7.648394	down	XM_001068320	Foxi3	502846
5.3086033	down	XM_001080864	Foxn1	287469
5.1497536	down	XM_001080414	Foxn4	288736

6.4873314	down	XM_001064339	Foxp4	363185
2.452566	down	NM_022858	Foxq1	64826
8.878513	down	XM_001078256	Fpgs	687266
7.0324125	down	XM_001058050	Fpr2l	681706
37.854576	down	XM_001064148	Frg2b	685524
5.1490593	down	XM_001071750	Frmd4a	307128
2.4779112	down	XM_001077268	Frmd4b	252858
5.488242	down	NM_001017382	Frs3	316213
2.2570195	down	NM_001004232	Fscn3	296947
6.5910773	down	XM_238588	Fsd1	301506
2.7324162	down	XM_001056728	Fsip2	680337
2.0910313	down	NM_024369	Fstl1	79210
2.153344	down	NM_001012014	Ftsj3	303608
3.3928618	down	NM_001012360	fut10	497619
4.0316267	down	NM_173308	Fut11	286971
4.471944	down	NM_031635	Fut2	58924
11.325842	down	NM_145717	Fxyd2	29639
6.025684	down	XM_001054436	Fyb	499537
2.5663202	down	NM_012755	Fyn	25150
2.1255345	down	NM_153474	Fzd3	266715
4.3849554	down	NM_173838	Fzd5	317674
6.324716	down	XM_001069570	Fzd7	301440
19.506899	down	NM_001009632	G0s2	289388
4.7947736	down	NM_053417	Gab2	84477
4.5736957	down	XM_001053889	Gabpb1l	364738
2.4889445	down	NM_017065	Gabrb3	24922
13.967577	down	NM_017291	Gabrr1	29694
23.187233	down	NM_138897	Gabrr3	192258
4.5564027	down	NM_080783	Gale	114860
85.693504	down	XM_001053341	Galntl1	362760
12.099077	down	XM_001061731	Galntl2	306268
2.094732	down	XM_001054513	Galntl6	361142
8.853339	down	XM_001066435	Gapt	686075
27.596952	down	XM_001068014	Gas2l1	360973
5.0831933	down	NM_033442	Gata2	25159
10.45043	down	NM_133293	Gata3	85471
2.5937562	down	NM_144730	Gata4	54254
2.9147847	down	NM_001013881	Gatad2a	290669
5.6130924	down	XM_001060894	Gbx1	246149
4.130013	down	NM_012565	Gck	24385
15.4753895	down	NM_001014211	Gcom1	363091
2.4187193	down	NM_021672	Gdf9	59304
4.523665	down	NM_012959	Gfra1	25454
7.420499	down	NM_001013065	Ggn	292765

4.7688837	down	NM_019235	Ggt5	29566
2.077402	down	XM_001081466	Ghdc	303542
14.719739	down	NM_001034849	Gimap1	312312
2.5553222	down	NM_145680	Gimap5	246774
11.67204	down	XM_001078890	Gins2	292058
2.1446757	down	NM_133563	Giot1	171090
2.2227073	down	NM_019630	Gip	25040
3.4484186	down	XM_001063402	Gja10	313126
25.671972	down	XM_001054584	Glipr2	679819
14.30508	down	XM_001066448	Glis1	298732
3.9701812	down	NM_022278	Glrx1	64045
2.9288938	down	XM_001065700	Glt25d1	290637
3.0763764	down	XM_001070927	Glt25d2	289081
5.7193117	down	XM_001054574	Gm672	364900
3.7626085	down	XM_001070048	Gmip	306357
23.604225	down	NM_021858	Gnb3	60449
6.3928566	down	NM_139185	Gng8	245986
20.580946	down	XM_001072812	Gnpnat1	498486
4.056334	down	XM_001081063	Gns	299825
7.314615	down	XM_001060893	Golga1	311919
5.8776684	down	NM_022596	Golga2	64528
3.660437	down	XM_001064007	Golsyn	500865
2.1633427	down	XM_001074030	Golt1b	362460
2.0364935	down	NM_053930	Gp1bb	116727
2.6644547	down	NM_001007013	Gphb5	366668
6.0785666	down	NM_181770	Gpr119	302813
6.514622	down	XM_001058657	Gpr123	309097
3.9412491	down	XM_001073056	Gpr132	314480
26.982615	down	XM_001064191	Gpr162	362436
5.197465	down	XM_001075182	Gpr172b	362942
4.80222	down	XM_001080723	Gpr176	117257
4.079625	down	XM_001054002	Gpr183	679975
7.8570023	down	NM_022216	Gpr20	60667
10.062853	down	NM_057201	Gpr37	117549
2.6262329	down	XM_001060511	Gpr45	301372
3.733049	down	XM_001055077	Gpr52	680192
4.306576	down	NM_152242	Gpr56	260326
2.3311088	down	XM_001066164	Gpr75	498434
3.1509044	down	NM_001003974	Gpsm3	406163
2.8865848	down	XM_574039	Gpx5	113919
2.7719367	down	XM_001060175	Gpx7	298376
3.3079958	down	NM_001034944	Grap2	366962
2.5206437	down	NM_138894	Grasp	192254
9.320332	down	NM_019282	Grem1	50566

13.1405945	down	XM_001068377	Grhl3	298555
4.054878	down	NM_057187	Grifin	117130
16.947485	down	NM_181373	Grik3	298521
6.9988503	down	NM_012572	Grik4	24406
7.068731	down	NM_017262	Grik5	24407
4.019495	down	NM_133308	Grin3b	170796
8.622385	down	XM_001053080	Grlf1-ps1	678709
13.552975	down	XM_001062001	Grm3	24416
11.6129875	down	XM_001071059	Grpcb	360395
4.512591	down	XM_001066936	Grrp1	500563
17.403383	down	NM_001013166	Gsg1	312793
2.3130774	down	XM_001057027	Gspt2	501582
3.9849594	down	NM_022284	Guca2b	64055
2.2102482	down	NM_153468	Gzma	266708
7.914151	down	NM_138517	Gzmb	171528
5.011134	down	NM_134332	Gzmc	171290
3.3050406	down	NM_153466	Gzmf	266704
9.522507	down	XM_001075299	H1fx	500252
4.020607	down	XM_001066891	H2afx	500987
19.010405	down	XM_001056152	H2afy2	361844
2.2635677	down	NM_053493	Hacl1	85255
5.405161	down	XM_001060358	Haghl	302995
14.558945	down	NM_022696	Hand2	64637
2.7162488	down	XM_001081256	Hao1	311446
6.014529	down	NM_001008559	Hapln3	308773
6.019471	down	NM_172323	Has1	282821
4.6778755	down	NM_001008890	Hbe1	293267
7.1709514	down	NM_001005900	Hcst	474146
3.3455818	down	NM_001025409	Hdac1	297893
2.8851902	down	XM_001073226	Hdac11	297453
6.315763	down	XM_001059685	Hdac1l	84576
4.0193663	down	XM_001054796	Hdac7	84582
14.37479	down	NM_133549	Hdgfl1	171074
24.879353	down	XM_001056925	Hdhd1a	291585
3.8685188	down	XM_001081620	Helz	287773
4.5081797	down	NM_001013179	Hes6	316626
4.9521627	down	XM_001079254	Hes7	287423
3.1509929	down	XM_001081535	Hexim2	303580
3.4200702	down	XM_342216	Hey1	155437
4.443264	down	XM_001058425	Hey2	155430
13.816539	down	XM_001055126	Heyl	313575
7.149685	down	XM_221303	Hic2	287940
3.44816	down	XM_001062714	Hist1h2ac	306962
9.220332	down	XM_001071473	Hist1h2ah	502125

6.4978766	down	XM_001061350	Hist1h2ai	502129
2.9503407	down	NM_001013056	Hist1h2ail	291159
3.169988	down	XM_001058007	Hist1h2ak	680615
2.9854267	down	XM_001061635	Hist1h2an	306970
5.1272893	down	XM_001061734	Hist1h2ao	364723
2.9052703	down	XM_001057941	Hist1h3g	680599
3.1353414	down	NM_022686	Hist1h4b	64627
2.13909	down	XM_001061900	Hist1h4m	291152
2.664784	down	XM_001061850	Hist2h2ac	499675
4.083617	down	XM_001059351	Hist2h2bb	295278
5.8065324	down	XM_001055051	Hist2h3c	679950
11.885053	down	XM_001062251	Hist2h3c2	310678
6.666092	down	XM_001062199	Hist2h4	295277
13.833487	down	NM_021840	Hist3h2a	64646
18.815746	down	XM_001053243	Hivep3	313557
18.534754	down	XM_001070255	Hmcn1	289094
2.7601879	down	XM_001067527	Hmcn2	686132
12.9447	down	NM_032070	Hmga2	84017
19.58648	down	XM_001054623	Hmgb2	29395
2.3303754	down	NM_001013184	Hmgn1	360704
2.9476204	down	XM_001073188	Hmha1	314618
24.987713	down	NM_012669	Hnf1a	24817
2.1095374	down	NM_152849	Homez	260325
2.3875277	down	XM_001054663	Hook1	313370
35.717987	down	NM_133621	Hopx	171160
23.526966	down	XM_575481	Hoxa13	500129
8.756385	down	NM_012581	Hoxa2	24452
8.719842	down	XM_220896	Hoxb1	303491
3.1937227	down	XM_001081336	Hoxb13	303480
3.1062312	down	XM_001081342	Hoxb3	303488
2.935354	down	XM_345881	Hoxc11	366996
14.687344	down	XM_001068426	Hoxc13	315337
2.5927312	down	XM_235702	Hoxc5	315341
3.460925	down	XM_001064329	Hoxd1	288151
4.8949127	down	XM_213633	Hoxd3	288152
35.720997	down	NM_017122	Hpca	29177
2.2419927	down	XM_001071788	Hs1bp3	313950
2.2444942	down	XM_001079125	Hs2st1	292155
3.3249786	down	XM_001061473	Hs3st5	294449
7.9657474	down	XM_001078574	Hs6st3	364476
2.6979325	down	NM_017080	Hsd11b1	25116
2.9235086	down	NM_001009684	Hsd17b13	305150
2.7145605	down	XM_001079257	Hsf2bp	499413
3.674394	down	XM_001063049	Hspa4l	294993

2.2268267	down	NM_139261	Hspbp1	246146
4.0864716	down	NM_012852	Htr1d	25323
3.59598	down	NM_024365	Htr6	64354
22.186857	down	XM_344185	Iars2	364070
17.834438	down	NM_012587	Ibsp	24477
4.913451	down	NM_013058	Id3	25585
7.0647554	down	XM_577595	Idi2	502143
2.4382696	down	NM_001025041	Ier5l	499772
8.62179	down	XM_001065459	Iffo1	362437
7.4445024	down	XM_001079914	Ifi44l	310968
15.645349	down	XM_001075191	Igdcc3	315759
2.5171053	down	XM_001076376	Igdcc4	363081
9.32524	down	NM_013122	Igfbp2	25662
4.2510934	down	NM_001004274	Igfbp4	360622
13.636884	down	XM_341798	Igfl3	361516
4.242663	down	NM_001013945	Ighg	299354
2.007353	down	XM_001066340	Igsf2	310727
3.2077398	down	XM_227520	Igsf3	295325
3.05018	down	XM_001058036	Ikbke	363984
3.4619627	down	NM_199103	Ikbkg	309295
6.1711802	down	XM_001054364	Ikzf2	301476
4.098453	down	NM_145789	Il13ra1	252963
14.173477	down	XM_001079675	Il17d	691799
20.298407	down	XM_001063391	Il17rb	306247
2.9918394	down	XM_001078782	Il17rc	297520
2.1053984	down	NM_053374	Il18bp	84388
19.59212	down	XM_001077078	Il1f5	311783
2.529716	down	XM_001077066	Il1f8	362076
3.0684068	down	NM_012968	Il1rap	25466
2.3235013	down	NM_177935	Il1rapl1	317553
2.6115758	down	NM_053836	Il2	116562
4.1228843	down	XM_001060362	Il20	681112
3.7071714	down	XM_576228	Il22	500836
2.1081553	down	NM_130410	Il23a	155140
3.6290896	down	NM_133380	Il4ra	25084
3.7242372	down	NM_012589	Il6	24498
16.272247	down	XM_001060982	Ildr1	303914
5.658124	down	XM_001055452	Inadl	313382
6.6257887	down	XM_001080838	Ino80	296084
3.9175978	down	NM_133562	Inpp5j	171088
7.9807816	down	NM_019130	Ins2	24506
4.544123	down	XM_001079190	Insm2	314131
4.8500843	down	NM_001047904	Ints6	361057
3.9978495	down	XM_001067002	Ints7	289382

2.1608407	down	XM_001056122	Invs	313228
11.514593	down	XM_226752	Ipo11	310056
3.0995722	down	XM_001073653	Iqce	304318
11.2973795	down	XM_001073634	Iqcf1	503227
10.046645	down	NM_001025153	Iqcf3	501050
3.1046987	down	NM_001008722	Irf8	292060
5.9799957	down	XM_001076309	Irs2	29376
3.8101485	down	NM_032074	Irs3	84021
6.366266	down	XM_001057357	Irx1	306659
2.587284	down	XM_001061997	Irx3	307721
2.121976	down	NM_001014242	Isoc1	364879
2.5822453	down	XM_001061626	Itfg3	360502
9.85307	down	XM_001070203	Itga5	315346
3.2309256	down	XM_001067562	Itgb7	25713
2.2902827	down	NM_001017505	Itgbl1	498564
8.420692	down	NM_017351	Itih3	50693
10.390746	down	XM_001058872	Itpk1	500709
17.669123	down	NM_031045	Itpka	81677
2.0200698	down	NM_031046	Itpr2	81678
6.9360538	down	NM_022195	Ivl	60583
2.4639213	down	NM_019147	Jag1	29146
2.5031683	down	XM_001073124	Jag2	29147
9.004467	down	NM_001003711	Jph4	445271
8.009029	down	NM_138875	Jund	24518
36.118233	down	NM_001008813	Kb23	407759
13.852383	down	XM_236723	Kbtbd5	316088
2.5858958	down	XM_001076969	Kbtbd8	500262
6.3321395	down	NM_173095	Kcna1	24520
2.1980379	down	NM_012972	Kcna5	25470
4.5248346	down	NM_017303	Kcnab1	29737
5.9871745	down	NM_139217	Kcnc2	246153
2.9916852	down	XM_001063865	Kcnd1	116695
2.151941	down	NM_133603	Kcne2	171138
2.1658273	down	NM_212526	Kcne4	367302
7.5976577	down	NM_032462	Kcnip3	65199
2.3508701	down	NM_133321	Kcnj15	170847
8.102123	down	NM_053806	Kcnk6	116491
5.416419	down	NM_053405	Kcnk9	84429
9.402325	down	NM_019273	Kcnmb1	29747
14.750075	down	XM_227040	Kcnmb3	310303
14.022831	down	NM_133322	Kcnq2	170848
23.871393	down	NM_021853	Kcnt1	60444
4.8100433	down	XM_001064164	Kcp	296952
4.327476	down	XM_001062225	Kctd1	291772

6.837943	down	XM_001077040	Kctd15	499129
5.088406	down	XM_001066967	Kctd3	305055
2.562041	down	NM_001025123	Kdelc2	315664
11.796368	down	XM_235478	Kdelr3	315131
7.997618	down	XM_001068749	Kdm2a	361700
5.926503	down	XM_001068791	Kel	297025
6.0912733	down	XM_001080530	Kera	314771
2.819424	down	XM_001069525	Khdc1b	689101
5.6477075	down	XM_001058042	Kif12	313254
5.8313036	down	NM_145877	Kif1c	113886
2.3190806	down	XM_001061764	Kif20a	361308
13.146506	down	XM_001073723	Kif23	315740
11.740578	down	XM_001061158	Kif26b	305012
3.0963805	down	XM_215883	Kif3b	296284
3.8548121	down	NM_198752	KIFC2	300053
2.8125086	down	NM_053394	Klf5	84410
3.379196	down	XM_001071689	Klf7	363243
7.3967466	down	XM_001055437	Klhl34	302703
4.6288347	down	XM_218953	Klhl35	308850
3.947486	down	NM_001005382	Klk1	292855
40.203278	down	NM_031523	Klk1b3	24594
66.81084	down	XM_001080426	Klk7	292852
4.0057735	down	NM_001013888	Klkbl4	291846
46.47709	down	NM_175759	Klks3	292868
6.8687243	down	NM_198746	Klra5	297666
2.603196	down	NM_001010964	Klrb1a	362443
3.8017027	down	NM_031649	Klrg1	58975
5.0606747	down	NM_139187	Klrh1	246043
2.9669492	down	NM_001004021	Krt13	287699
5.1571116	down	NM_001004022	Krt15	287700
9.667218	down	NM_053976	Krt18	294853
36.179276	down	NM_001004131	Krt24	287675
4.5314617	down	NM_001008822	Krt25	303519
2.999144	down	NM_001008824	Krt27	450229
4.144241	down	NM_001008758	Krt34	303528
17.252165	down	NM_001008809	Krt72	406227
14.925727	down	XM_001057910	Krt73	300248
3.5503294	down	XM_001060532	Krt83	681126
26.398386	down	XM_001055744	Krtap1-3	680104
24.913305	down	NM_001025135	Krtap1-5	497995
54.406693	down	XM_001056757	Krtap16-5	680703
50.936672	down	XM_001063382	Krtap19-3	685333
97.1236	down	XM_001063981	Krtap21-2	685485
50.113308	down	XM_577124	Krtap2-4	501720

2.2937143	down	XM_001057263	Krtap31-1	680454
38.97253	down	XM_001055517	Krtap3-3l1	680060
16.549788	down	XM_001056094	Krtap4-7	680195
30.835663	down	XM_001063784	Krtap5-5	685436
48.410484	down	XM_001072481	Krtap6-2	498056
19.835638	down	XM_001057319	Krtap9-5	680468
7.966753	down	XM_001065035	Lace1	502479
2.7727468	down	NM_212513	Lag3	297596
4.6477847	down	XM_001069930	Lamb3	305078
5.953803	down	XM_001079850	Lamc3	311862
2.2392075	down	NM_030853	Lat	81511
4.8473086	down	XM_218062	Lats1	308265
2.7585254	down	XM_001075793	Lbxcor1	315748
30.616093	down	XM_001066061	Lce1d	685981
37.91591	down	XM_001056303	Lce1f	499663
54.560337	down	XM_001066620	Lce1l	686125
40.59836	down	XM_001066389	Lce1s	686064
4.231949	down	NM_017266	Ldhc	29634
5.383965	down	XM_001067557	Ldlrap1	500564
15.269066	down	XM_001055597	Ldoc1	367956
3.9970977	down	XM_001078075	Ldoc1l	300114
2.9052196	down	NM_130429	Lef1	161452
4.638454	down	XM_001061810	Lefty1	498299
3.7127042	down	NM_053614	Lenep	113917
2.6039119	down	XM_001066214	Leng1	292535
3.3251677	down	NM_012976	Lgals5	25475
6.212136	down	NM_001009706	Lhpp	361663
3.7060726	down	XM_001079382	Lhx6	311901
9.159647	down	NM_022196	Lif	60584
7.231573	down	NM_031727	Limk1	65172
5.181989	down	NM_024135	Limk2	29524
2.0071	down	XM_001053331	Lims1	499443
10.384	down	XM_001067034	Lin28c	298542
5.636558	down	XM_001068291	Lingo1	315691
3.8210344	down	XM_001079892	Lipl3	309528
4.116048	down	NM_145790	Lipogenin	252964
5.7906632	down	XM_001072368	Lmln	363795
9.145774	down	XM_001076027	Lmnb2	299625
4.614557	down	XM_001062822	Lmod1	304816
3.3715806	down	XM_001072144	Lmx1a	289201
2.7081745	down	NM_147136	LOC257642	257642
5.095924	down	NM_147212	LOC259244	259244
11.020922	down	NM_147214	LOC259246	259246
76.629684	down	XM_001064280	LOC287921	287921

5.397456	down	NM_001047845	LOC287992	287992
20.908447	down	XM_001066324	LOC288419	288419
47.646233	down	XM_222037	LOC288574	288574
54.671227	down	XM_223807	LOC289918	289918
34.431328	down	XM_223906	LOC289977	289977
8.787532	down	XM_001074926	LOC290415	290415
24.772429	down	XM_001061314	LOC290508	290508
4.3548784	down	XM_001065661	LOC290595	290595
7.355594	down	XM_001062951	LOC291276	291276
12.227631	down	XM_225800	LOC291480	291480
36.609283	down	XM_225786	LOC291516	291516
2.0917075	down	XM_001054025	LOC291758	291758
5.0026727	down	NM_001013889	LOC291863	291863
2.9976764	down	NM_001013893	LOC292516	292516
31.728966	down	XM_001071880	LOC293697	293697
2.8218002	down	XM_001061024	LOC294446	294446
54.617657	down	XM_228268	LOC294497	294497
26.785362	down	XM_001068962	LOC295200	295200
12.984414	down	XM_001080231	LOC295528	295528
12.19084	down	XM_001081785	LOC295810	295810
5.77375	down	NM_001047859	LOC296300	296300
38.618946	down	XM_216072	LOC296778	296778
12.099913	down	XM_001059270	LOC298016	298016
6.2940135	down	NM_203512	LOC298109	298109
10.413017	down	NM_001024248	LOC298111	298111
5.46117	down	NM_001003409	LOC298116	298116
73.80514	down	XM_001065571	LOC298820	298820
7.5684013	down	XM_001061760	LOC299214	299214
8.067879	down	XM_001071400	LOC299915	299915
21.935684	down	XM_001073983	LOC300024	300024
6.5657816	down	XM_001063650	LOC300225	300225
3.922555	down	NM_001013952	LOC300308	300308
31.317795	down	XM_236873	LOC301193	301193
2.6822066	down	XM_001070601	LOC301289	301289
3.6147962	down	XM_001060850	LOC301379	301379
32.81259	down	XM_001073715	LOC301748	301748
36.180233	down	XM_229238	LOC301772	301772
34.306293	down	XM_229292	LOC301816	301816
54.848618	down	XM_001061753	LOC301839	301839
6.0871153	down	XM_229375	LOC301893	301893
45.049156	down	XM_217508	LOC301959	301959
53.387497	down	XM_217510	LOC301977	301977
33.119057	down	XM_229524	LOC302000	302000
13.900495	down	XM_001073811	LOC302022	302022

25.313652	down	XM_217530	LOC302085	302085
16.385357	down	XM_001081459	LOC302157	302157
49.933308	down	XM_001065392	LOC302192	302192
48.783268	down	XM_217542	LOC302199	302199
62.27303	down	XM_229842	LOC302228	302228
6.235931	down	XM_229908	LOC302282	302282
2.4253204	down	NM_001013962	LOC302845	302845
3.3739038	down	XM_001073573	LOC303140	303140
9.709272	down	NM_001017988	LOC303566	303566
2.9469543	down	NM_001006990	LOC304000	304000
6.278987	down	XM_001071596	LOC304239	304239
35.478786	down	XM_223806	LOC305698	305698
29.162956	down	XM_001081925	LOC305771	305771
8.35964	down	NM_001013993	LOC305806	305806
2.0692008	down	XM_001073815	LOC306096	306096
27.695354	down	XM_001065941	LOC306312	306312
33.04537	down	XM_224750	LOC306365	306365
37.88786	down	XM_001081637	LOC307706	307706
79.05432	down	XM_217963	LOC308198	308198
6.001076	down	XM_001073801	LOC312502	312502
50.159195	down	XM_236887	LOC316192	316192
29.196102	down	XM_001078812	LOC316493	316493
19.782467	down	XM_001081471	LOC316856	316856
58.300297	down	XM_001064910	LOC316873	316873
79.447174	down	XM_229488	LOC316919	316919
34.990723	down	XM_229505	LOC316931	316931
18.9407	down	XM_229508	LOC316933	316933
23.485727	down	XM_229514	LOC316935	316935
18.10336	down	XM_229775	LOC317070	317070
22.147703	down	XM_001078949	LOC317085	317085
28.50243	down	NM_001047892	LOC317165	317165
3.2362971	down	XM_001055457	LOC317546	317546
2.724282	down	XM_001059691	LOC317618	317618
16.187134	down	NM_001003706	LOC360228	360228
2.5272849	down	XM_340956	LOC360684	360684
50.89191	down	XM_001057404	LOC360998	360998
4.6817565	down	XM_341405	LOC361117	361117
17.488012	down	XM_001068100	LOC362347	362347
2.9171846	down	XM_001065062	LOC362710	362710
13.89541	down	NM_001008878	LOC362921	362921
38.4886	down	XM_001061883	LOC363181	363181
84.31829	down	XM_347120	LOC363301	363301
41.064148	down	XM_001062328	LOC363306	363306
62.630455	down	XM_343653	LOC363313	363313

34.666893	down	XM_001075455	LOC363320	363320
60.855377	down	XM_343663	LOC363324	363324
46.58918	down	XM_001060748	LOC363336	363336
99.97071	down	NM_001014221	LOC363337	363337
70.77789	down	XM_001081023	LOC363343	363343
49.445343	down	XM_001081888	LOC363351	363351
74.4476	down	XM_001076189	LOC363354	363354
52.330788	down	XM_001077951	LOC363363	363363
36.29742	down	XM_343700	LOC363366	363366
36.700314	down	XM_001074547	LOC363380	363380
68.19827	down	XM_343727	LOC363397	363397
49.782394	down	XM_001072586	LOC363405	363405
15.956937	down	XM_001081758	LOC363408	363408
67.35839	down	XM_343747	LOC363424	363424
65.56989	down	XM_343755	LOC363433	363433
41.290493	down	XM_343756	LOC363434	363434
5.7461357	down	XM_001056684	LOC363458	363458
9.318912	down	XM_001061625	LOC363645	363645
14.598273	down	XM_344092	LOC363894	363894
46.312874	down	XM_344247	LOC364164	364164
51.480583	down	XM_344294	LOC364250	364250
38.489822	down	XM_344313	LOC364281	364281
3.43735	down	XM_001053364	LOC364560	364560
5.995216	down	XM_001056850	LOC364653	364653
66.71507	down	XM_001067171	LOC364707	364707
5.020904	down	XM_001059009	LOC365076	365076
46.87522	down	XM_001053352	LOC365499	365499
48.82164	down	XM_001055159	LOC365501	365501
5.903428	down	XM_345058	LOC365502	365502
42.6632	down	XM_345061	LOC365506	365506
5.078224	down	XM_001077769	LOC365559	365559
7.3207607	down	NM_001014251	LOC365778	365778
6.752	down	XM_345306	LOC365969	365969
3.5607095	down	XM_001053873	LOC366369	366369
3.7635088	down	NM_001014264	LOC366772	366772
71.83227	down	XM_001070902	LOC366780	366780
3.24095	down	XM_001076470	LOC367036	367036
3.1853135	down	XM_345916	LOC367050	367050
13.562914	down	XM_001065443	LOC367269	367269
5.1754184	down	XM_001080734	LOC367277	367277
63.389614	down	XM_001078369	LOC367377	367377
56.86602	down	XM_001067423	LOC367381	367381
5.873363	down	XM_001068482	LOC367390	367390
49.55452	down	XM_346138	LOC367436	367436

56.937374	down	XM_001059570	LOC367485	367485
54.40452	down	XM_001075365	LOC367493	367493
73.13089	down	XM_001062392	LOC367516	367516
55.84053	down	XM_346193	LOC367523	367523
49.670025	down	XM_001063146	LOC367539	367539
18.67063	down	XM_001073778	LOC367597	367597
49.90691	down	XM_001081934	LOC367617	367617
19.400614	down	XM_001071580	LOC367619	367619
49.2415	down	XM_346234	LOC367632	367632
41.131454	down	XM_346241	LOC367653	367653
10.383574	down	XM_001065575	LOC367746	367746
2.44992	down	XM_001070043	LOC367960	367960
8.534635	down	XM_001077158	LOC367994	367994
98.06581	down	NM_001009977	LOC494539	494539
4.5898643	down	NM_001017476	LOC497940	497940
4.337418	down	XM_001071636	LOC498132	498132
2.4001164	down	NM_001017485	LOC498145	498145
2.628841	down	NM_001047923	LOC498152	498152
25.87609	down	XM_001068258	LOC498316	498316
65.6798	down	XM_573546	LOC498320	498320
3.7333462	down	NM_001017500	LOC498368	498368
40.14987	down	XM_001061548	LOC498372	498372
48.425095	down	XM_573609	LOC498374	498374
25.058294	down	XM_573699	LOC498444	498444
77.51499	down	XM_001079520	LOC498446	498446
54.967068	down	XM_001076556	LOC498463	498463
23.163778	down	XM_001069250	LOC498465	498465
38.140503	down	XM_573724	LOC498467	498467
32.35807	down	XM_573725	LOC498468	498468
31.567074	down	XM_001062043	LOC498470	498470
31.097637	down	XM_573728	LOC498471	498471
26.388048	down	XM_573730	LOC498473	498473
34.225624	down	XM_573731	LOC498474	498474
30.754328	down	XM_573735	LOC498477	498477
25.507704	down	XM_573738	LOC498480	498480
2.0517464	down	NM_001047932	LOC498525	498525
22.590673	down	XM_573840	LOC498565	498565
28.812107	down	XM_573955	LOC498672	498672
12.057077	down	NM_001047934	LOC498705	498705
56.168907	down	XM_001063340	LOC498781	498781
2.084799	down	XM_001061235	LOC498872	498872
13.701689	down	XM_574261	LOC498974	498974
3.3679075	down	XM_001077990	LOC499110	499110
58.844715	down	NM_001047938	LOC499219	499219

3.0223188	down	NM_001024287	LOC499240	499240
2.711534	down	NM_001024293	LOC499331	499331
55.388878	down	XM_579956	LOC499384	499384
5.49474	down	XM_574708	LOC499392	499392
8.962447	down	NM_001024296	LOC499418	499418
5.1969323	down	XM_001059085	LOC499618	499618
2.3368175	down	XM_001068073	LOC499729	499729
4.749247	down	XM_575069	LOC499735	499735
2.0358603	down	NM_001024309	LOC499749	499749
2.725816	down	XM_575114	LOC499781	499781
2.0756445	down	NM_001024311	LOC499806	499806
20.298588	down	NM_001047952	LOC499823	499823
4.1074724	down	XM_001075695	LOC499827	499827
4.2825265	down	NM_001024312	LOC499886	499886
3.8673208	down	NM_001047954	LOC500028	500028
3.3532336	down	NM_001047955	LOC500035	500035
4.357604	down	NM_001024325	LOC500077	500077
3.3022542	down	NM_001035253	LOC500148	500148
2.200718	down	XM_575564	LOC500213	500213
24.264557	down	NM_001047956	LOC500270	500270
5.95522	down	XM_001058924	LOC500420	500420
26.75008	down	XM_001077472	LOC500584	500584
2.7623198	down	XM_001063476	LOC500682	500682
11.884667	down	XM_576202	LOC500815	500815
17.733654	down	NM_001029926	LOC500893	500893
2.0852857	down	NM_001047965	LOC501038	501038
39.370865	down	XM_576504	LOC501089	501089
49.70811	down	XM_576506	LOC501091	501091
41.50523	down	XM_576507	LOC501092	501092
32.461784	down	XM_580172	LOC501210	501210
37.256493	down	XM_580174	LOC501212	501212
62.282063	down	XM_001067568	LOC501220	501220
57.768894	down	XM_576645	LOC501222	501222
50.40664	down	XM_001071000	LOC501223	501223
45.530228	down	XM_576649	LOC501226	501226
33.531048	down	XM_001076269	LOC501230	501230
2.7204716	down	NM_001047967	LOC501233	501233
74.534515	down	XM_576655	LOC501234	501234
72.67911	down	XM_576656	LOC501235	501235
36.328243	down	XM_580178	LOC501237	501237
52.67234	down	XM_576669	LOC501250	501250
61.580544	down	XM_576670	LOC501251	501251
29.867342	down	XM_576671	LOC501252	501252
44.989357	down	XM_576674	LOC501255	501255

47.22134	down	XM_576675	LOC501256	501256
5.4703984	down	XM_001074384	LOC501263	501263
11.20864	down	XM_001081788	LOC501270	501270
56.725456	down	XM_576687	LOC501271	501271
3.7174315	down	XM_001081100	LOC501272	501272
77.09637	down	XM_576689	LOC501274	501274
3.6362576	down	XM_576703	LOC501289	501289
4.341948	down	XM_580185	LOC501291	501291
79.066284	down	XM_576705	LOC501292	501292
82.09782	down	XM_001081221	LOC501296	501296
80.206535	down	XM_576712	LOC501299	501299
65.295586	down	XM_576715	LOC501302	501302
60.429276	down	XM_576718	LOC501305	501305
81.5156	down	XM_576719	LOC501306	501306
65.72291	down	XM_576720	LOC501307	501307
54.60504	down	XM_576721	LOC501308	501308
41.882526	down	XM_576725	LOC501312	501312
36.761806	down	XM_576728	LOC501315	501315
42.224422	down	XM_576729	LOC501317	501317
22.039133	down	XM_001068431	LOC501321	501321
41.509964	down	XM_001077981	LOC501325	501325
60.01675	down	XM_001059339	LOC501329	501329
98.19113	down	XM_001068278	LOC501333	501333
78.07093	down	XM_576745	LOC501334	501334
7.913709	down	XM_576746	LOC501335	501335
80.08177	down	XM_576750	LOC501339	501339
42.656647	down	XM_001081326	LOC501342	501342
65.337616	down	XM_576757	LOC501346	501346
20.543316	down	XM_001073636	LOC501349	501349
52.878555	down	XM_001077232	LOC501350	501350
3.1297772	down	XM_576764	LOC501353	501353
51.122845	down	XM_576770	LOC501358	501358
54.183525	down	XM_001075605	LOC501361	501361
67.64535	down	XM_001074895	LOC501362	501362
66.232155	down	XM_576779	LOC501366	501366
59.060158	down	XM_576781	LOC501368	501368
97.2149	down	XM_576782	LOC501369	501369
49.329563	down	XM_001081874	LOC501370	501370
42.3697	down	XM_001080720	LOC501372	501372
58.151516	down	XM_001069914	LOC501373	501373
33.168373	down	XM_576788	LOC501375	501375
36.56026	down	XM_576790	LOC501377	501377
42.9892	down	XM_001081029	LOC501383	501383
57.739212	down	XM_576797	LOC501384	501384

54.617477	down	XM_576799	LOC501386	501386
16.845032	down	XM_576801	LOC501388	501388
32.153854	down	XM_576802	LOC501389	501389
10.539881	down	XM_580189	LOC501391	501391
35.98629	down	XM_576808	LOC501396	501396
46.932518	down	XM_576812	LOC501399	501399
60.54399	down	XM_576813	LOC501400	501400
26.788948	down	XM_576828	LOC501417	501417
33.46265	down	XM_580192	LOC501420	501420
76.24212	down	XM_001058335	LOC501421	501421
3.5861003	down	XM_576832	LOC501422	501422
36.505405	down	XM_001078724	LOC501427	501427
21.215935	down	XM_001074315	LOC501437	501437
10.190827	down	XM_576866	LOC501456	501456
68.500404	down	XM_576877	LOC501467	501467
58.274464	down	XM_576879	LOC501469	501469
64.53245	down	XM_001066518	LOC501470	501470
51.4297	down	XM_576882	LOC501474	501474
35.368927	down	XM_001080724	LOC501476	501476
64.278305	down	XM_576885	LOC501477	501477
75.38921	down	XM_001081012	LOC501479	501479
60.38268	down	XM_001080503	LOC501482	501482
63.510117	down	XM_576890	LOC501483	501483
64.9935	down	XM_580199	LOC501485	501485
65.15481	down	XM_576894	LOC501488	501488
36.432842	down	XM_576897	LOC501491	501491
2.1161444	down	NM_001047112	LOC501503	501503
24.711481	down	XM_001066422	LOC501791	501791
19.864445	down	XM_001069885	LOC501799	501799
44.026287	down	XM_001070011	LOC501800	501800
53.69245	down	XM_001055287	LOC501842	501842
35.807655	down	XM_001081795	LOC501937	501937
14.595794	down	XM_577380	LOC501953	501953
2.4812622	down	XM_001063944	LOC502111	502111
7.697377	down	XM_001055993	LOC502167	502167
80.29056	down	XM_001071460	LOC502276	502276
57.724865	down	XM_001071332	LOC502279	502279
9.736459	down	XM_001077835	LOC502382	502382
59.81986	down	XM_577967	LOC502486	502486
32.155865	down	XM_001053637	LOC502549	502549
6.3431997	down	XM_001080659	LOC502572	502572
3.0464823	down	NM_001025064	LOC502894	502894
2.588255	down	XM_578409	LOC502907	502907
14.766846	down	XM_001055190	LOC502933	502933

28.558718	down	XM_001064499	LOC503152	503152
5.323973	down	XM_001071281	LOC503156	503156
3.3995025	down	XM_578715	LOC503192	503192
2.358313	down	XM_001067730	LOC503224	503224
43.427658	down	XM_578861	LOC503327	503327
4.0841746	down	XM_578871	LOC503337	503337
3.9662766	down	XM_001071495	LOC503366	503366
11.972129	down	XM_001067328	LOC503381	503381
3.6734514	down	XM_578925	LOC503388	503388
42.50232	down	XM_001076870	LOC503392	503392
3.7398708	down	XM_001072828	LOC503393	503393
8.044205	down	XM_001070645	LOC503407	503407
6.2922993	down	XM_001053009	LOC678700	678700
7.8380704	down	XM_001052993	LOC678702	678702
61.860386	down	XM_001053092	LOC678713	678713
23.545341	down	XM_001053098	LOC678714	678714
30.000948	down	XM_001053175	LOC678732	678732
48.3934	down	XM_001053285	LOC678755	678755
5.404641	down	XM_001053091	LOC678768	678768
20.515442	down	XM_001053190	LOC678790	678790
2.983803	down	XM_001053228	LOC678801	678801
2.788418	down	XM_001053238	LOC678805	678805
2.7414322	down	XM_001053279	LOC678812	678812
11.600071	down	XM_001053286	LOC678814	678814
10.652635	down	XM_001053442	LOC678841	678841
2.7459228	down	XM_001053785	LOC678893	678893
8.889208	down	XM_001053690	LOC678897	678897
3.6629314	down	XM_001054058	LOC678934	678934
10.51544	down	XM_001053931	LOC678948	678948
5.297027	down	XM_001053935	LOC678950	678950
72.25829	down	XM_001053936	LOC678951	678951
3.2999911	down	XM_001054033	LOC678966	678966
78.06979	down	XM_001054045	LOC678971	678971
88.14118	down	XM_001054164	LOC678999	678999
2.8394742	down	XM_001054295	LOC679024	679024
9.58112	down	XM_001054475	LOC679052	679052
66.312546	down	XM_001054541	LOC679066	679066
4.105487	down	XM_001054590	LOC679075	679075
2.0304298	down	XM_001054613	LOC679081	679081
6.9046307	down	XM_001054621	LOC679083	679083
2.1106317	down	XM_001054639	LOC679087	679087
14.997544	down	XM_001054758	LOC679116	679116
15.219467	down	XM_001054775	LOC679119	679119
3.3534632	down	XM_001054006	LOC679149	679149

44.176758	down	XM_001054917	LOC679152	679152
3.0962157	down	XM_001054441	LOC679159	679159
43.33089	down	XM_001055060	LOC679174	679174
3.1340888	down	XM_001055076	LOC679177	679177
6.2864933	down	XM_001055169	LOC679196	679196
21.06258	down	XM_001055262	LOC679220	679220
22.612465	down	XM_001055295	LOC679225	679225
2.9264631	down	XM_001055396	LOC679240	679240
2.6322908	down	XM_001055528	LOC679267	679267
29.498405	down	XM_001055566	LOC679276	679276
15.894373	down	XM_001055619	LOC679284	679284
6.631154	down	XM_001055683	LOC679296	679296
20.971912	down	XM_001055684	LOC679297	679297
2.1222107	down	XM_001055779	LOC679315	679315
32.570717	down	XM_001055874	LOC679336	679336
17.796152	down	XM_001055925	LOC679345	679345
6.2329392	down	XM_001055994	LOC679355	679355
30.91143	down	XM_001056071	LOC679373	679373
3.6487734	down	XM_001056286	LOC679424	679424
5.239325	down	XM_001056296	LOC679426	679426
14.817795	down	XM_001056304	LOC679430	679430
4.9114738	down	XM_001056318	LOC679434	679434
120.320885	down	XM_001056500	LOC679467	679467
5.2735105	down	XM_001056505	LOC679468	679468
51.609055	down	XM_001056539	LOC679474	679474
45.657265	down	XM_001056542	LOC679475	679475
5.006436	down	XM_001056556	LOC679478	679478
2.5450473	down	XM_001056611	LOC679495	679495
9.885952	down	XM_001053309	LOC679516	679516
5.397563	down	XM_001053356	LOC679525	679525
14.289302	down	XM_001053363	LOC679527	679527
3.4855673	down	XM_001053376	LOC679531	679531
7.7570686	down	XM_001053382	LOC679533	679533
2.9312336	down	XM_001053716	LOC679539	679539
31.282175	down	XM_001053426	LOC679546	679546
2.427537	down	XM_001053432	LOC679548	679548
18.252293	down	XM_001053447	LOC679551	679551
27.788486	down	XM_001053493	LOC679559	679559
37.914944	down	XM_001053499	LOC679562	679562
2.6707914	down	XM_001053570	LOC679578	679578
21.616371	down	XM_001053578	LOC679580	679580
34.309525	down	XM_001053601	LOC679587	679587
30.967909	down	XM_001053602	LOC679588	679588
4.406338	down	XM_001053687	LOC679601	679601

34.427746	down	XM_001053704	LOC679606	679606
73.20467	down	XM_001053708	LOC679608	679608
67.58584	down	XM_001053745	LOC679613	679613
43.152756	down	XM_001053751	LOC679615	679615
20.989014	down	XM_001053772	LOC679624	679624
11.094345	down	XM_001053810	LOC679635	679635
10.490124	down	XM_001053811	LOC679636	679636
55.301037	down	XM_001053817	LOC679639	679639
4.3769755	down	XM_001053854	LOC679646	679646
8.043753	down	XM_001053871	LOC679653	679653
52.794327	down	XM_001053909	LOC679662	679662
4.9591403	down	XM_001053910	LOC679663	679663
22.901371	down	XM_001053929	LOC679665	679665
2.9195824	down	XM_001054023	LOC679685	679685
11.121289	down	XM_001054071	LOC679694	679694
9.833316	down	XM_001054075	LOC679695	679695
56.43643	down	XM_001054076	LOC679696	679696
46.92979	down	XM_001054104	LOC679703	679703
3.032061	down	XM_001054134	LOC679708	679708
84.28407	down	XM_001054169	LOC679718	679718
41.95026	down	XM_001054170	LOC679719	679719
3.5372097	down	XM_001054194	LOC679721	679721
22.016186	down	XM_001054200	LOC679723	679723
48.07786	down	XM_001054202	LOC679724	679724
36.205616	down	XM_001054210	LOC679726	679726
46.20429	down	XM_001054232	LOC679730	679730
35.7607	down	XM_001054293	LOC679742	679742
56.03829	down	XM_001054303	LOC679745	679745
27.959135	down	XM_001054329	LOC679752	679752
4.579852	down	XM_001054337	LOC679756	679756
16.819729	down	XM_001054394	LOC679766	679766
4.2924685	down	XM_001054418	LOC679772	679772
81.076256	down	XM_001054477	LOC679792	679792
15.432025	down	XM_001054507	LOC679797	679797
42.623455	down	XM_001054511	LOC679798	679798
17.381884	down	XM_001054523	LOC679800	679800
89.93563	down	XM_001054539	LOC679807	679807
4.566302	down	XM_001054562	LOC679814	679814
13.339006	down	XM_001054575	LOC679817	679817
19.494324	down	XM_001053659	LOC679822	679822
10.109344	down	XM_001054610	LOC679825	679825
3.8788562	down	XM_001054617	LOC679826	679826
43.775078	down	XM_001054620	LOC679828	679828
15.017341	down	XM_001054637	LOC679832	679832

9.65233	down	XM_001054181	LOC679835	679835
10.804071	down	XM_001054681	LOC679839	679839
2.0526023	down	XM_001054690	LOC679843	679843
17.790236	down	XM_001054761	LOC679858	679858
48.78583	down	XM_001054768	LOC679861	679861
37.867855	down	XM_001054782	LOC679867	679867
40.78223	down	XM_001054810	LOC679878	679878
30.134045	down	XM_001054812	LOC679879	679879
9.388521	down	XM_001054867	LOC679891	679891
9.51941	down	XM_001054878	LOC679896	679896
15.154018	down	XM_001054886	LOC679901	679901
4.8491354	down	XM_001054892	LOC679902	679902
39.27926	down	XM_001054925	LOC679909	679909
51.67115	down	XM_001054988	LOC679927	679927
7.9497056	down	XM_001054996	LOC679931	679931
6.034984	down	XM_001055003	LOC679933	679933
41.94165	down	XM_001055005	LOC679935	679935
9.901363	down	XM_001055048	LOC679948	679948
10.111776	down	XM_001055069	LOC679954	679954
31.855618	down	XM_001054115	LOC679958	679958
11.546809	down	XM_001055105	LOC679964	679964
2.1677015	down	XM_001055107	LOC679966	679966
21.769081	down	XM_001055170	LOC679982	679982
2.0443957	down	XM_001055174	LOC679983	679983
7.050724	down	XM_001055218	LOC679992	679992
11.400962	down	XM_001055292	LOC680009	680009
66.074165	down	XM_001055300	LOC680010	680010
20.767387	down	XM_001055308	LOC680011	680011
50.282192	down	XM_001055404	LOC680029	680029
2.129526	down	XM_001055432	LOC680037	680037
23.5185	down	XM_001055521	LOC680062	680062
5.5608783	down	XM_001055524	LOC680063	680063
6.136398	down	XM_001055568	LOC680072	680072
2.2538528	down	XM_001055570	LOC680073	680073
2.5413573	down	XM_001055571	LOC680074	680074
80.23305	down	XM_001055573	LOC680075	680075
5.0519495	down	XM_001055577	LOC680077	680077
6.6390557	down	XM_001055746	LOC680105	680105
59.38656	down	XM_001055748	LOC680106	680106
48.566307	down	XM_001055754	LOC680108	680108
40.202988	down	XM_001055766	LOC680112	680112
16.706978	down	XM_001055804	LOC680120	680120
17.957325	down	XM_001055813	LOC680122	680122
76.2912	down	XM_001055864	LOC680136	680136

31.817432	down	XM_001055875	LOC680137	680137
62.085087	down	XM_001055919	LOC680154	680154
2.1357415	down	XM_001055658	LOC680160	680160
21.950697	down	XM_001055979	LOC680164	680164
3.7165704	down	XM_001055982	LOC680165	680165
54.6537	down	XM_001055983	LOC680166	680166
13.056774	down	XM_001055984	LOC680167	680167
3.4070501	down	XM_001056034	LOC680182	680182
82.19893	down	XM_001056044	LOC680185	680185
10.662138	down	XM_001056059	LOC680187	680187
27.711058	down	XM_001056064	LOC680189	680189
5.53208	down	XM_001056076	LOC680191	680191
34.885777	down	XM_001056098	LOC680198	680198
5.491178	down	XM_001056114	LOC680205	680205
3.6606913	down	XM_001056138	LOC680214	680214
5.2112	down	NM_001047974	LOC680227	680227
58.505043	down	XM_001056223	LOC680228	680228
58.89355	down	XM_001056232	LOC680230	680230
16.246264	down	XM_001056253	LOC680235	680235
24.489313	down	XM_001056256	LOC680236	680236
85.020676	down	XM_001056284	LOC680239	680239
13.598194	down	XM_001056294	LOC680242	680242
25.640368	down	XM_001056352	LOC680255	680255
2.3559642	down	XM_001056376	LOC680262	680262
17.038364	down	XM_001056408	LOC680272	680272
28.372545	down	XM_001056413	LOC680273	680273
16.525328	down	XM_001056418	LOC680277	680277
42.81023	down	XM_001056442	LOC680281	680281
7.020464	down	XM_001056463	LOC680286	680286
4.099851	down	XM_001056561	LOC680307	680307
13.607326	down	XM_001056586	LOC680310	680310
43.605404	down	XM_001056624	LOC680318	680318
65.68631	down	XM_001056660	LOC680323	680323
35.43944	down	XM_001056676	LOC680326	680326
3.3507862	down	XM_001056711	LOC680330	680330
15.961326	down	XM_001056719	LOC680334	680334
17.303646	down	XM_001056741	LOC680340	680340
24.473867	down	XM_001056745	LOC680341	680341
90.586586	down	XM_001056791	LOC680347	680347
38.578426	down	XM_001056978	LOC680387	680387
2.5158753	down	XM_001057030	LOC680396	680396
13.160196	down	XM_001057041	LOC680400	680400
28.448305	down	XM_001057042	LOC680401	680401
2.1557858	down	XM_001057119	LOC680426	680426

85.71498	down	XM_001057146	LOC680429	680429
52.012936	down	XM_001057154	LOC680431	680431
32.453876	down	XM_001057208	LOC680443	680443
63.904415	down	XM_001053549	LOC680453	680453
11.179648	down	XM_001057278	LOC680459	680459
85.79768	down	XM_001057350	LOC680475	680475
26.662613	down	XM_001057490	LOC680507	680507
2.4016733	down	XM_001057500	LOC680511	680511
23.46436	down	XM_001057558	LOC680525	680525
21.555656	down	XM_001057599	LOC680530	680530
31.0204	down	XM_001057668	LOC680547	680547
24.406586	down	XM_001057697	LOC680552	680552
35.060623	down	XM_001057734	LOC680563	680563
13.065753	down	XM_001057889	LOC680586	680586
24.200914	down	XM_001057937	LOC680596	680596
33.36841	down	XM_001057948	LOC680601	680601
52.188286	down	XM_001057966	LOC680608	680608
6.2284374	down	XM_001058078	LOC680631	680631
24.021774	down	XM_001058083	LOC680633	680633
72.990326	down	XM_001058086	LOC680636	680636
21.950829	down	XM_001058089	LOC680637	680637
30.160082	down	XM_001058120	LOC680643	680643
5.025041	down	XM_001058134	LOC680645	680645
15.789375	down	XM_001058172	LOC680650	680650
2.141346	down	XM_001058181	LOC680652	680652
27.686806	down	XM_001058184	LOC680653	680653
45.70478	down	XM_001058208	LOC680658	680658
7.3248897	down	XM_001058364	LOC680684	680684
2.1008434	down	XM_001058388	LOC680688	680688
5.0321097	down	XM_001058400	LOC680691	680691
3.5442913	down	XM_001056825	LOC680692	680692
4.691506	down	XM_001058504	LOC680704	680704
20.803848	down	XM_001058521	LOC680706	680706
11.096777	down	XM_001056892	LOC680711	680711
37.931664	down	XM_001058554	LOC680716	680716
39.20315	down	XM_001058607	LOC680730	680730
10.641303	down	XM_001058618	LOC680734	680734
2.1189418	down	XM_001058677	LOC680745	680745
57.652103	down	XM_001058696	LOC680748	680748
55.40358	down	XM_001058748	LOC680758	680758
100.96294	down	XM_001058749	LOC680759	680759
12.612502	down	XM_001058769	LOC680764	680764
3.7467499	down	XM_001058777	LOC680767	680767
28.622343	down	XM_001058789	LOC680771	680771

55.317196	down	XM_001058811	LOC680777	680777
7.759974	down	XM_001058892	LOC680789	680789
6.274562	down	XM_001058955	LOC680802	680802
2.9995723	down	XM_001058962	LOC680804	680804
3.754154	down	XM_001058971	LOC680807	680807
19.645037	down	XM_001058983	LOC680810	680810
31.258053	down	XM_001058989	LOC680812	680812
67.35011	down	XM_001059100	LOC680831	680831
3.225905	down	XM_001059174	LOC680848	680848
38.832687	down	XM_001059196	LOC680854	680854
7.378768	down	XM_001059256	LOC680870	680870
12.598732	down	XM_001059260	LOC680871	680871
4.828091	down	XM_001059262	LOC680872	680872
79.45127	down	XM_001059281	LOC680879	680879
19.63454	down	XM_001059315	LOC680886	680886
16.298923	down	XM_001059328	LOC680888	680888
7.533215	down	XM_001059362	LOC680893	680893
44.255955	down	XM_001059364	LOC680894	680894
22.32287	down	XM_001057692	LOC680905	680905
7.6211133	down	XM_001059444	LOC680917	680917
37.303253	down	XM_001059478	LOC680922	680922
4.5125175	down	XM_001059495	LOC680927	680927
2.1797585	down	XM_001059605	LOC680949	680949
26.54648	down	XM_001059609	LOC680950	680950
8.166848	down	XM_001059858	LOC681000	681000
18.920715	down	XM_001059901	LOC681010	681010
28.47294	down	XM_001059970	LOC681020	681020
37.035954	down	XM_001059994	LOC681027	681027
4.697706	down	XM_001060039	LOC681038	681038
39.89092	down	XM_001060083	LOC681047	681047
36.017025	down	XM_001060161	LOC681067	681067
38.558697	down	XM_001060180	LOC681069	681069
33.718067	down	XM_001060188	LOC681071	681071
84.4843	down	XM_001060331	LOC681105	681105
3.2886999	down	XM_001060359	LOC681110	681110
41.2691	down	XM_001060374	LOC681115	681115
19.733124	down	XM_001060390	LOC681122	681122
43.973003	down	XM_001060454	LOC681139	681139
8.067282	down	XM_001060483	LOC681146	681146
8.245278	down	XM_001060506	LOC681150	681150
68.96246	down	XM_001060513	LOC681151	681151
11.217131	down	XM_001060545	LOC681154	681154
19.82939	down	XM_001060556	LOC681160	681160
4.9637794	down	XM_001060565	LOC681164	681164

3.884197	down	XM_001060571	LOC681166	681166
43.78994	down	XM_001060628	LOC681176	681176
37.22562	down	XM_001059937	LOC681180	681180
39.10162	down	XM_001060675	LOC681187	681187
11.287025	down	XM_001060713	LOC681196	681196
47.320896	down	XM_001060741	LOC681204	681204
28.971521	down	XM_001060746	LOC681206	681206
2.6762145	down	XM_001060776	LOC681214	681214
34.52686	down	XM_001060797	LOC681220	681220
39.78892	down	XM_001060840	LOC681228	681228
42.607918	down	XM_001060847	LOC681230	681230
9.595362	down	XM_001060874	LOC681237	681237
38.742893	down	XM_001060969	LOC681259	681259
7.552107	down	XM_001061032	LOC681275	681275
46.330284	down	XM_001061073	LOC681283	681283
39.218327	down	XM_001061123	LOC681296	681296
54.47687	down	XM_001061128	LOC681299	681299
10.572636	down	XM_001061188	LOC681311	681311
2.1890028	down	XM_001061226	LOC681318	681318
11.155504	down	XM_001061236	LOC681322	681322
51.325264	down	XM_001061284	LOC681329	681329
28.836792	down	XM_001061295	LOC681332	681332
39.494003	down	XM_001061298	LOC681333	681333
26.947668	down	XM_001061311	LOC681335	681335
64.455475	down	XM_001061313	LOC681336	681336
45.547344	down	XM_001061340	LOC681341	681341
42.68419	down	XM_001061355	LOC681346	681346
10.3903	down	XM_001061364	LOC681350	681350
38.02511	down	XM_001061372	LOC681353	681353
2.4231074	down	XM_001061373	LOC681354	681354
21.329838	down	XM_001061464	LOC681372	681372
4.6621113	down	XM_001061496	LOC681382	681382
6.6339383	down	XM_001059639	LOC681383	681383
39.137306	down	XM_001061547	LOC681391	681391
55.382782	down	XM_001061602	LOC681405	681405
2.0835469	down	XM_001061633	LOC681411	681411
38.635033	down	XM_001061638	LOC681412	681412
2.3893917	down	XM_001056840	LOC681446	681446
4.68253	down	XM_001056910	LOC681457	681457
13.977492	down	XM_001056987	LOC681471	681471
43.115944	down	XM_001057059	LOC681486	681486
37.329353	down	XM_001057300	LOC681537	681537
4.165686	down	XM_001057383	LOC681552	681552
28.271978	down	XM_001057478	LOC681575	681575

30.19091	down	XM_001057495	LOC681582	681582
10.213049	down	XM_001057510	LOC681587	681587
6.8896947	down	XM_001057623	LOC681610	681610
3.111232	down	XM_001057644	LOC681616	681616
18.927752	down	XM_001057652	LOC681620	681620
11.719363	down	XM_001057676	LOC681626	681626
4.439578	down	XM_001057732	LOC681643	681643
5.622571	down	XM_001057737	LOC681644	681644
41.202698	down	XM_001057766	LOC681652	681652
66.30763	down	XM_001057769	LOC681653	681653
2.255575	down	XM_001057802	LOC681664	681664
5.5393624	down	XM_001057939	LOC681685	681685
60.799988	down	XM_001058241	LOC681743	681743
2.9743376	down	XM_001058322	LOC681765	681765
57.75536	down	XM_001058327	LOC681766	681766
14.356676	down	XM_001058573	LOC681819	681819
12.298655	down	XM_001058665	LOC681839	681839
7.493009	down	XM_001058688	LOC681847	681847
6.2849956	down	XM_001058855	LOC681893	681893
30.134901	down	XM_001059139	LOC681968	681968
7.967875	down	XM_001059190	LOC681979	681979
2.4615016	down	XM_001059425	LOC682020	682020
2.7057428	down	XM_001059486	LOC682033	682033
25.532164	down	XM_001059517	LOC682039	682039
23.11028	down	XM_001059537	LOC682041	682041
5.867802	down	XM_001059739	LOC682078	682078
18.866392	down	XM_001059853	LOC682102	682102
50.739475	down	XM_001060368	LOC682197	682197
56.777546	down	XM_001060373	LOC682199	682199
5.1734924	down	XM_001060429	LOC682208	682208
70.90834	down	XM_001060443	LOC682210	682210
9.913683	down	XM_001060490	LOC682216	682216
4.5190907	down	XM_001060537	LOC682225	682225
11.509122	down	XM_001060538	LOC682226	682226
37.326847	down	XM_001060609	LOC682238	682238
9.405426	down	XM_001060768	LOC682270	682270
33.190823	down	XM_001060854	LOC682289	682289
2.8346028	down	XM_001060911	LOC682301	682301
43.040382	down	XM_001060950	LOC682310	682310
20.774624	down	XM_001061095	LOC682341	682341
2.0663476	down	XM_001061167	LOC682355	682355
12.020871	down	XM_001061186	LOC682360	682360
7.6646633	down	XM_001061422	LOC682416	682416
2.0271149	down	XM_001061522	LOC682438	682438

21.595106	down	XM_001061593	LOC682458	682458
2.6184757	down	XM_001061705	LOC682487	682487
51.524536	down	XM_001061832	LOC682515	682515
3.1431513	down	XM_001061917	LOC682535	682535
11.765349	down	XM_001061959	LOC682543	682543
47.81573	down	XM_001062011	LOC682556	682556
3.564282	down	XM_001062026	LOC682560	682560
54.02105	down	XM_001062148	LOC682585	682585
6.5398784	down	XM_001062158	LOC682587	682587
29.587152	down	XM_001062253	LOC682603	682603
38.923714	down	XM_001062440	LOC682639	682639
7.004849	down	XM_001062475	LOC682647	682647
7.9182744	down	XM_001062486	LOC682649	682649
4.881095	down	XM_001062593	LOC682679	682679
30.80606	down	XM_001062679	LOC682695	682695
11.699973	down	XM_001062684	LOC682696	682696
33.05187	down	XM_001062715	LOC682701	682701
6.977499	down	XM_001062737	LOC682706	682706
48.236073	down	XM_001062936	LOC682754	682754
7.001122	down	XM_001062944	LOC682757	682757
21.755365	down	XM_001063036	LOC682774	682774
2.3642352	down	XM_001063068	LOC682778	682778
48.861904	down	XM_001063145	LOC682796	682796
7.818471	down	XM_001063184	LOC682803	682803
50.807777	down	XM_001063233	LOC682815	682815
2.2244785	down	XM_001063311	LOC682831	682831
9.265849	down	XM_001063412	LOC682856	682856
49.438286	down	XM_001063439	LOC682858	682858
37.774178	down	XM_001063448	LOC682859	682859
2.9351504	down	XM_001061205	LOC682869	682869
36.393497	down	XM_001063558	LOC682885	682885
34.228928	down	XM_001063578	LOC682891	682891
4.7058043	down	XM_001063627	LOC682906	682906
27.652246	down	XM_001063744	LOC682932	682932
5.3663554	down	XM_001061259	LOC682937	682937
10.005081	down	XM_001060862	LOC682938	682938
10.168259	down	XM_001063846	LOC682956	682956
2.5007684	down	XM_001063879	LOC682964	682964
14.833642	down	XM_001063891	LOC682966	682966
29.729624	down	XM_001063918	LOC682972	682972
38.663033	down	XM_001064451	LOC682986	682986
4.246557	down	XM_001065118	LOC682988	682988
45.990013	down	XM_001064012	LOC682996	682996
28.463457	down	XM_001064138	LOC683020	683020

3.4677138	down	XM_001062345	LOC683033	683033
3.5774925	down	XM_001061331	LOC683034	683034
6.9583635	down	XM_001064196	LOC683035	683035
13.916722	down	XM_001064254	LOC683045	683045
67.66212	down	XM_001064336	LOC683070	683070
3.853579	down	XM_001058171	LOC683077	683077
16.51926	down	XM_001064410	LOC683086	683086
2.926908	down	XM_001062927	LOC683099	683099
16.337212	down	XM_001064639	LOC683138	683138
10.782382	down	XM_001064668	LOC683146	683146
2.6641095	down	XM_001064715	LOC683158	683158
46.181953	down	XM_001064734	LOC683166	683166
47.96582	down	XM_001064818	LOC683184	683184
30.343609	down	XM_001064924	LOC683210	683210
6.4867268	down	XM_001064967	LOC683219	683219
12.815384	down	XM_001064973	LOC683221	683221
2.9230266	down	XM_001064980	LOC683223	683223
56.90545	down	XM_001065020	LOC683233	683233
25.104734	down	XM_001065323	LOC683308	683308
10.347362	down	XM_001065424	LOC683324	683324
2.0923808	down	XM_001065432	LOC683326	683326
4.7019095	down	XM_001070523	LOC683342	683342
100.02085	down	XM_001065651	LOC683369	683369
2.4604514	down	XM_001065716	LOC683389	683389
12.4439535	down	XM_001065347	LOC683460	683460
3.2430258	down	XM_001066042	LOC683463	683463
20.155195	down	XM_001066056	LOC683469	683469
5.650062	down	XM_001066080	LOC683472	683472
3.2296836	down	XM_001061872	LOC683474	683474
2.6640673	down	XM_001066111	LOC683479	683479
23.033321	down	XM_001066162	LOC683490	683490
113.04247	down	XM_001066243	LOC683507	683507
14.306933	down	XM_001066437	LOC683539	683539
3.9518266	down	XM_001066476	LOC683548	683548
4.7253833	down	XM_001066521	LOC683561	683561
5.49478	down	XM_001066537	LOC683564	683564
6.235927	down	XM_001066659	LOC683588	683588
27.647558	down	XM_001066697	LOC683601	683601
13.337028	down	XM_001066822	LOC683633	683633
40.11196	down	XM_001066890	LOC683649	683649
2.0007696	down	XM_001067070	LOC683689	683689
28.0463	down	XM_001067117	LOC683698	683698
10.066568	down	XM_001067123	LOC683700	683700
5.3299875	down	XM_001064064	LOC683722	683722

5.856394	down	XM_001067284	LOC683741	683741
9.150478	down	XM_001067289	LOC683743	683743
2.2051275	down	XM_001067331	LOC683751	683751
2.4873846	down	XM_001067334	LOC683753	683753
3.190623	down	XM_001064232	LOC683788	683788
64.14812	down	XM_001067513	LOC683800	683800
40.65556	down	XM_001067561	LOC683806	683806
15.132651	down	XM_001067758	LOC683852	683852
9.775224	down	XM_001067774	LOC683855	683855
23.879774	down	XM_001067816	LOC683865	683865
57.00516	down	XM_001067901	LOC683878	683878
11.180309	down	XM_001067987	LOC683902	683902
10.036413	down	XM_001068012	LOC683908	683908
7.3334312	down	XM_001068058	LOC683919	683919
2.037515	down	XM_001068062	LOC683921	683921
72.11933	down	XM_001068139	LOC683944	683944
4.444441	down	XM_001059945	LOC683953	683953
43.155766	down	XM_001068219	LOC683958	683958
5.4214354	down	XM_001068242	LOC683965	683965
27.733185	down	XM_001068264	LOC683970	683970
84.12614	down	XM_001068277	LOC683973	683973
7.3764906	down	XM_001068380	LOC683990	683990
39.369003	down	XM_001068387	LOC683992	683992
21.59761	down	XM_001068467	LOC684009	684009
6.16242	down	XM_001068831	LOC684077	684077
31.072342	down	XM_001068852	LOC684083	684083
43.43878	down	XM_001068871	LOC684087	684087
2.8977213	down	XM_001068952	LOC684105	684105
20.854448	down	XM_001069018	LOC684119	684119
2.3398454	down	XM_001069045	LOC684125	684125
34.748844	down	XM_001069050	LOC684128	684128
31.510792	down	XM_001069080	LOC684134	684134
11.799877	down	XM_001069099	LOC684138	684138
14.921711	down	XM_001069294	LOC684180	684180
3.643424	down	XM_001069416	LOC684209	684209
12.459144	down	XM_001069434	LOC684215	684215
13.299426	down	XM_001069592	LOC684254	684254
2.99279	down	XM_001069706	LOC684280	684280
6.4510317	down	XM_001069722	LOC684285	684285
67.63424	down	XM_001069803	LOC684306	684306
84.69002	down	XM_001069913	LOC684337	684337
16.190151	down	XM_001070181	LOC684393	684393
35.31649	down	XM_001070572	LOC684471	684471
75.3441	down	XM_001070790	LOC684515	684515

4.1310687	down	XM_001070830	LOC684525	684525
2.346958	down	XM_001070844	LOC684529	684529
2.1879547	down	XM_001070960	LOC684556	684556
20.108915	down	XM_001070974	LOC684559	684559
38.101532	down	XM_001071070	LOC684575	684575
45.4884	down	XM_001071142	LOC684587	684587
6.764408	down	XM_001071212	LOC684605	684605
37.83255	down	XM_001071307	LOC684627	684627
84.88739	down	XM_001055278	LOC684652	684652
10.947163	down	XM_001071489	LOC684660	684660
2.593608	down	XM_001071632	LOC684692	684692
49.30169	down	XM_001071663	LOC684706	684706
2.4317136	down	XM_001071688	LOC684715	684715
6.534451	down	XM_001071690	LOC684716	684716
65.99255	down	XM_001071746	LOC684725	684725
14.090421	down	XM_001071766	LOC684730	684730
60.237682	down	XM_001071819	LOC684746	684746
5.7560277	down	XM_001071837	LOC684753	684753
13.760056	down	XM_001071876	LOC684769	684769
16.058575	down	XM_001071887	LOC684773	684773
30.943558	down	XM_001071888	LOC684774	684774
5.9639482	down	XM_001071901	LOC684776	684776
66.17719	down	XM_001071914	LOC684779	684779
16.960512	down	XM_001071929	LOC684782	684782
30.764902	down	XM_001071939	LOC684787	684787
4.0450435	down	XM_001071963	LOC684792	684792
6.0569754	down	XM_001071973	LOC684798	684798
36.072422	down	XM_001072000	LOC684803	684803
5.723761	down	XM_001072050	LOC684819	684819
4.9646444	down	XM_001072065	LOC684822	684822
50.601326	down	XM_001072070	LOC684824	684824
60.95214	down	XM_001072084	LOC684827	684827
2.030189	down	XM_001072089	LOC684828	684828
44.89923	down	XM_001072117	LOC684833	684833
7.8520985	down	XM_001072148	LOC684840	684840
17.18831	down	XM_001072184	LOC684850	684850
15.348039	down	XM_001072236	LOC684864	684864
2.3679929	down	XM_001072266	LOC684871	684871
7.9215126	down	XM_001072267	LOC684872	684872
12.434995	down	XM_001072294	LOC684879	684879
4.041843	down	XM_001072338	LOC684888	684888
27.724388	down	XM_001072371	LOC684897	684897
21.871195	down	XM_001072377	LOC684898	684898
2.6448288	down	XM_001072410	LOC684907	684907

57.59253	down	XM_001072445	LOC684915	684915
2.2573156	down	XM_001072630	LOC684947	684947
5.1243143	down	XM_001061754	LOC684978	684978
31.291628	down	XM_001061782	LOC684986	684986
12.488226	down	XM_001061793	LOC684989	684989
72.58083	down	XM_001061802	LOC684990	684990
23.616804	down	XM_001061833	LOC684993	684993
26.286036	down	XM_001061848	LOC684998	684998
7.7777634	down	XM_001061886	LOC685007	685007
46.8872	down	XM_001061927	LOC685018	685018
34.706654	down	XM_001061948	LOC685019	685019
7.3976684	down	XM_001062010	LOC685030	685030
2.9894662	down	XM_001062044	LOC685040	685040
60.36003	down	XM_001057025	LOC685044	685044
11.288925	down	XM_001062057	LOC685046	685046
61.207943	down	XM_001062073	LOC685053	685053
6.1912713	down	XM_001062123	LOC685060	685060
17.821451	down	XM_001062130	LOC685064	685064
46.20162	down	XM_001062150	LOC685071	685071
34.295967	down	XM_001062177	LOC685075	685075
7.992132	down	XM_001062252	LOC685089	685089
35.164825	down	XM_001062311	LOC685105	685105
3.9626734	down	XM_001062332	LOC685109	685109
48.328423	down	XM_001063598	LOC685110	685110
6.0094266	down	XM_001062388	LOC685120	685120
3.1492083	down	XM_001062416	LOC685124	685124
49.84199	down	XM_001062428	LOC685125	685125
2.322914	down	XM_001062604	LOC685160	685160
20.605654	down	XM_001062622	LOC685162	685162
25.02738	down	XM_001062656	LOC685166	685166
33.190987	down	XM_001062681	LOC685176	685176
6.6639156	down	XM_001058763	LOC685178	685178
35.395756	down	XM_001062718	LOC685183	685183
40.94081	down	XM_001062796	LOC685205	685205
4.301106	down	XM_001062816	LOC685208	685208
20.152956	down	XM_001062826	LOC685210	685210
46.46998	down	XM_001062892	LOC685226	685226
12.612435	down	XM_001062910	LOC685231	685231
31.001274	down	XM_001062972	LOC685242	685242
2.6635866	down	XM_001060017	LOC685244	685244
27.31596	down	XM_001063120	LOC685275	685275
49.475063	down	XM_001057196	LOC685285	685285
35.7738	down	XM_001063186	LOC685291	685291
55.32943	down	XM_001057314	LOC685300	685300

65.62106	down	XM_001063228	LOC685303	685303
29.332785	down	XM_001063242	LOC685306	685306
144.83769	down	XM_001063338	LOC685323	685323
4.2195783	down	XM_001057424	LOC685331	685331
17.598925	down	XM_001063413	LOC685340	685340
6.392415	down	XM_001063414	LOC685341	685341
81.50776	down	XM_001057537	LOC685344	685344
56.178562	down	XM_001063434	LOC685347	685347
48.813026	down	XM_001063435	LOC685348	685348
43.74813	down	XM_001063442	LOC685349	685349
65.107124	down	XM_001063496	LOC685363	685363
31.594494	down	XM_001063504	LOC685366	685366
63.550095	down	XM_001063513	LOC685369	685369
26.356415	down	XM_001063529	LOC685371	685371
5.7822213	down	XM_001063533	LOC685372	685372
8.195505	down	XM_001063547	LOC685376	685376
44.309963	down	XM_001063548	LOC685377	685377
55.997963	down	XM_001063606	LOC685392	685392
6.0262136	down	XM_001063633	LOC685397	685397
99.70129	down	XM_001063655	LOC685403	685403
33.09599	down	XM_001063692	LOC685411	685411
21.525541	down	XM_001063696	LOC685414	685414
60.34457	down	XM_001063747	LOC685424	685424
10.3155775	down	XM_001063765	LOC685428	685428
46.817806	down	XM_001063769	LOC685429	685429
67.85382	down	XM_001057638	LOC685435	685435
41.43775	down	XM_001063788	LOC685437	685437
64.71326	down	XM_001063799	LOC685442	685442
2.9300306	down	XM_001057762	LOC685449	685449
2.852654	down	XM_001063855	LOC685454	685454
7.066689	down	XM_001063875	LOC685458	685458
47.909035	down	XM_001063877	LOC685459	685459
37.411713	down	XM_001063881	LOC685460	685460
23.41377	down	XM_001063912	LOC685469	685469
42.076637	down	XM_001063931	LOC685476	685476
10.806789	down	XM_001061142	LOC685482	685482
37.306534	down	XM_001063977	LOC685484	685484
56.472034	down	XM_001063993	LOC685488	685488
36.530582	down	XM_001064025	LOC685493	685493
5.663477	down	XM_001064032	LOC685494	685494
40.512356	down	XM_001064038	LOC685496	685496
3.4976676	down	XM_001064056	LOC685501	685501
18.546452	down	XM_001064061	LOC685503	685503
32.8828	down	XM_001064084	LOC685509	685509

26.529219	down	XM_001064094	LOC685512	685512
49.511265	down	XM_001064100	LOC685513	685513
13.596217	down	XM_001064154	LOC685526	685526
49.658073	down	XM_001064282	LOC685552	685552
21.890896	down	XM_001064306	LOC685559	685559
38.305103	down	XM_001064312	LOC685563	685563
49.69571	down	XM_001064363	LOC685573	685573
4.122992	down	XM_001064431	LOC685585	685585
41.25857	down	XM_001064447	LOC685593	685593
15.639191	down	XM_001054124	LOC685594	685594
3.814826	down	XM_001064471	LOC685597	685597
6.1858735	down	XM_001064490	LOC685601	685601
23.885727	down	XM_001064502	LOC685604	685604
51.302826	down	XM_001057826	LOC685609	685609
6.894287	down	XM_001064534	LOC685616	685616
37.138332	down	XM_001064543	LOC685617	685617
13.182072	down	XM_001064598	LOC685632	685632
30.885838	down	XM_001064613	LOC685636	685636
15.781533	down	XM_001064644	LOC685643	685643
13.74766	down	XM_001064652	LOC685645	685645
35.18665	down	XM_001064704	LOC685661	685661
16.755354	down	XM_001064718	LOC685663	685663
7.4317627	down	XM_001064759	LOC685667	685667
34.965855	down	XM_001064760	LOC685668	685668
2.5573652	down	XM_001064894	LOC685699	685699
50.161648	down	XM_001064946	LOC685711	685711
42.787697	down	XM_001064972	LOC685717	685717
9.668001	down	XM_001064987	LOC685720	685720
22.582552	down	XM_001064994	LOC685722	685722
2.3963625	down	XM_001065028	LOC685729	685729
92.38186	down	XM_001065049	LOC685735	685735
13.931398	down	XM_001065085	LOC685742	685742
13.955427	down	XM_001065090	LOC685743	685743
76.58995	down	XM_001065160	LOC685759	685759
70.23497	down	XM_001065161	LOC685760	685760
8.832701	down	XM_001065164	LOC685763	685763
6.110524	down	XM_001065185	LOC685767	685767
83.57015	down	XM_001065189	LOC685769	685769
86.86864	down	XM_001065214	LOC685773	685773
75.26684	down	XM_001057991	LOC685779	685779
4.221198	down	XM_001065266	LOC685784	685784
43.42222	down	XM_001065272	LOC685786	685786
55.68025	down	XM_217497	LOC685792	685792
51.987514	down	XM_001065304	LOC685796	685796

35.06623	down	XM_001065305	LOC685797	685797
88.7873	down	XM_001065332	LOC685805	685805
7.749696	down	XM_001065358	LOC685810	685810
79.488884	down	XM_001065446	LOC685835	685835
78.367676	down	XM_001065511	LOC685853	685853
43.07952	down	XM_001065537	LOC685863	685863
52.459595	down	XM_001065550	LOC685864	685864
2.470529	down	XM_001065551	LOC685865	685865
64.81163	down	XM_001065569	LOC685871	685871
36.19778	down	XM_001065570	LOC685872	685872
12.661789	down	XM_001065626	LOC685885	685885
15.318807	down	XM_001062645	LOC685888	685888
44.1653	down	XM_001065656	LOC685891	685891
3.4513662	down	XM_001065667	LOC685894	685894
31.154621	down	XM_001065677	LOC685898	685898
31.021404	down	XM_001065688	LOC685901	685901
34.73897	down	XM_001065695	LOC685904	685904
78.72594	down	XM_001065708	LOC685905	685905
45.658836	down	XM_001065719	LOC685908	685908
17.570324	down	XM_001065720	LOC685909	685909
66.03406	down	XM_001065748	LOC685918	685918
26.496717	down	XM_001065784	LOC685926	685926
45.767227	down	XM_001065801	LOC685929	685929
3.8365772	down	XM_001065802	LOC685930	685930
2.826458	down	XM_001065897	LOC685947	685947
40.034256	down	XM_001065901	LOC685948	685948
56.816067	down	XM_001065963	LOC685965	685965
65.72191	down	XM_001065967	LOC685966	685966
53.928425	down	XM_001065995	LOC685968	685968
38.731716	down	XM_001066016	LOC685974	685974
8.914893	down	XM_001066071	LOC685986	685986
45.155655	down	XM_001066074	LOC685987	685987
52.15992	down	XM_001066078	LOC685988	685988
78.015114	down	XM_001066079	LOC685989	685989
26.48578	down	XM_001066081	LOC685990	685990
73.63794	down	XM_001066107	LOC685994	685994
26.925642	down	XM_001066158	LOC686010	686010
2.889335	down	XM_001066168	LOC686013	686013
79.83359	down	XM_001066192	LOC686017	686017
64.340324	down	XM_001066193	LOC686018	686018
26.488224	down	XM_001066217	LOC686023	686023
5.7117534	down	XM_001066247	LOC686030	686030
18.154266	down	XM_001066250	LOC686031	686031
18.76002	down	XM_001066280	LOC686036	686036

30.272615	down	XM_001066294	LOC686038	686038
101.15336	down	XM_001066301	LOC686041	686041
19.44372	down	XM_001066333	LOC686046	686046
4.517275	down	XM_001066349	LOC686051	686051
88.00687	down	XM_001066356	LOC686053	686053
45.30945	down	XM_001066372	LOC686058	686058
22.077534	down	XM_001066380	LOC686061	686061
12.796879	down	XM_001066400	LOC686068	686068
62.673424	down	XM_001066470	LOC686082	686082
13.190135	down	XM_001066471	LOC686083	686083
28.404297	down	XM_001066478	LOC686085	686085
3.1184704	down	XM_001066513	LOC686092	686092
2.612402	down	XM_001066552	LOC686103	686103
21.516031	down	XM_001066563	LOC686107	686107
3.0318599	down	XM_001066569	LOC686109	686109
8.502085	down	XM_001066582	LOC686113	686113
23.570747	down	XM_001066607	LOC686119	686119
68.05165	down	XM_001066623	LOC686126	686126
97.32427	down	XM_001066636	LOC686130	686130
53.660427	down	XM_001066637	LOC686131	686131
49.043133	down	XM_001066652	LOC686135	686135
86.87125	down	XM_001066658	LOC686138	686138
2.7633426	down	XM_001066685	LOC686146	686146
59.867725	down	XM_001066698	LOC686147	686147
9.193101	down	XM_001072665	LOC686151	686151
29.625914	down	XM_001072725	LOC686165	686165
12.218003	down	XM_001072815	LOC686189	686189
2.391988	down	XM_001072847	LOC686198	686198
51.065712	down	XM_001072947	LOC686222	686222
23.536821	down	XM_001073195	LOC686269	686269
3.8735201	down	XM_001073234	LOC686274	686274
3.1778831	down	XM_001073302	LOC686288	686288
13.54863	down	XM_001073336	LOC686292	686292
3.467503	down	XM_001073372	LOC686298	686298
41.33542	down	XM_001073483	LOC686312	686312
3.0105715	down	XM_001073659	LOC686342	686342
3.4403744	down	XM_001073688	LOC686349	686349
4.50232	down	XM_001073812	LOC686374	686374
27.635242	down	XM_001073883	LOC686385	686385
19.057858	down	XM_001073908	LOC686388	686388
28.171556	down	XM_001073953	LOC686402	686402
17.446665	down	XM_001073995	LOC686411	686411
15.779254	down	XM_001074093	LOC686437	686437
3.3428009	down	XM_001074123	LOC686441	686441

36.39148	down	XM_001074203	LOC686458	686458
10.551707	down	XM_001074697	LOC686550	686550
8.634127	down	XM_001074770	LOC686567	686567
7.6202745	down	XM_001074822	LOC686576	686576
17.9321	down	XM_001074929	LOC686602	686602
32.513294	down	XM_001075007	LOC686620	686620
18.866362	down	XM_001075122	LOC686647	686647
7.780502	down	XM_001075152	LOC686657	686657
47.961777	down	XM_001075396	LOC686718	686718
67.67914	down	XM_001075397	LOC686719	686719
53.041466	down	XM_001075421	LOC686726	686726
25.4857	down	XM_001075726	LOC686784	686784
40.67228	down	XM_001075792	LOC686805	686805
10.477555	down	XM_001075922	LOC686833	686833
3.1959562	down	XM_001076002	LOC686849	686849
28.678259	down	XM_001076338	LOC686921	686921
7.477935	down	XM_001079025	LOC686930	686930
18.691704	down	XM_001076367	LOC686931	686931
42.335423	down	XM_001076379	LOC686934	686934
23.504473	down	XM_001076667	LOC686997	686997
8.49451	down	XM_001076650	LOC686999	686999
5.4545984	down	XM_001076680	LOC687001	687001
43.930584	down	XM_001076692	LOC687003	687003
2.5006578	down	XM_001076731	LOC687014	687014
75.027756	down	XM_001076767	LOC687021	687021
4.8002567	down	XM_001078538	LOC687022	687022
6.0898237	down	XM_001076805	LOC687033	687033
10.028852	down	XM_001076829	LOC687039	687039
2.643504	down	XM_001076904	LOC687056	687056
102.5269	down	XM_001076927	LOC687062	687062
50.942616	down	XM_001076949	LOC687067	687067
42.226307	down	XM_001077103	LOC687100	687100
29.868961	down	XM_001077115	LOC687103	687103
42.41067	down	XM_001077135	LOC687109	687109
2.1371982	down	XM_001077136	LOC687110	687110
44.419483	down	XM_001077138	LOC687111	687111
11.644762	down	XM_001077149	LOC687116	687116
32.41866	down	XM_001077217	LOC687140	687140
9.799984	down	XM_001077230	LOC687143	687143
40.180576	down	XM_001077315	LOC687164	687164
75.995415	down	XM_001077430	LOC687185	687185
8.373547	down	XM_001077517	LOC687207	687207
21.33105	down	XM_001077519	LOC687208	687208
2.0591085	down	XM_001077635	LOC687233	687233

6.975523	down	XM_001077684	LOC687244	687244
9.283893	down	XM_001077729	LOC687257	687257
6.793201	down	XM_001077837	LOC687284	687284
3.335757	down	XM_001077911	LOC687302	687302
30.818657	down	XM_001077913	LOC687303	687303
7.0113807	down	XM_001077997	LOC687326	687326
3.4524786	down	XM_001078010	LOC687329	687329
42.33868	down	XM_001078031	LOC687332	687332
9.617699	down	XM_001078036	LOC687334	687334
54.550797	down	XM_001078051	LOC687341	687341
21.879301	down	XM_001078132	LOC687356	687356
46.31574	down	XM_001078171	LOC687363	687363
4.0904174	down	XM_001078298	LOC687387	687387
2.8768554	down	XM_001078323	LOC687393	687393
13.634027	down	XM_001078329	LOC687396	687396
3.0763175	down	XM_001078361	LOC687399	687399
19.113956	down	XM_001078386	LOC687401	687401
7.9293084	down	XM_001078464	LOC687416	687416
23.722473	down	XM_001078530	LOC687432	687432
23.146046	down	XM_001078536	LOC687434	687434
26.812529	down	XM_001078688	LOC687464	687464
12.445636	down	XM_001078823	LOC687493	687493
39.552956	down	XM_001078830	LOC687494	687494
54.45329	down	XM_001078866	LOC687506	687506
2.6779017	down	XM_001075606	LOC687508	687508
41.678745	down	XM_001078885	LOC687512	687512
2.2246594	down	XM_001078888	LOC687513	687513
4.2787404	down	XM_001078904	LOC687516	687516
14.257421	down	XM_001078905	LOC687517	687517
39.02891	down	XM_001079008	LOC687537	687537
80.073616	down	XM_001079048	LOC687546	687546
43.370266	down	XM_001079078	LOC687553	687553
3.0506942	down	XM_001079200	LOC687573	687573
36.069107	down	XM_001079213	LOC687577	687577
83.58769	down	XM_001079286	LOC687589	687589
27.110493	down	XM_001079425	LOC687619	687619
4.316467	down	XM_001079474	LOC687636	687636
10.323531	down	XM_001079481	LOC687638	687638
28.606009	down	XM_001079500	LOC687642	687642
17.630638	down	XM_001079501	LOC687643	687643
4.101265	down	XM_001079557	LOC687650	687650
48.335884	down	XM_001079569	LOC687652	687652
24.37251	down	XM_001079681	LOC687675	687675
84.44011	down	XM_001079796	LOC687695	687695

2.803369	down	XM_001079840	LOC687704	687704
2.5558474	down	XM_001079780	LOC687707	687707
14.858839	down	XM_001079867	LOC687710	687710
3.667524	down	XM_001079883	LOC687714	687714
16.30547	down	XM_001079922	LOC687721	687721
2.7317758	down	XM_001079932	LOC687726	687726
47.486393	down	XM_001079968	LOC687736	687736
28.73535	down	XM_001079985	LOC687742	687742
6.381651	down	XM_001080001	LOC687746	687746
4.2066255	down	XM_001080031	LOC687759	687759
2.2726495	down	XM_001080152	LOC687789	687789
5.45598	down	XM_001080173	LOC687793	687793
7.7819366	down	XM_001080273	LOC687827	687827
2.4385412	down	XM_001080339	LOC687849	687849
3.8105552	down	XM_001080355	LOC687853	687853
3.3205252	down	XM_001080410	LOC687866	687866
67.74615	down	XM_001080491	LOC687900	687900
14.155491	down	XM_001080548	LOC687915	687915
28.210934	down	XM_001080561	LOC687920	687920
43.752308	down	XM_001080585	LOC687929	687929
65.63802	down	XM_001080588	LOC687931	687931
10.693789	down	XM_001080598	LOC687936	687936
3.4035645	down	XM_001080603	LOC687939	687939
32.25568	down	XM_001080652	LOC687954	687954
32.060017	down	XM_001080671	LOC687961	687961
11.302204	down	XM_001080713	LOC687970	687970
11.156057	down	XM_001080743	LOC687980	687980
2.5435648	down	XM_001080803	LOC687990	687990
35.549896	down	XM_001080804	LOC687991	687991
3.56501	down	XM_001080827	LOC687999	687999
19.373905	down	XM_001080924	LOC688029	688029
16.333685	down	XM_001080957	LOC688040	688040
5.8442545	down	XM_001080989	LOC688054	688054
32.86658	down	XM_001080993	LOC688055	688055
43.82072	down	XM_001081032	LOC688068	688068
25.312422	down	XM_001081035	LOC688069	688069
13.189616	down	XM_001081064	LOC688077	688077
48.839493	down	XM_001081092	LOC688091	688091
4.61995	down	XM_001081134	LOC688099	688099
2.2508192	down	XM_001081169	LOC688111	688111
23.833055	down	XM_001081232	LOC688127	688127
42.923676	down	XM_001081282	LOC688143	688143
80.45909	down	XM_001081341	LOC688160	688160
44.00499	down	XM_001081359	LOC688164	688164

70.94897	down	XM_001081391	LOC688172	688172
57.32129	down	XM_001081397	LOC688175	688175
2.1945555	down	XM_001081415	LOC688177	688177
44.21674	down	XM_001081419	LOC688178	688178
4.191743	down	XM_001081440	LOC688192	688192
6.935994	down	XM_001081497	LOC688205	688205
6.330815	down	XM_001081516	LOC688213	688213
36.072315	down	XM_001081518	LOC688215	688215
46.578712	down	XM_001081523	LOC688217	688217
5.141325	down	XM_001081533	LOC688219	688219
4.842204	down	XM_001081580	LOC688234	688234
6.1850004	down	XM_001081610	LOC688241	688241
2.9697354	down	XM_001081618	LOC688242	688242
62.575146	down	XM_001081629	LOC688246	688246
25.374475	down	XM_001081638	LOC688247	688247
36.55016	down	XM_001081643	LOC688249	688249
12.705298	down	XM_001081650	LOC688251	688251
2.5136676	down	XM_001081683	LOC688256	688256
29.654324	down	XM_001081727	LOC688274	688274
20.209259	down	XM_001081740	LOC688282	688282
45.365955	down	XM_001081781	LOC688302	688302
2.2769256	down	XM_001081840	LOC688314	688314
2.8886235	down	XM_001081814	LOC688319	688319
2.4822097	down	XM_001081836	LOC688330	688330
5.62864	down	XM_001081872	LOC688345	688345
78.92832	down	XM_001081886	LOC688351	688351
21.970222	down	XM_001081890	LOC688353	688353
3.9082124	down	XM_001081904	LOC688361	688361
15.829228	down	XM_001081918	LOC688370	688370
52.18425	down	XM_001081935	LOC688377	688377
13.163729	down	XM_001081936	LOC688378	688378
13.993079	down	XM_001081939	LOC688379	688379
4.6552916	down	XM_001081946	LOC688383	688383
40.969944	down	XM_001081948	LOC688384	688384
5.9995613	down	XM_001066771	LOC688398	688398
76.661674	down	XM_001066775	LOC688399	688399
83.58432	down	XM_001066825	LOC688410	688410
108.043884	down	XM_001066840	LOC688414	688414
9.015441	down	XM_001066867	LOC688421	688421
2.3496404	down	XM_001066874	LOC688424	688424
59.325157	down	XM_001066883	LOC688426	688426
79.652885	down	XM_001066895	LOC688428	688428
32.495083	down	XM_001066911	LOC688431	688431
96.59045	down	XM_001066958	LOC688441	688441

79.4378	down	XM_001066993	LOC688450	688450
84.960815	down	XM_001067045	LOC688460	688460
18.048967	down	XM_001067048	LOC688461	688461
60.967354	down	XM_001067063	LOC688464	688464
13.857585	down	XM_001067095	LOC688474	688474
52.924103	down	XM_001056972	LOC688479	688479
17.327042	down	XM_001067114	LOC688480	688480
39.157322	down	XM_001067133	LOC688487	688487
31.736286	down	XM_001067146	LOC688490	688490
6.5366564	down	XM_001067220	LOC688508	688508
69.553696	down	XM_001067241	LOC688519	688519
38.537598	down	XM_001067313	LOC688532	688532
80.8906	down	XM_001067327	LOC688538	688538
29.12666	down	XM_001067374	LOC688549	688549
23.25279	down	XM_001067410	LOC688558	688558
46.142178	down	XM_001067419	LOC688560	688560
13.066677	down	XM_001067468	LOC688570	688570
77.76228	down	XM_001058486	LOC688572	688572
3.0144775	down	XM_001067487	LOC688578	688578
8.275848	down	XM_001067516	LOC688585	688585
41.69992	down	XM_001067519	LOC688586	688586
87.587	down	XM_001067520	LOC688587	688587
38.552464	down	XM_001067531	LOC688592	688592
7.0865726	down	XM_001067543	LOC688595	688595
19.821527	down	XM_001067545	LOC688596	688596
30.433342	down	XM_001067566	LOC688603	688603
26.4782	down	XM_001067598	LOC688609	688609
29.366474	down	XM_001067670	LOC688625	688625
42.80296	down	XM_001067700	LOC688635	688635
74.05528	down	XM_001067734	LOC688644	688644
71.05983	down	XM_001058542	LOC688649	688649
37.31683	down	XM_001067766	LOC688653	688653
10.388799	down	XM_001067791	LOC688661	688661
70.06719	down	XM_001067795	LOC688663	688663
23.790619	down	XM_001067801	LOC688665	688665
43.19191	down	XM_001067832	LOC688670	688670
19.547953	down	XM_001067851	LOC688676	688676
31.932772	down	XM_001067853	LOC688678	688678
61.58795	down	XM_001058650	LOC688679	688679
49.274372	down	XM_001058713	LOC688687	688687
21.583681	down	XM_001067922	LOC688690	688690
27.919575	down	XM_001068044	LOC688720	688720
45.851093	down	XM_001068087	LOC688732	688732
14.258763	down	XM_001068112	LOC688741	688741

32.079994	down	XM_001068185	LOC688758	688758
23.983267	down	XM_001068207	LOC688764	688764
6.2491193	down	XM_001068253	LOC688778	688778
10.42045	down	XM_001068255	LOC688779	688779
23.097885	down	XM_001068261	LOC688780	688780
27.392838	down	XM_001068318	LOC688793	688793
53.065243	down	XM_001068328	LOC688797	688797
23.657476	down	XM_001068329	LOC688798	688798
5.4748063	down	XM_001068361	LOC688801	688801
26.791508	down	XM_001068371	LOC688803	688803
61.638027	down	XM_001068412	LOC688816	688816
45.478794	down	XM_001068419	LOC688818	688818
31.928793	down	XM_001068465	LOC688825	688825
56.39267	down	XM_001068481	LOC688831	688831
6.118022	down	XM_001068517	LOC688838	688838
60.437565	down	XM_001068610	LOC688861	688861
39.78659	down	XM_001068669	LOC688874	688874
10.792433	down	XM_001068681	LOC688876	688876
38.05601	down	XM_001068684	LOC688878	688878
38.796944	down	XM_001068707	LOC688888	688888
48.16997	down	XM_001068717	LOC688892	688892
68.15046	down	XM_001068783	LOC688909	688909
7.398708	down	XM_001068798	LOC688910	688910
24.345709	down	XM_001068828	LOC688920	688920
49.0023	down	XM_001068891	LOC688935	688935
7.6756406	down	XM_001068901	LOC688939	688939
15.430796	down	XM_001068909	LOC688943	688943
34.333633	down	XM_001068932	LOC688946	688946
5.1082225	down	XM_001068947	LOC688950	688950
2.4503613	down	XM_001068964	LOC688954	688954
6.2713895	down	XM_001068968	LOC688956	688956
73.96106	down	XM_001068988	LOC688964	688964
61.274517	down	XM_001069010	LOC688970	688970
4.891471	down	XM_001069014	LOC688972	688972
9.752943	down	XM_001069020	LOC688974	688974
24.70151	down	XM_001069078	LOC688991	688991
39.191246	down	XM_001069111	LOC688997	688997
74.38186	down	XM_001069134	LOC689001	689001
95.20825	down	XM_001069178	LOC689016	689016
10.353164	down	XM_001069210	LOC689024	689024
44.216347	down	XM_001069258	LOC689032	689032
16.989187	down	XM_001069291	LOC689039	689039
12.066406	down	XM_001069303	LOC689042	689042
7.8912024	down	XM_001069310	LOC689044	689044

60.22297	down	XM_001069354	LOC689058	689058
42.52373	down	XM_001069379	LOC689066	689066
47.77147	down	XM_001069422	LOC689076	689076
2.8396075	down	XM_001069425	LOC689077	689077
29.919302	down	XM_001069432	LOC689080	689080
48.09105	down	XM_001069448	LOC689083	689083
55.55339	down	XM_001058891	LOC689084	689084
27.279474	down	XM_001069469	LOC689086	689086
59.21226	down	XM_001069491	LOC689091	689091
73.82856	down	XM_001058953	LOC689094	689094
11.813415	down	XM_001069508	LOC689096	689096
87.5874	down	XM_001069556	LOC689107	689107
3.120316	down	XM_001058170	LOC689117	689117
8.318963	down	XM_001069594	LOC689119	689119
20.270517	down	XM_001069609	LOC689122	689122
56.45022	down	XM_001069621	LOC689124	689124
7.6265583	down	XM_001069645	LOC689128	689128
14.618992	down	XM_001069647	LOC689129	689129
6.8342557	down	XM_001069692	LOC689136	689136
14.4876375	down	XM_001069703	LOC689138	689138
98.24171	down	XM_001069756	LOC689150	689150
4.070094	down	XM_001069768	LOC689154	689154
29.900648	down	XM_001069824	LOC689169	689169
31.928625	down	XM_001069867	LOC689177	689177
34.86194	down	XM_001069895	LOC689182	689182
54.860584	down	XM_001069925	LOC689190	689190
95.62068	down	XM_001069934	LOC689193	689193
68.96887	down	XM_001069974	LOC689206	689206
53.815147	down	XM_001069993	LOC689215	689215
6.876138	down	XM_001070024	LOC689220	689220
56.7401	down	XM_001070040	LOC689225	689225
50.679035	down	XM_001070055	LOC689228	689228
26.290424	down	XM_001070064	LOC689231	689231
3.32627	down	XM_001070084	LOC689236	689236
56.646202	down	XM_001070094	LOC689241	689241
30.47463	down	XM_001070111	LOC689244	689244
38.29132	down	XM_001070112	LOC689245	689245
4.42432	down	XM_001070134	LOC689254	689254
18.906582	down	XM_001070169	LOC689261	689261
30.972649	down	XM_001070188	LOC689268	689268
102.80043	down	XM_001070205	LOC689270	689270
3.8558226	down	XM_001070234	LOC689278	689278
58.16741	down	XM_001070237	LOC689281	689281
22.55988	down	XM_001070260	LOC689287	689287

47.38658	down	XM_001070335	LOC689299	689299
52.3142	down	XM_001070367	LOC689312	689312
78.40627	down	XM_001070376	LOC689315	689315
2.1964989	down	XM_001070400	LOC689323	689323
53.866592	down	XM_001070403	LOC689326	689326
74.87943	down	XM_001059417	LOC689329	689329
7.2254763	down	XM_001070420	LOC689331	689331
4.2288837	down	XM_001070424	LOC689333	689333
71.9342	down	XM_001070438	LOC689338	689338
3.8651574	down	XM_001070451	LOC689341	689341
67.360176	down	XM_001070459	LOC689342	689342
13.528373	down	XM_001070469	LOC689346	689346
54.027836	down	XM_001054976	LOC689347	689347
4.9666085	down	XM_001070510	LOC689359	689359
71.570114	down	XM_001070518	LOC689362	689362
72.206894	down	XM_001070539	LOC689368	689368
18.669254	down	XM_001070623	LOC689390	689390
43.228683	down	XM_001070630	LOC689392	689392
74.06037	down	XM_001070689	LOC689410	689410
20.590792	down	XM_001070715	LOC689416	689416
65.938484	down	XM_001070719	LOC689417	689417
61.648502	down	XM_001070729	LOC689418	689418
6.2192583	down	XM_001070743	LOC689423	689423
5.5244865	down	XM_001070770	LOC689428	689428
44.41565	down	XM_001070788	LOC689435	689435
5.305113	down	XM_001070789	LOC689436	689436
4.47303	down	XM_001070812	LOC689444	689444
34.734253	down	XM_001059655	LOC689445	689445
10.431926	down	XM_001070833	LOC689447	689447
71.92005	down	XM_001070864	LOC689453	689453
6.2712693	down	XM_001070885	LOC689458	689458
24.39689	down	XM_001070913	LOC689465	689465
23.861471	down	XM_001059762	LOC689467	689467
50.55679	down	XM_001070948	LOC689478	689478
35.861298	down	XM_001070949	LOC689479	689479
3.2861443	down	XM_001070970	LOC689486	689486
2.4043906	down	XM_001070976	LOC689489	689489
51.704273	down	XM_001070998	LOC689497	689497
58.677612	down	XM_001070999	LOC689498	689498
47.09767	down	XM_001060016	LOC689501	689501
13.199415	down	XM_001071008	LOC689503	689503
49.57524	down	XM_001071038	LOC689511	689511
2.3186789	down	XM_001071057	LOC689518	689518
4.5842595	down	XM_001071058	LOC689519	689519

15.704088	down	XM_001071087	LOC689528	689528
48.78306	down	XM_001071141	LOC689541	689541
64.112885	down	XM_001060123	LOC689545	689545
68.65691	down	XM_001071174	LOC689554	689554
33.876106	down	XM_001071251	LOC689579	689579
7.5421367	down	XM_001071303	LOC689596	689596
46.316826	down	XM_001071362	LOC689612	689612
4.0370483	down	XM_001071429	LOC689628	689628
2.0865488	down	XM_001071446	LOC689634	689634
53.562225	down	XM_001071448	LOC689635	689635
30.844889	down	XM_001071465	LOC689640	689640
12.092289	down	XM_001071518	LOC689651	689651
69.386375	down	XM_001071539	LOC689658	689658
35.94934	down	XM_001071579	LOC689664	689664
66.33951	down	XM_001060225	LOC689666	689666
69.45555	down	XM_001055155	LOC689667	689667
58.736694	down	XM_001071623	LOC689677	689677
61.156055	down	XM_001071624	LOC689678	689678
34.846306	down	XM_001071625	LOC689679	689679
45.612694	down	XM_001060291	LOC689680	689680
46.10504	down	XM_001055277	LOC689681	689681
21.263905	down	XM_001071635	LOC689682	689682
59.795033	down	XM_001055335	LOC689690	689690
32.47841	down	XM_001071675	LOC689692	689692
17.900272	down	XM_001071752	LOC689714	689714
3.6855838	down	XM_001071764	LOC689718	689718
22.022844	down	XM_001071772	LOC689720	689720
53.69142	down	XM_001071797	LOC689727	689727
25.56198	down	XM_001071809	LOC689730	689730
24.90057	down	XM_001071817	LOC689734	689734
56.84238	down	XM_001071835	LOC689740	689740
4.6379356	down	XM_001071836	LOC689741	689741
3.7515354	down	XM_001071853	LOC689743	689743
41.563877	down	XM_001071871	LOC689749	689749
83.073616	down	XM_001071877	LOC689750	689750
20.063984	down	XM_001071886	LOC689753	689753
2.6408432	down	XM_001071896	LOC689757	689757
38.169117	down	XM_001071903	LOC689759	689759
6.5767956	down	XM_001071906	LOC689761	689761
63.34113	down	XM_001071912	LOC689762	689762
61.399338	down	XM_001071913	LOC689763	689763
62.562984	down	XM_001071936	LOC689767	689767
35.642525	down	XM_001071944	LOC689769	689769
55.419296	down	XM_001071950	LOC689774	689774

4.5307097	down	XM_001071955	LOC689776	689776
60.154854	down	XM_001071983	LOC689781	689781
4.1302876	down	XM_001072007	LOC689787	689787
22.871727	down	XM_001072019	LOC689791	689791
65.00725	down	XM_001072032	LOC689796	689796
69.99548	down	XM_001070608	LOC689797	689797
34.50156	down	XM_001072049	LOC689799	689799
43.643913	down	XM_001072069	LOC689802	689802
3.4743001	down	XM_001072103	LOC689811	689811
6.061954	down	XM_001072162	LOC689826	689826
90.54743	down	XM_001072168	LOC689827	689827
51.493618	down	XM_001072188	LOC689829	689829
47.794415	down	XM_001072212	LOC689837	689837
45.95997	down	XM_001072245	LOC689846	689846
69.14601	down	XM_001072263	LOC689850	689850
50.01259	down	XM_001072288	LOC689857	689857
61.261154	down	XM_001072303	LOC689861	689861
17.519491	down	XM_001072316	LOC689867	689867
60.393314	down	XM_001060593	LOC689870	689870
32.305515	down	XM_001072332	LOC689871	689871
79.507645	down	XM_001072354	LOC689878	689878
9.262281	down	XM_001072387	LOC689887	689887
11.132057	down	XM_001072398	LOC689889	689889
55.985287	down	XM_001072467	LOC689905	689905
32.807667	down	XM_001072493	LOC689913	689913
11.870276	down	XM_001073448	LOC689926	689926
29.075815	down	XM_001069035	LOC689927	689927
2.725153	down	XM_001072611	LOC689939	689939
81.2311	down	XM_001072652	LOC689948	689948
100.56399	down	XM_001072684	LOC689957	689957
39.431202	down	XM_001072685	LOC689958	689958
2.1264973	down	XM_001072700	LOC689962	689962
22.069115	down	XM_001069365	LOC689992	689992
6.4558587	down	XM_001072851	LOC689996	689996
76.59611	down	XM_001072858	LOC689999	689999
33.332264	down	XM_001072899	LOC690010	690010
52.823425	down	XM_001072965	LOC690024	690024
51.922054	down	XM_001073001	LOC690039	690039
3.0701487	down	XM_001073002	LOC690040	690040
39.797535	down	XM_001060929	LOC690042	690042
50.486305	down	XM_001073030	LOC690048	690048
63.048943	down	XM_001073060	LOC690054	690054
77.08811	down	XM_001073094	LOC690061	690061
22.941032	down	XM_001073109	LOC690062	690062

9.110562	down	XM_001073156	LOC690075	690075
65.59103	down	XM_001073178	LOC690081	690081
29.312374	down	XM_001073200	LOC690086	690086
4.177639	down	XM_001073272	LOC690103	690103
60.43265	down	XM_001073344	LOC690122	690122
6.608111	down	XM_001073355	LOC690124	690124
5.950752	down	XM_001073389	LOC690130	690130
75.91696	down	XM_001073403	LOC690135	690135
5.384746	down	XM_001073439	LOC690142	690142
56.368576	down	XM_001073445	LOC690145	690145
2.3823717	down	XM_001073454	LOC690146	690146
14.980081	down	XM_001073480	LOC690152	690152
84.295166	down	XM_001073536	LOC690167	690167
36.300682	down	XM_001073545	LOC690170	690170
5.0815334	down	XM_001073557	LOC690175	690175
68.29658	down	XM_001073570	LOC690179	690179
12.807584	down	XM_001073351	LOC690190	690190
13.159766	down	XM_001073728	LOC690222	690222
60.99428	down	XM_001073746	LOC690228	690228
45.236366	down	XM_001073844	LOC690250	690250
11.418671	down	XM_001073870	LOC690256	690256
7.961515	down	XM_001073924	LOC690268	690268
31.929342	down	XM_001073925	LOC690269	690269
9.984599	down	XM_001073928	LOC690270	690270
2.0771432	down	XM_001073940	LOC690274	690274
6.8605156	down	XM_001073943	LOC690276	690276
16.301693	down	XM_001073947	LOC690279	690279
39.803192	down	XM_001073950	LOC690280	690280
56.849464	down	XM_001073951	LOC690281	690281
9.533491	down	XM_001073960	LOC690284	690284
34.268444	down	XM_001073964	LOC690286	690286
106.25849	down	XM_001073967	LOC690288	690288
45.510067	down	XM_001074009	LOC690298	690298
91.75664	down	XM_001074152	LOC690341	690341
2.7730932	down	XM_001074160	LOC690343	690343
15.843454	down	XM_001074223	LOC690358	690358
60.898296	down	XM_001074232	LOC690362	690362
26.076832	down	XM_001074233	LOC690363	690363
5.3599825	down	XM_001074272	LOC690375	690375
78.10928	down	XM_001074284	LOC690379	690379
93.617294	down	XM_001074306	LOC690389	690389
47.686047	down	XM_001074324	LOC690395	690395
32.675297	down	XM_001074336	LOC690398	690398
41.45548	down	XM_001074345	LOC690400	690400

29.609497	down	XM_001074375	LOC690411	690411
3.4324386	down	XM_001074383	LOC690413	690413
46.909847	down	XM_001074400	LOC690415	690415
8.469469	down	XM_001072906	LOC690422	690422
2.1068673	down	XM_001074425	LOC690425	690425
53.562794	down	XM_001074433	LOC690429	690429
36.184643	down	XM_001074447	LOC690431	690431
94.892494	down	XM_001074459	LOC690439	690439
27.948122	down	XM_001074463	LOC690440	690440
36.11994	down	XM_001074469	LOC690443	690443
22.524773	down	XM_001074472	LOC690445	690445
46.92399	down	XM_001074473	LOC690446	690446
30.14306	down	XM_001074476	LOC690447	690447
25.981474	down	XM_001074485	LOC690452	690452
17.335434	down	XM_001074496	LOC690456	690456
60.571213	down	XM_001074498	LOC690457	690457
14.076051	down	XM_001074502	LOC690459	690459
16.643557	down	XM_001074509	LOC690463	690463
70.57289	down	XM_001074527	LOC690467	690467
41.883675	down	XM_001074534	LOC690469	690469
39.310677	down	XM_001074545	LOC690474	690474
9.484367	down	XM_001074554	LOC690476	690476
6.0076137	down	XM_001074561	LOC690479	690479
19.041714	down	XM_001074567	LOC690482	690482
46.084126	down	XM_001074598	LOC690490	690490
18.014143	down	XM_001074661	LOC690517	690517
78.66861	down	XM_001074680	LOC690523	690523
7.05655	down	XM_001074689	LOC690524	690524
59.04349	down	XM_001074751	LOC690542	690542
5.074503	down	XM_001074816	LOC690562	690562
82.36319	down	XM_001074825	LOC690565	690565
13.188784	down	XM_001074841	LOC690570	690570
38.515606	down	XM_001074851	LOC690576	690576
60.709225	down	XM_001074852	LOC690577	690577
47.09269	down	XM_001074868	LOC690582	690582
51.943596	down	XM_001074893	LOC690590	690590
8.653634	down	XM_001074983	LOC690618	690618
54.71622	down	XM_001074985	LOC690619	690619
55.510864	down	XM_001075002	LOC690624	690624
45.776123	down	XM_001075006	LOC690626	690626
23.509018	down	XM_001075019	LOC690629	690629
39.459476	down	XM_001075028	LOC690631	690631
58.965015	down	XM_001075069	LOC690643	690643
4.318817	down	XM_001075079	LOC690647	690647

85.93601	down	XM_001075121	LOC690659	690659
5.4325027	down	XM_001075132	LOC690662	690662
32.423172	down	XM_001062005	LOC690665	690665
43.2316	down	XM_001075149	LOC690667	690667
41.409504	down	XM_001075162	LOC690672	690672
13.380032	down	XM_001075204	LOC690684	690684
4.9970126	down	XM_001075254	LOC690694	690694
42.56233	down	XM_001075284	LOC690700	690700
15.12907	down	XM_001075303	LOC690707	690707
3.159078	down	XM_001075349	LOC690717	690717
10.823107	down	XM_001075359	LOC690719	690719
57.9827	down	XM_001075363	LOC690721	690721
45.99255	down	XM_001075438	LOC690739	690739
36.282738	down	XM_001075453	LOC690741	690741
6.598037	down	XM_001075478	LOC690744	690744
60.634247	down	XM_001075495	LOC690748	690748
5.8673716	down	XM_001075530	LOC690761	690761
63.35939	down	XM_001075604	LOC690782	690782
51.18491	down	XM_001075660	LOC690798	690798
9.872211	down	XM_001075714	LOC690811	690811
51.949673	down	XM_001075725	LOC690815	690815
69.61183	down	XM_001075749	LOC690822	690822
49.965847	down	XM_001075795	LOC690828	690828
35.58312	down	XM_001075885	LOC690854	690854
67.78772	down	XM_001075892	LOC690855	690855
36.393307	down	XM_001075910	LOC690860	690860
2.2146409	down	XM_001075927	LOC690864	690864
79.00221	down	XM_001075937	LOC690867	690867
2.0562112	down	XM_001075950	LOC690871	690871
81.406235	down	XM_001075967	LOC690874	690874
5.3230686	down	XM_001076026	LOC690885	690885
20.723368	down	XM_001076036	LOC690889	690889
66.34819	down	XM_001076048	LOC690892	690892
57.23547	down	XM_001076066	LOC690896	690896
36.398132	down	XM_001076113	LOC690910	690910
55.471836	down	XM_001076169	LOC690924	690924
12.816829	down	XM_001076181	LOC690927	690927
64.67827	down	XM_001076188	LOC690929	690929
30.360683	down	XM_001078193	LOC690930	690930
2.9913085	down	XM_001076204	LOC690934	690934
59.582264	down	XM_001076213	LOC690937	690937
30.525276	down	XM_001076239	LOC690943	690943
70.83947	down	XM_001076268	LOC690947	690947
13.400257	down	XM_001076286	LOC690951	690951

59.49424	down	XM_001076337	LOC690969	690969
7.8700547	down	XM_001076357	LOC690974	690974
75.481285	down	XM_001076377	LOC690977	690977
16.725073	down	XM_001076398	LOC690985	690985
54.046158	down	XM_001076453	LOC690998	690998
7.860008	down	XM_001071458	LOC691000	691000
29.201902	down	XM_001076475	LOC691005	691005
5.699181	down	XM_001076490	LOC691008	691008
6.343252	down	XM_001076494	LOC691009	691009
4.594149	down	XM_001076557	LOC691021	691021
37.899372	down	XM_001076573	LOC691025	691025
4.4744363	down	XM_001076600	LOC691033	691033
52.800243	down	XM_001076744	LOC691070	691070
25.159592	down	XM_001076750	LOC691073	691073
23.476376	down	XM_001076760	LOC691077	691077
33.346348	down	XM_001076823	LOC691094	691094
72.13982	down	XM_001076856	LOC691108	691108
23.166252	down	XM_001076859	LOC691109	691109
3.7605805	down	XM_001076883	LOC691114	691114
52.85701	down	XM_001076889	LOC691115	691115
35.185905	down	XM_001076891	LOC691117	691117
5.884287	down	XM_001076892	LOC691118	691118
8.768778	down	XM_001076905	LOC691125	691125
45.35032	down	XM_001076960	LOC691136	691136
7.7251887	down	XM_001076975	LOC691141	691141
7.290775	down	XM_001076976	LOC691142	691142
32.291126	down	XM_001076998	LOC691146	691146
44.526638	down	XM_001077145	LOC691188	691188
10.3089	down	XM_001077165	LOC691192	691192
83.6373	down	XM_001077250	LOC691219	691219
24.927282	down	XM_001077260	LOC691221	691221
3.7167044	down	XM_001077271	LOC691224	691224
2.5010834	down	XM_001077272	LOC691225	691225
22.88434	down	XM_001077347	LOC691241	691241
16.194223	down	XM_001077373	LOC691247	691247
68.71651	down	XM_001077436	LOC691262	691262
45.318104	down	XM_001077468	LOC691270	691270
33.06413	down	XM_001077501	LOC691274	691274
36.85168	down	XM_001077529	LOC691282	691282
21.204136	down	XM_001077753	LOC691331	691331
26.71564	down	XM_001077757	LOC691332	691332
23.630014	down	XM_001077758	LOC691333	691333
61.43072	down	XM_001077759	LOC691334	691334
43.019363	down	XM_001077840	LOC691353	691353

5.4830275	down	XM_001077864	LOC691358	691358
23.630625	down	XM_001077906	LOC691366	691366
2.0751593	down	XM_001077980	LOC691381	691381
24.619114	down	XM_001077994	LOC691383	691383
63.993656	down	XM_001078062	LOC691395	691395
53.12758	down	XM_001078077	LOC691401	691401
49.29711	down	XM_001078092	LOC691403	691403
59.67002	down	XM_001078110	LOC691406	691406
60.420288	down	XM_001078125	LOC691410	691410
61.78119	down	XM_001078150	LOC691414	691414
11.251637	down	XM_001068693	LOC691418	691418
4.8848987	down	XM_001078185	LOC691421	691421
2.1227236	down	XM_001078268	LOC691439	691439
23.744083	down	XM_001078288	LOC691443	691443
53.818295	down	XM_001078322	LOC691451	691451
36.99558	down	XM_001078439	LOC691473	691473
35.54924	down	XM_001078495	LOC691487	691487
4.1092987	down	XM_001078547	LOC691499	691499
38.021133	down	XM_001078554	LOC691502	691502
6.969195	down	XM_001078628	LOC691519	691519
35.91053	down	XM_001078640	LOC691522	691522
87.18262	down	XM_001078740	LOC691539	691539
26.033316	down	XM_001078835	LOC691561	691561
56.386166	down	XM_001078865	LOC691569	691569
14.442444	down	XM_001078893	LOC691573	691573
64.28848	down	XM_001078898	LOC691575	691575
70.643555	down	XM_001078899	LOC691576	691576
51.261486	down	XM_001078931	LOC691586	691586
30.684412	down	XM_001078957	LOC691594	691594
59.706417	down	XM_001078958	LOC691595	691595
6.1078267	down	XM_001078967	LOC691596	691596
38.981792	down	XM_001078980	LOC691600	691600
57.613567	down	XM_001078992	LOC691605	691605
11.783403	down	XM_001079023	LOC691612	691612
8.974823	down	XM_001079034	LOC691615	691615
29.751987	down	XM_001079035	LOC691616	691616
4.47654	down	XM_001079045	LOC691619	691619
45.541847	down	XM_001079070	LOC691627	691627
51.481007	down	XM_001079096	LOC691633	691633
4.60462	down	XM_001079135	LOC691642	691642
12.845527	down	XM_001079143	LOC691646	691646
2.1262844	down	XM_001079159	LOC691653	691653
2.9235134	down	XM_001079177	LOC691660	691660
23.512321	down	XM_001079199	LOC691666	691666

34.847424	down	XM_001079224	LOC691672	691672
54.491943	down	XM_001079225	LOC691673	691673
4.2270813	down	XM_001079245	LOC691679	691679
70.669174	down	XM_001079247	LOC691681	691681
9.396431	down	XM_001079259	LOC691684	691684
73.355225	down	XM_001079285	LOC691692	691692
71.283325	down	XM_001079331	LOC691705	691705
15.693861	down	XM_001079351	LOC691712	691712
44.032154	down	XM_001079400	LOC691728	691728
56.572483	down	XM_001079430	LOC691735	691735
63.767822	down	XM_001079476	LOC691744	691744
36.5234	down	XM_001079486	LOC691748	691748
58.43748	down	XM_001079519	LOC691754	691754
70.76428	down	XM_001079538	LOC691760	691760
68.28949	down	XM_001079592	LOC691773	691773
2.253587	down	XM_001079602	LOC691777	691777
82.76729	down	XM_001079611	LOC691780	691780
69.08682	down	XM_001079621	LOC691784	691784
83.396484	down	XM_001079655	LOC691793	691793
43.337265	down	XM_001079664	LOC691795	691795
79.68269	down	XM_001079666	LOC691796	691796
9.585123	down	XM_001079667	LOC691797	691797
65.42204	down	XM_001079679	LOC691800	691800
72.45451	down	XM_001079688	LOC691802	691802
3.3715205	down	XM_001079726	LOC691809	691809
34.23636	down	XM_001079748	LOC691818	691818
20.827887	down	XM_001079793	LOC691833	691833
47.51803	down	XM_001079807	LOC691838	691838
2.4097273	down	XM_001079808	LOC691839	691839
18.092453	down	XM_001079876	LOC691854	691854
2.809453	down	XM_001079928	LOC691872	691872
57.41471	down	XM_001079964	LOC691883	691883
2.0404181	down	XM_001080046	LOC691904	691904
2.1381824	down	XM_001080055	LOC691906	691906
34.319633	down	XM_001080938	LOC692003	692003
30.748688	down	XM_001080954	LOC692006	692006
6.229728	down	XM_001081234	LOC692026	692026
14.722045	down	XM_001081377	LOC692042	692042
26.46031	down	XM_001081410	LOC692050	692050
44.66004	down	NM_023969	Lpar3	66025
4.0432563	down	XM_001055334	Lpin2	316737
32.997707	down	XM_001070693	Lrfn4	688721
2.6263492	down	NM_139331	Lrit1	246214
3.5104558	down	XM_231214	Lrp1b	311926

3.170277	down	XM_001067386	Lrp5	293649
4.2827444	down	XM_001066578	Lrp8	362558
3.2989945	down	XM_001070748	Lrrc16a	306941
6.031827	down	XM_001070537	Lrrc25	498605
9.597979	down	XM_001056018	Lrrc27	499281
3.7323048	down	NM_001024995	Lrrc33	303875
2.6841311	down	NM_001004201	Lrrc46	287653
2.1527662	down	XM_342990	Lrrc47	362672
3.407298	down	XM_001073479	Lrrc49	300763
2.906591	down	NM_001077434	Lrrc52	289199
5.427192	down	XM_001070170	Lrrc55	311171
6.039981	down	XM_001075896	Lrrc68	308398
6.075941	down	XM_001055887	Lrrc69	500415
8.838449	down	NM_001014269	Lrrfip1	367314
3.3820095	down	NM_001024307	Lrriq3	499732
22.825962	down	NM_022634	Lst1	64569
2.4534311	down	NM_212507	Ltb	361795
8.296159	down	NM_053640	Ltb4r2	114098
6.417817	down	NM_153726	Ly49s3	266768
3.8834832	down	NM_001009494	Ly49s7	494203
20.22765	down	XM_001068565	Ly49si2	494207
2.7200787	down	NM_001001969	Ly6g6c	294241
13.607564	down	NM_001001970	Ly6g6d	415062
5.9729137	down	NM_001007677	Lyl1	304663
29.795622	down	XM_001053512	Lypd6	679564
8.359736	down	NM_031342	Lypla2	83510
8.867958	down	XM_217189	Lysmd2	300839
32.240692	down	XM_001078029	Lyzl4	363168
35.38889	down	NM_001014247	Lzts2	365468
9.547706	down	XM_001061025	Mad2l1	297176
3.2358937	down	NM_017190	Mag	29409
14.357118	down	NM_001014182	Mall	362211
5.6015406	down	XM_576071	Map3k9	500690
3.1104002	down	XM_001071701	Map4k2	293694
3.4108315	down	XM_214968	Map7	293016
3.5150697	down	XM_341399	Mapk8	116554
6.2471967	down	XM_001080877	Mapkbp1	362197
12.514434	down	NM_030862	Marcksl1	81520
3.0561066	down	NM_053947	Mark1	117016
7.8157635	down	XM_001057742	Mark4	680407
5.502951	down	XM_001055222	Marveld1	309375
5.0564394	down	XM_345145	Marveld2	365657
7.5161986	down	NM_022210	Max	60661
3.4017055	down	XM_001074311	Mbd3l2	690392

2.1557026	down	XM_001055890	Mbd6	362892
2.179505	down	NM_001024996	Mblac1	304346
5.847035	down	NM_001025291	Mbp	24547
8.001954	down	NM_013099	Mc4r	25635
4.533139	down	XM_229172	Mcf2	317598
2.2931247	down	XM_001068436	Mcm4	29728
2.8328946	down	XM_226316	Mcm5	291885
21.791693	down	XM_001058060	Mcpt1l2	408238
11.386038	down	XM_573785	Mcpt1l3	408209
5.4947343	down	XM_573784	Mcpt1l4	408239
5.735345	down	NM_019321	Mcpt4	54270
6.2785344	down	NM_021598	Mcpt8	29269
2.2921593	down	XM_573782	Mcpt8l2	408240
2.6488986	down	NM_019323	Mcpt9	54272
3.1351993	down	XM_001054964	Mctp1	309928
2.1919465	down	NM_001044237	Mcts1	302500
8.034596	down	NM_030859	Mdk	81517
12.574654	down	XM_341880	Me3	361602
5.197479	down	NM_001047115	Mect1	684527
2.8948483	down	XM_001069345	Med19	311165
2.0202715	down	XM_001059017	Med28	305391
7.155323	down	NM_031634	Mefv	58923
5.4075146	down	XM_001077460	Mei1	315162
2.5235405	down	XM_001055664	Meig1	679834
3.499409	down	NM_001009962	Metrn	287151
2.2521274	down	XM_001074994	Mettl11b	289167
27.796404	down	XM_001060093	Mettl8	502633
2.7630832	down	XM_001068105	Mex3b	308790
2.0651255	down	XM_001062342	Mex3c	307271
2.0480537	down	XM_001076492	Mex3d	299613
22.360043	down	XM_001060365	Mfsd7	305625
4.7274547	down	XM_001059451	Mfsd9	316356
3.8546414	down	NM_019239	Mgat3	29582
4.864619	down	NM_001044300	MGC112715	690899
4.5682716	down	NM_001024893	MGC114427	317297
7.854323	down	NM_001024909	MGC114464	500925
4.7481155	down	NM_001024895	MGC114492	317324
34.140636	down	NM_001025755	MGC116197	367620
3.479783	down	NM_001044281	MGC93861	682010
2.313706	down	NM_001007645	MGC95152	297109
3.018324	down	NM_030852	Mia	81510
3.8310487	down	XM_001076784	Midn	314623
2.2539222	down	XM_001074414	Mier2	362841
3.8645608	down	XM_001056221	Mki67	291234

4.4437003	down	XM_001057262	Mkrn3	292988
6.8396497	down	XM_001056158	Mllt1	301119
8.64005	down	XM_001079320	Mlxip	304479
4.144559	down	NM_001037217	Mmd2	304301
3.9238944	down	NM_001013967	Mmgt2	303211
5.659312	down	NM_080776	Mmp16	65205
4.0149035	down	XM_001072313	Mmp1b	300338
2.5774508	down	NM_031054	Mmp2	81686
11.781693	down	NM_031757	Mmp24	83513
2.1626241	down	XM_001059491	Mmrn2	306288
3.239179	down	XM_001072054	Mnd1	295160
6.8971767	down	XM_001080632	Mnt	287521
48.97693	down	XM_001077777	Mobp	25037
4.9920454	down	XM_001056788	Mocos	361300
10.397816	down	XM_001062730	Morn2	500606
7.898843	down	XM_001068860	Mov10	310756
4.5440974	down	XM_001077024	Mpn2	287358
4.9639454	down	XM_001081487	Mpp2	85275
4.5286646	down	XM_001077948	Mpped1	362971
14.026122	down	NM_001037642	Mptx	289243
2.082786	down	XM_001070218	Mpv17l2	290645
2.5303257	down	NM_203470	Mrgprg	309133
2.3597064	down	XM_001081102	Mrm1	363661
9.095984	down	XM_001054992	Mrpl32	291206
3.828479	down	XM_001075218	Ms4a1	309217
11.82182	down	XM_001075502	Ms4a11	361735
6.6323705	down	NM_024352	Mst1	24566
3.1401222	down	XM_001070192	Mst4	317589
2.832362	down	XM_001062660	Mt4	498911
6.090808	down	XM_001056154	Mthfd2	680308
3.6531768	down	XM_001058546	Mtmr15	309256
6.2810917	down	XM_001064860	Mtss1	362918
22.749544	down	XM_001063115	Muc6	282586
16.131128	down	NM_001002826	Mug2	408236
6.028449	down	NM_203325	Mup5	298107
7.025984	down	NM_133316	Mutyh	170841
8.210325	down	NM_031062	Mvd	81726
23.345438	down	XM_342716	Mxd1	362391
5.059559	down	XM_001074923	Mxra7	690599
31.748907	down	NM_001013096	Mycn	298894
16.3899	down	XM_001064527	Myh15	303965
3.2789793	down	NM_017239	Myh6	29556
2.3204646	down	XM_001065619	Myl6b	685883
5.4573236	down	XM_001079144	Myo1f	314654

5.7231236	down	XM_001053631	Myo3a	498806
7.1127553	down	XM_001058020	Mysm1	298247
6.2055545	down	XM_213675	N6amt1	288309
2.2863724	down	XM_001069804	Nacad	289786
8.04616	down	NM_001024789	Nap1l2	317247
9.791512	down	XM_001072040	Nat14	361500
9.706375	down	XM_001055639	Ncaph	680089
2.0732782	down	NM_053734	Ncf1	114553
8.280826	down	XM_001066310	Ncf2	364018
2.4034896	down	NM_031822	Ncoa2	83724
3.9462192	down	XM_001058653	Ncoa7	498995
4.597035	down	NM_198783	Ndufaf4	362495
6.034013	down	NM_022302	Necab1	64169
5.278409	down	NM_199096	Necap2	298598
2.8999805	down	NM_031783	Nefl	83613
22.285177	down	NM_017029	Nefm	24588
3.5057588	down	XM_001072927	Nek11	315978
3.418913	down	XM_001055166	Nek2	114482
7.532752	down	XM_001080757	Nek8	287473
7.744982	down	NM_057190	Nelf	117536
9.513484	down	NM_054010	Neu3	117185
2.6788435	down	XM_001069279	Neurl	309459
3.9271693	down	NM_053909	Nfasc	116690
6.14865	down	XM_001072834	Nfatc2	311658
6.3411474	down	XM_001062534	Nfkbiz	304005
3.140116	down	XM_001064398	Nfx1	313166
2.1277094	down	NM_031553	Nfyb	25336
20.916132	down	NM_012610	Ngfr	24596
4.03198	down	XM_001077752	Nhedc2	365946
2.2028677	down	XM_001070369	Nhsl2	317253
2.8003852	down	NM_021595	Ninj2	59115
6.7066956	down	XM_001054918	Nipa1	308668
3.1094205	down	XM_233308	Nkrf	298316
15.649263	down	XM_001056222	Nkx2-3	309389
3.0778236	down	NM_022239	Nmu	63887
7.148584	down	XM_001066533	Nnmt	300691
9.34008	down	XM_001068034	Nol6	313167
2.0595224	down	XM_001060437	Nol7	498727
2.6617675	down	XM_240636	Nonol1	306912
7.098425	down	NM_012611	Nos2	24599
6.540032	down	NM_001002827	Notch4	406162
2.9057543	down	NM_053683	Nox1	114243
2.0220826	down	XM_001077267	Noxa1	311793
5.3632717	down	NM_153293	Npb	259222

5.090219	down	NM_001004231	Npdc1	296562
5.942974	down	NM_022586	Npff	60337
2.1319845	down	NM_012612	Nppa	24602
2.2299125	down	NM_053750	Nppc	114593
3.5727162	down	NM_030841	Nptxr	81005
4.6726403	down	NM_023952	Npvf	60570
2.1178966	down	NM_022941	Nr1i3	65035
8.882359	down	NM_031130	Nr2f1	81808
3.0644684	down	NM_019328	Nr4a2	54278
6.578611	down	NM_178096	Nrep	338475
14.358019	down	XM_001063076	Nrg2	432361
44.821434	down	NM_201418	Ns5atp9	300795
3.032383	down	XM_001058107	Nsbp1_predicted	365273
4.442925	down	NM_024128	Nsg1	25247
12.288395	down	NM_001007617	Nuak2	289419
11.843052	down	XM_001056321	Nudt11	680248
4.6374454	down	XM_001063939	Nudt13	682978
10.848962	down	XM_001072855	Nudt16	363129
2.028462	down	XM_001061438	Nup98	81738
2.0701797	down	NM_053611	Nupr1	113900
3.0628047	down	XM_001055001	Nxph2	679932
2.4575198	down	NM_001009493	Oas3	494202
10.521086	down	XM_218245	Obox2	292574
10.061436	down	XM_001073855	Obox6	292629
3.30411	down	NM_001033958	Obp3	259247
2.2060997	down	NM_031329	Ocln	83497
3.8640757	down	NM_024126	Odf1	24610
4.018731	down	XM_001077384	Odf3l2	299600
11.682218	down	NM_001007670	Odf4	303236
10.296021	down	XM_001080849	Oip5	499873
2.2653391	down	NM_053573	Olfm1	93667
6.380356	down	NM_001013192	Olfml1	361621
26.194511	down	NM_001000077	Olr1000	288891
5.2013435	down	NM_001001371	Olr1002	288881
6.2929773	down	NM_001000070	Olr1016	288858
3.7397015	down	NM_001000149	Olr108	293247
2.852506	down	NM_001000709	Olr1084	404958
3.850191	down	XM_234873	Olr1088	299582
34.582493	down	NM_001001379	Olr1090	299553
26.968447	down	NM_001001380	Olr1092	299570
27.239723	down	NM_001001388	Olr1093	366844
11.302276	down	NM_001000416	Olr1095	299576
2.4902768	down	NM_001000743	Olr110	405007
5.0335655	down	NM_001000424	Olr1107	300202

6.217832	down	NM_001000869	Olr1163	405166
7.0649843	down	NM_001000594	Olr1193	367032
44.941307	down	NM_001000438	Olr1214	300538
4.5216036	down	NM_001000442	Olr1226	300551
4.7892838	down	NM_001000813	Olr1231	405099
5.2224593	down	NM_001000446	Olr1234	300558
7.54174	down	NM_001000447	Olr1235	300560
4.67142	down	NM_001000807	Olr1247	405093
17.076181	down	NM_173300	Olr1271	286959
4.8689923	down	NM_001000460	Olr1293	300599
2.838081	down	NM_001000598	Olr1295	367065
42.935184	down	NM_001000471	Olr1320	300621
28.715971	down	NM_001000476	Olr1330	300630
18.459726	down	NM_001000480	Olr1337	300637
47.15089	down	NM_001001121	Olr1347	406024
7.5632777	down	NM_001000493	Olr1356	302954
12.587814	down	NM_001000858	Olr1364	405150
15.550094	down	NM_214828	Olr1378	287086
8.651469	down	NM_214833	Olr1385	287236
38.337124	down	NM_001000788	Olr1388	405066
21.04149	down	NM_001000161	Olr139	293278
5.075781	down	NM_001001089	Olr1391	405983
78.14939	down	NM_001001091	Olr1394	405985
19.783892	down	NM_001001092	Olr1395	405986
19.71244	down	NM_001001095	Olr1399	405990
7.9897656	down	NM_001000162	Olr140	293279
12.226789	down	NM_001001096	Olr1400	405991
17.948668	down	NM_001000003	Olr1401	287247
2.7149303	down	NM_001000784	Olr1410	405062
2.1245646	down	NM_001000008	Olr1418	287313
29.22779	down	NM_001000007	Olr1422	287310
20.251244	down	NM_001000779	Olr1424	405056
13.490339	down	NM_001000010	Olr1425	287316
8.1715	down	NM_001000011	Olr1428	287318
24.200699	down	NM_001000016	Olr1439	287328
29.595545	down	NM_001000774	Olr1450	405049
55.049885	down	NM_001000957	Olr1451	405306
47.95111	down	NM_001000772	Olr1453	405047
59.85625	down	NM_001000771	Olr1455	405046
6.048687	down	NM_001000770	Olr1459	405045
7.1467223	down	NM_001000944	Olr1472	405286
29.402283	down	NM_001000527	Olr1481	363639
5.9359736	down	NM_001000719	Olr1488	404971
15.969616	down	NM_001000718	Olr1490	404970

6.925745	down	NM_001000528	Olr1509	363642
7.300809	down	NM_001000036	Olr1514	287514
12.41223	down	NM_001001098	Olr1515	405994
45.895306	down	NM_001000038	Olr1516	287520
8.785128	down	NM_001000039	Olr1521	287613
2.8680081	down	NM_001000547	Olr154	365333
3.8902767	down	NM_001001105	Olr1545	406001
3.481496	down	NM_001000044	Olr1566	287973
14.639068	down	NM_001000043	Olr1567	287970
28.619844	down	NM_001000042	Olr1569	287969
2.4950273	down	XM_001072702	Olr1572	288490
3.6514602	down	NM_001000084	Olr1592	289254
9.341271	down	NM_001000502	Olr1606	305711
3.827247	down	NM_001000534	Olr1607	364275
10.615909	down	NM_001000101	Olr1644	290055
2.8416383	down	NM_001000172	Olr165	293312
4.368802	down	NM_001001007	Olr1664	405375
7.133879	down	NM_001000267	Olr1668	294140
4.032165	down	NM_001000891	Olr1680	405192
3.7697225	down	NM_001000270	Olr1684	294151
12.335599	down	NM_001001373	Olr1686	294152
6.5970187	down	NM_001000274	Olr1690	294162
2.3744366	down	NM_001000272	Olr1695	294159
3.8883882	down	NM_001001111	Olr1697	406010
13.516739	down	NM_001001112	Olr1699	406011
21.10054	down	NM_001000271	Olr1704	294155
8.263615	down	NM_001000887	Olr1705	405188
22.24594	down	NM_001001115	Olr1707	406014
11.352087	down	NM_001000276	Olr1708	294166
15.534661	down	NM_001000886	Olr1710	405186
32.405903	down	NM_214460	Olr1718	405205
20.889286	down	NM_001001116	Olr1720	406015
14.846115	down	NM_001001117	Olr1722	406016
7.142195	down	NM_001001428	Olr1748	405199
11.625212	down	NM_001000489	Olr1765	302821
18.535606	down	NM_001000181	Olr183	293328
2.6462069	down	NM_001001031	Olr186	405917
19.529459	down	NM_001000538	Olr1l	365170
3.1661525	down	NM_001000194	Olr208	293355
4.164333	down	NM_001000199	Olr217	293364
22.903816	down	NM_001000552	Olr220	365341
5.7903495	down	NM_001000200	Olr221	293367
6.255267	down	NM_001000211	Olr239	293383
5.794699	down	NM_001000226	Olr279	293429

7.1540747	down	NM_001000233	Olr295	293595
47.39451	down	NM_001001039	Olr304	405925
19.975225	down	NM_001000240	Olr305	293603
3.240359	down	NM_001000955	Olr308	405304
5.1219826	down	NM_001000764	Olr312	405038
22.799376	down	NM_001000247	Olr325	293763
25.751652	down	NM_001000248	Olr326	293764
7.771758	down	NM_001000250	Olr332	293769
10.622918	down	NM_001000689	Olr34	404894
13.164186	down	NM_001000260	Olr376	293809
28.666424	down	NM_001001286	Olr378	405366
2.979711	down	NM_001000914	Olr392	405227
5.0539417	down	NM_001000559	Olr395	366024
2.3326173	down	NM_001000111	Olr4	292560
5.4720583	down	NM_001001014	Olr402	405388
3.7374625	down	NM_001000382	Olr404	296667
7.709469	down	NM_001000391	Olr423	296685
8.773262	down	NM_001000393	Olr425	296687
10.573541	down	XM_001072921	Olr437	502758
41.617474	down	NM_001000282	Olr440	295711
2.1064467	down	NM_001001050	Olr446	405937
10.82622	down	NM_001000298	Olr466	295736
29.359922	down	NM_001001125	Olr471	406046
2.0543778	down	NM_001000304	Olr477	295747
4.077801	down	NM_001000305	Olr479	295748
4.228673	down	NM_001000561	Olr482	366099
2.399478	down	NM_001000674	Olr529	404877
9.885054	down	NM_001001051	Olr531	405939
4.5540347	down	NM_001000565	Olr541	366104
3.6836207	down	NM_001000322	Olr550	295795
4.7667437	down	NM_001000748	Olr60	405017
3.4667673	down	NM_001001022	Olr61	405906
2.480171	down	NM_001000640	Olr649	404833
17.065022	down	NM_001000638	Olr652	404831
5.6662946	down	NM_001000925	Olr661	405243
4.8645334	down	NM_001001060	Olr662	405948
4.9182963	down	NM_001001279	Olr67	365324
15.146868	down	NM_001001064	Olr697	405952
3.2620354	down	NM_001000768	Olr7	405043
2.430229	down	NM_001001065	Olr707	405953
3.7186642	down	NM_001000360	Olr713	295907
5.4902997	down	NM_001000620	Olr726	404812
3.576153	down	NM_001000619	Olr727	404811
2.3004646	down	NM_001000618	Olr733	404810

13.385167	down	NM_001000134	Olr74	293218
6.9101443	down	NM_001000575	Olr741	366120
3.4093866	down	NM_001000577	Olr744	366122
2.3737156	down	NM_001000615	Olr749	404804
16.386234	down	XM_001080296	Olr763	296016
2.5303323	down	XM_001080310	Olr765	499860
18.053871	down	NM_001000917	Olr779	405232
6.541452	down	NM_001000604	Olr784	404793
3.6530972	down	NM_001000113	Olr8	292563
23.446629	down	NM_001000853	Olr803	405143
2.3562772	down	NM_001000976	Olr806	405331
3.3652248	down	NM_001000851	Olr807	405141
14.8159	down	NM_001000850	Olr808	405140
5.60217	down	NM_001000846	Olr813	405136
4.441015	down	NM_001000843	Olr819	405132
7.8784976	down	NM_001000974	Olr820	405329
22.335098	down	NM_001000904	Olr825	405214
4.2647486	down	NM_001000903	Olr826	405213
17.980494	down	NM_001000901	Olr829	405211
70.25865	down	NM_001000414	Olr840	298733
8.345334	down	NM_001000585	Olr859	366458
31.15422	down	NM_001000410	Olr865	298474
14.588429	down	NM_001000821	Olr868	405110
16.968374	down	NM_001000708	Olr876	404957
17.96841	down	NM_001000055	Olr880	288790
14.650229	down	NM_001001358	Olr883	288803
6.981686	down	NM_001000745	Olr89	405012
26.317287	down	NM_001001354	Olr917	288795
6.569083	down	NM_001000588	Olr927	366799
4.9880724	down	NM_001001024	Olr95	405909
4.4375887	down	NM_001000487	Olr962	302045
2.5508068	down	NM_001001073	Olr964	405963
3.2873838	down	XM_001075549	Opa3	308409
2.9446223	down	XM_001068634	Ophn1	312108
5.0530195	down	NM_053904	Oplah	116684
2.5166354	down	XM_001081327	Osbpl7	303497
22.28867	down	XM_001076555	Ostbeta	300790
9.324993	down	NM_013078	Otc	25611
6.8959436	down	XM_001053452	Otor	366206
3.1753423	down	XM_001071098	Otud3	500572
59.92183	down	XM_344878	Ovol3	365226
6.033452	down	NM_012996	Oxt	25504
6.67529	down	NM_057124	P2ry6	117264
3.339777	down	XM_001066817	P4ha3	361612

12.220164	down	XM_001070732	Pabpc1l	296351
28.4569	down	XM_001054954	Pabpc1l2b	302405
12.652932	down	XM_001069283	Pabpc2	291615
14.253769	down	NM_001077677	Pacrg	499021
2.1999106	down	NM_017230	Padi3	29520
9.006503	down	NM_017227	Padi4	29512
3.9393933	down	NM_053654	Pafah1b3	114113
2.226316	down	XM_001073872	Pak4	292756
16.64341	down	XM_001055668	Pald	294508
2.936134	down	XM_001071590	Pamr1	311252
2.4831288	down	XM_001080007	Pank1	294088
22.426128	down	XM_001069675	Papola	314417
2.8959854	down	XM_215701	Papss1	295443
4.446474	down	NM_001003654	Pard6a	307799
6.7981467	down	XM_001058462	Pate4	363041
2.3731909	down	NM_033485	Pawr	64513
5.9741836	down	XM_230663	Pax1	311505
22.453587	down	NM_013001	Pax6	25509
2.9925733	down	XM_001072018	Pbp2	246145
2.7421346	down	XM_001078717	Pbx3	311876
2.305115	down	XM_001071111	Pcbp4	363133
22.586628	down	XM_225997	Pcdh1	307481
7.2150474	down	XM_001075929	Pcdh15	690865
7.7654443	down	XM_001053200	Pcdh18	295027
2.474787	down	XM_001073680	Pcdh9	306091
59.294476	down	NM_199503	Pcdha1	393085
7.202784	down	NM_053934	Pcdha13	116742
36.784195	down	NM_053941	Pcdha3	116780
2.7711096	down	NM_053933	Pcdha4	116741
2.8015406	down	NM_199507	Pcdha7	393089
3.472972	down	NM_053942	Pcdha8	116781
4.7311187	down	NM_199508	Pcdha9	393090
2.8324132	down	XM_001055294	Pcdhb7	291652
4.3299155	down	XM_001055350	Pcdhb8	680020
5.8107195	down	XM_001055473	Pcdhb9	680047
2.7704694	down	NM_001037154	Pcdhga3	498847
8.287633	down	XM_001057332	Pcdhgb6	680470
2.0874474	down	NM_012746	Pcsk2	25121
3.7013707	down	XM_001078022	Pcsk5	116548
18.070192	down	NM_199253	Pcsk9	298296
2.1234815	down	XM_001059896	Pctk3	289019
2.6824584	down	NM_017229	Pde3b	29516
39.32133	down	NM_053688	Pde6h	114248
3.0616813	down	NM_080894	Pde7b	140929

2.5254345	down	XM_001064592	Pdgfrl	290771
9.431591	down	NM_053994	Pdha2	117098
3.8961494	down	NM_001007622	Pdlim2	290354
3.7662404	down	NM_173125	Pdlim7	286908
9.58353	down	NM_022852	Pdx1	29535
3.9760985	down	NM_022940	Pdzd2	65034
2.7008858	down	XM_001057829	Pdzd7	293996
12.038482	down	NM_130401	Pdzk1ip1	81916
8.904531	down	XM_218737	Peg12	308692
6.2150297	down	XM_001060915	Peli1	305549
11.94627	down	NM_017139	Penk	29237
2.1581643	down	NM_001033964	Pfkfb2	24640
5.3251343	down	NM_001025272	Pgam5	288731
2.5957355	down	NM_031640	Pgcp	58952
2.046237	down	NM_053373	Pglyrp1	84387
14.627596	down	XM_001057607	Pglyrp3	499658
13.851005	down	XM_001057786	Pglyrp3b	295180
2.4219863	down	XM_001076262	Phf13	313742
6.4624	down	NM_031620	Phgdh	58835
4.2446404	down	XM_001053345	Phka2	678739
8.1470995	down	XM_001076710	Phldb3	308431
2.1614215	down	NM_139093	Phrf1	245925
3.2643003	down	NM_001012076	Phyhipl	309901
7.5605035	down	XM_001078697	Pi16	294312
3.5001006	down	NM_001044253	Pif1	367645
6.638211	down	XM_001055659	Pign	309051
2.7746398	down	XM_001069442	Pigo	313341
6.88273	down	NM_053923	Pik3c2g	116720
2.6052332	down	XM_001070149	Pik3r4	363131
3.2385356	down	XM_213732	Pilra	288568
2.0454986	down	XM_001077069	Pin1	298696
9.432854	down	NM_001033970	Pip5k1c	314641
2.1407812	down	XM_001081613	Pitpnc1	498015
2.755407	down	XM_001080102	Pitpnm3	287467
27.25614	down	NM_019247	Pitx3	29609
24.82071	down	NM_001076553	Pkib	24678
11.3629055	down	XM_001063502	Pkp1	304822
3.5740001	down	XM_213560	Pkp2	287925
2.0092282	down	XM_001071062	Pla2g2e	298581
2.4126983	down	XM_001080878	Pla2g4b	311341
18.119612	down	XM_001080057	Pla2g4f	691907
2.737015	down	NM_001005560	Pla2g6	360426
12.9686775	down	NM_001024894	Plac1	317316
2.3503146	down	XM_001067597	Plac8	360914

2.4471757	down	NM_012760	Plagl1	25157
13.57903	down	NM_033350	Plcb3	29322
18.604471	down	XM_001080726	Plcxd1	304575
33.927887	down	XM_001077240	Pld6	287366
8.488635	down	XM_001060716	Plekha6	360842
7.277137	down	XM_001078528	Plekha7	499249
6.306791	down	NM_172033	Plekhb1	64471
4.643866	down	XM_217372	Plekhb2	301337
4.9833136	down	NM_001013148	Plekhf1	308543
2.5367658	down	XM_001061544	Plin5	501283
4.6595926	down	NM_017100	Plk1	25515
3.3765645	down	XM_001064185	Pnmal2	308393
2.4686265	down	XM_001081395	Pnmt	24661
3.7082758	down	NM_013007	Pnoc	25516
17.640524	down	XM_001062157	Pof1b	302328
6.0813518	down	XM_001066743	Polh	316235
2.138142	down	XM_001069113	Poli	291526
2.0042644	down	NM_001011912	Polm	289757
4.3958197	down	XM_001054408	Polr3f	311487
2.4130254	down	NM_053622	Pom121	113975
2.2330413	down	XM_001063037	Porcn	317368
2.7486234	down	XM_001075635	Pou2f1	171068
6.320027	down	XM_001059708	Pou2f3	116544
32.375816	down	NM_138838	Pou3f1	192110
7.596729	down	XM_001076964	Pou4f1	114503
3.8846295	down	XM_001069878	Pou4f3	364855
2.080026	down	NM_001081751	Pou5f2	680620
5.868182	down	XM_001055787	Pou6f2	681092
3.1260018	down	XM_219376	Ppapdc1a	309014
4.538859	down	NM_013196	Ppara	25747
3.6066928	down	NM_198773	Ppm1e	360593
10.9198065	down	NM_175755	Ppm1f	287931
6.9651375	down	NM_022951	Ppp1r10	65045
5.695155	down	XM_001081393	Ppp1r1b	360616
23.560825	down	XM_001067607	Ppp1r1c	499818
5.760769	down	NM_053473	Ppp1r9a	84685
7.9745193	down	NM_053474	Ppp1r9b	84686
2.8283496	down	NM_057116	Ppp2r2c	117256
2.648988	down	NM_181379	Ppp2r5b	309179
4.4033175	down	NM_012626	Ppy	24677
3.2419155	down	XM_001058767	Praf2	367743
2.8620205	down	XM_001073706	Pramef8	502994
5.7759123	down	NM_031669	Prap1	60574
49.070972	down	NM_172065	Prb1	257651

5.7704053	down	XM_001072014	Prdm5	689788
3.289785	down	XM_001062048	Prdm8	305198
5.098102	down	XM_001073957	Prickle2	312563
2.6343675	down	NM_001044271	Prl2c1	502122
10.329463	down	NM_001083940	Prl3d2	691552
2.2972157	down	NM_012630	Prlr	24684
7.2502575	down	NM_012873	Prm2	25345
3.4422045	down	XM_001081220	Prnd	113910
3.5020516	down	NM_138851	Prok1	192205
3.8127537	down	NM_001037541	Prok2	192206
25.589918	down	NM_133513	Prol1	65182
18.991482	down	NM_153627	Prop1	266738
3.3484735	down	NM_031086	Pros1	81750
3.1156158	down	NM_001013211	Prp2	362450
10.151052	down	XM_001081954	Prp2l2	685766
2.6919193	down	NM_172064	Prpg1	257652
3.3180492	down	NM_012634	Prps2	24689
2.1743994	down	XM_001057092	Prr16	361327
2.0368948	down	XM_001069759	Prr18	361481
4.1486883	down	XM_001059223	Prrg1	363472
14.520605	down	NM_012635	Prss1	24691
6.821976	down	NM_012729	Prss2	25052
2.8235133	down	NM_181477	Prss21	353251
2.1315222	down	NM_182949	Prss27	287108
12.082708	down	NM_173127	Prss3	286911
2.7724516	down	XM_001056587	Prss32	302970
18.41963	down	XM_001056464	Prss33	497873
3.4671662	down	XM_001070589	Prss37	362346
2.104812	down	NM_001037651	Prtg	315806
2.970147	down	NM_023976	Prx	78960
7.46312	down	XM_001070777	Psd3	306380
7.9907193	down	XM_001053201	Psd4	311785
15.170155	down	XM_001076864	Psx1	296535
4.718217	down	NM_022516	Ptbp1	29497
2.866434	down	NM_021583	Ptges	59103
4.535218	down	NM_017043	Ptgs1	24693
2.0660143	down	NM_017044	Pth	24694
5.1014633	down	NM_020073	Pth1r	56813
21.464485	down	NM_012636	Pthlh	24695
4.2418413	down	XM_217346	Ptk7	301242
3.6147985	down	NM_017066	Ptn	24924
3.4012816	down	XM_213610	Ptplb	288058
3.8493576	down	NM_019253	Ptpn5	29644
8.777158	down	NM_053908	Ptpn6	116689

5.7518516	down	NM_001024289	Ptprcap	499300
2.1514957	down	XM_001056164	Ptpre	114767
5.395697	down	NM_053881	Ptprn	116660
7.988881	down	XM_342568	Ptprt	362263
5.8229413	down	XM_001078427	Ptrh1	362113
3.583722	down	XM_001081808	Pycr1	287877
3.8234184	down	XM_001079880	Pygo1	691857
3.2256417	down	XM_001073224	Pygo2	295251
2.7420022	down	NM_001004234	Pyroxd1	297708
4.0223446	down	NM_001004261	Pyroxd2	309381
27.186008	down	NM_198200	Qrfp	379044
2.35342	down	XM_577148	Qrich2	501747
5.499126	down	XM_001080932	Rab11fip4	303337
4.508273	down	NM_198749	Rab15	299156
2.2561731	down	NM_001015012	Rab30	308821
2.1778595	down	XM_001054246	Rab33b	365793
3.2186224	down	XM_001072166	Rab39	315668
3.4658966	down	NM_080580	Rab3d	140665
3.7959797	down	NM_001024331	Rab43	500249
11.188545	down	NM_001024871	Rabepk	296649
4.2705836	down	XM_001061761	Rabl3	360720
4.32581	down	NM_001011902	Rabl5	288585
3.854416	down	XM_576058	Rad51l1	500679
4.6251354	down	XM_001080997	Rad51l3	303375
3.152398	down	NM_001037218	Radil	304299
29.7983	down	NM_001013063	Raet1l	292461
2.4684412	down	NM_031093	Rala	81757
3.9518383	down	NM_032067	Ralbp1	84014
14.095321	down	NM_020100	Ramp3	56820
7.539689	down	XM_001078472	Rap1gds1	310909
3.6528146	down	NM_053741	Rap2a	114560
2.484939	down	NM_031528	Rara	24705
5.53658	down	XM_001059459	Rarb	24706
2.5173442	down	XM_001065154	Rasgrp3	313874
5.3467813	down	XM_001081057	Rasl10b	303382
2.0657945	down	NM_022959	Rassf9	65053
3.679165	down	XM_001053414	Rbm12b	313069
3.576633	down	XM_222200	Rbm19	304512
4.614622	down	XM_001069277	Rbmx2	367930
4.1249423	down	XM_001053717	Rbpj	679028
15.954881	down	XM_001076681	Rbpms2	503214
6.349102	down	XM_001066157	Rccd1	308760
15.348485	down	XM_001073225	Rcn1	362182
10.56829	down	NM_001008694	Rcn3	494125

7.934746	down	NM_001013994	Rcor2	305811
27.501417	down	XM_341147	Rcsd1	360872
5.0580173	down	NM_133543	Rdh7	360420
3.8204246	down	XM_215383	Reep3	294375
2.5777626	down	NM_001013218	Reep6	362835
30.766945	down	NM_173097	Reg3g	24620
26.320063	down	NM_001025753	Rem1	366232
4.794569	down	NM_012642	Ren	24715
5.547248	down	NM_031095	Renbp	81759
7.037423	down	XM_001075300	Rep15	500366
5.6264935	down	XM_001080107	Rfc5	304528
3.507196	down	NM_001014106	Rfk	499328
2.422531	down	XM_001072525	Rfpl4a	292583
8.612277	down	XM_001055742	Rfx2	301121
17.877066	down	XM_001064554	RGD1304595	298104
2.2460823	down	XM_001079093	RGD1304624	314128
2.9440978	down	XM_001075066	RGD1304745	300381
3.1506963	down	NM_001014059	RGD1304952	312711
5.5491147	down	XM_342916	RGD1305090	362598
5.1905227	down	NM_001014267	RGD1305207	366990
6.621113	down	XM_001073162	RGD1305222	290686
10.070771	down	NM_001009659	RGD1305225	297458
4.0285764	down	XM_001066987	RGD1305274	298377
6.7902937	down	NM_001009430	RGD1305457	314730
5.2841063	down	NM_001025011	RGD1305464	315702
2.2593317	down	XM_001066163	RGD1305469	305466
8.466673	down	XM_217663	RGD1305500	308004
6.192475	down	XM_001063925	RGD1305560	301561
2.038791	down	NM_001024749	RGD1305713	293059
2.146931	down	XM_001057760	RGD1306119	314306
4.659745	down	XM_001080995	RGD1306282	314858
2.0994315	down	XM_001073356	RGD1306484	363594
2.0962615	down	XM_001069342	RGD1306526	310764
2.4179287	down	XM_001073260	RGD1306565	365057
2.9188154	down	XM_001068867	RGD1306576	298002
2.8235383	down	XM_001053501	RGD1306613	306994
11.364814	down	XM_001078017	RGD1306636	293498
5.375272	down	XM_215709	RGD1306704	295483
4.56712	down	NM_001044226	RGD1306772	288683
5.3250375	down	XM_001080215	RGD1306928	293509
6.016991	down	XM_001069247	RGD1306995	288094
5.3413677	down	XM_001073278	RGD1307220	315980
3.0488777	down	XM_001065607	RGD1307225	310269
8.877293	down	XM_001081575	RGD1307288	287761

3.9949622	down	XM_001075843	RGD1307357	291975
6.176463	down	XM_001054857	RGD1307365	300880
4.878087	down	XM_001070349	RGD1307443	361244
2.2318997	down	XM_215189	RGD1307603	293656
11.297961	down	XM_222746	RGD1307830	304863
33.622562	down	XM_342009	RGD1308106	361719
29.701212	down	XM_001062423	RGD1308112	289038
2.0487964	down	XM_001081031	RGD1308261	299818
4.16959	down	XM_237548	RGD1308319	316764
3.071924	down	XM_226525	RGD1308461	307901
3.2780008	down	XM_001059141	RGD1308612	311713
2.3556566	down	XM_236102	RGD1309108	315578
5.415354	down	NM_001034835	RGD1309220	316328
2.9716413	down	XM_001055914	RGD1309374	361485
2.2374732	down	XM_001057453	RGD1309403	310417
5.6425543	down	XM_347094	RGD1309410	363020
2.1060228	down	XM_001057186	RGD1309414	361004
2.2215242	down	XM_001055847	RGD1309453	310689
2.0382922	down	XM_001058624	RGD1309492	314330
2.2067235	down	XM_001066609	RGD1309586	364073
7.202855	down	XM_001078912	RGD1309651	361424
18.383722	down	XM_215788	RGD1309730	295952
3.9446533	down	XM_001075822	RGD1309735	360776
2.74383	down	XM_341726	RGD1309759	361448
3.3839571	down	XM_342856	RGD1309931	362538
9.742908	down	XM_001071523	RGD1309998	367088
2.6147585	down	XM_001078350	RGD1310212	299557
6.107417	down	XM_001064794	RGD1310384	289530
2.0115058	down	NM_001025666	RGD1310572	304646
3.1253002	down	XM_001071413	RGD1310727	363070
2.5800922	down	XM_001079537	RGD1310754	311264
2.3299797	down	NM_001024246	RGD1310794	296840
2.52296	down	NM_001025007	RGD1310852	314992
3.2819035	down	XM_001063794	RGD1310877	305667
4.7232237	down	XM_001056694	RGD1310951	313202
3.9639308	down	XM_001067680	RGD1310964	304376
3.042257	down	XM_001065822	RGD1311021	308765
6.1592636	down	NM_001037792	RGD1311122	364154
2.3973463	down	XM_001057068	RGD1311186	293587
14.920316	down	XM_001059245	RGD1311190	287130
3.216993	down	NM_001008307	RGD1311257	294333
16.475052	down	NM_001039024	RGD1311267	311429
4.8204484	down	NM_001030043	RGD1311300	364378
15.7222185	down	XM_001066764	RGD1311447	363276

2.1123624	down	XM_001078807	RGD1311501	296599
2.7659614	down	NM_001014072	RGD1311517	313775
4.789803	down	NM_001079705	RGD1311558	292139
2.8194854	down	XM_001059424	RGD1311783	294012
13.171824	down	NM_001007738	RGD1359349	361744
22.115452	down	XM_235207	RGD1559489	299843
20.37836	down	XM_579978	RGD1559494	499535
4.201423	down	XM_577489	RGD1559508	502056
12.63832	down	XM_001058189	RGD1559526	364929
14.674456	down	XM_001070396	RGD1559532	297671
3.2614403	down	XM_001060667	RGD1559582	501852
5.430101	down	XM_001075881	RGD1559588	304349
4.976366	down	XM_229219	RGD1559599	317624
17.043726	down	XM_001068894	RGD1559613	499067
31.085983	down	XM_225620	RGD1559615	291346
19.396112	down	XM_577371	RGD1559628	501945
14.256798	down	XM_001054198	RGD1559643	498100
5.4342914	down	XM_578466	RGD1559679	502961
57.55036	down	XM_344831	RGD1559699	365132
14.106077	down	XM_001078945	RGD1559724	299021
3.221514	down	XM_001057862	RGD1559731	501512
7.317103	down	XM_001054410	RGD1559747	312310
6.589252	down	XM_001058285	RGD1559751	498895
2.8921418	down	XM_001059587	RGD1559786	298384
36.243484	down	XM_227432	RGD1559837	295256
30.881979	down	XM_347117	RGD1559859	363244
3.4665358	down	XM_573162	RGD1559875	497970
8.019896	down	XM_001071693	RGD1559892	367788
5.528505	down	XM_001054336	RGD1559896	498967
4.3481317	down	XM_001080109	RGD1559903	502418
3.5795257	down	XM_001053130	RGD1559904	313061
27.36057	down	XM_001055068	RGD1559932	298221
39.99376	down	XM_344837	RGD1559946	365141
5.3558583	down	XM_001065029	RGD1559950	498686
49.110302	down	XM_576496	RGD1559960	501084
2.8142009	down	XM_001074365	RGD1559962	498988
2.5383544	down	XM_001080931	RGD1559980	499158
26.933083	down	XM_001058237	RGD1559982	293850
53.000248	down	XM_001061596	RGD1560019	367121
2.028903	down	XM_001066502	RGD1560028	500157
59.589725	down	XM_001058468	RGD1560060	501598
5.43044	down	XM_001075637	RGD1560069	364450
78.82594	down	XM_574777	RGD1560095	499454
34.243793	down	XM_001054032	RGD1560096	310590

6.2284083	down	XM_001053693	RGD1560109	310597
3.1290843	down	XM_575275	RGD1560115	499930
26.273788	down	XM_001080514	RGD1560125	360825
62.16751	down	XM_574775	RGD1560135	499452
76.74227	down	XM_577949	RGD1560140	502469
3.8940368	down	XM_001059094	RGD1560146	500505
29.988367	down	XM_001057615	RGD1560185	502880
3.1302636	down	XM_001071412	RGD1560187	362641
59.010967	down	XM_344641	RGD1560209	364800
3.6260898	down	XM_001063983	RGD1560218	501777
42.706245	down	XM_574066	RGD1560221	498780
29.402885	down	XM_001062614	RGD1560224	500608
18.697388	down	XM_574986	RGD1560232	499667
7.9487257	down	XM_001066238	RGD1560248	499797
41.66997	down	XM_578421	RGD1560270	502920
4.803658	down	XM_001068602	RGD1560273	498405
17.388441	down	XM_001080117	RGD1560350	365554
3.2883391	down	XM_001081416	RGD1560383	497994
4.9119673	down	XM_001080358	RGD1560398	498192
7.0216565	down	XM_577218	RGD1560412	501808
2.4060862	down	XM_001074947	RGD1560474	501814
2.0230834	down	XM_001064461	RGD1560481	500409
3.151265	down	XM_231013	RGD1560483	296516
65.685295	down	XM_579915	RGD1560485	499053
40.340405	down	XM_579909	RGD1560516	499038
3.485807	down	XM_578481	RGD1560532	502975
33.599075	down	XM_001055007	RGD1560539	502966
47.563976	down	XM_574327	RGD1560543	499046
29.02389	down	XM_578061	RGD1560554	502576
73.217545	down	XM_577908	RGD1560555	502426
48.177166	down	XM_229665	RGD1560570	302091
56.189426	down	XM_001078732	RGD1560580	288646
52.326912	down	XM_001054843	RGD1560585	317597
14.34938	down	XM_577379	RGD1560592	501952
17.713291	down	XM_344921	RGD1560663	365308
16.60195	down	XM_573890	RGD1560677	498612
6.8873496	down	XM_001074018	RGD1560695	365063
5.017714	down	XM_001071572	RGD1560716	298444
18.846113	down	XM_001053122	RGD1560771	498511
49.06948	down	XM_577919	RGD1560777	502438
12.555094	down	XM_580207	RGD1560792	501530
19.055119	down	XM_001066194	RGD1560803	501583
14.911703	down	XM_225502	RGD1560805	291269
63.905495	down	XM_577734	RGD1560808	502273

23.71019	down	XM_576015	RGD1560812	500640
64.48377	down	XM_001065293	RGD1560825	685793
3.2418652	down	XM_001075200	RGD1560826	295423
2.8218403	down	XM_001056252	RGD1560842	299415
4.411252	down	XM_573350	RGD1560888	498140
65.70486	down	XM_001074010	RGD1560918	364248
2.9968967	down	XM_001079189	RGD1560961	311241
2.4877234	down	XM_001076947	RGD1560962	503190
47.218452	down	XM_577704	RGD1560972	502244
2.2926927	down	XM_001067264	RGD1561029	304252
3.576613	down	XM_001070819	RGD1561034	308908
3.0937843	down	XM_001062296	RGD1561065	317425
55.07478	down	XM_001056240	RGD1561089	499664
5.4729805	down	XM_228749	RGD1561106	302548
10.17589	down	XM_575113	RGD1561113	499780
16.993826	down	XM_577620	RGD1561114	502163
3.5864298	down	XM_001076251	RGD1561143	304382
2.4055662	down	XM_001057166	RGD1561150	298241
15.657955	down	XM_577741	RGD1561155	502280
2.1371028	down	XM_001056166	RGD1561157	360487
61.69936	down	XM_001054260	RGD1561185	502251
3.2413187	down	XM_001056406	RGD1561206	308678
41.63887	down	XM_576388	RGD1561216	500978
2.1801636	down	XM_001061341	RGD1561229	502492
79.80251	down	XM_001081347	RGD1561231	291345
4.144175	down	XM_001070211	RGD1561232	498086
4.6632333	down	XM_001053102	RGD1561246	502959
14.863697	down	XM_001080261	RGD1561251	499352
31.598806	down	XM_217531	RGD1561273	302093
4.44891	down	XM_001057102	RGD1561282	499958
7.406904	down	XM_220927	RGD1561285	303555
21.105354	down	XM_580123	RGD1561305	500808
29.618258	down	XM_001074856	RGD1561311	501693
18.76211	down	XM_001074083	RGD1561314	502487
3.5408733	down	XM_001066702	RGD1561318	317568
61.382942	down	XM_577732	RGD1561339	502271
27.28675	down	XM_001058390	RGD1561341	502769
25.977541	down	XM_001054863	RGD1561388	499042
85.89052	down	XM_574763	RGD1561401	499440
6.620966	down	XM_235208	RGD1561410	299844
2.1156228	down	XM_001074420	RGD1561412	498490
6.6696377	down	XM_001075863	RGD1561415	498943
71.31637	down	XM_577708	RGD1561446	502249
70.94777	down	XM_574061	RGD1561493	498775

3.0001743	down	XM_001077812	RGD1561507	292078
5.898966	down	XM_001070135	RGD1561527	301282
2.8145676	down	XM_578218	RGD1561533	502719
78.48385	down	XM_229383	RGD1561546	301899
38.016487	down	XM_345090	RGD1561557	365547
2.2979007	down	XM_001075737	RGD1561589	305284
4.624073	down	XM_001067850	RGD1561620	291274
34.581966	down	XM_001055578	RGD1561627	291595
45.60873	down	XM_001079552	RGD1561654	295557
32.469833	down	XM_344647	RGD1561656	364810
29.189262	down	XM_001054195	RGD1561667	308209
2.318902	down	XM_001053081	RGD1561680	361140
47.217716	down	XM_577125	RGD1561684	501721
3.433984	down	XM_001060826	RGD1561694	498388
50.157055	down	XM_001064028	RGD1561705	499290
33.485363	down	XM_344825	RGD1561706	365122
10.777372	down	XM_001076037	RGD1561730	498169
52.509365	down	XM_217910	RGD1561759	308183
4.478844	down	XM_001077483	RGD1561787	364149
4.837485	down	XM_578508	RGD1561813	502998
3.2104955	down	XM_001058905	RGD1561819	502004
16.551146	down	XM_001079665	RGD1561827	502960
21.114765	down	XM_001053629	RGD1561828	679595
3.954152	down	XM_001080370	RGD1561832	499143
79.16665	down	XM_346315	RGD1561834	367829
2.9527893	down	XM_218061	RGD1561843	308263
11.945449	down	XM_344287	RGD1561956	364242
11.890718	down	XM_001053365	RGD1561975	291188
19.73735	down	XM_001078624	RGD1561977	501903
2.7247694	down	XM_001066085	RGD1562011	499297
29.688736	down	XM_577621	RGD1562019	502164
7.8675423	down	XM_001057648	RGD1562035	290247
27.537235	down	XM_001059310	RGD1562043	502083
57.736885	down	XM_577947	RGD1562058	502467
2.0715199	down	XM_001053262	RGD1562059	361993
4.338214	down	XM_001075004	RGD1562063	500896
3.937947	down	XM_001076742	RGD1562114	500795
34.22749	down	XM_221091	RGD1562156	501740
2.814241	down	XM_001058636	RGD1562200	363471
37.220345	down	XM_001076534	RGD1562205	307168
55.84618	down	XM_578760	RGD1562282	367183
2.3975215	down	XM_001058420	RGD1562284	313837
46.39317	down	XM_001074199	RGD1562287	300026
5.5959725	down	XM_001071441	RGD1562342	500762

9.79345	down	XM_574072	RGD1562352	498787
3.6733363	down	XM_001053073	RGD1562415	303862
2.4687512	down	XM_573791	RGD1562422	498522
4.8130093	down	XM_577326	RGD1562423	501902
24.828718	down	XM_001068716	RGD1562428	296323
10.083498	down	XM_001079744	RGD1562446	499344
3.2372632	down	XM_001059208	RGD1562449	366333
11.607934	down	XM_001071739	RGD1562454	292938
18.002138	down	XM_229083	RGD1562485	302772
3.8488553	down	XM_001071419	RGD1562489	287250
30.472868	down	XM_001064042	RGD1562508	502058
10.11852	down	XM_344096	RGD1562525	363899
42.791092	down	XM_001061198	RGD1562545	365857
26.976238	down	XM_001067081	RGD1562546	364953
7.2334976	down	XM_574124	RGD1562552	498840
2.5310793	down	XM_001068226	RGD1562582	499935
29.321598	down	XM_577381	RGD1562612	501954
20.932053	down	XM_001072696	RGD1562618	501007
22.7292	down	XM_001071626	RGD1562624	364249
30.393906	down	XM_578244	RGD1562640	502745
34.145683	down	XM_001076214	RGD1562649	363887
36.861694	down	XM_001054943	RGD1562650	502965
11.971132	down	XM_001081656	RGD1562667	501742
61.589317	down	XM_577950	RGD1562670	502470
6.64562	down	XM_222212	RGD1562674	288691
54.84976	down	XM_344833	RGD1562677	365136
47.04084	down	XM_001055582	RGD1562704	499665
61.99517	down	XM_574093	RGD1562707	498809
41.612576	down	XM_576389	RGD1562714	500979
6.849479	down	XM_001073387	RGD1562726	498060
11.082717	down	XM_001068930	RGD1562739	500875
5.698164	down	XM_223623	RGD1562776	289795
6.001007	down	XM_001073886	RGD1562802	502505
4.96522	down	XM_580242	RGD1562804	502222
31.06307	down	XM_001071282	RGD1562811	315651
5.172605	down	XM_001080078	RGD1562814	366866
12.566223	down	XM_001070013	RGD1562853	501889
57.126648	down	XM_216073	RGD1562877	296780
5.9445853	down	XM_001055250	RGD1562882	503195
5.1250477	down	XM_001073084	RGD1562885	502412
24.388683	down	XM_001068190	RGD1562886	363636
76.1431	down	XM_577935	RGD1562893	502454
47.217	down	XM_577932	RGD1562919	502451
66.6423	down	XM_001071176	RGD1562931	502439

5.187207	down	XM_577316	RGD1562932	501893
46.0796	down	XM_344812	RGD1562948	365096
18.638279	down	XM_001060329	RGD1562960	503057
3.2396576	down	XM_001071898	RGD1562978	502911
6.3321414	down	XM_001063038	RGD1562980	302448
2.7052796	down	XM_001069698	RGD1563100	299964
67.44017	down	XM_579906	RGD1563107	499034
105.10544	down	XM_001081417	RGD1563149	502466
4.3959126	down	XM_001064380	RGD1563154	296788
12.225904	down	XM_001079515	RGD1563222	362175
4.033698	down	XM_001068872	RGD1563224	500874
58.781284	down	XM_344630	RGD1563247	364781
9.12067	down	XM_576197	RGD1563259	500809
3.677244	down	XM_001079457	RGD1563325	362857
10.779356	down	XM_001078529	RGD1563350	293963
3.7495496	down	XM_001068469	RGD1563375	366392
14.5901985	down	XM_001059106	RGD1563378	302719
6.5077806	down	XM_001063679	RGD1563425	498129
6.268182	down	XM_235209	RGD1563427	299845
7.1318703	down	XM_001065559	RGD1563440	298301
10.098069	down	XM_227852	RGD1563455	311003
2.5328407	down	XM_577328	RGD1563483	501904
30.507233	down	XM_001068047	RGD1563486	367926
32.719433	down	XM_574318	RGD1563490	499025
24.957422	down	XM_342547	RGD1563510	362240
3.7923858	down	XM_574297	RGD1563545	499004
7.7652183	down	XM_001081444	RGD1563552	503129
8.918577	down	XM_001064617	RGD1563606	367749
8.886067	down	XM_001055699	RGD1563622	502165
2.0573058	down	XM_001078978	RGD1563625	503124
63.178963	down	XM_344827	RGD1563626	365125
24.69635	down	XM_001053995	RGD1563667	365855
5.5500593	down	XM_001068301	RGD1563703	361206
72.80935	down	XM_001080342	RGD1563723	502462
20.401329	down	XM_001066716	RGD1563727	498080
49.786144	down	XM_345920	RGD1563738	367059
49.335907	down	XM_577701	RGD1563741	502241
75.868195	down	XM_001081170	RGD1563747	499439
30.89371	down	XM_214327	RGD1563748	290675
5.6518607	down	XM_001076316	RGD1563757	367030
79.03987	down	XM_577939	RGD1563772	502458
7.6924124	down	XM_001062651	RGD1563782	498115
17.998432	down	XM_240367	RGD1563825	306366
62.194412	down	XM_577929	RGD1563833	502448

55.103424	down	XM_343520	RGD1563862	363180
55.84555	down	XM_344835	RGD1563865	365139
2.021885	down	XM_580106	RGD1563913	500691
2.898771	down	XM_001072488	RGD1563937	364539
4.3278165	down	XM_345580	RGD1563973	366466
34.669952	down	XM_236920	RGD1563979	316212
69.45059	down	XM_577705	RGD1564000	502246
29.340425	down	XM_001056054	RGD1564016	498969
65.720665	down	XM_001059672	RGD1564033	680963
2.6831608	down	XM_001071594	RGD1564036	497895
6.193224	down	XM_001062502	RGD1564040	313410
25.890831	down	XM_344095	RGD1564052	363898
2.3824985	down	XM_001065792	RGD1564053	500390
2.0662525	down	XM_001075668	RGD1564067	499401
16.189354	down	XM_001069553	RGD1564069	305202
2.6903217	down	XM_001073215	RGD1564081	500943
3.9311976	down	XM_001078704	RGD1564086	497862
9.80291	down	XM_001067611	RGD1564095	500396
9.561854	down	XM_001070381	RGD1564102	501895
29.882528	down	XM_001055303	RGD1564109	500115
83.38731	down	XM_574773	RGD1564135	499450
4.9520006	down	XM_573858	RGD1564142	498582
17.105919	down	XM_001053552	RGD1564173	367947
3.6851697	down	XM_001078254	RGD1564195	361651
42.521904	down	XM_573954	RGD1564205	498671
14.548396	down	XM_577618	RGD1564261	502161
6.3232946	down	XM_001072962	RGD1564268	300001
9.663855	down	XM_577533	RGD1564276	502093
29.265394	down	XM_001079282	RGD1564285	299662
62.51689	down	XM_001080768	RGD1564288	302278
47.602978	down	XM_001070096	RGD1564295	317585
5.857145	down	XM_001053680	RGD1564300	361661
26.59502	down	XM_578051	RGD1564313	502566
76.85075	down	XM_001072043	RGD1564317	498054
31.997917	down	XM_001055123	RGD1564321	500114
10.511169	down	XM_344622	RGD1564347	364764
95.777824	down	XM_001078658	RGD1564355	307681
9.351596	down	XM_577770	RGD1564357	502306
2.8826091	down	XM_573549	RGD1564364	498325
13.264487	down	XM_344301	RGD1564372	364258
11.9525385	down	XM_001067675	RGD1564406	501586
5.519887	down	XM_234980	RGD1564409	314670
3.4778469	down	XM_575480	RGD1564419	500128
2.3819134	down	XM_001075226	RGD1564421	498939

2.066299	down	XM_001058987	RGD1564447	367254
5.3711905	down	XM_001068812	RGD1564477	498882
3.1933713	down	XM_001063065	RGD1564519	498072
3.3470428	down	XM_577205	RGD1564563	501796
72.10692	down	XM_574764	RGD1564568	499441
4.585708	down	XM_001075395	RGD1564575	501056
2.806011	down	XM_001075441	RGD1564634	294052
10.204957	down	XM_001064059	RGD1564645	300874
70.69095	down	XM_573610	RGD1564665	498375
8.938612	down	XM_001062492	RGD1564666	499627
87.96133	down	XM_574778	RGD1564734	499455
43.498753	down	XM_001070478	RGD1564736	367932
36.29862	down	XM_579977	RGD1564743	499534
40.24581	down	XM_001064846	RGD1564763	367747
4.109993	down	NM_001024282	RGD1564767	498753
20.983784	down	XM_574052	RGD1564791	498766
7.6717777	down	XM_001061968	RGD1564792	499976
36.009716	down	XM_001065957	RGD1564798	502726
23.951017	down	XM_577315	RGD1564814	501892
4.8693624	down	XM_001054151	RGD1564824	310382
49.430393	down	XM_001071767	RGD1564836	302676
9.020917	down	XM_001061065	RGD1564843	302765
25.385649	down	XM_001056625	RGD1564844	301172
83.0519	down	XM_577726	RGD1564848	502265
7.523262	down	XM_001066084	RGD1564877	501581
12.717138	down	XM_001067005	RGD1564927	499929
2.8376765	down	XM_001070908	RGD1564937	500995
8.733192	down	XM_344499	RGD1564941	364538
62.85847	down	XM_001076029	RGD1564977	502457
3.401564	down	XM_001061359	RGD1564989	499550
35.331142	down	XM_001069747	RGD1564998	498323
5.849451	down	XM_001066828	RGD1565014	290790
5.0152664	down	XM_001074859	RGD1565043	306618
5.4491315	down	XM_001068189	RGD1565058	501587
3.6842685	down	XM_001076350	RGD1565088	502595
120.91681	down	XM_001071285	RGD1565094	502437
51.895752	down	XM_001069114	RGD1565104	498924
77.307976	down	XM_001080335	RGD1565115	499449
44.809067	down	XM_001055593	RGD1565117	299848
4.5671453	down	XM_001077736	RGD1565158	288959
2.1082864	down	XM_001053261	RGD1565159	310365
12.146149	down	XM_001053795	RGD1565164	361582
2.775423	down	XM_001079046	RGD1565166	287059
63.821968	down	XM_001075702	RGD1565191	499451

8.108523	down	XM_343847	RGD1565203	363523
27.404728	down	XM_574322	RGD1565209	499033
79.47711	down	XM_001054671	RGD1565231	365140
6.8028736	down	XM_001059320	RGD1565257	311115
49.802803	down	XM_344642	RGD1565321	364801
11.694885	down	XM_231601	RGD1565367	312226
22.549719	down	XM_001070230	RGD1565372	366307
2.2233965	down	XM_001058473	RGD1565374	498128
62.571712	down	XM_001054516	RGD1565379	498770
45.861263	down	XM_344843	RGD1565390	365149
4.9631248	down	XM_001081318	RGD1565395	303471
7.047041	down	XM_343330	RGD1565411	363000
48.750904	down	XM_576498	RGD1565421	501086
42.48444	down	XM_343720	RGD1565423	363385
34.24915	down	XM_001068143	RGD1565430	367928
26.424084	down	XM_001063293	RGD1565469	502584
2.0479853	down	XM_001056411	RGD1565472	498067
3.941963	down	XM_001058342	RGD1565514	317614
3.3516192	down	XM_001074987	RGD1565536	311323
65.35155	down	XM_574776	RGD1565550	499453
64.34743	down	XM_577941	RGD1565576	502460
2.4201868	down	XM_001055923	RGD1565598	502118
4.944102	down	XM_580086	RGD1565622	500582
2.914194	down	XM_575255	RGD1565648	499911
7.3410726	down	XM_001076237	RGD1565653	367176
9.997885	down	XM_001070115	RGD1565660	501896
6.989474	down	XM_576548	RGD1565676	501128
6.3610916	down	XM_577714	RGD1565723	502254
8.04413	down	XM_001076528	RGD1565742	295470
2.7143092	down	XM_001059479	RGD1565784	497874
2.6333387	down	XM_001061435	RGD1565785	501570
2.9411497	down	XM_001080115	RGD1565800	304529
3.5099952	down	XM_573888	RGD1565815	498610
71.293976	down	XM_001063482	RGD1565859	499291
2.762488	down	XM_235047	RGD1565866	314740
25.280767	down	XM_233160	RGD1565911	298220
2.4472566	down	XM_346994	RGD1565926	361629
2.3409593	down	XM_001065138	RGD1565975	291903
6.3541884	down	NM_001079892	RGD1565983	317628
47.598984	down	XM_001053399	RGD1566008	317604
3.7309668	down	XM_001067260	RGD1566010	499195
22.723927	down	XM_577863	RGD1566018	502386
69.881966	down	XM_344619	RGD1566040	364759
30.169085	down	XM_574062	RGD1566047	498776

2.0214705	down	XM_001080280	RGD1566059	499858
2.7026806	down	XM_001079270	RGD1566085	361819
23.521967	down	XM_001081902	RGD1566091	311772
3.7045357	down	XM_001060048	RGD1566107	500131
3.696241	down	XM_001073756	RGD1566132	502360
14.766973	down	XM_001065548	RGD1566175	502142
15.794219	down	XM_001070155	RGD1566186	288313
2.7823706	down	XM_001079348	RGD1566207	366139
39.60701	down	XM_001069834	RGD1566225	317584
3.9036217	down	XM_001065647	RGD1566243	500992
12.402121	down	XM_234969	RGD1566251	299668
6.7879534	down	XM_001067237	RGD1566252	366937
9.980073	down	XM_001068981	RGD1566264	366999
7.7366743	down	XM_346024	RGD1566272	367226
13.026837	down	XM_577997	RGD1566273	502513
2.6365948	down	XM_001078793	RGD1566281	364480
41.24105	down	XM_001056130	RGD1566311	500963
2.1646767	down	XM_001067695	RGD1566314	288106
23.51745	down	XM_001054086	RGD1566337	310589
14.945956	down	XM_001058667	RGD1566353	502051
26.707146	down	XM_580152	RGD1566370	501019
8.972131	down	XM_001062354	Rgma	308739
6.3643765	down	XM_573061	Rgs11	54291
2.345901	down	NM_001077589	Rgs16	360857
4.6970787	down	NM_017214	Rgs4	29480
3.719243	down	NM_001012347	Rgs7bp	294715
14.255748	down	NM_182825	Rhebl1	359959
2.1401496	down	NM_001013430	Rhoh	305341
22.348547	down	NM_001024873	Rhox11	298346
3.744244	down	NM_001024889	Rhox4g	313451
11.489252	down	NM_001025776	Rhox8	503423
4.1163797	down	NM_001024874	Rhox9	298352
4.910295	down	XM_001069922	Rhpn1	300030
6.927771	down	NM_001013949	Ribc2	300122
2.2463102	down	XM_001072861	Ric3	687147
51.272144	down	XM_001064091	Rimbp3	303785
3.6682599	down	XM_001060530	Rimklb	362428
22.911516	down	NM_145881	Rims2	116839
2.312433	down	NM_139342	Ripk3	246240
14.411763	down	NM_001029904	Rnase1	364304
15.542345	down	NM_001025116	Rnase1l2	305844
5.8001237	down	NM_001007015	Rnase2	474169
4.0849333	down	XM_001057893	Rnf144b	364681
4.2224913	down	XM_001067775	Rnf150	364983

22.232473	down	XM_001053340	Rnf20	313216
7.411403	down	XM_001079113	Rnf222	363627
2.912643	down	NM_001012095	Rnf32	311936
2.679267	down	NM_001024795	Rnf44	361212
3.678659	down	XM_001076855	Rnft2	304521
6.1842585	down	NM_022188	Robo1	58946
3.5480654	down	NM_181375	Robo4	300518
6.341256	down	XM_001053288	Ror2	306782
8.211432	down	XM_001066326	Rp1l1	289463
2.937693	down	XM_001074462	Rp9	363032
21.988493	down	NM_133518	Rph3a	171039
26.420914	down	XM_001060195	Rpl21-ps2	681072
14.060911	down	XM_001077212	Rpl29-ps2	691206
26.920214	down	XM_001078234	Rps16	140655
2.5962539	down	XM_001057290	Rps6kl1	299202
2.4815412	down	XM_001081775	Rptor	287871
4.2120037	down	NM_001009702	Rrp15	360895
17.374216	down	NM_001024748	Rshl1	292684
6.0081654	down	NM_001013867	Rsph10b	288478
4.6214314	down	XM_001062232	Rspo2	500863
14.16391	down	XM_575261	Rspo4	499918
3.1972642	down	NM_001014172	Rsrc1	361956
9.202664	down	NM_001008830	RT1-A3	309627
2.4003894	down	NM_001008832	RT1-CE1	309603
4.6786933	down	NM_001008833	RT1-CE10	414792
8.205629	down	NM_001008834	RT1-CE11	414791
3.7835855	down	NM_001008836	RT1-CE13	414790
9.946089	down	NM_001008837	RT1-CE14	414270
2.0771928	down	NM_001008838	RT1-CE15	414789
11.320853	down	NM_001008842	RT1-CE4	414783
3.186575	down	NM_001008845	RT1-CE7	368153
6.082417	down	NM_001008856	RT1-O1	24751
2.1627822	down	NM_001008857	RT1-S2	24994
2.3332803	down	XM_001073390	Rufy3	360921
3.165538	down	XM_001053893	Runx1t1	362489
30.459314	down	NM_001034950	Rup2	619560
2.6656642	down	NM_001008310	Rxfp3	294807
3.4385579	down	XM_232220	Rybp	312603
12.818985	down	NM_053681	S100a3	114216
2.024448	down	NM_053587	S100a9	94195
16.588533	down	NM_013191	S100b	25742
34.22198	down	XM_001067777	S100z	294661
3.1979976	down	XM_001059195	Sap130	307527
16.527136	down	XM_001061870	Satl1	302325

2.674293	down	NM_001044231	Sbsn	292793
2.612256	down	XM_001059643	Scamp1	29521
3.8340788	down	XM_342279	Scamp3	65169
6.638437	down	XM_001066597	Scand1	362252
5.9634256	down	NM_139192	Scd1	246074
6.3547745	down	NM_001007755	Scly	363285
4.3507943	down	XM_001078257	Scn10a	29571
2.7648692	down	NM_030875	Scn1a	81574
3.456404	down	NM_139097	Scn3b	245956
4.06827	down	NM_013125	Scn5a	25665
4.263318	down	NM_013026	Sdc1	25216
15.340193	down	NM_053893	Sdc3	116673
5.930665	down	XM_001081634	Sdk2	360652
2.2853909	down	NM_001007712	Sdpr	316384
20.858728	down	XM_001067215	Sec14l4	498399
2.205835	down	XM_001078406	Sec16a	114089
10.549559	down	NM_001047921	Sec61g-ps1	367253
2.1088905	down	NM_019177	Sell	29259
2.1068206	down	NM_017310	Sema3a	29751
13.356896	down	XM_001064828	Sema3e	296789
12.357933	down	XM_001065817	Sema4b	293042
2.1557467	down	XM_001053912	Sema4d	306790
2.414029	down	XM_001066339	Sema5a	310207
9.356468	down	NM_017308	Sema6c	29744
96.20738	down	NM_001002833	Senp17	408216
3.186419	down	NM_022519	Serpina1	24648
6.965761	down	XM_001067371	Serpina9	299274
2.1964931	down	XM_001056687	Serpinb3a	498209
4.300026	down	NM_012620	Serpine1	24617
5.451405	down	XM_001069683	Sertad4	360899
20.499249	down	XM_001078230	Sez6l	304554
12.967701	down	NM_001011986	Sf3a2	299620
4.328619	down	XM_001063601	Sfi1	305467
4.390878	down	XM_001058167	Sgip1	313413
7.4653945	down	NM_139184	Sgp158	245985
2.894941	down	NM_173116	Sgpl1	286896
7.9632545	down	XM_001080492	Sgsm1	288743
3.4384158	down	NM_031621	Sh2b3	58838
2.4967134	down	NM_001048180	Sh2bpsm1	89817
3.0006394	down	NM_207605	Sh2d2a	310688
15.998186	down	XM_001062584	Sh2d4b	290612
2.0538917	down	XM_236252	Sh2d7	300718
4.601962	down	NM_022693	Sh3bp4	64634
9.623292	down	XM_001074803	Sh3bp5l	690898

10.625829	down	NM_031238	Sh3gl3	81921
2.4867697	down	XM_001054057	Sh3md4	294557
27.553213	down	XM_001072907	Sh3yl1	362724
7.380468	down	XM_001061022	Shcbp1	364648
4.905348	down	XM_001062249	She	685088
4.468835	down	XM_342507	Shf	362205
3.1051915	down	XM_001078662	Shisa6	497926
4.554388	down	NM_013028	Shox2	25546
3.504356	down	NM_001033682	Shpk	287479
2.4554617	down	NM_021693	Sik1	59329
2.3934352	down	XM_001065493	Sim1	309888
3.5749335	down	XM_001062980	Sin3b	683381
18.494488	down	XM_345630	Six2	366542
6.389035	down	XM_001075407	Six5	308406
19.673355	down	NM_173311	Skap1	286975
10.594855	down	XM_001054873	Slamf1	498286
23.399157	down	XM_222909	Slamf8	289237
4.0223365	down	XM_001060131	Slbp	681062
3.710918	down	NM_001010948	Slc10a7	291942
2.55904	down	NM_001031658	Slc11a1	316519
3.847254	down	NM_019345	Slc12a3	54300
2.135007	down	XM_001066648	Slc12a6	691209
2.3651917	down	NM_001012621	Slc13a4	503568
13.607092	down	NM_147216	Slc16a2	259248
9.118868	down	NM_001030024	Slc19a2	289175
5.5625196	down	XM_001077404	Slc22a14	316061
8.890682	down	NM_001004260	Slc22a18	309131
12.633112	down	NM_053537	Slc22a7	89776
6.762331	down	NM_020090	Slc24a1	56814
3.8748274	down	NM_001017488	Slc24a6	498185
9.805252	down	NM_017307	Slc25a1	29743
2.76666	down	XM_001080562	Slc25a16	361836
3.3412764	down	NM_053500	Slc25a27	85262
2.0518467	down	XM_001078825	Slc25a35	497933
2.305566	down	XM_001070712	Slc25a42	689414
2.5740523	down	XM_001071416	Slc25a45	689625
3.7492485	down	XM_001055712	Slc26a10	366909
3.1816266	down	NM_024143	Slc27a5	79111
2.4221232	down	NM_031782	Slc32a1	83612
5.816059	down	NM_013030	Slc34a1	25548
2.4192822	down	XM_001059113	Slc35d2	290959
5.499409	down	XM_001067921	Slc35e1	498599
3.1714897	down	NM_001024764	Slc36a3	303148
3.6065927	down	XM_001081800	Slc38a10	303740

2.9130175	down	NM_001013099	Slc38a6	299139
2.3821173	down	NM_001003705	Slc38a7	291840
2.6995747	down	XM_001075087	Slc39a1	361986
4.167592	down	XM_001070144	Slc39a14	306009
8.934229	down	XM_344140	Slc41a1	363985
2.2551084	down	XM_001077428	Slc44a2	363024
41.26858	down	XM_001077125	Slc5a10	303205
6.6926985	down	NM_022590	Slc5a2	64522
22.426277	down	NM_001033079	Slc6a17	613226
6.9344583	down	NM_053996	Slc6a7	117100
4.053668	down	NM_017353	Slc7a5	50719
5.350253	down	XM_001074601	Slc7a6	307811
3.595406	down	NM_078620	Slc8a3	140448
3.5264657	down	NM_173098	Slc9a4	24785
7.2894835	down	NM_022667	Slco2a1	24546
3.3205109	down	NM_001014270	Slco6d1	367321
4.067201	down	XM_001057409	Slitrk2	309349
5.439242	down	NM_053372	Slpi	84386
17.372597	down	XM_001073602	Slurp1	300016
2.3912158	down	NM_031583	Smc3	29486
15.3255825	down	NM_031536	Smcp	24899
3.507605	down	XM_001077659	Smcr8	497918
17.643684	down	XM_217877	Smok2a	292316
2.2448044	down	NM_001017497	Smr3a	498341
4.5935235	down	NM_030991	Snap25	25012
7.568907	down	NM_080777	Sncb	113893
4.9012475	down	XM_001068874	Sned1	316638
3.0875938	down	XM_001062546	Snhg8	361111
4.9521627	down	NM_019378	Snip	56029
29.195969	down	NM_001034083	Snn	29140
3.7222118	down	NM_138833	Snrk	170837
2.740366	down	XM_001064438	Snrpe	685587
4.744989	down	XM_001065908	Snrpel	360844
3.2504313	down	XM_001058102	Snrpg	681031
3.8337493	down	XM_001075060	Snrpgl2	690640
3.50479	down	XM_001060066	Snrpg-ps1	680779
14.423381	down	XM_001071003	Sntb2	689421
4.080997	down	XM_001066932	Sntg1	500394
5.950488	down	NM_153728	Soat2	266770
2.3025825	down	XM_001081372	Socs7	287659
2.5037823	down	XM_001078103	Sohlh1	362085
11.005976	down	XM_001070775	Sorcs1	309533
7.618917	down	NM_153737	Sostdc1	266803
14.812423	down	NM_019193	Sox10	29361

4.310573	down	XM_001060642	Sox13	289026
6.8710675	down	XM_344594	Sox4	364712
9.327687	down	XM_001081628	Sox9	140586
3.7633796	down	NM_181374	Sp7	300260
3.7098596	down	XM_001061854	Sp9	366078
14.912797	down	NM_145087	Spag11b	246305
8.52484	down	NM_031792	Spag4	83623
4.984041	down	XM_001063703	Spag4l	296289
2.098576	down	XM_001055656	Spanxn4	302832
2.7964966	down	NM_134384	Spata19	171403
11.409575	down	NM_001004447	Spata21	366491
13.167501	down	XM_001069981	Spdef	689210
2.8541186	down	XM_345315	Spen	365981
22.014265	down	NM_001009976	Spetex-2A	364261
20.690113	down	NM_001011697	Spetex-2B	497084
32.986237	down	NM_001011701	Spetex-2D	364260
33.00796	down	NM_001011702	Spetex-2E	361012
7.4049683	down	NM_001009968	Spetex-2F	316940
24.5716	down	NM_001009971	Spetex-2G	361099
14.015352	down	NM_001009969	Spetex-2H	361008
2.4592133	down	XM_001066641	Spin4	299038
8.391985	down	XM_001055082	Spock2	361840
4.295527	down	NM_172067	Spon1	64456
2.3562298	down	XM_001065906	Spp2	94168
6.4076667	down	XM_001056859	Sprr1al	499660
4.419601	down	XM_001053127	Sprr3	310575
11.875715	down	NM_001009660	Spsb2	297592
3.8297334	down	NM_212522	Sptb	314251
6.3092723	down	XM_001081310	Sptlc3	296188
9.25785	down	XM_001080836	Spty2d1	500144
3.2183204	down	XM_001080260	Srcap	361652
4.172045	down	XM_001077242	Srcrb4d	304401
27.39704	down	XM_001076245	Srgap3	500287
10.478328	down	XM_001074215	Srp72	498351
7.300302	down	XM_573405	Srrm4	498189
2.888992	down	NM_001014236	Ssbp4	364534
5.5894194	down	NM_019348	Sstr2	54305
3.218166	down	NM_053635	St14	114093
4.2346144	down	XM_001060438	Stard4	291699
2.1430657	down	XM_001067555	Steap1	297738
4.135857	down	XM_001070878	Stfa2	303909
2.0769675	down	XM_577078	Stk10	29398
8.883028	down	XM_001058046	Stk36	301516
5.78747	down	NM_019362	Stk39	54348

3.2141066	down	NM_024346	Stmn3	29246
4.721293	down	NM_031126	Stxbp2	81804
23.95302	down	XM_001081248	Stxbp4	303443
2.525851	down	XM_001078123	Stxbp6	362734
3.3270826	down	NM_001009618	Sub1	192269
3.0107605	down	NM_134378	Sulf1	171396
52.0222	down	NM_133547	Sult1c2	171072
40.261364	down	NM_001013177	Sult1c2a	316153
22.78751	down	NM_031732	Sult1c3	65185
17.326527	down	NM_001039665	Sult2b1	292915
14.128263	down	XM_001076682	Susd5	301039
6.839019	down	XM_001058818	Suv420h2	308345
24.005527	down	NM_001007605	Svs3	192239
21.0679	down	XM_001070983	Svs3b	296354
6.364759	down	XM_001071089	Svs6	362267
4.82884	down	NM_001025058	Syce1	502372
9.838761	down	XM_001071232	Syce2	364976
5.16124	down	NM_013041	Sycp3	25561
2.2157347	down	NM_012758	Syk	25155
6.1263666	down	NM_021695	Synpo	60324
7.5448565	down	NM_134328	Taar1	113914
25.675163	down	NM_175590	Taar7e	294133
42.109985	down	NM_175587	Taar7h	294130
3.038993	down	NM_175600	Taar8c	319105
3.6318052	down	NM_012667	Tacr1	24807
7.9131494	down	NM_017053	Tacr3	24808
2.8358464	down	XM_237792	Taf15	287571
10.615102	down	NM_001014155	Taf1c	361420
4.227436	down	XM_001058968	Taf4b	680806
2.1487796	down	XM_001053692	Taf5l	307927
3.1761997	down	XM_001065779	Taf7	307485
6.1170917	down	XM_001067756	Tagap	308097
2.2598703	down	NM_001002854	Tanc1	311055
3.0550249	down	NM_001080938	Tas2r124	690472
3.5335898	down	NM_001025419	Tax1bp3	360564
12.813313	down	XM_221188	Tbc1d16	303734
28.580124	down	XM_001081058	Tbc1d30	299824
2.2528095	down	XM_001057308	Tbc1d7	361227
8.8596	down	NM_172021	Tbkbp1	266764
14.02303	down	XM_001081363	Tbx21	303496
3.878214	down	NM_012687	Tbxas1	24886
5.090951	down	XM_001069071	Tcea3	298559
2.9629235	down	NM_022593	Tceb1	64525
3.4466717	down	NM_031129	Tceb2	81807

10.584422	down	NM_213561	Tcf19	406195
7.0150175	down	XM_001067558	Tcf23	688600
7.045187	down	XM_001073458	Tcf7	363595
16.169626	down	XM_001062658	Tcfap2a	306862
12.17776	down	XM_233526	Tcfap2e	313596
8.993745	down	XM_001064620	Tcp10b	308169
5.1214204	down	XM_001063024	Tctex1d1	362553
2.6400044	down	XM_001072977	Tctex1d2	498095
11.221302	down	XM_001073003	Tdpoz1	365854
5.4460526	down	XM_001063929	Tdrd1	292129
3.562421	down	XM_001069186	Tead1	361630
3.22328	down	XM_001080702	Tead2	308582
6.6962366	down	NM_001037191	Tecpr1	304285
2.5850232	down	NM_001024739	Tekt3	287392
4.479706	down	XM_001076953	Tesc	288689
3.9748976	down	NM_001013226	Tesp1	363215
23.580832	down	XM_001055364	Tesp2	316318
3.2970421	down	NM_001008864	Tessp5	408244
2.085742	down	XM_576992	Tex11	501588
3.9375358	down	NM_057129	Tff1	117270
9.832263	down	NM_053844	Tff2	116592
14.541262	down	XM_001060570	Tgfbrap1	301373
2.1227784	down	NM_019386	Tgm2	56083
5.475905	down	XM_001079039	Tgm6	296152
5.0678086	down	NM_138840	Tgoln1	192152
7.4444385	down	NM_001008340	Thap1	306547
2.74669	down	NM_001013966	Thg1l	303067
3.1480958	down	XM_001064856	Thsd1	364630
11.364161	down	XM_222597	Thsd7b	289007
8.264286	down	NM_001012108	Thumpd2	313851
2.2411628	down	NM_001007661	Thyn1	300470
2.474867	down	XM_001054397	Ticam2	364867
15.252517	down	NM_001025029	Tifab	364674
10.430116	down	XM_001067871	Tigit	363784
7.125589	down	NM_001013855	Timd2	287222
2.0869474	down	XM_001065015	Timm50	685725
18.832434	down	XM_001058904	Timm8a2	680794
2.252321	down	XM_001073142	Tktl2	290685
5.7779307	down	XM_001080690	Tlcd2	497955
10.95	down	NM_001039013	Tle2	299636
3.0694475	down	XM_001060099	Tlr12	362604
34.430576	down	XM_001054552	Tlx2	680117
2.2486167	down	XM_001079837	Tm4sf5	303256
3.5544846	down	NM_001011970	Tm7sf3	297725

2.33084	down	NM_023020	Tmeff1	63845
3.4728181	down	XM_217247	Tmem108	300967
26.079529	down	XM_001057254	Tmem127	311405
37.02928	down	XM_001071422	Tmem130	304280
2.86347	down	XM_001081021	Tmem132e	287564
2.3456194	down	XM_001079995	Tmem143	308593
4.4577413	down	XM_001079138	Tmem149	499126
47.266262	down	XM_001067823	Tmem150c	360916
3.554387	down	NM_057212	Tmem158	117582
2.8086674	down	XM_001061410	Tmem170b	361230
6.2555194	down	XM_001076493	Tmem184b	362959
2.2480125	down	XM_001059247	Tmem191c	680867
4.676673	down	XM_001080277	Tmem20	294072
6.4052186	down	XM_001071866	Tmem213	689748
2.795846	down	XM_001074731	Tmem216	361727
3.2390783	down	NM_001014192	Tmem39b	362608
5.5261703	down	XM_001064861	Tmem63b	363197
2.8862386	down	XM_001068973	Tmem68	312946
12.517086	down	XM_001072960	Tmem82	298605
5.8393135	down	XM_001075148	Tmem92	690666
6.954381	down	NM_022630	Tmprss11d	64565
3.9975812	down	XM_001074782	Tmprss11f	498345
3.627165	down	XM_345932	Tmprss4	367074
2.752349	down	XM_001065494	Tnfaip6	84397
2.325662	down	XM_001056720	Tnfaip8	307428
14.340917	down	XM_236246	Tnfaip8l3	315673
2.3546276	down	XM_001069338	Tnfrsf10b	364420
5.654168	down	XM_001076437	Tnfrsf25	500592
2.7568755	down	NM_013049	Tnfrsf4	25572
18.640078	down	XM_001079627	Tnk1	303247
3.3417504	down	NM_001008336	Tnk2	303882
10.446853	down	XM_001073586	Tnn	304913
4.4298162	down	NM_017144	Tnni3	29248
99.705536	down	NM_017057	Tnp2	24840
5.2462616	down	NM_001024881	Tns4	303517
2.2251227	down	XM_001077625	Top3a	303194
12.50912	down	XM_001063870	Tp53inp2	362246
2.594347	down	NM_019221	Tp63	246334
9.717764	down	XM_001077013	Tp73	362675
3.5161586	down	NM_001079531	Tpmt	690050
3.3659635	down	NM_053534	Tpra1	85494
2.1753612	down	XM_001060281	Tpx2	311546
3.2151585	down	NM_001044248	Traf3ip2	361857
18.309402	down	XM_001064383	Tram1	312903

14.83324	down	XM_001058030	Trap1a	680619
2.0409422	down	XM_001063859	Trem1	301229
5.6933727	down	XM_001063589	Trem2	301227
61.934856	down	XM_576511	Treml1	501096
10.066272	down	XM_001059217	Treml4	680862
6.4654016	down	NM_013046	Trh	25569
3.3014073	down	NM_001082572	Trim21	308901
3.9790828	down	NM_001011665	Trim26	309586
6.3013506	down	XM_574516	Trim30	499223
2.1345108	down	XM_001053970	Trim36	291597
2.1898646	down	NM_001013203	Trim44	362172
2.7656956	down	XM_001077454	Trim52	290458
17.178259	down	XM_001075898	Trim58	303167
2.042422	down	XM_001060201	Trim62	313045
5.897168	down	XM_001065684	Trip11	314393
2.0856304	down	NM_198051	Trmt11	378794
2.818917	down	NM_001011895	Trmt2a	287953
13.34085	down	NM_001007706	Trmt61a	314462
5.2546	down	NM_001083115	Trpc4	84494
5.0272355	down	NM_080898	Trpc5	140933
5.6737666	down	NM_001037733	Trpm1	361586
2.6409469	down	NM_017207	Trpv2	29465
9.543892	down	XM_001052990	Trub2	366012
22.362726	down	XM_001054665	Tsfm	679068
9.114251	down	NM_013116	Tshb	25653
2.6804051	down	XM_001073538	Tsnaxip1	498944
10.098176	down	NM_001024262	Tspan11	312727
4.8950973	down	XM_001065183	Tspan32	685765
7.771517	down	NM_001012075	Tspyl4	309828
3.3878427	down	NM_001007650	Tssk3	297891
14.274149	down	XM_001066190	Ttbk1	316229
2.1958535	down	XM_001080916	Ttbk2	311349
2.3688211	down	XM_001078737	Ttc17	311224
3.0527537	down	NM_001013890	Ttc29	291944
7.4723063	down	XM_343094	Ttc7b	362768
31.891794	down	XM_576070	Ttc9	500689
5.825646	down	NM_001024758	Ttll10	298692
2.2060044	down	XM_001070417	Ttyh1	292597
8.592023	down	XM_221081	Ttyh2	287803
22.318333	down	XM_001073693	Ttyh3	304315
4.5726905	down	NM_139254	Tubb3	246118
2.4709783	down	XM_345244	Tuft1	365864
2.9435372	down	NM_053530	Twist1	85489
3.4286902	down	XM_001061592	Txlna	682457

2.0407162	down	XM_001067457	Txndc12	298370
4.873921	down	NM_001005559	Txndc2	316777
3.6692493	down	NM_212525	Tyrobp	361537
2.0179164	down	XM_001077578	Uap1l1	296560
2.8956628	down	XM_001065047	Ubash3b	315579
7.3723345	down	XM_228708	Ube2q2l	317341
6.8744273	down	XM_001074290	Ubiad1	313706
2.3589065	down	NM_001014117	Ublcp1	360514
3.1056738	down	XM_001063674	Ubtd2	287178
3.6114106	down	XM_001073045	Ubxn7	303878
20.031277	down	NM_019150	Ucn	29151
2.187101	down	NM_012683	Ugt1a1	24861
2.044559	down	XM_001057451	Ugt3a2	294793
2.049816	down	NM_019276	Ugt8	50555
2.0968475	down	XM_220541	Ulk2	303206
2.4731581	down	XM_001079051	Umodl1	365544
5.100558	down	XM_001077605	Unc119b	288702
2.365452	down	NM_199407	Unc5c	362049
3.8925326	down	NM_053845	Upb1	116593
23.265436	down	XM_001066120	Uroc1	685999
2.0424626	down	XM_001078228	Ush1c	308596
4.702354	down	NM_001033687	Ushbp1	290629
3.019208	down	XM_001074575	Usp4	290864
15.562094	down	NM_019160	Uts2	29180
79.87199	down	NM_001008898	V1ra14	297442
23.42862	down	NM_001008953	V1re18	494299
19.019907	down	XM_001056450	Vangl2	289229
6.9668207	down	XM_001055204	Vasn	679921
3.5489345	down	NM_001033683	Vat1	287721
5.9457936	down	XM_001057825	Vil1	316521
2.6421413	down	XM_001077346	Vill	301057
3.213076	down	NM_012685	Vipr1	24875
7.2471523	down	NM_173112	Vom1r101	286892
21.687485	down	NM_173113	Vom1r105	286893
2.2455554	down	NM_001008962	Vom1r12	494306
21.755983	down	NM_001008952	Vom1r27	494297
19.054249	down	NM_001008946	Vom1r29	494292
7.9714518	down	NM_001008939	Vom1r46	494286
13.574978	down	NM_001008930	Vom1r52	494278
2.0121384	down	NM_001008963	Vom1r54	365190
2.2132733	down	NM_001009530	Vom1r85	494250
8.630073	down	NM_153729	Vom1r90	266771
3.533272	down	NM_173297	Vom1r97	286956
55.709167	down	XM_001053213	Vom2r1	678740

7.2030125	down	XM_001074899	Vom2r13	690593
24.75204	down	XM_218099	Vom2r2	292438
21.361368	down	XM_577675	Vom2r3	502213
3.003517	down	NM_173319	Vom2r31	286985
10.111616	down	NM_173315	Vom2r32	286981
29.530499	down	XM_218106	Vom2r4	308248
10.795661	down	XM_001081473	Vom2r40	286983
2.2672434	down	XM_001053623	Vom2r43	679593
27.80621	down	XM_001053116	Vom2r5	679691
13.583003	down	XM_001075977	Vom2r63	288541
11.443346	down	XM_001060234	Vom2r64	681079
55.99071	down	XM_001070665	Vom2r65	689403
6.4504647	down	XM_001071469	Vom2r-ps124	689642
3.0744836	down	XM_001071402	Vom2r-ps39	502287
4.6234875	down	XM_001066381	Vom2r-ps89	304229
24.531763	down	XM_001062818	Vpreb2	363830
2.340953	down	XM_001077797	Vps13a	309243
5.2692103	down	XM_001064954	Vps36	290851
3.0801206	down	XM_001074854	Vps37b	288659
2.831319	down	XM_213395	Vps53	287535
2.3685493	down	NM_001037784	Vsig1	315920
23.668324	down	XM_001058168	Vsig2	300520
4.2755284	down	XM_001069552	Vstm2a	689106
6.056609	down	XM_001058827	Vsx2	171360
4.40635	down	XM_578230	Vwde	502731
4.147468	down	XM_001064536	Wdfy2	305956
2.99389	down	XM_001062583	Wdhd1	305827
2.6690361	down	NM_199410	Wdr12	363237
3.072194	down	XM_001057879	Wdr13	317370
3.7655234	down	XM_001071278	Wdr20a	314453
4.4579506	down	XM_001055617	Wdr27	308222
10.230326	down	XM_578535	Wdr35l	503018
3.2214599	down	XM_344019	Wdr52	363782
2.0325444	down	XM_001073107	Wdr53	498097
2.6549633	down	XM_218509	Wdr88	292809
2.122769	down	XM_001062276	Wdr90	287157
3.476677	down	XM_001064989	Wdtc1	313020
2.304006	down	NM_001012742	Wee1	308937
12.225084	down	NM_001008866	Wfdc12	408227
3.4563618	down	NM_001009179	Wfdc15b	408230
2.7327864	down	XM_001062781	Wfdc16	685201
3.2262995	down	XM_001062561	Wfdc6a	685153
24.087214	down	XM_001068398	Wibg	366790
5.159103	down	XM_340895	Wipf2	360620

6.2933874	down	NM_147211	Wipf3	259242
2.0795703	down	NM_031590	Wisp2	29576
2.2059984	down	XM_001074919	Wiz	314598
5.1831026	down	XM_001062326	Wnt10b	315294
4.4127584	down	XM_001059030	Wnt2	114487
4.8066373	down	XM_001081545	Wnt3	24882
5.4180894	down	XM_001076985	Wnt3a	303181
34.15898	down	XM_237295	Wnt6	316526
8.157717	down	XM_001061171	Wnt8a	291678
6.5309486	down	XM_001077006	Wnt9a	287357
11.90724	down	NM_031534	Wt1	24883
3.1337159	down	XM_001079532	Wtip	361552
18.38775	down	NM_001012042	Xkr6	305960
3.5652435	down	NM_001012099	Xkr8	313033
2.2347631	down	NM_001012230	Xkrx	497101
77.3982	down	XM_001073004	Xlr3a	690041
9.34971	down	XM_001054068	Xlr4a	293841
2.3579578	down	NM_001013863	Ydjc	287938
4.2478347	down	NM_033298	Yes1	24884
4.1754026	down	NM_001025729	Zbed3	361881
2.4493458	down	NM_199496	Zbtb25	314245
2.2144227	down	XM_001068541	Zbtb32	688845
5.843092	down	XM_001062353	Zbtb45	308366
8.414466	down	XM_001075474	Zbtb48	362668
2.6770947	down	XM_001060900	Zbtb8b	500553
2.0496092	down	XM_224295	Zc3h13	305955
2.9806151	down	XM_346320	Zc4h2	367838
2.4950998	down	XM_001073035	Zcchc10	360524
3.3127115	down	NM_001014065	Zcchc12	313436
2.8472152	down	XM_001079169	Zcchc14	365018
4.518474	down	XM_001076305	Zcwpw1	304368
6.8973913	down	NM_001039101	Zdhhc15	317235
35.44543	down	NM_001039009	Zdhhc21	298184
5.6518793	down	NM_001039100	Zdhhc24	293665
3.7060175	down	NM_001008363	Zfand2a	360772
59.75459	down	XM_001058915	Zfhx4	310250
2.4678466	down	XM_001072483	Zfp12	288486
15.272711	down	XM_001078438	Zfp167	363170
5.730972	down	XM_225349	Zfp192	306974
5.909386	down	XM_001059933	Zfp275	293849
2.622548	down	NM_001012058	Zfp276	307924
18.9585	down	XM_001064187	Zfp278	305471
10.034515	down	XM_001066602	Zfp29	686118
9.314803	down	XM_001055374	Zfp324	365192

2.8585935	down	NM_058209	Zfp37	115768
6.2916985	down	XM_001062461	Zfp385b	311137
3.1799476	down	XM_001061052	Zfp407	307213
6.6061435	down	NM_001079943	Zfp426l	367034
2.2661996	down	XM_001074185	Zfp653	300446
4.9964213	down	NM_001009656	Zgpat	296478
2.9106355	down	NM_133620	Zhx1	171159
6.4303336	down	XM_341381	Zic2	361096
3.266217	down	XM_001068350	Zim1	308322
4.080939	down	NM_001025760	Zkscan1	498160
2.4611647	down	XM_001061563	Zmym3	317260
2.6119797	down	XM_001055163	Znf213	287094
7.1302958	down	XM_001053378	Znf282	297065
2.3991108	down	NM_145724	Znf394	252860
4.893431	down	XM_001080639	Znf473	292884
8.742044	down	XM_001057553	Znf474	307310
6.061874	down	XM_001061809	Znf498	363872
3.7457666	down	XM_001079835	Znf507	292816
29.118053	down	XM_001080163	Znf553	308991
4.234754	down	XM_001076652	Znf575	308430
3.0262823	down	XM_001076726	Znf609	363412
3.5136085	down	XM_001064168	Znf618	313253
11.158872	down	XM_001080269	Znf629	308998
2.3363054	down	XM_001061309	Znf641	300197
4.0699096	down	NM_001080207	Znf652	497984
4.405996	down	NM_173330	Znf689	286996
2.2196004	down	XM_001058556	Znf703	680717
2.3207905	down	XM_001068197	Znf76	361809
2.0452445	down	XM_001078321	Znf763	314586
17.080917	down	XM_001079970	Znf764	691885
16.940266	down	XM_001067642	Znf773	502292
4.5289416	down	XM_001068316	Znf804a	295695
2.5268648	down	XM_226449	Znf821	307834
4.9695725	down	XM_001076915	Znhit1	360784
2.19726	down	XM_001059988	Zscan20	500549
2.1971748	down	NM_001008308	Zufsp	294390

MiR-434-3p overexpression _vs_Control 2.0 fold up regulated genes

Fold change	Regulation	ACCESSION	GeneSymbol	GeneID
2.3291411	up	NM_130399	Ada	24165
2.0532598	up	XM_001062804	Aldh5a1	291133
2.7336085	up	NM_012777	Apod	25239
2.3426118	up	XM_001061611	C1r	312705
2.031044	up	NM_016994	C3	24232
3.5443885	up	NM_001002805	C4-2	406161
3.224404	up	NM_031504	C4b	24233
2.0677345	up	NM_138915	Caly	192349
2.4624414	up	XM_001066248	Ccdc150	316399
2.0082316	up	NM_130812	Cdkn2b	25164
2.281728	up	XM_001074104	Cish	83681
2.1080773	up	XM_001074417	Cox19	304330
6.3228664	up	NM_080399	Ddit4l	140582
2.1313102	up	XM_001081786	Eif4a3	688288
2.0268247	up	XM_001071395	Exosc3	313243
2.2058744	up	NM_001014050	Fam110a	311535
2.2469897	up	XM_001062665	Fam163b	685169
2.5436077	up	XM_001061851	Fam186a	684999
2.2158797	up	NM_022182	Fgf7	29348
2.014882	up	XM_001064153	Frg1l	361156
4.285344	up	NM_001077640	Gadd45g	291005
2.0066333	up	NM_012736	Gpd2	25062
2.0962913	up	NM_181770	Gpr119	302813
2.0294363	up	NM_031623	Grb14	58844
2.1656728	up	NM_017122	Hpca	29177
2.2540815	up	XM_001053962	Hrasls	288025
2.1894298	up	NM_001082477	Igf1	24482
2.0168078	up	NM_001013048	Igfbp7	289560
2.2563148	up	XM_001074037	Imp3	315697
2.3453574	up	XM_001069483	Kdm1	500569
2.148561	up	XM_001065538	Klhl8	289457
4.997229	up	XM_001063981	Krtap21-2	685485
3.3775198	up	XM_577124	Krtap2-4	501720
2.5803301	up	XM_001055597	Ldoc1	367956
2.3878047	up	XM_001064280	LOC287921	287921
2.9513376	up	XM_001066324	LOC288419	288419
2.1946557	up	XM_223807	LOC289918	289918
2.2405474	up	XM_001071880	LOC293697	293697
4.0627837	up	XM_228268	LOC294497	294497
5.454705	up	XM_001065571	LOC298820	298820
2.2155619	up	XM_236873	LOC301193	301193
2.9118931	up	XM_001074409	LOC301748	301748

3.0154173	up	XM_229238	LOC301772	301772
2.292094	up	XM_229292	LOC301816	301816
3.9416857	up	XM_001061753	LOC301839	301839
2.5699413	up	XM_217510	LOC301977	301977
3.6752853	up	XM_229524	LOC302000	302000
3.0733488	up	XM_217530	LOC302085	302085
3.0800085	up	XM_001065392	LOC302192	302192
2.589499	up	XM_217542	LOC302199	302199
2.1098735	up	XM_223806	LOC305698	305698
2.2127109	up	XM_001081925	LOC305771	305771
3.5160017	up	XM_001065941	LOC306312	306312
8.524204	up	XM_224750	LOC306365	306365
2.6962013	up	XM_001081637	LOC307706	307706
4.970486	up	XM_217963	LOC308198	308198
3.7739859	up	XM_236887	LOC316192	316192
2.0648422	up	XM_001078812	LOC316493	316493
2.416704	up	XM_229366	LOC316856	316856
2.5627372	up	XM_001064910	LOC316873	316873
2.7373226	up	XM_229488	LOC316919	316919
3.304061	up	XM_229505	LOC316931	316931
2.9259698	up	XM_229508	LOC316933	316933
2.3500063	up	XM_001078949	LOC317085	317085
3.717472	up	XM_001057404	LOC360998	360998
3.668232	up	XM_001061883	LOC363181	363181
2.3723235	up	XM_347120	LOC363301	363301
4.092005	up	XM_001062328	LOC363306	363306
2.2208025	up	XM_343660	LOC363320	363320
3.3853881	up	XM_343663	LOC363324	363324
2.3424554	up	XM_343674	LOC363336	363336
2.5568564	up	NM_001014221	LOC363337	363337
3.63308	up	XM_001081023	LOC363343	363343
3.0022	up	XM_001076189	LOC363354	363354
2.3041904	up	XM_001077951	LOC363363	363363
3.0145245	up	XM_001074547	LOC363380	363380
3.8859959	up	XM_343727	LOC363397	363397
4.428336	up	XM_343755	LOC363433	363433
3.3838935	up	XM_343756	LOC363434	363434
3.4362752	up	XM_344247	LOC364164	364164
2.425887	up	XM_344294	LOC364250	364250
3.3842156	up	XM_001053352	LOC365499	365499
3.9976592	up	XM_001055159	LOC365501	365501
2.6256914	up	XM_345058	LOC365502	365502
3.2740605	up	XM_345061	LOC365506	365506
5.8581786	up	XM_001070902	LOC366780	366780

2.344636	up	XM_001078369	LOC367377	367377
4.3453546	up	XM_001067423	LOC367381	367381
4.2712193	up	XM_346138	LOC367436	367436
3.6702027	up	XM_001059570	LOC367485	367485
2.265313	up	XM_001075365	LOC367493	367493
2.0016234	up	XM_001062392	LOC367516	367516
2.1113546	up	XM_346193	LOC367523	367523
2.9983046	up	XM_001063146	LOC367539	367539
2.742218	up	XM_001081934	LOC367617	367617
2.7056704	up	XM_346234	LOC367632	367632
3.092772	up	XM_346241	LOC367653	367653
3.7604427	up	XM_573546	LOC498320	498320
2.0539243	up	XM_001061548	LOC498372	498372
4.212043	up	XM_573609	LOC498374	498374
2.037956	up	XM_573699	LOC498444	498444
3.5069547	up	XM_001079520	LOC498446	498446
3.5352485	up	XM_001076556	LOC498463	498463
2.1223006	up	XM_001072047	LOC498465	498465
2.4272547	up	XM_573724	LOC498467	498467
2.2594962	up	XM_573725	LOC498468	498468
2.4872637	up	XM_573738	LOC498480	498480
2.3099864	up	XM_573955	LOC498672	498672
5.9924226	up	XM_001063340	LOC498781	498781
4.522934	up	XM_579956	LOC499384	499384
2.6361122	up	XM_001079909	LOC499779	499779
2.885358	up	XM_001077472	LOC500584	500584
2.283609	up	XM_576202	LOC500815	500815
2.163841	up	XM_576504	LOC501089	501089
2.9291902	up	XM_576506	LOC501091	501091
3.1863053	up	XM_576507	LOC501092	501092
2.1161885	up	XM_580174	LOC501212	501212
2.6657073	up	XM_001067568	LOC501220	501220
3.758037	up	XM_576645	LOC501222	501222
2.9512527	up	XM_001071000	LOC501223	501223
4.0529714	up	XM_576649	LOC501226	501226
4.9073195	up	XM_001076269	LOC501230	501230
2.9731553	up	XM_576656	LOC501235	501235
4.0691037	up	XM_576669	LOC501250	501250
3.3649178	up	XM_576670	LOC501251	501251
2.0399616	up	XM_576671	LOC501252	501252
3.4047549	up	XM_576674	LOC501255	501255
2.7326708	up	XM_576675	LOC501256	501256
5.3115973	up	XM_576687	LOC501271	501271
3.6445477	up	XM_576689	LOC501274	501274

3.3997746	up	XM_576705	LOC501292	501292
3.4637735	up	XM_576712	LOC501299	501299
2.8907304	up	XM_576715	LOC501302	501302
2.3262646	up	XM_576718	LOC501305	501305
3.2684124	up	XM_576719	LOC501306	501306
2.5523398	up	XM_576720	LOC501307	501307
2.3789747	up	XM_001068431	LOC501321	501321
2.3029492	up	XM_001059339	LOC501329	501329
2.567176	up	XM_576744	LOC501333	501333
3.0535045	up	XM_576745	LOC501334	501334
2.406013	up	XM_001081326	LOC501342	501342
4.072971	up	XM_576757	LOC501346	501346
3.7041378	up	XM_576760	LOC501349	501349
2.5708494	up	XM_001077232	LOC501350	501350
2.3429332	up	XM_576770	LOC501358	501358
3.0204566	up	XM_001074895	LOC501362	501362
3.197504	up	XM_576779	LOC501366	501366
3.2608733	up	XM_576781	LOC501368	501368
3.5371256	up	XM_576782	LOC501369	501369
3.1269917	up	XM_001069914	LOC501373	501373
2.3987532	up	XM_576790	LOC501377	501377
2.2681165	up	XM_001081029	LOC501383	501383
2.7903717	up	XM_576799	LOC501386	501386
2.3583522	up	XM_576801	LOC501388	501388
2.75793	up	XM_576802	LOC501389	501389
2.290257	up	XM_580189	LOC501391	501391
4.4871964	up	XM_576812	LOC501399	501399
3.3765566	up	XM_576813	LOC501400	501400
2.1818562	up	XM_576828	LOC501417	501417
3.2680905	up	XM_001058335	LOC501421	501421
2.4480462	up	XM_001078724	LOC501427	501427
3.715179	up	XM_576877	LOC501467	501467
2.690287	up	XM_576879	LOC501469	501469
2.5234537	up	XM_001066518	LOC501470	501470
2.238637	up	XM_576882	LOC501474	501474
2.1973398	up	XM_001080724	LOC501476	501476
2.7639308	up	XM_576885	LOC501477	501477
4.469603	up	XM_001081012	LOC501479	501479
2.2795527	up	XM_001080503	LOC501482	501482
2.4388347	up	XM_576890	LOC501483	501483
4.5627065	up	XM_580199	LOC501485	501485
2.138379	up	XM_576894	LOC501488	501488
2.1375775	up	XM_576897	LOC501491	501491
3.6205425	up	XM_001066422	LOC501791	501791

2.4811885	up	XM_001069885	LOC501799	501799
3.9606607	up	XM_001070011	LOC501800	501800
3.3163695	up	XM_001081795	LOC501937	501937
6.074038	up	XM_001071460	LOC502276	502276
5.025355	up	XM_001071332	LOC502279	502279
3.1145644	up	XM_577967	LOC502486	502486
4.0135317	up	XM_578861	LOC503327	503327
5.105775	up	XM_001053092	LOC678713	678713
3.780789	up	XM_001053285	LOC678755	678755
3.0097826	up	XM_001053936	LOC678951	678951
2.9500892	up	XM_001054541	LOC679066	679066
2.733043	up	XM_001054917	LOC679152	679152
2.2564504	up	XM_001055060	LOC679174	679174
2.1192772	up	XM_001055619	LOC679284	679284
3.9960556	up	XM_001056500	LOC679467	679467
3.5477686	up	XM_001056539	LOC679474	679474
3.382224	up	XM_001053499	LOC679562	679562
2.3136287	up	XM_001053708	LOC679608	679608
5.125708	up	XM_001053745	LOC679613	679613
2.1267974	up	XM_001053751	LOC679615	679615
3.0059063	up	XM_001053909	LOC679662	679662
6.900456	up	XM_001054076	LOC679696	679696
4.2452507	up	XM_001054104	LOC679703	679703
3.2003598	up	XM_001054169	LOC679718	679718
3.7635226	up	XM_001054170	LOC679719	679719
4.036247	up	XM_001054202	LOC679724	679724
2.2849212	up	XM_001054232	LOC679730	679730
2.0428164	up	XM_001054303	LOC679745	679745
2.284564	up	XM_001054394	LOC679766	679766
2.5535085	up	XM_001054477	LOC679792	679792
2.0516806	up	XM_001054511	LOC679798	679798
3.681184	up	XM_001054539	LOC679807	679807
4.048343	up	XM_001054768	LOC679861	679861
4.9608517	up	XM_001054782	LOC679867	679867
2.0527868	up	XM_001054812	LOC679879	679879
2.1938846	up	XM_001054886	LOC679901	679901
2.7065601	up	XM_001054925	LOC679909	679909
4.729544	up	XM_001054988	LOC679927	679927
2.1972082	up	XM_001054993	LOC679930	679930
2.197498	up	XM_001055170	LOC679982	679982
6.4203715	up	XM_001055300	LOC680010	680010
2.781177	up	XM_001055404	LOC680029	680029
2.5484734	up	XM_001055521	LOC680062	680062
3.188386	up	XM_001055573	LOC680075	680075

3.363838	up	XM_001055748	LOC680106	680106
2.1907623	up	XM_001055766	LOC680112	680112
3.0750334	up	XM_001055864	LOC680136	680136
2.9590342	up	XM_001055983	LOC680166	680166
3.5477006	up	XM_001056044	LOC680185	680185
3.5951362	up	XM_001056442	LOC680281	680281
3.8544183	up	XM_001056624	LOC680318	680318
4.3920093	up	XM_001056791	LOC680347	680347
3.5489097	up	XM_001056978	LOC680387	680387
3.096708	up	XM_001057146	LOC680429	680429
5.219148	up	XM_001057154	LOC680431	680431
3.0004847	up	XM_001057208	LOC680443	680443
2.6329355	up	XM_001053549	LOC680453	680453
2.0044558	up	XM_001057350	LOC680475	680475
2.106625	up	XM_001057558	LOC680525	680525
2.303001	up	XM_001057668	LOC680547	680547
5.078352	up	XM_001057966	LOC680608	680608
5.158419	up	XM_001058086	LOC680636	680636
3.6746926	up	XM_001058089	LOC680637	680637
2.3301291	up	XM_001058184	LOC680653	680653
4.067345	up	XM_001058208	LOC680658	680658
2.6368074	up	XM_001058554	LOC680716	680716
3.6694374	up	XM_001058696	LOC680748	680748
7.110875	up	XM_001058749	LOC680759	680759
3.6066546	up	XM_001059100	LOC680831	680831
2.837026	up	XM_001059281	LOC680879	680879
2.404861	up	XM_001059970	LOC681020	681020
2.4544067	up	XM_001059994	LOC681027	681027
2.0882094	up	XM_001060161	LOC681067	681067
2.0192235	up	XM_001060188	LOC681071	681071
2.555301	up	XM_001060331	LOC681105	681105
3.056849	up	XM_001060374	LOC681115	681115
2.1558707	up	XM_001060390	LOC681122	681122
2.292319	up	XM_001060454	LOC681139	681139
6.7606597	up	XM_001060513	LOC681151	681151
3.4643826	up	XM_001060628	LOC681176	681176
3.019687	up	XM_001059937	LOC681180	681180
2.2534416	up	XM_001060675	LOC681187	681187
3.4798417	up	XM_001060741	LOC681204	681204
2.4023561	up	XM_001060797	LOC681220	681220
3.030925	up	XM_001060847	LOC681230	681230
2.8791451	up	XM_001060969	LOC681259	681259
2.2754366	up	XM_001061073	LOC681283	681283
2.7959552	up	XM_001061123	LOC681296	681296

2.1898184	up	XM_001061128	LOC681299	681299
2.4153967	up	XM_001061298	LOC681333	681333
2.4282184	up	XM_001061372	LOC681353	681353
2.3465579	up	XM_001061602	LOC681405	681405
3.1889477	up	XM_001057300	LOC681537	681537
2.6562676	up	XM_001057478	LOC681575	681575
3.3137681	up	XM_001057766	LOC681652	681652
2.7165701	up	XM_001057939	LOC681685	681685
2.319809	up	XM_001058241	LOC681743	681743
4.4879203	up	XM_001059139	LOC681968	681968
3.7391763	up	XM_001060373	LOC682199	682199
7.085001	up	XM_001060443	LOC682210	682210
2.5641801	up	XM_001060854	LOC682289	682289
2.302395	up	XM_001061066	LOC682335	682335
5.608393	up	XM_001061832	LOC682515	682515
2.5731301	up	XM_001062253	LOC682603	682603
4.378068	up	XM_001063145	LOC682796	682796
2.8800044	up	XM_001063233	LOC682815	682815
2.1568606	up	XM_001063412	LOC682856	682856
4.4688497	up	XM_001063439	LOC682858	682858
2.3172922	up	XM_001063448	LOC682859	682859
2.1036186	up	XM_001063527	LOC682880	682880
2.6834614	up	XM_001063558	LOC682885	682885
2.7515156	up	XM_001064138	LOC683020	683020
2.999833	up	XM_001064734	LOC683166	683166
2.1664553	up	XM_001065323	LOC683308	683308
3.388759	up	XM_001066056	LOC683469	683469
2.4293935	up	XM_001067117	LOC683698	683698
2.0699224	up	XM_001067816	LOC683865	683865
2.9391859	up	XM_001067901	LOC683878	683878
2.7309904	up	XM_001068139	LOC683944	683944
3.3860204	up	XM_001068277	LOC683973	683973
2.2300637	up	XM_001068831	LOC684077	684077
2.9982889	up	XM_001069050	LOC684128	684128
3.0683672	up	XM_001069080	LOC684134	684134
4.5896792	up	XM_001069913	LOC684337	684337
2.729958	up	XM_001071070	LOC684575	684575
3.3407145	up	XM_001071142	LOC684587	684587
2.723823	up	XM_001055278	LOC684652	684652
3.7084215	up	XM_001071746	LOC684725	684725
3.3427954	up	XM_001071914	LOC684779	684779
2.1340368	up	XM_001071939	LOC684787	684787
2.1939297	up	XM_001072070	LOC684824	684824
2.1462598	up	XM_001072410	LOC684907	684907

4.2841573	up	XM_001072445	LOC684915	684915
2.5040884	up	XM_001061802	LOC684990	684990
3.1784933	up	XM_001061948	LOC685019	685019
2.8992252	up	XM_001057025	LOC685044	685044
2.2749364	up	XM_001062073	LOC685053	685053
3.222528	up	XM_001062150	LOC685071	685071
2.4854565	up	XM_001062177	LOC685075	685075
3.0498958	up	XM_001063598	LOC685110	685110
3.0631053	up	XM_001062428	LOC685125	685125
2.9890542	up	XM_001062656	LOC685166	685166
2.2234259	up	XM_001062681	LOC685176	685176
2.5382268	up	XM_001062796	LOC685205	685205
2.4305863	up	XM_001057196	LOC685285	685285
2.157312	up	XM_001057314	LOC685300	685300
4.838328	up	XM_001063228	LOC685303	685303
3.9121387	up	XM_001063242	LOC685306	685306
3.0019865	up	XM_001057537	LOC685344	685344
2.962859	up	XM_001063434	LOC685347	685347
5.1111727	up	XM_001063435	LOC685348	685348
2.0465982	up	XM_001063442	LOC685349	685349
3.5002348	up	XM_001063496	LOC685363	685363
2.0128787	up	XM_001063513	LOC685369	685369
2.442179	up	XM_001063529	LOC685371	685371
2.5696907	up	XM_001063548	LOC685377	685377
3.9270394	up	XM_001063606	LOC685392	685392
2.136064	up	XM_001063747	LOC685424	685424
2.2203019	up	XM_001057638	LOC685435	685435
2.2578292	up	XM_001063788	LOC685437	685437
3.0589108	up	XM_001063877	LOC685459	685459
2.1970782	up	XM_001063912	LOC685469	685469
4.3820677	up	XM_001064038	LOC685496	685496
4.4127774	up	XM_001064282	LOC685552	685552
3.2769318	up	XM_001064363	LOC685573	685573
4.00499	up	XM_001064447	LOC685593	685593
2.4797194	up	XM_001057826	LOC685609	685609
3.91345	up	XM_001064704	LOC685661	685661
2.8989809	up	XM_001064760	LOC685668	685668
2.9885998	up	XM_001064946	LOC685711	685711
4.4952617	up	XM_001064972	LOC685717	685717
2.7023368	up	XM_001065049	LOC685735	685735
3.7006233	up	XM_001065160	LOC685759	685759
2.0263727	up	XM_001065161	LOC685760	685760
4.820369	up	XM_001065189	LOC685769	685769
4.1998515	up	XM_001065214	LOC685773	685773

3.0752347	up	XM_001057991	LOC685779	685779
4.2766576	up	XM_217497	LOC685792	685792
4.426156	up	XM_001065304	LOC685796	685796
2.5597396	up	XM_001065332	LOC685805	685805
3.3229418	up	XM_001065446	LOC685835	685835
3.9396029	up	XM_001065511	LOC685853	685853
3.0753963	up	XM_001065537	LOC685863	685863
2.4187818	up	XM_001065569	LOC685871	685871
2.6854799	up	XM_001065570	LOC685872	685872
2.4857466	up	XM_001065656	LOC685891	685891
2.3342624	up	XM_001065719	LOC685908	685908
3.8729274	up	XM_001065748	LOC685918	685918
2.6050885	up	XM_001065901	LOC685948	685948
6.782266	up	XM_001065963	LOC685965	685965
3.1563735	up	XM_001065967	LOC685966	685966
2.5970976	up	XM_001066016	LOC685974	685974
3.0852597	up	XM_001066071	LOC685986	685986
2.674438	up	XM_001066074	LOC685987	685987
3.623533	up	XM_001066079	LOC685989	685989
3.1311626	up	XM_001066107	LOC685994	685994
3.4545162	up	XM_001066158	LOC686010	686010
3.193804	up	XM_001066192	LOC686017	686017
2.6174963	up	XM_001066193	LOC686018	686018
3.1956804	up	XM_001066217	LOC686023	686023
2.2065754	up	XM_001066250	LOC686031	686031
3.1735628	up	XM_001066301	LOC686041	686041
3.309122	up	XM_001066372	LOC686058	686058
2.910974	up	XM_001066380	LOC686061	686061
5.181789	up	XM_001066470	LOC686082	686082
3.8070154	up	XM_001066478	LOC686085	686085
3.3964949	up	XM_001066607	LOC686119	686119
3.7423563	up	XM_001066636	LOC686130	686130
2.6460164	up	XM_001066637	LOC686131	686131
2.1358685	up	XM_001066698	LOC686147	686147
3.0563257	up	XM_001072947	LOC686222	686222
2.2587068	up	XM_001073659	LOC686342	686342
3.9584856	up	XM_001073953	LOC686402	686402
2.1841714	up	XM_001075122	LOC686647	686647
2.6662457	up	XM_001075396	LOC686718	686718
3.2948928	up	XM_001075397	LOC686719	686719
2.0315032	up	XM_001076338	LOC686921	686921
2.6386366	up	XM_001076379	LOC686934	686934
3.3898118	up	XM_001076692	LOC687003	687003
2.5835993	up	XM_001076767	LOC687021	687021

3.713098	up	XM_001076927	LOC687062	687062
4.743295	up	XM_001076949	LOC687067	687067
4.506484	up	XM_001077135	LOC687109	687109
2.021484	up	XM_001077138	LOC687111	687111
2.9060848	up	XM_001077315	LOC687164	687164
4.360078	up	XM_001077430	LOC687185	687185
2.2972953	up	XM_001078171	LOC687363	687363
2.017497	up	XM_001078386	LOC687401	687401
3.5423775	up	XM_001078688	LOC687464	687464
2.4795537	up	XM_001078885	LOC687512	687512
4.024105	up	XM_001079008	LOC687537	687537
3.3258305	up	XM_001079286	LOC687589	687589
2.141709	up	XM_001079425	LOC687619	687619
2.8015978	up	XM_001079569	LOC687652	687652
9.437647	up	XM_001079796	LOC687695	687695
5.1928735	up	XM_001079968	LOC687736	687736
3.4130542	up	XM_001081032	LOC688068	688068
2.3378341	up	XM_001081092	LOC688091	688091
2.6967735	up	XM_001081232	LOC688127	688127
3.2944028	up	XM_001081341	LOC688160	688160
4.6166177	up	XM_001081359	LOC688164	688164
2.0792835	up	XM_001081553	LOC688228	688228
3.0688634	up	XM_001081629	LOC688246	688246
3.1672816	up	XM_001081886	LOC688351	688351
2.956007	up	XM_001081935	LOC688377	688377
4.098281	up	XM_001081948	LOC688384	688384
6.6765323	up	XM_001066775	LOC688399	688399
6.2789164	up	XM_001066825	LOC688410	688410
4.449366	up	XM_001066895	LOC688428	688428
7.6754985	up	XM_001066958	LOC688441	688441
3.4063847	up	XM_001066993	LOC688450	688450
3.0382729	up	XM_001067045	LOC688460	688460
4.29556	up	XM_001067063	LOC688464	688464
3.4133735	up	XM_001056972	LOC688479	688479
2.378941	up	XM_001067241	LOC688519	688519
2.151363	up	XM_001067313	LOC688532	688532
4.011219	up	XM_001067327	LOC688538	688538
2.9315126	up	XM_001067419	LOC688560	688560
2.8522863	up	XM_001058486	LOC688572	688572
3.8058994	up	XM_001067520	LOC688587	688587
4.7494636	up	XM_001067531	LOC688592	688592
3.2666585	up	XM_001067734	LOC688644	688644
3.905089	up	XM_001058542	LOC688649	688649
4.2294264	up	XM_001067795	LOC688663	688663

2.8035886	up	XM_001067853	LOC688678	688678
3.0967205	up	XM_001058650	LOC688679	688679
3.4889815	up	XM_001058713	LOC688687	688687
2.2801547	up	XM_001067922	LOC688690	688690
2.7682452	up	XM_001068087	LOC688732	688732
3.0364027	up	XM_001068318	LOC688793	688793
2.2644835	up	XM_001068329	LOC688798	688798
3.2865016	up	XM_001068371	LOC688803	688803
3.4440663	up	XM_001068419	LOC688818	688818
2.8810127	up	XM_001068465	LOC688825	688825
2.6548297	up	XM_001068481	LOC688831	688831
3.466348	up	XM_001068669	LOC688874	688874
4.367969	up	XM_001068717	LOC688892	688892
2.7676826	up	XM_001068783	LOC688909	688909
5.1428494	up	XM_001068891	LOC688935	688935
3.0228784	up	XM_001068988	LOC688964	688964
7.0195518	up	XM_001069010	LOC688970	688970
2.6729171	up	XM_001069134	LOC689001	689001
3.9325895	up	XM_001069178	LOC689016	689016
2.1123164	up	XM_001069258	LOC689032	689032
2.7775264	up	XM_001069422	LOC689076	689076
2.5475974	up	XM_001069448	LOC689083	689083
2.0508358	up	XM_001058891	LOC689084	689084
2.0377884	up	XM_001058953	LOC689094	689094
8.283908	up	XM_001069556	LOC689107	689107
2.205693	up	XM_001069621	LOC689124	689124
8.417965	up	XM_001069756	LOC689150	689150
2.8548913	up	XM_001069925	LOC689190	689190
7.518614	up	XM_001069934	LOC689193	689193
6.265004	up	XM_001069974	LOC689206	689206
2.3274813	up	XM_001069993	LOC689215	689215
4.069657	up	XM_001070040	LOC689225	689225
5.0917754	up	XM_001070055	LOC689228	689228
3.2991405	up	XM_001070064	LOC689231	689231
2.4769762	up	XM_001070094	LOC689241	689241
3.4436588	up	XM_001070111	LOC689244	689244
2.1309729	up	XM_001070188	LOC689268	689268
4.3957415	up	XM_001070205	LOC689270	689270
5.110301	up	XM_001070335	LOC689299	689299
3.3359787	up	XM_001070367	LOC689312	689312
6.058637	up	XM_001070376	LOC689315	689315
2.4716702	up	XM_001070403	LOC689326	689326
2.5243797	up	XM_001059417	LOC689329	689329
3.4421613	up	XM_001070438	LOC689338	689338

5.8158975	up	XM_001070459	LOC689342	689342
2.5055	up	XM_001054976	LOC689347	689347
2.5016158	up	XM_001070518	LOC689362	689362
7.7163773	up	XM_001070539	LOC689368	689368
2.686199	up	XM_001070689	LOC689410	689410
2.1979485	up	XM_001070729	LOC689418	689418
2.5141811	up	XM_001070788	LOC689435	689435
3.9503222	up	XM_001070864	LOC689453	689453
2.6563764	up	XM_001070948	LOC689478	689478
2.0915172	up	XM_001070998	LOC689497	689497
2.4259186	up	XM_001070999	LOC689498	689498
2.2803326	up	XM_001071038	LOC689511	689511
2.6866617	up	XM_001060123	LOC689545	689545
3.154512	up	XM_001071174	LOC689554	689554
2.0190039	up	XM_001071448	LOC689635	689635
3.0517743	up	XM_001071539	LOC689658	689658
4.004074	up	XM_001055155	LOC689667	689667
2.0406084	up	XM_001071623	LOC689677	689677
2.26356	up	XM_001071624	LOC689678	689678
2.71687	up	XM_001060291	LOC689680	689680
2.1169782	up	XM_001055335	LOC689690	689690
2.9498162	up	XM_001071675	LOC689692	689692
2.2620943	up	XM_001071797	LOC689727	689727
2.0205173	up	XM_001071809	LOC689730	689730
3.0829835	up	XM_001071817	LOC689734	689734
2.5237398	up	XM_001071877	LOC689750	689750
2.6073005	up	XM_001071886	LOC689753	689753
6.1791935	up	XM_001071936	LOC689767	689767
2.140748	up	XM_001072032	LOC689796	689796
3.4269464	up	XM_001070608	LOC689797	689797
2.6058636	up	XM_001072049	LOC689799	689799
2.5797083	up	XM_001072069	LOC689802	689802
5.7846413	up	XM_001072168	LOC689827	689827
4.984366	up	XM_001072188	LOC689829	689829
2.8768764	up	XM_001072212	LOC689837	689837
2.6307254	up	XM_001072245	LOC689846	689846
8.004454	up	XM_001072263	LOC689850	689850
2.6983962	up	XM_001072288	LOC689857	689857
2.0292385	up	XM_001060657	LOC689870	689870
3.7182326	up	XM_001072354	LOC689878	689878
2.0790918	up	XM_001069091	LOC689927	689927
3.6283352	up	XM_001072652	LOC689948	689948
2.2924116	up	XM_001072684	LOC689957	689957
2.027531	up	XM_001072685	LOC689958	689958

3.8236458	up	XM_001072858	LOC689999	689999
2.1842856	up	XM_001072965	LOC690024	690024
3.5577593	up	XM_001073001	LOC690039	690039
3.4780698	up	XM_001073030	LOC690048	690048
2.0633712	up	XM_001073060	LOC690054	690054
3.1267157	up	XM_001073178	LOC690081	690081
3.0257201	up	XM_001073344	LOC690122	690122
2.6645453	up	XM_001073403	LOC690135	690135
2.4316936	up	XM_001073445	LOC690145	690145
4.6462855	up	XM_001073536	LOC690167	690167
2.6609151	up	XM_001073545	LOC690170	690170
3.491554	up	XM_001073746	LOC690228	690228
3.3121543	up	XM_001073951	LOC690281	690281
3.7940319	up	XM_001074009	LOC690298	690298
2.7225764	up	XM_001074152	LOC690341	690341
4.1685853	up	XM_001074233	LOC690363	690363
4.4550753	up	XM_001074284	LOC690379	690379
2.099612	up	XM_001074324	LOC690395	690395
5.219481	up	XM_001074336	LOC690398	690398
2.5336154	up	XM_001074345	LOC690400	690400
2.9989667	up	XM_001074400	LOC690415	690415
3.4276884	up	XM_001074459	LOC690439	690439
2.4338694	up	XM_001074469	LOC690443	690443
3.978992	up	XM_001074485	LOC690452	690452
3.093233	up	XM_001074498	LOC690457	690457
3.354503	up	XM_001074527	LOC690467	690467
2.5354156	up	XM_001074545	LOC690474	690474
2.7469923	up	XM_001074598	LOC690490	690490
3.4559252	up	XM_001074680	LOC690523	690523
3.7197988	up	XM_001074751	LOC690542	690542
3.644155	up	XM_001074825	LOC690565	690565
2.531357	up	XM_001074852	LOC690577	690577
3.0684261	up	XM_001074893	LOC690590	690590
2.006835	up	XM_001074985	LOC690619	690619
2.527783	up	XM_001075006	LOC690626	690626
2.6242597	up	XM_001075069	LOC690643	690643
2.5525482	up	XM_001075121	LOC690659	690659
3.901042	up	XM_001075162	LOC690672	690672
2.4047966	up	XM_001075284	LOC690700	690700
2.9781022	up	XM_001075303	LOC690707	690707
3.8087535	up	XM_001075438	LOC690739	690739
3.7106824	up	XM_001075495	LOC690748	690748
2.5637827	up	XM_001075604	LOC690782	690782
4.0664716	up	XM_001075725	LOC690815	690815

2.4743643	up	XM_001075795	LOC690828	690828
2.5553162	up	XM_001075885	LOC690854	690854
2.0747576	up	XM_001075892	LOC690855	690855
3.402658	up	XM_001075937	LOC690867	690867
4.226243	up	XM_001075967	LOC690874	690874
3.3478217	up	XM_001076048	LOC690892	690892
3.4504194	up	XM_001076066	LOC690896	690896
2.1039941	up	XM_001076169	LOC690924	690924
2.5286887	up	XM_001076188	LOC690929	690929
3.9049275	up	XM_001076268	LOC690947	690947
2.1316879	up	XM_001076337	LOC690969	690969
3.1919498	up	XM_001076377	LOC690977	690977
2.2396812	up	XM_001076453	LOC690998	690998
3.5830152	up	XM_001076823	LOC691094	691094
4.576451	up	XM_001076856	LOC691108	691108
2.3956227	up	XM_001076889	LOC691115	691115
2.8814528	up	XM_001076891	LOC691117	691117
2.0542603	up	XM_001076976	LOC691142	691142
3.0206861	up	XM_001076998	LOC691146	691146
2.9953172	up	XM_001077250	LOC691219	691219
2.9106479	up	XM_001077260	LOC691221	691221
2.4106665	up	XM_001077347	LOC691241	691241
2.1160529	up	XM_001077501	LOC691274	691274
3.1043437	up	XM_001077529	LOC691282	691282
4.805065	up	XM_001077758	LOC691333	691333
2.006458	up	XM_001077759	LOC691334	691334
3.4453483	up	XM_001077906	LOC691366	691366
3.0920975	up	XM_001078062	LOC691395	691395
4.1910596	up	XM_001078077	LOC691401	691401
2.7226617	up	XM_001078110	LOC691406	691406
2.8312266	up	XM_001078125	LOC691410	691410
2.5528908	up	XM_001078150	LOC691414	691414
5.3495955	up	XM_001078322	LOC691451	691451
2.3767042	up	XM_001078439	LOC691473	691473
3.2269053	up	XM_001078495	LOC691487	691487
3.0677173	up	XM_001078554	LOC691502	691502
4.576375	up	XM_001078865	LOC691569	691569
4.009314	up	XM_001078898	LOC691575	691575
3.2461743	up	XM_001078899	LOC691576	691576
2.4927926	up	XM_001078931	LOC691586	691586
2.9482498	up	XM_001078958	LOC691595	691595
2.010119	up	XM_001078980	LOC691600	691600
2.4639459	up	XM_001078992	LOC691605	691605
2.4732537	up	XM_001079070	LOC691627	691627

2.376365	up	XM_001079096	LOC691633	691633
4.3190756	up	XM_001079224	LOC691672	691672
2.6233718	up	XM_001079285	LOC691692	691692
2.7193432	up	XM_001079331	LOC691705	691705
3.102685	up	XM_001079400	LOC691728	691728
3.118619	up	XM_001079476	LOC691744	691744
2.7989008	up	XM_001079486	LOC691748	691748
2.249638	up	XM_001079519	LOC691754	691754
2.4397707	up	XM_001079538	LOC691760	691760
2.5704696	up	XM_001079592	LOC691773	691773
3.8600826	up	XM_001079621	LOC691784	691784
2.8815794	up	XM_001079655	LOC691793	691793
2.1148543	up	XM_001079664	LOC691795	691795
3.8220792	up	XM_001079679	LOC691800	691800
2.8756976	up	XM_001079748	LOC691818	691818
2.208641	up	NM_022257	Masp1	64023
2.1531305	up	XM_001074994	Mettl11b	289167
2.3390467	up	NM_001024893	MGC114427	317297
2.3170342	up	NM_012861	Mgmt	25332
2.9807434	up	XM_001059515	Mogat1	363261
2.92419	up	XM_001063115	Muc6	282586
2.0061393	up	XM_001069193	Neurod4	288821
2.2018769	up	NM_030868	Nov	81526
2.0864694	up	NM_053611	Nupr1	113900
2.8628862	up	NM_001001388	Olr1093	366844
2.2405453	up	NM_001001092	Olr1395	405986
2.1156318	up	NM_001000957	Olr1451	405306
2.1111286	up	NM_001000772	Olr1453	405047
2.0200279	up	NM_001001115	Olr1707	406014
3.1416836	up	NM_001000410	Olr865	298474
2.0306091	up	NM_001000708	Olr876	404957
2.4925346	up	NM_001001358	Olr883	288803
2.039659	up	XM_001080669	Ovca2	497954
2.1359892	up	XM_001054954	Pabpc1l2b	302405
3.0212991	up	XM_001054581	Pmepa1	311676
2.0185883	up	NM_001013195	Pold4	361698
2.0825188	up	NM_001025637	Psmb10	291983
2.3287978	up	NM_001013427	Rarres2	297073
2.6756449	up	XM_225620	RGD1559615	291346
4.686946	up	XM_344831	RGD1559699	365132
2.669751	up	XM_227432	RGD1559837	295256
2.7126	up	XM_001055068	RGD1559932	298221
3.7759798	up	XM_344837	RGD1559946	365141
4.1497316	up	XM_576496	RGD1559960	501084

2.6854346	up	XM_001074365	RGD1559962	498988
2.1527157	up	XM_219714	RGD1559982	293850
3.2657053	up	XM_001061596	RGD1560019	367121
6.399465	up	XM_574777	RGD1560095	499454
2.0912526	up	XM_227345	RGD1560096	310590
7.084692	up	XM_574775	RGD1560135	499452
5.645248	up	XM_577949	RGD1560140	502469
4.5052032	up	XM_344641	RGD1560209	364800
4.4745073	up	XM_574066	RGD1560221	498780
3.5155787	up	XM_578421	RGD1560270	502920
4.844002	up	XM_579915	RGD1560485	499053
4.085121	up	XM_579909	RGD1560516	499038
2.4213412	up	XM_001055007	RGD1560539	502966
2.9080698	up	XM_574327	RGD1560543	499046
2.315347	up	XM_578061	RGD1560554	502576
4.890742	up	XM_577908	RGD1560555	502426
2.6637635	up	XM_229665	RGD1560570	302091
3.3456182	up	XM_001078732	RGD1560580	288646
4.6979766	up	XM_001054843	RGD1560585	317597
2.2537963	up	XM_573890	RGD1560677	498612
4.995419	up	XM_577919	RGD1560777	502438
4.7557235	up	XM_577734	RGD1560808	502273
2.0183775	up	XM_001058159	RGD1560813	498894
2.5029664	up	XM_001064817	RGD1560815	291550
6.5587196	up	XM_001065293	RGD1560825	685793
2.0093584	up	XM_001074010	RGD1560918	364248
3.9688377	up	XM_577704	RGD1560972	502244
5.075892	up	XM_001054260	RGD1561185	502251
2.4912045	up	XM_576388	RGD1561216	500978
6.6498294	up	XM_001081347	RGD1561231	291345
2.4297156	up	XM_217531	RGD1561273	302093
2.3062148	up	XM_580123	RGD1561305	500808
6.3571653	up	XM_577732	RGD1561339	502271
9.83149	up	XM_574763	RGD1561401	499440
3.9402776	up	XM_001059953	RGD1561425	301378
5.549834	up	XM_577708	RGD1561446	502249
7.250037	up	XM_574061	RGD1561493	498775
5.954323	up	XM_229383	RGD1561546	301899
2.1157856	up	XM_345090	RGD1561557	365547
2.3512168	up	XM_001079552	RGD1561654	295557
2.3597782	up	XM_001054195	RGD1561667	308209
2.6443114	up	XM_577125	RGD1561684	501721
2.233384	up	XM_001060826	RGD1561694	498388
2.1620195	up	XM_001064028	RGD1561705	499290

3.35752	up	XM_344825	RGD1561706	365122
3.7564998	up	XM_217910	RGD1561759	308183
2.2986474	up	XM_001053629	RGD1561828	679595
5.4129987	up	XM_577947	RGD1562058	502467
3.9096904	up	XM_001076534	RGD1562205	307168
3.1116505	up	XM_578760	RGD1562282	367183
2.3090267	up	XM_577381	RGD1562612	501954
2.3654335	up	XM_001071626	RGD1562624	364249
3.0283484	up	XM_001054943	RGD1562650	502965
4.383787	up	XM_577950	RGD1562670	502470
3.9736614	up	XM_344833	RGD1562677	365136
5.254635	up	XM_574093	RGD1562707	498809
3.501679	up	XM_576389	RGD1562714	500979
3.0681484	up	XM_577935	RGD1562893	502454
2.7753866	up	XM_577932	RGD1562919	502451
4.533081	up	XM_001071176	RGD1562931	502439
2.9703107	up	XM_344812	RGD1562948	365096
3.2225876	up	XM_579906	RGD1563107	499034
6.576076	up	XM_001081417	RGD1563149	502466
4.48325	up	XM_344630	RGD1563247	364781
2.8895278	up	XM_574318	RGD1563490	499025
4.7430835	up	XM_344827	RGD1563626	365125
7.1279206	up	XM_001080342	RGD1563723	502462
3.6591501	up	XM_345920	RGD1563738	367059
4.3960786	up	XM_577701	RGD1563741	502241
9.867194	up	XM_001081170	RGD1563747	499439
2.3994462	up	XM_214327	RGD1563748	290675
9.534455	up	XM_577939	RGD1563772	502458
3.3109164	up	XM_240367	RGD1563825	306366
2.546369	up	XM_577929	RGD1563833	502448
3.0685246	up	XM_343520	RGD1563862	363180
6.0969696	up	XM_344835	RGD1563865	365139
4.753726	up	XM_577705	RGD1564000	502246
2.3314047	up	XM_001059672	RGD1564033	680963
9.517638	up	XM_574773	RGD1564135	499450
3.1573648	up	XM_573954	RGD1564205	498671
2.743313	up	XM_001079282	RGD1564285	299662
3.518623	up	XM_001072043	RGD1564317	498054
2.6595776	up	XM_001078658	RGD1564355	307681
2.5580685	up	XM_001063065	RGD1564519	498072
9.169112	up	XM_574764	RGD1564568	499441
6.5566564	up	XM_574778	RGD1564734	499455
2.3039901	up	XM_001070478	RGD1564736	367932
2.5463405	up	XM_574052	RGD1564791	498766

4.9813733	up	XM_577726	RGD1564848	502265
6.746093	up	XM_001076029	RGD1564977	502457
6.358895	up	XM_001071285	RGD1565094	502437
3.7318435	up	XM_001080335	RGD1565115	499449
8.611178	up	XM_001075702	RGD1565191	499451
3.0094273	up	XM_574322	RGD1565209	499033
3.9128575	up	XM_001054671	RGD1565231	365140
3.288377	up	XM_344642	RGD1565321	364801
5.279063	up	XM_001054516	RGD1565379	498770
2.1669135	up	XM_344843	RGD1565390	365149
4.4486275	up	XM_576498	RGD1565421	501086
2.556705	up	XM_343720	RGD1565423	363385
3.212835	up	XM_001068143	RGD1565430	367928
5.131314	up	XM_574776	RGD1565550	499453
8.959835	up	XM_577941	RGD1565576	502460
2.21782	up	XM_577714	RGD1565723	502254
3.5661001	up	XM_001063482	RGD1565859	499291
2.653893	up	XM_233160	RGD1565911	298220
5.0033393	up	XM_001053399	RGD1566008	317604
4.5692835	up	XM_344619	RGD1566040	364759
2.2249794	up	XM_574062	RGD1566047	498776
2.0053642	up	NM_001033060	Rpain	287463
2.0323105	up	XM_001063809	Rragd	297960
2.0117478	up	NM_001007636	S100a1	295214
2.0313382	up	NM_053681	S100a3	114216
2.2619948	up	XM_001061870	Satl1	302325
2.0129664	up	XM_001066597	Scand1	362252
2.707591	up	NM_199082	Sectm1b	287884
2.3605754	up	NM_080892	Selenbp1	140927
3.0886083	up	NM_017310	Sema3a	29751
2.0369534	up	XM_001063428	Sesn1	294518
2.662554	up	XM_001065174	Sfrp2	310552
3.3846195	up	NM_053544	Sfrp4	89803
2.678362	up	XM_217877	Smok2a	292316
2.0414772	up	XM_001058775	Smpd4	303790
2.3384128	up	XM_001058102	Snrpg	681031
2.1397648	up	XM_001057944	Snx2	291464
3.4700701	up	XM_001068302	Sox4	364712
2.4041092	up	NM_001009976	Spetex-2A	364261
3.6456068	up	NM_001011701	Spetex-2D	364260
2.8843484	up	NM_001009968	Spetex-2F	316940
4.03226	up	XM_001073572	Spetex-2G	361099
3.9324453	up	NM_001009969	Spetex-2H	361008
2.4246745	up	NM_021869	Stx7	60466

2.0986614	up	NM_001009618	Sub1	192269
4.8174334	up	NM_133547	Sult1c2	171072
3.3346868	up	NM_001013177	Sult1c2a	316153
2.3550239	up	NM_001007605	Svs3	192239
2.107874	up	NM_175590	Taar7e	294133
2.0230646	up	XM_001067756	Tagap	308097
2.1103387	up	NM_001015008	Tcea3	298559
2.2795045	up	XM_001073003	Tdpoz1	365854
2.2002795	up	NM_001009703	Tmed9	361207
2.5151005	up	NM_001009709	Tmem140	362334
2.0902395	up	XM_001062611	Tmem70	500384
2.396498	up	NM_001008953	V1re18	494299
2.4397182	up	NM_001008946	Vom1r29	494292
3.3853936	up	XM_001053213	Vom2r1	678740
2.5437665	up	XM_218106	Vom2r4	308248
2.2569673	up	XM_001070665	Vom2r65	689403
2.0599785	up	XM_001062818	Vpreb2	363830
2.1194217	up	XM_001080583	Vps53	287535
2.1313963	up	XM_001059030	Wnt2	114487
2.0093808	up	NM_053402	Wnt4	84426
3.9828842	up	XM_001073004	Xlr3a	690041
4.701024	up	XM_001056023	Zmynd17	289904
2.136492	up	XM_001076652	Znf575	308430

MiR-434-3p overexpression _vs_Control 2.0 fold down regulated genes

Fold change	Regulation	ACCESSION	GeneSymbol	GeneID
2.0157707	down	NM_013040	Abcc9	25560
4.4395676	down	NM_053922	Acacb	116719
2.0231595	down	NM_013084	Acadsb	25618
3.0987487	down	NM_172009	Ache	83817
2.7171745	down	NM_017340	Acox1	50681
2.0521078	down	NM_019183	Actc1	29275
2.9059446	down	NM_031675	Actn4	63836
2.3093443	down	NM_199230	Acvr1b	29381
2.1679087	down	XM_001067637	Adck2	312258
2.0552995	down	NM_031552	Add3	25230
2.022554	down	XM_001063833	Agbl1	308724
2.9907243	down	XM_001068488	Ahcyl1	362013
2.7331386	down	XM_001078032	Ahnak	191572
2.9400535	down	XM_001077954	Aif1l	362107
2.0304124	down	NM_001011974	Akap2	298024
2.9877937	down	NM_033230	Akt1	24185
3.2727547	down	NM_017093	Akt2	25233

2.254635	down	NM_031010	Alox15	81639
2.4924178	down	XM_001062954	Amfr	361367
2.2336266	down	XM_001056974	Amot	300289
4.5548353	down	NM_031544	Ampd3	25095
3.7188325	down	NM_133569	Angptl2	171100
2.8204174	down	XM_342337	Ank2	362036
2.1577528	down	NM_053714	Ankh	114506
3.4566061	down	NM_013220	Ankrd1	27064
2.0516331	down	NM_001012148	Ankrd13a	360823
3.2760317	down	XM_001055096	Ankrd2	309374
2.2958615	down	XM_001056748	Ankrd23	316330
2.2298198	down	XM_001066475	Ano1	309135
2.1787488	down	NM_001014255	Aph1a	365872
2.5272114	down	NM_012778	Aqp1	25240
3.0224397	down	NM_001012032	Arhgap24	305156
3.2634416	down	NM_001007005	Arhgdia	360678
2.2052927	down	NM_001005565	Arhgef6	363509
2.1500762	down	NM_207165	Arl10	306767
2.2495475	down	NM_001009455	Armc5	361653
2.0812266	down	XM_001071122	Armc6	306352
3.19201	down	NM_012780	Arnt	25242
2.5981114	down	XM_001074246	Ash1l	310638
2.3158536	down	NM_013113	Atp1b1	25650
2.1672573	down	NM_012507	Atp1b2	24214
2.15615	down	NM_017290	Atp2a2	29693
3.2743301	down	NM_031604	Atp6v0a1	29757
2.4691823	down	NM_001009539	B4galt3	494342
2.7381532	down	NM_001011936	Bag3	293524
2.0126724	down	NM_212462	Bat2	294250
4.324102	down	XM_001080943	Best3	314847
3.0631297	down	NM_133582	Blcap	171113
2.0410476	down	XM_001061326	Bsdc1	297890
2.4812295	down	NM_001013185	Cabc1	360887
2.6874948	down	NM_012920	Camk2a	25400
2.0116816	down	NM_001042354	Camk2b	24245
2.0862088	down	NM_012519	Camk2d	24246
2.00433	down	XM_001079889	Camta2	287462
2.3629408	down	NM_019152	Capn1	29153
2.0281074	down	XM_001052977	Caprin1	362173
2.5880685	down	NM_001030041	Carm1	363026
2.1792564	down	NM_147144	Casc3	259170
2.5460916	down	XM_001081695	Caskin2	303678
2.1301687	down	NM_017131	Casq2	29209
3.0259922	down	XM_001054087	Ccdc109a	294560

2.0924473	down	NM_001080151	Ccdc21	362622
2.112702	down	NM_001014061	Ccdc91	312863
2.10346	down	XM_001061914	Cd163	312701
2.6131516	down	NM_012752	Cd24	25145
4.048762	down	NM_001012164	Cd97	361383
2.2469764	down	XM_228203	Cdc2l6	309804
2.0928333	down	XM_001057656	Cdh5	307618
13.018074	down	NM_080782	Cdkn1a	114851
3.2872384	down	NM_001014097	Cdv3	315970
2.0057075	down	XM_001081194	Cenpb	362217
2.4095623	down	XM_001058641	Chd2	308738
2.1201797	down	NM_024139	Chp	64152
2.4877882	down	XM_001059575	Chrd	117275
5.023558	down	NM_019145	Chrng	25753
2.349006	down	XM_001053715	Clasp1	304740
2.0957475	down	NM_053363	Clcn3	84360
2.0344222	down	NM_022198	Clcn4-2	60586
2.1788273	down	NM_031818	Clic4	83718
2.8674033	down	NM_001002022	Clint1	360515
2.674918	down	XM_001076134	Col4a2	306628
2.6228151	down	XM_001068252	Col6a3	367313
2.4325054	down	NM_173303	Cox6c1	286962
2.408513	down	XM_001064017	Cpeb4	303010
2.006819	down	NM_019302	Crk	54245
2.3598657	down	NM_057144	Csrp3	117505
2.039621	down	XM_001053648	Ctps	313560
2.4962661	down	NM_021868	Cttn	60465
2.2306993	down	XM_001079353	Cyb5d1	363629
2.0155656	down	NM_138877	Cyb5r3	25035
2.0743167	down	NM_001014164	Cybasc3	361729
3.4897575	down	XM_001074892	Dag1	114489
2.613898	down	NM_001009686	Dcaf11	305895
3.0845237	down	NM_053404	Dctn4	84428
2.7038054	down	XM_001068407	Dda1	688813
2.5610852	down	NM_001012345	Dgat2	252900
2.4540606	down	XM_001081462	Dhx58	303538
2.6752222	down	NM_001006981	Dlst	299201
2.408337	down	NM_001005246	Dmd	24907
5.293943	down	XM_001066025	Dmpk	308405
2.6779027	down	NM_001025411	Dnaja4	300721
3.369737	down	XM_001061825	Dnajb5	313811
2.1354055	down	NM_013199	Dnm2	25751
2.0241542	down	XM_001057815	Dupd1	361003
2.0517015	down	XM_001064583	Dusp10	63995

2.340322	down	NM_031820	Dvl1	83721
2.1390088	down	NM_053596	Ece1	94204
2.1924262	down	NM_012660	Eef1a2	24799
4.412796	down	NM_012551	Egr1	24330
2.0820165	down	XM_001074569	Ehd1	293692
2.819918	down	NM_001025660	Ei24	300514
2.6495502	down	XM_001062415	Eif4g2-ps2	308446
2.008494	down	NM_001033681	Eif5a	287444
2.0373385	down	XM_001070626	Ell	306347
2.2154837	down	XM_001080308	Enpep	64017
3.2331913	down	XM_001068428	Enpp4	301261
2.861715	down	NM_203336	Esrrg	360896
2.0182095	down	XM_001068194	Ewsr1	289752
2.6711595	down	XM_001059758	Eya1	502935
2.000845	down	XM_001054174	Eya4	292172
2.1226408	down	XM_001081456	Ezh1	303547
2.2748163	down	NM_053445	Fads1	84575
2.2884145	down	NM_173137	Fads3	286922
2.3765354	down	XM_001057799	Fam134a	363252
2.0708854	down	XM_001071695	Fam189b	310640
2.5292027	down	XM_001063835	Fbxw11	303024
2.1894717	down	NM_024146	Fgfr1	79114
3.0932035	down	NM_001033926	Fhl1	25177
5.040366	down	XM_001056311	Fhl3	313582
2.2691858	down	NM_001014120	Fkbp10	360627
6.957848	down	XM_001061717	Flnc	362332
2.4400327	down	XM_001070746	Fndc3a	306022
2.1024334	down	XM_001057133	Fndc3b	294925
2.1183698	down	NM_133527	Folr1	171049
2.1585858	down	NM_022197	Fos	314322
2.7999067	down	XM_001055972	Foxd4	252886
3.4057667	down	XM_001074052	Foxj3	313554
2.63487	down	XM_001065012	Foxn3	314374
2.294917	down	XM_001062337	Fsd2	308779
2.1211357	down	NM_001039337	Fubp3	362106
2.130813	down	XM_001079469	Fxr2	287433
2.1940057	down	NM_022008	Fxyd7	63848
3.1590736	down	NM_001009632	G0s2	289388
2.048305	down	NM_199118	Gaa	367562
3.21024	down	NM_001017486	Gbas	498174
3.1502433	down	XM_001066196	Gbf1	309451
2.1029053	down	NM_017088	Gdi1	25183
2.582242	down	NM_001011994	Gga1	300066
2.9360895	down	NM_031814	Git1	83709

2.2183626	down	NM_017211	Glg1	29476
2.1453307	down	XM_001065857	Glt25d1	290637
2.5554016	down	NM_172335	Gm2a	282838
2.0562377	down	NM_001013119	Gna13	303634
2.8366694	down	NM_001011989	Gns	299825
2.0235262	down	XM_001062460	Golim4	310526
2.3761907	down	NM_013177	Got2	25721
3.129917	down	NM_030828	Gpc1	58920
3.3112435	down	NM_022215	Gpd1	60666
2.0151246	down	XM_001074228	Gpihbp1	300027
2.4728868	down	NM_031039	Gpt	81670
2.9110298	down	NM_001012057	Gpt2	307759
2.5342813	down	NM_001009920	Gsta5	494500
2.1495667	down	XM_574740	Gstt3	499422
2.344897	down	XM_001058711	Gtpbp2	363195
4.478064	down	XM_001076928	Gys1	690987
2.0419738	down	NM_001079940	Habp4	361196
4.8743625	down	NM_012945	Hbegf	25433
3.114225	down	NM_019387	Hgs	56084
2.0916438	down	XM_001072438	Hip1r	81917
3.6266866	down	XM_001066707	Hipk2	362342
3.5429852	down	NM_001080783	Hisppd2a	311355
2.0765824	down	NM_024137	Hivep2	29721
2.9080527	down	NM_012735	Hk2	25059
2.5211995	down	NM_012580	Hmox1	24451
2.138936	down	XM_001076759	Hnrnpll2	311776
2.2944856	down	NM_138887	Hspb6	192245
23.174852	down	XM_001072790	Hspb7	50565
2.0007193	down	XM_341259	Hspc159	360983
2.7245631	down	NM_031721	Htra1	65164
2.386734	down	XM_001075074	Ints3	361988
2.3744712	down	NM_001033691	Irf7	293624
2.6328511	down	XM_001059353	Itga6	114517
2.6188107	down	XM_001069915	Itgb1bp2	317258
3.6116076	down	NM_001004263	Itgb6	311061
2.0157993	down	XM_001058872	Itpk1	500709
2.034496	down	NM_021835	Jun	24516
2.3735986	down	NM_013186	Kcnb1	25736
2.3448172	down	NM_031358	Kcnj11	83535
2.5191863	down	NM_031828	Kcnma1	83731
2.1655066	down	NM_133602	Khsrp	171137
2.3750033	down	XM_001058501	Kif13a	308173
2.628528	down	NM_053486	Kif3c	85248
2.0068946	down	XM_214331	Klhl2	290692

2.667188	down	XM_001065835	Larp5	307070
2.9186301	down	XM_001060474	Lclat1	362702
2.7838018	down	XM_001058773	Ldb1	309447
2.8179986	down	XM_001059372	Ldb3	498587
2.4149106	down	NM_012595	Ldhb	24534
2.2234929	down	XM_001056203	Leng4	308309
2.2909951	down	NM_020099	Leprot	56766
2.034387	down	NM_139096	Lgals3bp	245955
2.3528314	down	NM_012977	Lgals9	25476
2.2010205	down	XM_001078972	Limd1	316101
2.358569	down	NM_152844	Llgl1	54265
2.3238568	down	XM_001076072	Lmcd1	494021
2.031095	down	NM_001079939	Lmf2	315218
2.3398912	down	XM_001076027	Lmnb2	299625
2.4541237	down	NM_001013853	LOC287167	287167
4.6755524	down	XM_001054688	LOC291746	291746
2.7550628	down	XM_001054611	LOC292662	292662
2.5687125	down	XM_001066983	LOC297826	297826
2.904464	down	XM_001059270	LOC298016	298016
2.7751424	down	XM_001054228	LOC300481	300481
4.1906	down	NM_001037190	LOC303448	303448
2.1238563	down	XM_001080824	LOC360570	360570
3.103765	down	XM_001062239	LOC363737	363737
3.0002496	down	XM_001065988	LOC363903	363903
2.2282526	down	XM_001064135	LOC365219	365219
2.4618766	down	XM_001077769	LOC365559	365559
3.2929974	down	XM_001058386	LOC366068	366068
2.652297	down	XM_345916	LOC367050	367050
3.2863681	down	XM_001071580	LOC367619	367619
2.6587489	down	XM_001076383	LOC499235	499235
4.9598417	down	XM_001078222	LOC499718	499718
3.2913065	down	XM_001053584	LOC499996	499996
2.081605	down	XM_001073134	LOC500912	500912
2.2035077	down	NM_001033072	LOC500959	500959
2.4369524	down	XM_577408	LOC501979	501979
3.1223638	down	XM_001063944	LOC502111	502111
5.959818	down	XM_001057051	LOC502730	502730
2.1178854	down	XM_001071495	LOC503366	503366
2.3183012	down	XM_001072828	LOC503393	503393
3.6563525	down	XM_578947	LOC503408	503408
4.238234	down	XM_001053228	LOC678801	678801
2.049402	down	XM_001053911	LOC678938	678938
3.063879	down	XM_001054963	LOC679221	679221
2.6705477	down	XM_001053597	LOC679586	679586

3.8458772	down	XM_001053642	LOC679597	679597
2.6281538	down	XM_001054023	LOC679685	679685
2.582184	down	XM_001054040	LOC679690	679690
2.735436	down	XM_001054162	LOC679715	679715
3.4197164	down	XM_001054617	LOC679826	679826
2.8781424	down	XM_001054952	LOC679920	679920
2.443848	down	XM_001055503	LOC680026	680026
2.134029	down	XM_001055430	LOC680036	680036
2.7055907	down	XM_001056856	LOC680363	680363
2.996659	down	XM_001056928	LOC680379	680379
3.252572	down	XM_001057008	LOC680393	680393
3.5817363	down	XM_001057446	LOC680499	680499
2.351425	down	XM_001058190	LOC680654	680654
2.6328077	down	XM_001058388	LOC680688	680688
3.90397	down	XM_001058740	LOC680755	680755
3.0433936	down	XM_001058966	LOC680805	680805
2.3900487	down	XM_001059174	LOC680848	680848
2.0174477	down	XM_001060542	LOC681153	681153
2.151028	down	XM_001060556	LOC681160	681160
5.109977	down	XM_001060821	LOC681222	681222
2.0849721	down	XM_001061373	LOC681354	681354
3.3148615	down	XM_001058015	LOC681699	681699
2.8943806	down	XM_001058263	LOC681754	681754
3.4369864	down	XM_001058730	LOC681858	681858
2.4786444	down	XM_001058855	LOC681893	681893
2.1434271	down	XM_001060977	LOC682316	682316
2.406557	down	XM_001062491	LOC682653	682653
3.3645396	down	XM_001063246	LOC682818	682818
2.0239916	down	XM_001063578	LOC682891	682891
3.8154705	down	XM_001064668	LOC683146	683146
3.2716873	down	XM_001065484	LOC683336	683336
2.4642944	down	XM_001070523	LOC683342	683342
2.1355946	down	XM_001065874	LOC683420	683420
2.2711065	down	XM_001065932	LOC683435	683435
2.36783	down	XM_001065985	LOC683447	683447
2.3440478	down	XM_001066111	LOC683479	683479
3.2119803	down	XM_001066181	LOC683494	683494
2.831613	down	XM_001066577	LOC683573	683573
2.557338	down	XM_001067070	LOC683689	683689
4.2173586	down	XM_001053482	LOC683761	683761
2.2830844	down	XM_001064232	LOC683788	683788
2.3330104	down	XM_001068630	LOC684035	684035
2.0067234	down	XM_001069378	LOC684202	684202
2.8819327	down	XM_001069774	LOC684297	684297

5.0293927	down	XM_001069904	LOC684333	684333
2.3754933	down	XM_001070490	LOC684352	684352
3.3041024	down	XM_001070213	LOC684399	684399
3.461679	down	XM_001072008	LOC684806	684806
3.5378366	down	XM_001063592	LOC685387	685387
2.6635602	down	XM_001064406	LOC685580	685580
2.4523764	down	XM_001065305	LOC685797	685797
2.688678	down	XM_001065503	LOC685851	685851
2.5693204	down	XM_001066160	LOC686011	686011
3.9118657	down	XM_001072758	LOC686172	686172
2.2749832	down	XM_001075155	LOC686658	686658
2.0496838	down	XM_001076367	LOC686931	686931
3.9322348	down	XM_001079384	LOC687610	687610
2.3016253	down	XM_001079878	LOC687713	687713
2.19288	down	XM_001080031	LOC687759	687759
2.165977	down	XM_001080804	LOC687991	687991
2.5332327	down	XM_001080924	LOC688029	688029
3.2382755	down	XM_001081883	LOC688349	688349
2.0921755	down	XM_001066874	LOC688424	688424
2.1054313	down	XM_001066030	LOC688462	688462
2.1348352	down	XM_001067817	LOC688667	688667
2.2238567	down	XM_001068479	LOC688829	688829
4.479032	down	XM_001070462	LOC689343	689343
4.413225	down	XM_001070764	LOC689427	689427
2.523	down	XM_001070770	LOC689428	689428
2.9251237	down	XM_001071429	LOC689628	689628
2.2046938	down	XM_001071836	LOC689741	689741
3.706674	down	XM_001072404	LOC689891	689891
2.089512	down	XM_001072820	LOC689989	689989
2.028385	down	XM_001073955	LOC690000	690000
2.0863266	down	XM_001073272	LOC690103	690103
3.0376253	down	XM_001075530	LOC690761	690761
4.230433	down	XM_001072433	LOC691098	691098
2.1238127	down	XM_001077205	LOC691204	691204
3.0429158	down	XM_001077221	LOC691211	691211
2.908142	down	XM_001077271	LOC691224	691224
2.7685645	down	XM_001077366	LOC691245	691245
2.1615086	down	XM_001079338	LOC691708	691708
2.1791866	down	XM_001080361	LOC691960	691960
2.4274585	down	NM_001012189	Lpcat3	362434
3.1072745	down	NM_012598	Lpl	24539
2.062748	down	XM_001070537	Lrrc25	498605
2.4408135	down	NM_001008280	Lrrc59	287633
2.0914018	down	NM_001017467	Ly6e	362934

2.8753772	down	NM_001008319	Maea	298982
2.499425	down	NM_145673	Mafk	246760
2.0529912	down	NM_030995	Map1a	25152
2.5211537	down	NM_053703	Map2k6	114495
3.5694008	down	XM_001081582	Map3k3	303604
2.048856	down	NM_001005556	Mavs	311430
2.0224288	down	NM_001024996	Mblac1	304346
2.3595693	down	XM_001062557	Mbnl1	282635
3.4273758	down	NM_001014035	Mef2a	309957
2.3847256	down	NM_001040186	Mfge8	25277
2.0067844	down	XM_001063359	Mfsd6	301388
2.881787	down	NM_001007751	MGC94207	362946
4.1508417	down	NM_001013964	Mgrn1	302938
2.0359304	down	XM_215562	Mgst2	295037
3.3430097	down	XM_001074357	Mical2	365352
2.544309	down	NM_134399	Mk1	171436
3.2678561	down	XM_001059647	Mll5	311968
2.7390385	down	XM_001061133	Mpp3	114202
2.0625107	down	XM_001056335	Mreg	501162
4.720925	down	NM_030863	Msn	81521
2.37339	down	NM_022588	Mta1	64520
2.4164085	down	XM_001059230	Mtmr1	317296
3.6082273	down	XM_001055709	Murc	313225
2.16103	down	XM_001067239	Mut	688517
2.0857453	down	NM_031063	Mvk	81727
2.6358612	down	NM_173096	Mx1	24575
2.4955485	down	NM_134350	Mx2	286918
2.6472802	down	NM_183332	Myadm	369016
16.525509	down	NM_017240	Myh7	29557
3.226564	down	XM_001078049	Myh8	252942
2.0340161	down	NM_013194	Myh9	25745
8.329767	down	NM_012606	Myl3	24585
2.757478	down	XM_239684	Myo18b	304551
2.5701942	down	NM_023092	Myo1c	65261
2.054862	down	NM_176079	Myod1	337868
3.4736204	down	XM_001075328	Myoz2	295426
2.1471794	down	XM_001080508	Mypn	309760
2.0209382	down	XM_001063777	Myst3	306571
2.080869	down	NM_001024765	Nbr1	303554
2.0496933	down	NM_133320	Ndel1	170845
2.1773753	down	XM_216878	Nedd1	299730
4.017796	down	XM_001057566	Nek9	299204
2.0575132	down	NM_012988	Nfia	25492
2.211351	down	XM_001056399	Nfrkb	315523

2.455029	down	NM_080577	Nploc4	140639
2.3020527	down	NM_012613	Npr1	24603
2.1434264	down	NM_013131	Nr3c2	25672
2.4721563	down	NM_024388	Nr4a1	79240
2.8878407	down	XM_001056969	Nrbp2	680451
2.7377768	down	NM_207607	Ns5atp4	311934
2.1663113	down	NM_001034152	Nsg2	497878
2.7653756	down	NM_053463	Nucb1	84595
2.4080863	down	XM_001068048	Numa1	308870
2.1224606	down	NM_053322	Nup210	58958
2.9780726	down	XM_001076876	Obscn	338458
2.2627285	down	XM_001077384	Odf3l2	299600
2.0455365	down	NM_017107	Ogt	26295
2.0298595	down	NM_001000853	Olr803	405143
2.6856673	down	NM_001047897	Ormdl3	360618
2.518035	down	NM_001013079	Osbpl2	296461
2.1005802	down	XM_001074167	Otud1	498803
2.4003084	down	NM_080780	P2rx5	113995
2.5961835	down	NM_012998	P4hb	25506
2.0322366	down	NM_001008328	Parp3	300985
2.2188487	down	NM_001002828	Pbx2	406164
2.9202118	down	XM_001071111	Pcbp4	363133
2.0463147	down	NM_031077	Pctk1	81741
2.9018579	down	NM_145085	Pcyox1	246302
2.2478485	down	NM_022236	Pde10a	63885
2.0428839	down	NM_001004072	Pdha1	29554
4.259142	down	NM_030872	Pdk2	81530
2.1823444	down	NM_001013231	Pea15a	364052
2.4806743	down	XM_001059705	Phc2	313038
2.7798004	down	NM_022626	Phka1	64561
2.3866038	down	NM_001014152	Phkb	361377
2.257644	down	XM_001054196	Pik3ap1	294048
2.3121724	down	NM_001042505	Pitx2	54284
2.5185435	down	NM_001005560	Pla2g6	360426
2.451639	down	NM_001009353	Pla2g7	301265
3.5024602	down	NM_022401	Plec	64204
2.1088047	down	XM_001055776	Plekhb2	301337
2.7119007	down	NM_053827	Plod1	116552
2.0071428	down	XM_001062456	Pnpla2	361676
2.4227262	down	XM_001059753	Pnpla7	246246
2.8999317	down	XM_001079162	Polr2a	363633
2.1128905	down	NM_001007747	Pomgnt1	362567
2.0795076	down	NM_031576	Por	29441
2.7025092	down	NM_031347	Ppargc1a	83516

2.3407729	down	XM_001066863	Ppme1	361613
2.2434025	down	NM_212542	Ppp1r11	294207
2.7960105	down	NM_017309	Ppp3r1	29748
2.0818691	down	NM_031729	Ppp5c	65179
2.556138	down	XM_001079551	Pptc7	304488
2.5891328	down	NM_023991	Prkaa2	78975
2.3417401	down	XM_001072411	Prkaca	25636
2.4199011	down	NM_001077645	Prkacb	293508
2.342025	down	NM_001007697	Prune	310664
2.0590498	down	XM_001076023	Pskh1	364993
2.8918896	down	XM_001055737	Ptpn3	362524
3.4934504	down	NM_019140	Ptprs	25529
2.2159429	down	NM_001012147	Pxn	360820
3.8704295	down	XM_001056644	R3hdm2	362894
2.7686763	down	NM_001008370	Rab1b-ps1	361706
3.1703956	down	NM_013018	Rab3a	25531
2.0310347	down	XM_001072217	Rabgef1	360797
2.3944821	down	XM_001061761	Rabl3	360720
2.0885766	down	XM_343471	Rad54l2	363135
2.322015	down	XM_001069856	Rapgef2	310533
2.0543914	down	XM_001060956	Rapgef4	252857
2.2197084	down	XM_001074931	Rassf8	312846
2.1535063	down	NM_021764	Rbck1	60383
2.6769733	down	XM_001073426	Rbm24	690139
2.4202452	down	XM_001054287	Rbm38	366262
3.0273027	down	NM_001005882	Rbm47	305340
2.059981	down	XM_001053333	Rc3h2	311909
2.200579	down	XM_001071544	Rcsd1	360872
2.2184396	down	NM_199208	Rdh2	299511
2.072556	down	NM_001013218	Reep6	362835
2.5001817	down	XM_001080107	Rfc5	304528
2.2950673	down	XM_001057069	RGD1305455	288545
2.0475929	down	XM_001067947	RGD1305793	309456
3.5532267	down	XM_001078821	RGD1306074	296601
2.2781284	down	XM_001073356	RGD1306484	363594
2.0834305	down	XM_001077056	RGD1307067	362840
2.0136478	down	NM_001014190	RGD1307218	362518
5.607524	down	XM_001080615	RGD1307222	303300
2.6712885	down	XM_001077827	RGD1307615	362084
3.4033272	down	XM_001058624	RGD1309492	314330
2.1123004	down	XM_001068567	RGD1309821	366360
2.2639506	down	XM_001065132	RGD1310587	360894
2.388834	down	XM_001081569	RGD1311429	360642
2.4418674	down	XM_001059424	RGD1311783	294012

4.9539375	down	XM_001072259	RGD1559590	303889
2.2854726	down	XM_001075526	RGD1559682	361080
2.8192265	down	XM_001057559	RGD1559704	499433
2.952845	down	XM_001078945	RGD1559724	299021
2.583351	down	XM_001060803	RGD1559834	367816
2.6660738	down	XM_001081019	RGD1559940	503115
2.4695218	down	XM_001061907	RGD1559963	310284
2.3503814	down	XM_001073916	RGD1560076	294722
2.0803752	down	XM_001057578	RGD1560099	302388
2.1411493	down	XM_001071572	RGD1560716	298444
7.894291	down	XM_001075200	RGD1560826	295423
2.1948798	down	XM_343494	RGD1561176	363155
2.0521796	down	XM_001075088	RGD1561178	295420
2.1782706	down	XM_001081153	RGD1561317	366191
2.146464	down	XM_001066702	RGD1561318	317568
2.3715818	down	XM_001059755	RGD1561394	311743
2.8438923	down	XM_344159	RGD1561635	364023
4.900081	down	XM_001078200	RGD1561881	293876
3.1257105	down	XM_001070383	RGD1562020	290820
2.7434926	down	XM_001062088	RGD1562143	299504
4.4683475	down	XM_001058517	RGD1562165	366374
3.114225	down	XM_001053572	RGD1562266	364863
3.038725	down	XM_001081708	RGD1562394	364129
2.4676418	down	XM_001059720	RGD1562397	364060
2.8996987	down	XM_001071419	RGD1562489	287250
2.6997879	down	XM_001073075	RGD1562500	498096
2.9723172	down	XM_001080210	RGD1562542	294077
2.5242195	down	XM_001053283	RGD1562658	498278
2.038074	down	XM_001055403	RGD1562755	289656
2.7947726	down	XM_001080078	RGD1562814	366866
2.4669845	down	XM_001071898	RGD1562978	502911
2.369399	down	XM_001074453	RGD1563157	298910
3.8542578	down	XM_577969	RGD1563254	502488
2.0039635	down	XM_001061078	RGD1563579	367874
4.321126	down	XM_001068593	RGD1563636	292466
4.3308387	down	XM_001068508	RGD1563658	366058
2.0861356	down	XM_001070015	RGD1563739	289930
2.4602358	down	XM_001076316	RGD1563757	367030
2.01963	down	XM_001053961	RGD1564071	363748
2.8190625	down	XM_001068761	RGD1564088	291290
2.1528409	down	XM_001074520	RGD1564379	499758
3.15561	down	XM_001060995	RGD1564899	498579
2.9011655	down	XM_001081651	RGD1565238	498019
4.4284315	down	XM_001056445	RGD1565579	367860

2.406553	down	XM_001077692	RGD1565599	502599
2.8010182	down	XM_001059981	RGD1565767	311120
2.738117	down	XM_001063845	RGD1565894	291561
2.9863129	down	XM_235089	RGD1566161	314782
2.1153905	down	XM_001079348	RGD1566207	366139
2.3471327	down	XM_001078793	RGD1566281	364480
2.1280413	down	XM_001056130	RGD1566311	500963
2.1226113	down	NM_139040	RGD621098	207123
2.2826333	down	XM_001081746	Rhbdf2	303690
2.5569365	down	XM_001080699	Rilp	287531
2.2677875	down	NM_212549	Ring1	309626
2.189146	down	NM_001033702	Rnf126	314613
2.0257425	down	XM_001067906	Rnf145	287212
3.1206245	down	XM_001064065	Rnf216	304294
2.008361	down	NM_013022	Rock2	25537
2.6121602	down	XM_001077212	Rpl29-ps2	691206
5.155535	down	NM_031570	Rps7	29258
2.0330422	down	NM_138881	Rsad2	65190
3.706712	down	XM_001059176	Rspo3	498997
2.6692867	down	NM_001008833	RT1-CE10	414792
2.309804	down	NM_001008834	RT1-CE11	414791
2.4001737	down	NM_001008835	RT1-CE12	309600
2.9299657	down	NM_001008845	RT1-CE7	368153
2.5571706	down	NM_206848	RT1-Cl	24977
2.0052907	down	NM_017301	S1pr1	29733
2.146008	down	XM_001067905	Saps3	309144
2.2069728	down	XM_001074344	Scamp3	65169
2.3276858	down	XM_001057528	Scarf1	303313
2.0684173	down	NM_031841	Scd	83792
2.0395026	down	XM_001077553	Scube2	499241
2.4841754	down	XM_001080769	Sec1	308586
2.0438998	down	NM_177933	Sel1l	314352
2.3767114	down	NM_001079942	Sema3b	363142
2.0499961	down	XM_001077367	Sema6d	311384
2.5481954	down	NM_058210	Sf1	117855
2.1666071	down	NM_001077826	Shisa4	360848
2.1058934	down	NM_001006989	Shisa5	301013
2.6348786	down	NM_001015036	Siat7F	407765
3.2194288	down	XM_001068825	Sidt2	315617
2.0491107	down	NM_019229	Slc12a4	29501
2.1602037	down	XM_001066528	Slc12a6	691209
2.9318247	down	NM_017302	Slc16a7	29735
2.0770376	down	NM_022270	Slc22a4	64037
2.991711	down	NM_019269	Slc22a5	29726

2.3954003	down	NM_001017488	Slc24a6	498185
2.3532808	down	XM_342445	Slc25a12	362145
2.763128	down	XM_001069918	Slc25a44	365841
2.7709355	down	XM_001079409	Slc27a4	311839
2.6132743	down	NM_031664	Slc28a2	60423
2.786641	down	NM_031684	Slc29a1	63997
2.336503	down	NM_012751	Slc2a4	25139
2.274299	down	NM_181090	Slc38a2	29642
2.5350485	down	NM_145776	Slc38a3	252919
2.8022072	down	NM_001039196	Slc39a13	295928
2.2420156	down	XM_001080703	Slc43a2	287532
2.2979538	down	NM_017049	Slc4a3	24781
2.1634905	down	NM_130746	Slc5a6	170551
5.3909163	down	NM_017206	Slc6a6	29464
2.6261635	down	NM_017348	Slc6a8	50690
3.584671	down	NM_177481	Slco3a1	140915
2.3531563	down	NM_019349	Slk	54308
3.8178911	down	XM_001077609	Smcr7	497916
2.0926437	down	XM_001053117	Smoc2	292401
2.0900323	down	XM_001053796	Smtnl2	679629
2.046237	down	XM_001066311	Smyd2	289372
3.1299634	down	NM_031688	Sncg	64347
3.0760033	down	XM_001067142	Sntb1	299940
2.1863694	down	XM_217115	Sorl1	300652
3.2666845	down	XM_001061383	Spire1	307348
8.615779	down	XM_001079265	Srl	302948
2.1660442	down	XM_233556	Srrm1	313620
2.4118962	down	XM_001080435	Ssh1	304580
2.0069463	down	NM_175597	Ssx2ip	308023
2.902086	down	NM_031695	St3gal2	64442
2.2006502	down	NM_031337	St3gal5	83505
2.5358582	down	NM_001012085	Stam2	311030
2.038714	down	XM_001081093	Stard7	296128
2.139544	down	XM_001068833	Stard8	312113
2.087413	down	NM_012747	Stat3	25125
2.1930132	down	NM_134466	Stau2	171500
3.014088	down	XM_001069624	Stim1	361618
2.0800164	down	NM_001011965	Stom	296655
2.0041072	down	NM_013038	Stxbp1	25558
2.615189	down	XM_001080796	Supt6h	303281
2.1269457	down	NM_001033868	Surf4	619346
2.0082037	down	XM_001068585	Suv420h1	361688
2.5421133	down	XM_001056293	Svil	361256
2.0095801	down	XM_001060926	Sync	362606

2.5753763	down	NM_053553	Syngr2	89815
2.9847853	down	XM_001055657	Synm	308709
2.3467472	down	XM_001074385	Synpo2	499702
5.6425505	down	XM_001064648	Synpo2l	305675
2.377336	down	XM_001071432	Sypl2	362018
2.333312	down	NM_001012152	Tbc1d14	360956
2.0295544	down	XM_001056572	Tbl1xr1	365755
4.1418185	down	XM_001062167	Tbx15	295315
2.7087653	down	XM_001065059	Tcf3	312451
3.2906473	down	NM_031578	Tesk1	29460
2.951199	down	XM_001081600	Tex2	303611
3.069057	down	XM_001072774	Tfrc	64678
3.2638643	down	NM_012740	Th	25085
2.411838	down	NM_031771	Thbd	83580
3.4522252	down	NM_001013062	Thbs1	445442
2.0306695	down	NM_001017960	Thra	81812
2.4193668	down	NM_001009693	Thrap3	313591
2.259414	down	NM_053773	Tjp2	115769
3.1126647	down	XM_001053379	Tln1	313494
4.274692	down	NM_001007713	Tmbim1	316516
2.7930927	down	XM_001059693	Tmcc2	305095
2.3073614	down	NM_001034949	Tmco4	500573
2.2125404	down	XM_001061814	Tmem129	305458
2.0354877	down	NM_178021	Tmem132a	338474
2.2348864	down	XM_001079995	Tmem143	308593
2.4233341	down	NM_057212	Tmem158	117582
2.195924	down	XM_001076493	Tmem184b	362959
2.1711562	down	XM_001053713	Tmem185a	309357
2.9344053	down	XM_001061935	Tmem63a	289318
2.261035	down	XM_001064861	Tmem63b	363197
2.2083628	down	XM_001081259	Tmx4	296182
2.8539546	down	XM_001067689	Tnfrsf21	316256
2.2361085	down	XM_001080123	Tnks2	309512
7.329537	down	NM_001034105	Tnnc1	290561
6.3649735	down	NM_134388	Tnnt1	171409
2.781977	down	NM_001007146	Tob2	315159
2.308192	down	NM_031357	Tpp1	83534
2.3807926	down	XM_001068491	Trappc9	315059
3.4490924	down	NM_023985	Trib1	78969
2.1735091	down	NM_001025142	Trim35	498538
2.3022277	down	NM_001004090	Tspan5	362048
2.1483507	down	NM_001013214	Ttc4	362556
2.5161896	down	NM_001011995	Tuba1c	300218
2.0540063	down	NM_031614	Txnrd1	58819

3.483637	down	XM_001077658	U2af2	308335
2.4030392	down	XM_001081723	Ube2o	303689
2.2437162	down	NM_001037643	Ube2z	303478
2.3493235	down	XM_001074121	Ubqln4	310633
2.3166728	down	XM_001076724	Unc84b	315135
2.0477154	down	XM_001081707	Unk	360663
2.0759985	down	NM_001001516	Usp19	361190
2.0713022	down	XM_001070037	Usp28	315639
2.2770789	down	XM_001080428	Usp30	304579
2.379507	down	XM_001063162	Usp5	297593
2.00357	down	NM_013090	Vamp1	25624
2.7587414	down	XM_001066524	Vash2	498309
2.4385476	down	XM_001057629	Vcl	305679
2.06068	down	XM_001058013	Vgll2	309772
2.1769507	down	NM_013155	Vldlr	25696
3.604743	down	NM_001013170	Wars	314442
2.3041856	down	NM_001009661	Wbp11	297695
2.6789675	down	NM_138975	Wbp2	192645
2.0867481	down	NM_001014135	Wdr1	360950
2.8853447	down	XM_001075797	Wdr62	308492
2.1431231	down	NM_023975	Wdr7	66031
4.863394	down	XM_001077697	Xirp1	316071
2.0413888	down	XM_001079683	Ybx2	303250
2.0864377	down	NM_019376	Ywhag	56010
3.448511	down	NM_001039338	Zdhhc5	362156
2.0070956	down	XM_001053455	Zfp212	297066
2.1437082	down	NM_022678	Zfp238	64619
2.346958	down	NM_001036626	Zfp36l2	298765
2.6507993	down	NM_001034830	Zfp384	171018
2.2654312	down	XM_001061052	Zfp407	307213
2.4031687	down	NM_203367	Zmynd11	291259
2.2494397	down	XM_230846	Zswim1	311631
2.3455925	down	XM_001080236	Zzef1	287476
